# Pleiotropic shared heritability quantifies the shared genetic variance of common diseases

**DOI:** 10.1038/s41588-026-02607-w

**Published:** 2026-06-09

**Authors:** Yujie Zhao, Benjamin Strober, Kangcheng Hou, Gaspard Kerner, John Danesh, Steven Gazal, Wei Cheng, Michael Inouye, Alkes L. Price, Xilin Jiang

**Affiliations:** 1 Institute of Science and Technology for Brain-Inspired Intelligence, Fudan University, Shanghai, China; 2 Department of Epidemiology, Harvard T.H. Chan School of Public Health, Boston, MA USA; 3 Computational Health Informatics Program, Boston Children’s Hospital, Boston, MA USA; 4 Department of Pediatrics, Harvard Medical School, Boston, MA USA; 5 British Heart Foundation Cardiovascular Epidemiology Unit, Department of Public Health and Primary Care, University of Cambridge, Cambridge, UK; 6 Victor Phillip Dahdaleh Heart and Lung Research Institute, University of Cambridge, Cambridge, UK; 7 British Heart Foundation Centre of Research Excellence, University of Cambridge, Cambridge, UK; 8 National Institute for Health and Care Research Blood and Transplant Research Unit in Donor Health and Behaviour, University of Cambridge, Cambridge, UK; 9 Health Data Research UK Cambridge, Wellcome Genome Campus and University of Cambridge, Cambridge, UK; 10 Department of Human Genetics, Wellcome Sanger Institute, Hinxton, UK; 11 Department of Population and Public Health Sciences, Keck School of Medicine, University of Southern California, Los Angeles, CA USA; 12 Center for Genetic Epidemiology, Keck School of Medicine, University of Southern California, Los Angeles, CA USA; 13 Department of Quantitative and Computational Biology, University of Southern California, Los Angeles, CA USA; 14 State Key Laboratory of Brain Function and Disorders, Fudan University, Ministry of Education, Shanghai, China; 15 Fudan ISTBI—ZJNU Algorithm Centre for Brain-Inspired Intelligence, Zhejiang Normal University, Zhejiang, China; 16 Cambridge Baker Systems Genomics Initiative, Department of Public Health and Primary Care, University of Cambridge, Cambridge, UK; 17 Cambridge Baker Systems Genomics Initiative, Baker Heart and Diabetes Institute, Melbourne, Victoria Australia; 18 Broad Institute of MIT and Harvard, Cambridge, MA USA; 19 Department of Biostatistics, Harvard T.H. Chan School of Public Health, Boston, MA USA; 20 Cambridge Centre for AI in Medicine, University of Cambridge, Cambridge, UK

**Keywords:** Genome-wide association studies, Genome-wide association studies, Genome informatics, Software

## Abstract

The overall contribution of pleiotropy to disease architectures is unknown, as most studies estimate genetic correlations with each auxiliary disease in turn. Here we propose a method—pleiotropic shared heritability with bias correction (PHBC)—to estimate the liability-scale genetic variance of a target disease that is shared with a specific set of auxiliary diseases (${{h}^{2}}_{\mathrm{pleio}}$). PHBC estimates ${{h}^{2}}_{\mathrm{pleio}}$ from a genetic correlation matrix using a Monte Carlo bias correction procedure to account for sampling noise. The average ratio of ${{h}^{2}}_{\mathrm{pleio}}$ to total single-nucleotide polymorphism heritability (${{h}^{2}}_{\mathrm{pleio}}/{h}^{2}$) across 15 UK Biobank diseases (spanning seven disease categories) was 27 ± 3%, increasing to 48 ± 5% when expanding to 62 auxiliary diseases/traits. ${{h}^{2}}_{\mathrm{pleio}}/{h}^{2}$ was broadly distributed across disease categories, decreasing only modestly when removing the most informative auxiliary disease categories. The average ${{h}^{2}}_{\mathrm{pleio}}/{h}^{2}$ was 1.51 ± 0.16-times larger than the proportion of total phenotypic variance explained by auxiliary diseases, implying higher pleiotropy for genetic effects. In summary, roughly half of common disease heritability is pleiotropic with a broad range of diseases.

## Abstract (web summary)

Pleiotropic shared heritability with bias correction (PHBC) estimates the genetic variance of a target disease that is shared with a set of auxiliary diseases. Applying PHBC to diseases in the UK Biobank shows pervasive sharing of genetic etiologies across disease categories.

## Main

Common diseases are highly pleiotropic, and extensive efforts have been made to understand the shared components of disease risk^1–6^. Previous studies have quantified the shared genetic variance between pairs of diseases^7–14^, identified latent genetic components that are shared across many diseases^15–23^ and leveraged the sharing of genetic variant associations across diseases to increase association power^17,19,24–29^. However, we lack a method to estimate the genetic variance of a given target disease that is shared with other diseases/traits.

To address this gap, we introduce pleiotropic shared heritability (${{h}^{2}}_{\mathrm{pleio}}$), which quantifies the genetic variance of a target disease that is shared with a specific set of auxiliary diseases/traits. We develop a method, pleiotropic shared heritability with bias correction (PHBC), to estimate ${{h}^{2}}_{\mathrm{pleio}}$ from genome-wide association study (GWAS) summary statistics. PHBC uses a Monte Carlo bias correction procedure to correct bias from sampling noise in genetic correlation estimates due to finite GWAS sample size. We validate PHBC using extensive simulations and estimate ${{h}^{2}}_{\mathrm{pleio}}$ for 15 diseases in the UK Biobank and, subsequently, for 30 diseases from large GWAS meta-analyses. Finally, we extend PHBC to estimate the liability-scale total phenotypic variance of a target disease that is shared with a set of auxiliary diseases.

## Results

### Definition of pleiotropic shared heritability

**Fig. 1:**
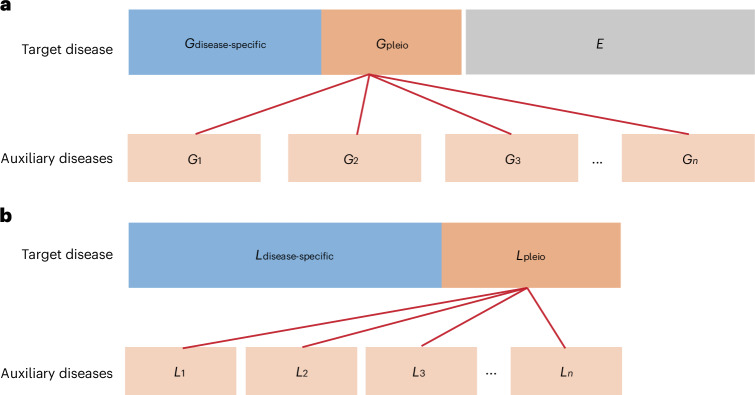
Definition of pleiotropic shared heritability and pleiotropic phenotypic variance. **a**, The total phenotypic variance of the target disease consists of genetic variance (*G*) and environmental variance (*E*). The genetic variance of the target disease is partitioned into a disease-specific component and a pleiotropic component. The disease-specific component is not shared with the auxiliary diseases, and the pleiotropic component consists of a linear combination of the genetic values (${G}_{1}$, ${G}_{2}$, …, ${G}_{n}$) for auxiliary diseases 1 to *n*. Instances of G and E denote vectors of genetic and environmental values across individuals, and areas of rectangles denote variances. **b**, The total phenotypic variance of the target disease is partitioned into a disease-specific component that is not shared with the auxiliary diseases, and a pleiotropic component that is shared with the auxiliary diseases. The pleiotropic phenotypic variance consists of a linear combination of total liabilities (${L}_{1}$, ${L}_{2}$, …, ${L}_{n}$) for auxiliary diseases 1 to *n*. Instances of *L* denote vectors of total liabilities across individuals, and areas of rectangles denote variances.

We define pleiotropic shared heritability as the liability-scale genetic variance (from single-nucleotide polymorphisms, SNPs) of a target disease that is shared with a specific set of auxiliary diseases. We can view the genetic value of the target disease as the sum of a disease-specific genetic value that is not shared with the auxiliary diseases and a pleiotropic genetic value that is shared with auxiliary diseases (Fig. 1a), with the variance of the latter denoting the pleiotropic shared heritability (${{h}^{2}}_{\mathrm{pleio}}$). We use the ratio of pleiotropic shared heritability to total SNP heritability (${{h}^{2}}_{\mathrm{pleio}}/{h}^{2}$) to quantify the proportion of genetic variance (from SNPs) that is pleiotropic. We note that the definition of ${{h}^{2}}_{\mathrm{pleio}}$ depends on both the target disease and the selected set of auxiliary diseases and reflects specific phenotype definitions in specific cohorts.

In detail, ${{h}^{2}}_{\mathrm{pleio}}$ is the liability-scale variance explained by the weighted linear combination of auxiliary disease genetic liabilities that explains the maximum proportion of target-disease heritability (in the entire population with infinite sample size),

1 $${h}_{\mathrm{pleio}}^{2}/{h}^{2}=\mathop{\max }\limits_{{\alpha }_{j}}{r}^{2}\left[\mathop{\sum }\limits_{j=1}^{n}{\alpha }_{j}G_{j},{G}_{T}\right],$$

where ${\alpha }_{j}$ is the weight of the *j*th auxiliary disease $(D_{j})$, *r* is a correlation across individuals, $G_{j}$ is the genetic liability of $D_{j}$ and ${G}_{{T}}$ is the genetic liability of the target disease; ${{h}^{2}}_{\mathrm{pleio}}$ is the corresponding ${r}^{2}$ with the total liability ${L}_{T}$ instead of the genetic liability ${G}_{T}$.

Alternatively, we can define ${{h}^{2}}_{\mathrm{pleio}}/{h}^{2}$ using the proportion of variance of causal SNP effect sizes on the target disease explained by the weighted linear combination of causal SNP effect sizes on the auxiliary diseases that explains the maximum proportion of variance,

2 $${{h}^{2}}_{\mathrm{pleio}}/{h}^{2}=\mathop{\max }\limits_{{\alpha }_{j}}{r}^{2}\left[\mathop{\sum }\limits_{j=1}^{n}{\alpha }_{j}{\beta }_{j},{\beta }_{T}\right],$$

where ${\alpha }_{j}$ is the weight for the *j*th auxiliary disease $(D_{j})$, *r* is a correlation across SNPs, ${\beta }_{j}$ is the vector of standardized causal SNP effect sizes on $D_{j}$ (the number of s.d. increase in phenotype per 1 s.d. increase in genotype) and ${\beta }_{T}$ is the vector of standardized causal SNP effect sizes on the target disease. We note that, under the assumption of no correlation between causal effect sizes of SNPs in linkage disequilibrium (LD) with each other, the two definitions are equivalent (Methods).

We note that when an auxiliary disease $D_{j}$ has zero genetic correlation with the target disease, its contribution to ${{h}^{2}}_{\mathrm{pleio}}$ of the target disease is zero. We emphasize that ${{h}^{2}}_{\mathrm{pleio}}$ is different from ${h}^{2}$ contributed by pleiotropic variants; if an auxiliary disease shares all causal variants with the target disease but with zero genetic correlation, its contribution to ${{h}^{2}}_{\mathrm{pleio}}$ of the target disease is zero, but 100% of the target disease ${h}^{2}$ is contributed by pleiotropic variants. In this study, we restrict both ${{h}^{2}}_{\mathrm{pleio}}$ and total ${h}^{2}$ to the heritability contributed by common SNPs (see below).

### Overview of estimation of pleiotropic shared heritability

We estimate ${{h}^{2}}_{\mathrm{pleio}}$ from estimates of total SNP heritability and genetic correlations between the target and auxiliary diseases, and use a Monte Carlo bias correction procedure to obtain unbiased estimates. We emphasize the need for a bias correction procedure: for example, if the target disease has a true genetic correlation of 0 with each auxiliary disease (implying true ${{h}^{2}}_{\mathrm{pleio}}$ of 0), genetic correlation estimates will be nonzero owing to sampling noise, implying an ${{h}^{2}}_{\mathrm{pleio}}$ estimate greater than 0 without bias correction. Monte Carlo sampling is a widely used method that relies on random sampling to capture properties of a noise distribution^30^. In our application, Monte Carlo sampling captures the noise distribution of the noisily estimated genetic correlations; when the sampled genetic correlations are used to estimate ${{h}^{2}}_{\mathrm{pleio}}/{h}^{2}$, the resulting distribution captures the bias in ${{h}^{2}}_{\mathrm{pleio}}/{h}^{2}$ arising from noisily estimated genetic correlations. Specifically, Monte Carlo sampling is used to estimate the expected ratio between the true value (unbiased) versus initial estimate (biased) of ${{h}^{2}}_{\mathrm{pleio}}/{h}^{2}$, so that the initial estimate can be scaled by this ratio to obtain an unbiased estimate. In this study, we apply cross-trait LD score regression (LDSC)^8^ to summary statistics and reference LD to estimate genetic correlations; we note that the cross-trait LDSC estimand is the genetic correlation of causal effect sizes of common SNPs (Methods). We obtain standard errors via genomic block-jackknife, as in cross-trait LDSC^8^, and scale standard errors using Monte Carlo samples of estimation errors to account for the impact of our bias correction procedure (see below). We have publicly released open-source software implementing our method (see ‘Code availability’ section).

In detail, it can be shown that equation (2) is equivalent to (Methods)

3 $${{h}^{2}}_{\mathrm{pleio}}/{h}^{2}={r}_{g}{\left[{\bf{D}},T\,\right]}^{T}{r}_{g}{\left[{\bf{D}},{\bf{D}}\right]}^{-1}{r}_{g}[{\bf{D}},T\,],$$

where ${h}^{2}$ is the SNP heritability of the target disease, ${r}_{g}\left[{\bf{D}},T\right]$ is the *n* × 1 vector of genetic correlations between the target and auxiliary diseases, and ${r}_{g}[{\bf{D}},{\bf{D}}]$ is the *n* × *n* genetic correlation matrix among the set of auxiliary diseases, where *n* is the number of auxiliary diseases. We initially estimate ${{h}^{2}}_{\mathrm{pleio}}/{h}^{2}$ by estimating the corresponding quantities in equation (3). However, the errors in estimates of ${r}_{g}\left[{\bf{D}},{\bf{D}}\right]$ and ${r}_{g}[{\bf{D}},T]$ in finite GWAS sample size introduce an upward bias in estimates of ${{h}^{2}}_{\mathrm{pleio}}/{h}^{2}$, if ${r}_{g}{\left[{\bf{D}},{\bf{D}}\right]}^{-1}$ is positive definite (which is likely to be the case; Methods).

To obtain an unbiased estimate, we first generate Monte Carlo samples of estimation errors in ${r}_{g}\left[{\bf{D}},{\bf{D}}\right]$ and ${r}_{g}[{\bf{D}},T]$ from the sampling covariance matrix computed from genomic block-jackknife^17^ (we use the same genomic block-jackknife for the target disease *T* and all auxiliary diseases **D**; see ‘Code availability’ section); these samples capture the joint distribution of estimation errors across elements in ${r}_{g}\left[{\bf{D}},{\bf{D}}\right]$ and ${r}_{g}[{\bf{D}},T]$. Then, we add the estimation errors to the point estimates of ${r}_{g}\left[{\bf{D}},{\bf{D}}\right]$ and ${r}_{g}[{\bf{D}},T]$ to create Monte Carlo samples of ${{h}^{2}}_{\mathrm{pleio}}/{h}^{2}$, which are larger than the initial estimate. We then use binary search to estimate a scaling coefficient ${\xi }_{c}$ multiplying ${r}_{g}\left[{\bf{D}},T\right]$ so that the average ${{h}^{2}}_{\mathrm{pleio}}/{h}^{2}$ across Monte Carlo samples matches the initial estimate of ${{h}^{2}}_{\mathrm{pleio}}/{h}^{2}$ (Methods). Finally, we multiply the initial estimate of ${{h}^{2}}_{\mathrm{pleio}}/{h}^{2}$ by ${\xi }_{c}^{2}$ to obtain an unbiased estimate of ${{h}^{2}}_{\mathrm{pleio}}/{h}^{2}$. To obtain a corrected s.e. for the bias-corrected estimates of ${{h}^{2}}_{\mathrm{pleio}}/{h}^{2}$, we scale the jackknife s.e. of the uncorrected estimate of ${{h}^{2}}_{\mathrm{pleio}}/{h}^{2}$ by the ratio of the s.d. of corrected estimates across Monte Carlo samples divided by the s.d. of uncorrected estimates across Monte Carlo samples (Methods).

In the presence of collinearity among auxiliary diseases and uncertainty in genetic correlation estimates, the estimate from equation (3) can be numerically unstable, which we define as s.e. (${r}_{g}{\left[{\bf{D}},T\right]}^{T}{r}_{g}{\left[{\bf{D}},{\bf{D}}\right]}^{-1}{r}_{g}\left[{\bf{D}},T\right]$) >0.5 and/or ${\xi }_{c} < 0.5$ (Methods). Whenever this occurs, we prune auxiliary traits as follows: for each pair of auxiliary traits with ${r}_{g}^{2} > 0.5$, we remove the trait with the lower ${r}_{g}$ *z* score with the target trait; we repeat this procedure for ${r}_{g}^{2}$ thresholds of 0.5, 0.4, 0.3, 0.2 and 0.1 until the estimate is numerically stable.

We extend our method to estimate the liability-scale total phenotypic variance of a target disease that is shared with a specific set of auxiliary diseases (${{V}^{2}}_{\mathrm{pleio}}$) by estimating the liability-scale phenotypic correlation between pairs of diseases using individual-level data^31^ (Fig. 1b). We note that the term pleiotropy generally refers to genetic effects, but we use the term pleiotropic phenotypic variance for consistency. Further details are provided in Methods.

An overview of empirical data that were analyzed is as follows. We first analyzed 15 highly heritable target diseases (prevalence >1%, heritability *z* score >6 and liability-scale heritability ranging from 0.061 to 0.21) from the UK Biobank^32^ (average *n* = 157k, spanning seven PheCode disease categories^33^; Table 1); the sample size reflects a restriction to unrelated individuals of British ancestry with diagnostic records from both primary care data and hospital inpatient data, which we imposed to mitigate the impact of missing diagnoses. In UK Biobank analyses, we used the same 15 diseases as auxiliary diseases; we also performed analyses incorporating 17 quantitative traits^34,35^ from the UK Biobank as auxiliary traits (including blood biochemistry measurements, for example, total cholesterol, blood pressure and hemoglobin A1c (HbA1c), and demographic traits, for example, height, body mass index (BMI) and years of education; details in Supplementary Table 2 and Methods). We subsequently incorporated 30 diseases with large meta-analyses available (average *n* = 483k) as target and/or auxiliary diseases. In all analyses, we restricted to auxiliary diseases with modest genetic correlation to the target disease (${{r}_{g}}^{2} < 0.5$). We note that while we elected to focus on target diseases, definition and estimation of ${{h}^{2}}_{\mathrm{pleio}}$ are also applicable to target quantitative traits. We recommend that users restrict to target and auxiliary traits that have a heritability *z* score >6 and auxiliary traits that have an ${{r}_{g}}^{2} < 0.5$ with the target trait (Methods).

**Table 1:** Overview of 15 UK Biobank diseases

Disease	Prevalence	${h}_{\mathrm{liab}}^{2}$ (s.e.)	*h*^2^ *z* score	Category
Hypothyroidism	0.054	0.18 (0.024)	7.7	Endocrine
Type 2 diabetes	0.11	0.19 (0.014)	13	Endocrine
Obesity	0.20	0.14 (0.0087)	16	Endocrine
Depression	0.17	0.087 (0.0082)	11	Mental disorders
Tobacco use disorder	0.049	0.14 (0.017)	7.8	Mental disorders
Hypertension	0.37	0.21 (0.011)	19	Circulatory system
Coronary atherosclerosis	0.083	0.16 (0.015)	11	Circulatory system
Atrial fibrillation	0.051	0.12 (0.019)	6.2	Circulatory system
Varicose veins	0.069	0.12 (0.017)	6.9	Circulatory system
Respiratory diseases	0.30	0.061 (0.0061)	10	Respiratory
GERD	0.17	0.067 (0.0075)	9.0	Digestive
Inguinal hernia	0.074	0.11 (0.015)	7.1	Digestive
Diverticulosis	0.15	0.13 (0.010)	13	Digestive
Bunion	0.048	0.15 (0.018)	8.2	Musculoskeletal
Radiculitis	0.10	0.073 (0.010)	7.0	Symptoms

We report the names of 15 UK Biobank diseases, prevalence in the UK Biobank, liability-scale heritability, heritability *z* score and the PheCode disease category. ${h}_{\mathrm{liab}}^{2}$ (s.e.) denotes the liability-scale heritability and its standard error. Full details are presented in Supplementary Table 1. GERD, gastroesophageal reflux disease.

### Simulations

We performed simulations to evaluate the unbiasedness of pleiotropic shared heritability estimates. We specified genetic architectures for 16 diseases using liability-scale heritabilities of 0.13 (median of 15 UK Biobank diseases; Table 1) and prevalences of 0.1 (median of 15 UK Biobank diseases; Table 1). True genetic correlations were set to 0.5 within disease categories and 0.1 between disease categories for the first 15 diseases (based on the seven disease categories from Table 1) and 0.0 for the last disease (with each other disease), implying five different values of true ${{h}^{2}}_{\mathrm{pleio}}/{h}^{2}$ for each target disease (ranging from 0.00 to 0.38). The proportion of causal SNPs was set to 5%. Additional simulation settings were also evaluated. We simulated phenotypes from real UK Biobank genotypes using 1.1M HapMap3^36^ SNPs across 157,206 unrelated individuals. We applied PLINK2^37^ to compute summary statistics and applied cross-trait LDSC^8^ to summary statistics for the 1.1M SNPs (minor allele frequency (MAF) >0.01) and reference LD from 1000 Genomes European individuals (9.3M SNPs) to estimate genetic correlations. We then estimated ${{h}^{2}}_{\mathrm{pleio}}$ for each disease using the other 15 diseases as auxiliary diseases. Further details of the simulation framework are provided in Methods.

**Fig. 2:**
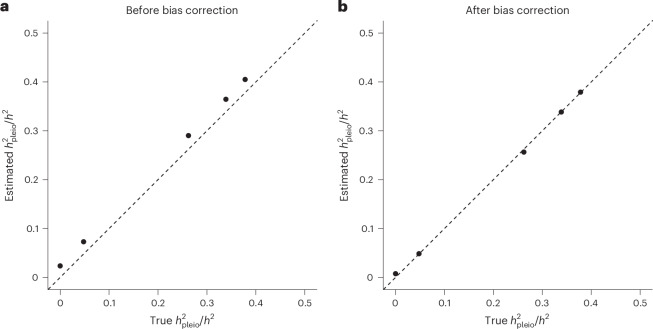
PHBC corrects upwards bias in simulations. **a**, Estimates of ${{h}^{2}}_{\mathrm{pleio}}/{h}^{2}$ without the bias correction step suffer upward bias. **b**, Estimates of ${{h}^{2}}_{\mathrm{pleio}}/{h}^{2}$ are approximately unbiased after the Monte Carlo bias correction. Each dot represents the mean of all estimates for diseases with the same true value of ${{h}^{2}}_{\mathrm{pleio}}/{h}^{2}$ across 100 simulations. Error bars denote s.e.m. (but are generally smaller than dot size). Numerical results are presented in Supplementary Table 4.

Results are shown in Fig. 2 and presented in Supplementary Table 4. Before Monte Carlo bias correction, ${{h}^{2}}_{\mathrm{pleio}}/{h}^{2}$ estimates suffered upward biases (between 0.024 (s.e.m. 0.0012) and 0.028 (s.e.m. 0.0034); average bias of 0.026 (s.e.m. 0.0010)), highlighting the need for a bias correction procedure. After Monte Carlo bias correction, ${{h}^{2}}_{\mathrm{pleio}}/{h}^{2}$ estimates were approximately unbiased across the 16 simulated diseases with varying pleiotropic architectures. Most biases were small (between −0.006 (s.e.m. 0.003) and 0.008 (s.e.m. 0.0006); average bias of 0.0006 (s.e.m. 0.0009)). We determined that the estimated standard errors were approximately well calibrated, with the ratio of estimated versus true errors reasonably close to 1 (1.07 or 0.95 or 1.27 or 0.93, varying across four ways to compute this ratio; details in Supplementary Fig. 1 and Supplementary Table 4 captions).

We performed six secondary analyses. First, we simulated pleiotropic architectures with ${r}_{g}$ within disease categories equal to 0.4, 0.3 or 0.2 (instead of 0.5) (and ${r}_{g}$ between disease categories still equal to 0.1), and we observed approximately unbiased results (Supplementary Figs. 2–7 and Supplementary Tables 5–7). Second, we performed simulations with liability-scale heritabilities equal to 0.25 or 0.06 (instead of 0.13). We observed approximately unbiased results with liability-scale heritability equal to 0.25 (Supplementary Figs. 8 and 9 and Supplementary Table 8). With liability-scale heritability equal to 0.06, we observed modest downward bias for values of ${{h}^{2}}_{\mathrm{pleio}}/{h}^{2}$ above 25% and modest upward bias for values below 5%, with average bias of −0.011 (s.e.m. 0.0021) (Supplementary Figs. 10 and 11 and Supplementary Table 9). Third, we performed simulations to evaluate the genomic block-jackknife standard errors on the reduction in ${{h}^{2}}_{\mathrm{pleio}}/{h}^{2}$ in analyses with one auxiliary disease category removed, and determined that standard errors were approximately well calibrated (Supplementary Fig. 19 and Supplementary Table 13). Additional secondary analyses are described in Supplementary Notes, Supplementary Figs. 12–17 and Supplementary Tables 10–12.

### Application to 15 diseases from the UK Biobank

**Fig. 3:**
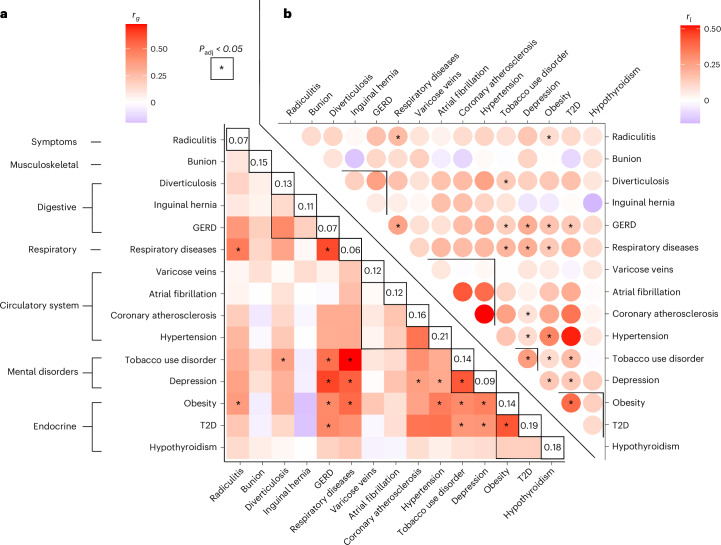
Estimates of genetic correlation and liability-scale phenotypic correlation. **a**, Estimates of genetic correlation across 15 UK Biobank diseases. **b**, Estimates of liability-scale phenotypic correlation across 15 UK Biobank diseases. We performed a one-sided Wald *z* test to assess whether the genetic correlation was significantly greater than the liability-scale phenotypic correlation. Asterisks denote pairs of diseases for which the genetic correlation is significantly larger than the liability-scale phenotypic correlation with Bonferroni-corrected one-sided *P* < 4.76 × 10^−4^ (= 0.05/105) for multiple comparisons; no pairs have liability-scale phenotypic correlation significantly larger than the genetic correlation. Black boundaries demarcate correlations between diseases within the same disease category. Numerical results are presented in Supplementary Table 15.

We estimated ${{h}^{2}}_{\mathrm{pleio}}/{h}^{2}$ for 15 highly heritable target diseases from the UK Biobank (heritability *z* score >6; average *n* = 157k, liability-scale heritability ranging from 0.061 to 0.21, spanning seven PheCode disease categories^33^; Table 1). Genetic correlations across these 15 diseases are shown in Fig. 3a and Supplementary Table 14, and corresponding liability-scale phenotypic correlations are shown in Fig. 3b and Supplementary Table 15. We observed moderate genetic correlations within disease categories and between disease categories as well as smaller phenotypic correlations.

**Fig. 4:**
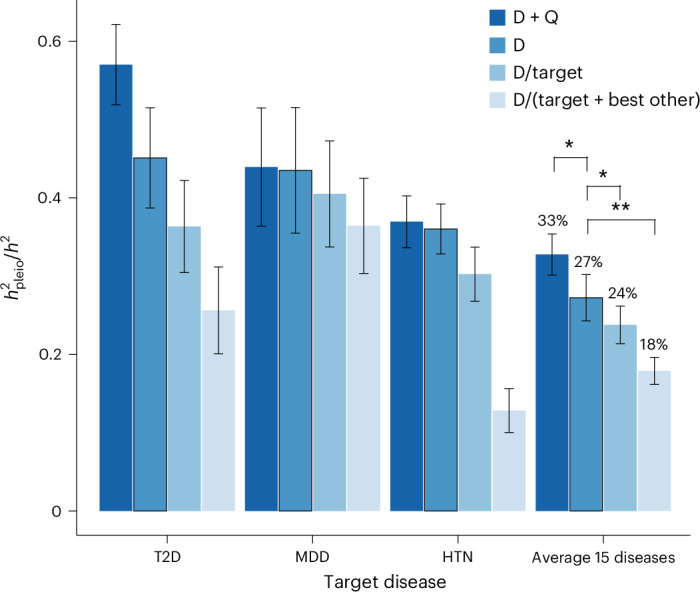
*h*^2^_pleio_/*h*^2^ estimates for three representative diseases and the average across 15 UK Biobank diseases. D + Q: ${{h}^{2}}_{\mathrm{pleio}}/{h}^{2}$ estimates with respect to 15 UK Biobank auxiliary diseases and 17 UK Biobank quantitative auxiliary traits. D: ${{h}^{2}}_{\mathrm{pleio}}/{h}^{2}$ estimates with respect to 15 UK Biobank auxiliary diseases. D/target: ${{h}^{2}}_{\mathrm{pleio}}/{h}^{2}$ estimates with respect to 15 UK Biobank auxiliary diseases excluding those from the same disease category as the target disease. D/(target + best other): ${{h}^{2}}_{\mathrm{pleio}}/{h}^{2}$ estimates with respect to 15 UK Biobank auxiliary diseases excluding those from the same disease category as the target disease and those from one other disease category whose removal had the greatest impact. Data are presented as point estimate ± s.e. Error bars denote jackknife standard errors obtained via 200 genomic blocks. To assess statistical significance, we compare average estimates from D + Q, D/target and D/(target + best other) to estimates from D as the control group using inverse-variance weighted Wald *z* test across diseases/traits. *two-sided *P* < 0.05; **two-sided *P* < 0.001. Numerical results are presented in Supplementary Tables 17 and 18.

**Extended Data Fig. 1:**
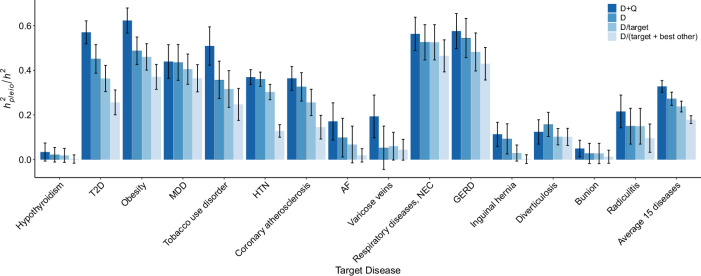
*h*^2^_*pleio*_/*h*^2^ after bias correction for all 15 UK Biobank diseases and the average. D + Q: ${{h}^{2}}_{{pleio}}/{h}^{2}$ estimates with respect to 14 UK Biobank auxiliary diseases and 17 UK Biobank quantitative auxiliary traits. D: ${{h}^{2}}_{{pleio}}/{h}^{2}$ estimates with respect to 14 UK Biobank auxiliary diseases. D/target: ${{h}^{2}}_{{pleio}}/{h}^{2}$ estimates with respect to 14 UK Biobank auxiliary diseases excluding those from the same disease category as the target disease. D/(target+best other): ${{h}^{2}}_{{pleio}}/{h}^{2}$ estimates with respect to 14 UK Biobank auxiliary diseases excluding those from the same disease category as the target disease and from the next disease category whose removal had the greatest impact. Data are presented as point estimate ± s.e. Error bars represent jackknife standard errors obtained via 200 genomic blocks. We compute the post-bias-correction standard error of the average estimate across 15 diseases using the average of ratio to scale the uncorrected jackknife s.e. of average: ($\frac{1}{15}{\sum }_{i=1}^{15}\frac{{post}-{correction\; s}.e{.}_{i}}{{pre}-{correction\; s}.e{.}_{i}}$) × uncorrected jackknife s.e. of average. Detailed results are provided in Supplementary Table 17. T2D, type 2 diabetes; MDD, depression; HTN, hypertension; AF, atrial fibrillation; GERD, gastroesophageal reflux disease.

**Extended Data Fig. 2:**
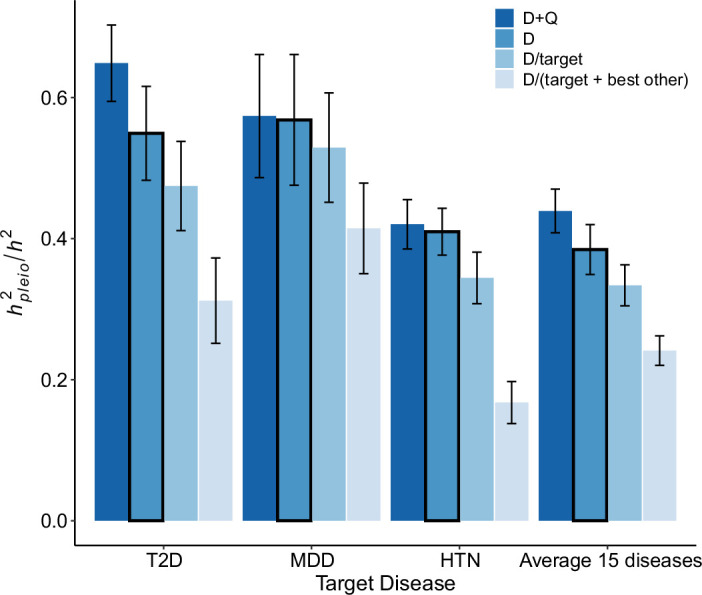
*h*^2^_*pleio*_/*h*^2^ before bias correction for three representative diseases and the average across 15 UK Biobank diseases. Comparing to Fig. 4, we observed an upward bias for ${{h}^{2}}_{{pleio}}/{h}^{2}$ before bias correction. D + Q: ${{h}^{2}}_{{pleio}}/{h}^{2}$ estimates with respect to 14 UK Biobank auxiliary diseases and 17 UK Biobank quantitative auxiliary traits. D: ${{h}^{2}}_{{pleio}}/{h}^{2}$ estimates with respect to 14 UK Biobank auxiliary diseases. D/target: ${{h}^{2}}_{{pleio}}/{h}^{2}$ estimates with respect to 14 UK Biobank auxiliary diseases excluding those from the same disease category as the target disease. D/(target+best other): ${{h}^{2}}_{{pleio}}/{h}^{2}$ estimates with respect to 14 UK Biobank auxiliary diseases excluding those from the same disease category as the target disease and from the next disease category whose removal had the greatest impact. Data are presented as point estimate ± s.e. Error bars represent jackknife standard errors obtained via 200 genomic blocks. Detailed results are provided in Supplementary Table 17. T2D, type 2 diabetes; MDD, depression; HTN, hypertension.

Estimates of ${{h}^{2}}_{\mathrm{pleio}}/{h}^{2}$ for three representative diseases (type 2 diabetes (T2D), major depressive disorder (MDD) and hypertension (HTN)), and the average across 15 diseases, are shown in Fig. 4, Extended Data Fig. 1 and Supplementary Table 17. We determined that 27% of SNP-heritability is pleiotropic on average (jackknife s.e. (average) = 3%, s.d. = 19% across diseases); the Monte Carlo bias correction procedure had a substantial impact on estimates of ${{h}^{2}}_{\mathrm{pleio}}/{h}^{2}$ (pre-correction average = 38%, jackknife s.e. (average) = 4%; Extended Data Fig. 2 and Supplementary Table 17), consistent with simulations (Fig. 2) and underscoring the importance of correcting upward bias. Several diseases had fairly high ${{h}^{2}}_{\mathrm{pleio}}/{h}^{2}$ estimates, including 44% (s.e. 8%) for MDD and 45% (s.e. 6%) for T2D.

We performed three analyses in which we modified the set of auxiliary diseases (Fig. 4, Extended Data Fig. 1 and Supplementary Table 17). First, we additionally included 17 auxiliary quantitative traits from the UK Biobank (Supplementary Table 2), causing ${{h}^{2}}_{\mathrm{pleio}}/{h}^{2}$ estimates to increase (average = 33%, jackknife s.e. (average) = 3%, s.d. = 21%). Second, we restricted to auxiliary diseases excluding the target disease category, causing ${{h}^{2}}_{\mathrm{pleio}}/{h}^{2}$ estimates to decrease only slightly (average = 24% (s.e. 2%); 41% (s.e. 7%) for MDD (removing mental) and 36% (s.e. 6%) for T2D (removing endocrine)). Third, we further removed one other disease category whose removal had the greatest impact, causing ${{h}^{2}}_{\mathrm{pleio}}/{h}^{2}$ estimates to decrease only moderately (average = 18% (s.e. 2%); 36% (s.e. 6%) for MDD (removing mental and digestive) and 26% (s.e. 6%) for T2D (removing endocrine and circulatory system)). These results imply that ${{h}^{2}}_{\mathrm{pleio}}$ is broadly distributed across disease categories.

**Fig. 5:**
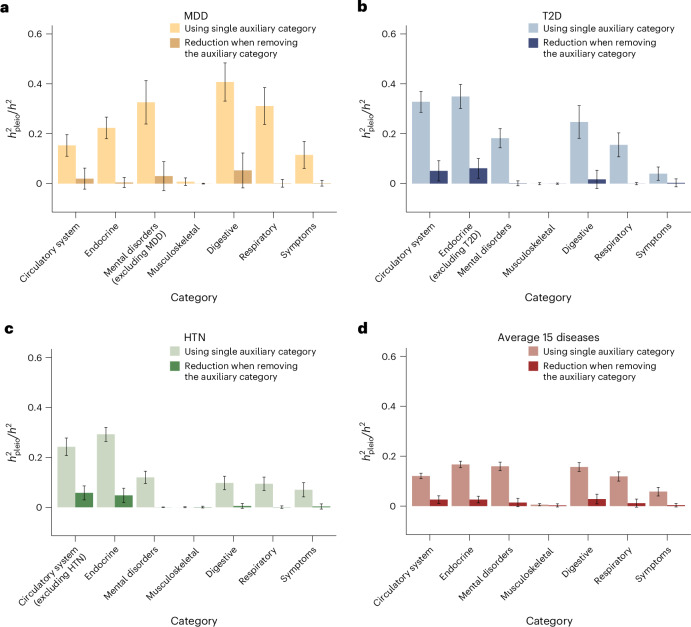
*h*^2^_pleio_/*h*^2^ is broadly shared across PheCode disease categories. **a**–**d**, Comparison between ${{h}^{2}}_{\mathrm{pleio}}/{h}^{2}$ using a single auxiliary disease category (light bars) and the reduction in ${{h}^{2}}_{\mathrm{pleio}}/{h}^{2}$ when removing the auxiliary disease category (dark bars), for three representative UK Biobank diseases (MDD, T2D and HTN) and the average across 15 UK Biobank diseases. Data are presented as point estimate ± s.e. Error bars denote jackknife standard errors obtained via 200 genomic blocks. Numerical results are presented in Supplementary Table 19.

Estimates of ${{h}^{2}}_{\mathrm{pleio}}/{h}^{2}$ with respect to auxiliary diseases in each individual disease category are shown in Fig. 5, Supplementary Fig. 20 and Supplementary Table 19. We determined that auxiliary diseases from a single disease category can often explain the majority of ${{h}^{2}}_{\mathrm{pleio}}$. For example, MDD had 41% (s.e. 8%) and 31% (s.e. 7%) of SNP heritability shared with digestive and respiratory disease categories, respectively (versus 44% for all disease categories, consistent with the high pleiotropic overlap between brain–gut and brain–lung axes in previous studies^38,39^); T2D had 33% (s.e. 4%) and 25% (s.e. 7%) of SNP heritability shared with circulatory and digestive disease categories, respectively (versus 45% for all disease categories, consistent with the importance of T2D-related processes in the etiology of cardiometabolic diseases in previous studies^22^); and the 15 target diseases on average had 17% (s.e. 1%) and 16% (s.e. 2%) of SNP heritability shared with endocrine and mental disorder categories, respectively (versus 27% for all disease categories).

However, removing a single disease category from the set of auxiliary diseases generally reduced ${{h}^{2}}_{\mathrm{pleio}}/{h}^{2}$ much less than the ${{h}^{2}}_{\mathrm{pleio}}/{h}^{2}$ shared with that disease category (Fig. 5, Supplementary Fig. 20 and Supplementary Table 19). For example, MDD ${{h}^{2}}_{\mathrm{pleio}}/{h}^{2}$ was reduced by only 5% (s.e. 7%) and 0.1% (s.e. 1.5%) when removing digestive and respiratory categories, respectively; T2D ${{h}^{2}}_{\mathrm{pleio}}/{h}^{2}$ was reduced by only 5% (s.e. 4%) and 2% (s.e. 4%) when removing circulatory and digestive disease categories, respectively; and average ${{h}^{2}}_{\mathrm{pleio}}/{h}^{2}$ across the 15 target diseases was reduced by only 3% (s.e. 1%) and 1% (s.e. 2%) when removing endocrine and mental disorder categories, respectively. These results imply that ${{h}^{2}}_{\mathrm{pleio}}/{h}^{2}$ is broadly shared across disease categories, such that the removal of one disease category has a limited impact owing to shared ${{h}^{2}}_{\mathrm{pleio}}/{h}^{2}$ with other disease categories.

**Extended Data Fig. 3:**
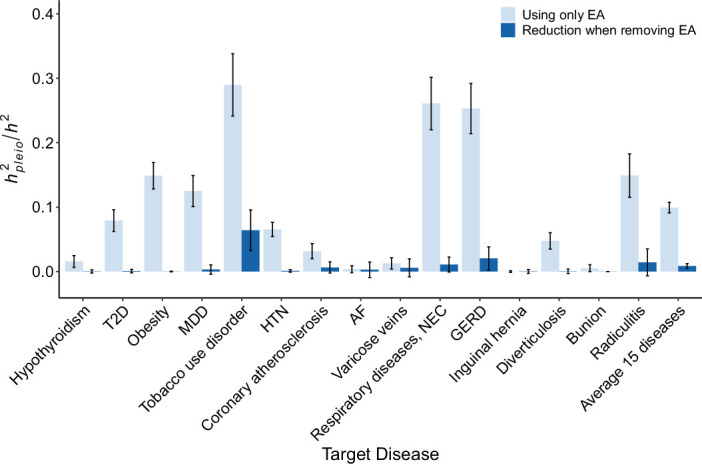
The impact of educational attainment (EA) on pleiotropy. We performed two analyses. First, we estimated ${{h}^{2}}_{{pleio}}/{h}^{2}$ with respect to years of education as the only auxiliary trait for each of the 15 UK Biobank diseases. Light color bar is the ${{h}^{2}}_{{pleio}}/{h}^{2}$ using only EA as auxiliary trait with average of 9.9% (s.e. 0.8%). Second, we assessed the impact of removing EA from the set of auxiliary traits by estimating the difference between (i) ${{h}^{2}}_{{pleio}}/{h}^{2}$ with respect to 15 UK Biobank auxiliary diseases + 17 UK Biobank auxiliary quantitative traits vs. (ii) ${{h}^{2}}_{{pleio}}/{h}^{2}$ with respect to 15 UK Biobank auxiliary diseases + 16 UK Biobank quantitative traits excluding EA. Dark color bar is the reduction of ${{h}^{2}}_{{pleio}}/{h}^{2}$ when removing EA from the auxiliary trait set with average of 0.87% (s.e. 0.38%). Data are presented as point estimate ± s.e. Error bars represent jackknife standard errors obtained via 200 genomic blocks. We reported s.e. using jackknife standard error of the $\frac{{{h}^{2}}_{{pleio}}}{{h}^{2}}$ reduction before bias correction. We compute the post-bias-correction standard error of the average estimate across 15 diseases using the average of ratio to scale the uncorrected jackknife s.e. of average: ($\frac{1}{15}{\sum }_{i=1}^{15}\frac{{post}-{correction\; s}.e{.}_{i}}{{pre}-{correction\; s}.e{.}_{i}}$) × uncorrected jackknife s.e. of average. Detailed results are provided in Supplementary Table 28. T2D, type 2 diabetes; MDD, depression; HTN, hypertension; GERD, gastroesophageal reflux disease; AF, atrial fibrillation.

To investigate the impact of educational attainment (EA) on ${{h}^{2}}_{\mathrm{pleio}}/{h}^{2}$, we performed two analyses. First, we estimated ${{h}^{2}}_{\mathrm{pleio}}/{h}^{2}$ with respect to EA (years of education) as the only auxiliary trait. We determined that ${{h}^{2}}_{\mathrm{pleio}}/{h}^{2}$ with respect to EA is 9.9% (s.e. 0.8%) on average across 15 UK Biobank diseases (Extended Data Fig. 3 and Supplementary Table 28). Second, we assessed the impact of removing EA from the set of auxiliary traits by estimating the difference between (1) ${{h}^{2}}_{\mathrm{pleio}}/{h}^{2}$ with respect to 15 UK Biobank auxiliary diseases and 17 UK Biobank auxiliary quantitative traits versus (2) ${{h}^{2}}_{\mathrm{pleio}}/{h}^{2}$ with respect to 15 UK Biobank auxiliary diseases and 16 UK Biobank quantitative traits excluding EA. We determined that the differences were small, with an average reduction of 0.87% (s.e. 0.38%) (Extended Data Fig. 3 and Supplementary Table 28). This implies that the large contribution of EA to ${{h}^{2}}_{\mathrm{pleio}}/{h}^{2}$ is mostly captured by other auxiliary traits.

**Extended Data Fig. 4:**
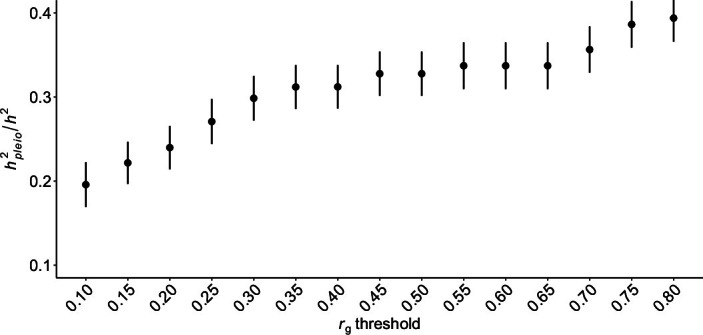
Changes in average *h*^2^_*pleio*_/*h*^2^ across 15 UK Biobank diseases for *r*_*g*_^2^ thresholds between target and auxiliary diseases ranging from 0.1 to 0.8 (step = 0.05). Average ${{h}^{2}}_{{pleio}}/{h}^{2}$ increased at ${{r}_{g}}^{2}$ thresholds larger than 0.5, but we consider this increase to be not biologically meaningful because most instances of ${{r}_{g}}^{2}$ > 0.5 involved auxiliary traits that *are not biologically distinct* from the target trait, for example hypertension−diastolic blood pressure (${{r}_{g}}^{2}=0.72$), hypertension−systolic blood pressure (${{r}_{g}}^{2}=0.72$), type 2 diabetes−glucose (${{r}_{g}}^{2}=0.69$), type 2 diabetes−HbA1C (${{r}_{g}}^{2}=0.79$). In addition, average ${{h}^{2}}_{{pleio}}/{h}^{2}$ decreased at ${{r}_{g}}^{2}$ thresholds smaller than 0.5, but all instances of $0.25 < {{r}_{g}}^{2} < 0.5$ involved auxiliary traits that *are biologically distinct* from the target trait: coronary atherosclerosis−hypertension (${{r}_{g}}^{2}=0.25$), coronary atherosclerosis−type 2 diabetes (${{r}_{g}}^{2}=0.25$), hypertension−type 2 diabetes (${{r}_{g}}^{2}=0.26$), depression−tobacco use disorder (${{r}_{g}}^{2}=0.35$), gastroesophageal reflux disease−tobacco use disorder (${{r}_{g}}^{2}=0.27$), obesity−type 2 diabetes (${{r}_{g}}^{2}=0.36$), obesity−respiratory diseases (${{r}_{g}}^{2}=0.29$), obesity−HbA1c (${{r}_{g}}^{2}=0.26$), obesity−glucose (${{r}_{g}}^{2}=0.26$), type 2 diabetes−body WHR (${{r}_{g}}^{2}=0.36$), type 2 diabetes−BMI (${{r}_{g}}^{2}=0.34$), type 2 diabetes−triglycerides (${{r}_{g}}^{2}=0.31$), gastroesophageal reflux disease−depression (${{r}_{g}}^{2}=0.40$), gastroesophageal reflux disease−respiratory diseases (${{r}_{g}}^{2}=0.39$) and depression−respiratory diseases (${{r}_{g}}^{2}=0.32$)—such that we believe that an estimand based on an ${{r}_{g}}^{2}$ threshold of 0.5 is preferred. Data are presented as point estimate ± s.e. Error bars represent jackknife standard errors obtained via 200 genomic blocks. Detailed genetic correlation results refer to Supplementary Table 29.

We performed six secondary analyses. First, we investigated whether there is a correlation between liability-scale heritability and ${{h}^{2}}_{\mathrm{pleio}}/{h}^{2}$ across the 15 UK Biobank diseases. We observed no significant correlation (Pearson’s *r* = −0.18 (*P* = 0.52); Supplementary Fig. 21). Second, we estimated the average ${{h}^{2}}_{\mathrm{pleio}}/{h}^{2}$ across 15 UK Biobank diseases using different ${{r}_{g}}^{2}$ thresholds between target and auxiliary diseases (ranging from 0.1 to 0.8 in increments of 0.05) (details in Extended Data Fig. 4, Supplementary Notes and Supplementary Table 29), and the results support the use of an estimand based on ${{r}_{g}}^{2} < 0.5$ between target and auxiliary diseases/traits. Third, we compared ${{h}^{2}}_{\mathrm{pleio}}/{h}^{2}$ to the sum of (bias-corrected) ${{r}_{g}}^{2}$ across auxiliary diseases for a given target disease and found that ${{h}^{2}}_{\mathrm{pleio}}/{h}^{2}$ captures different information than the sum of (bias-corrected) ${{r}_{g}}^{2}$ (details in Supplementary Note and Supplementary Tables 30 and 31). Additional secondary analyses are described in Supplementary Note, Supplementary Figs. 22 and 23 and Supplementary Tables 20, 21, 27 and 32.

### Application to 30 diseases from publicly available GWAS meta-analyses

We expanded our analyses to 30 highly heritable diseases with publicly available GWAS summary statistics (heritability *z* score >6; observed-scale heritability under case–control ascertainment ranging from 0.007 to 0.821 (with unknown prevalence); ${{r}_{g}}^{2} < 0.5$; average *n* = 483 K, spanning ten PheCode disease categories^33^) (Supplementary Table 3). These include many diseases (for example, autoimmune diseases and cancers) that were not included in the 15 diseases in the UK Biobank on the basis of the criteria that we applied. For brevity, we subsequently refer to the above 15 as ‘UK Biobank’ diseases and the 30 as ‘non-UK Biobank’ diseases (while duly noting that a subset of the latter includes both non-UK Biobank and UK Biobank data). Genetic correlations across these 30 diseases are shown in Supplementary Fig. 24 and Supplementary Table 16. We again observed that most diseases have moderate genetic correlations within disease categories and between disease categories; we note that it is not feasible to estimate corresponding liability-scale phenotypic correlations in the absence of individual-level data.

**Fig. 6:**
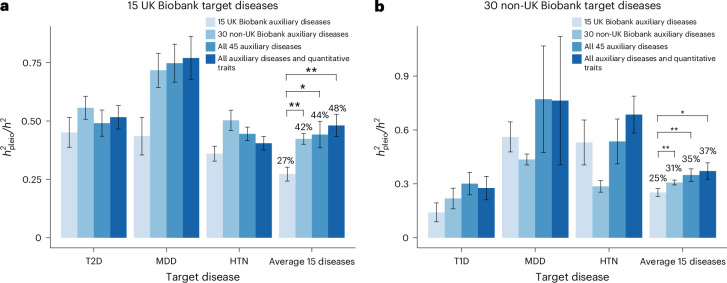
*h*^2^_pleio_/*h*^2^ estimates with respect to different sets of auxiliary diseases/traits. **a**, ${{h}^{2}}_{\mathrm{pleio}}/{h}^{2}$ estimates for three representative UK Biobank diseases and the average across 15 UK Biobank diseases with respect to four sets of auxiliary diseases/traits. **b**, ${{h}^{2}}_{\mathrm{pleio}}/{h}^{2}$ estimates for three representative non-UK Biobank diseases and the average across 30 non-UK Biobank diseases with respect to four sets of auxiliary diseases/traits. Data are presented as point estimate ± s.e. Error bars denote jackknife standard errors obtained via 200 genomic blocks. To assess statistical significance, we compare average estimates from 30 non-UK Biobank auxiliary diseases, all 45 auxiliary diseases and all 62 auxiliary diseases and quantitative traits to estimates from 15 UK Biobank auxiliary diseases as the control group using inverse-variance weighted Wald *z* test across diseases/traits: *two-sided *P* < 0.05; **two-sided *P* < 0.001. Numerical results are presented in Supplementary Tables 18, 22 and 23.

**Extended Data Fig. 5:**
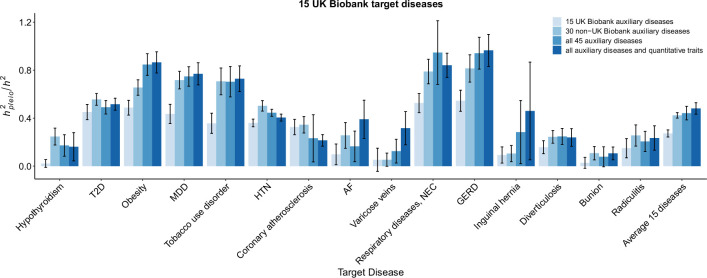
*h*^2^_*pleio*_/*h*^2^ of all 15 UK Biobank target diseases with respect to four types of auxiliary disease sets: 15 UK Biobank auxiliary diseases, 30 non-UK Biobank auxiliary diseases, all 45 auxiliary diseases and all 62 auxiliary diseases and quantitative traits. Data are presented as point estimate ± s.e. Error bars represent jackknife standard errors obtained via 200 genomic blocks. We compute the post-bias-correction standard error of the average estimate across 15 diseases using the average of ratio to scale the uncorrected jackknife s.e. of average: ($\frac{1}{15}{\sum }_{i=1}^{15}\frac{{post}-{correction\; s}.e{.}_{i}}{{pre}-{correction\; s}.e{.}_{i}}$) × uncorrected jackknife s.e. of average. Detailed results are provided in Supplementary Table 22. T2D, type 2 diabetes; MDD, depression; HTN, hypertension; GERD, gastroesophageal reflux disease; AF, atrial fibrillation.

We first estimated ${{h}^{2}}_{\mathrm{pleio}}/{h}^{2}$ for 15 UK Biobank target diseases with respect to different sets of auxiliary diseases. Results for three representative diseases (T2D, MDD and HTN), and the average across 15 diseases, are shown in Fig. 6a, Extended Data Fig. 5 and Supplementary Table 22. Average ${{h}^{2}}_{\mathrm{pleio}}/{h}^{2}$ increased to 42% with respect to 30 non-UK Biobank auxiliary diseases (jackknife s.e. (average) = 2%, s.d. = 27% across diseases), 44% with respect to all 45 UK Biobank and non-UK Biobank auxiliary diseases (jackknife s.e. (average) = 6%, s.d. = 31% across diseases) and 48% (jackknife s.e. (average) = 5%, s.d. = 28% across diseases) with respect to all 45 UK Biobank and non-UK Biobank auxiliary diseases and 17 UK Biobank quantitative traits, as compared with 27% with respect to 15 UK Biobank auxiliary diseases. Several diseases were dominated by ${{h}^{2}}_{\mathrm{pleio}}$, including MDD (77%, s.e. 9% with respect to all 62 auxiliary traits) and T2D (52%, s.e. 5% with respect to all 62 auxiliary traits).

**Extended Data Fig. 6:**
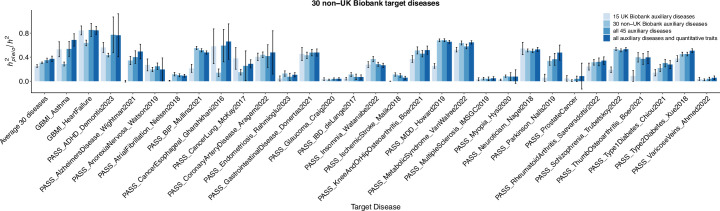
*h*^2^_*pleio*_/*h*^2^ of all 30 non-UK Biobank target diseases with respect to four types of auxiliary disease sets: 15 UK Biobank auxiliary diseases, 30 non-UK Biobank auxiliary diseases, all 45 auxiliary diseases and all 62 auxiliary diseases and quantitative traits. Data are presented as point estimate ± s.e. Error bars represent jackknife standard errors obtained via 200 genomic blocks. We compute the post-bias-correction standard error of the average estimate across 30 diseases using the average of ratio to scale the uncorrected jackknife s.e. of average: ($\frac{1}{30}{\sum }_{i=1}^{30}\frac{{post}-{correction\; s}.e{.}_{i}}{{pre}-{correction\; s}.e{.}_{i}}$) × uncorrected jackknife s.e. of average. Detailed results are provided in Supplementary Table 23.

We next estimated ${{h}^{2}}_{\mathrm{pleio}}/{h}^{2}$ for 30 non-UK Biobank target diseases with respect to different sets of auxiliary diseases. Results for three representative diseases (type 1 diabetes, attention deficit/hyperactivity disorder and asthma), and the average across 30 diseases, are shown in Fig. 6b, Extended Data Fig. 6 and Supplementary Table 23. We observed a similar trend (increasing and then showing diminishing increases as the number of auxiliary traits increases) but with lower overall estimates: average ${{h}^{2}}_{\mathrm{pleio}}/{h}^{2}$ increased to 31% with respect to 30 non-UK Biobank auxiliary diseases (jackknife s.e. (average) = 1%, s.d. = 21% across diseases), 35% with respect to all 45 UK Biobank and non-UK Biobank auxiliary diseases (jackknife s.e. (average) = 4%, s.d. = 23% across diseases) and 37% with respect to all 45 UK Biobank and non-UK Biobank auxiliary diseases and 17 UK Biobank auxiliary quantitative traits (jackknife s.e. (average) = 5%, s.d. of 25% across diseases), as compared with 25% with respect to 15 UK Biobank auxiliary diseases. We note that further increases in ${{h}^{2}}_{\mathrm{pleio}}/{h}^{2}$ are possible if additional auxiliary traits are incorporated.

Finally, we estimated ${{h}^{2}}_{\mathrm{pleio}}/{h}^{2}$ of 15 UK Biobank target diseases or 30 non-UK Biobank target diseases with respect to auxiliary diseases in each of the 11 PheCode disease categories^33^ spanned by all 45 UK Biobank and non-UK Biobank auxiliary diseases (analogous to Fig. 5). Results are shown in Supplementary Fig. 25 and Supplementary Table 24. We again determined that auxiliary diseases from a single disease category often explain the majority of ${{h}^{2}}_{\mathrm{pleio}}$ (for example, MDD had 68% (s.e. 8%) of SNP heritability shared with other mental disorders) and that removing a single disease category from the set of auxiliary diseases reduced ${{h}^{2}}_{\mathrm{pleio}}/{h}^{2}$ much less than the ${{h}^{2}}_{\mathrm{pleio}}/{h}^{2}$ shared with that disease category (for example, MDD ${{h}^{2}}_{\mathrm{pleio}}/{h}^{2}$ was reduced by only 23% (s.e. 6%) when removing other mental disorders).

### Comparing shared genetic and nongenetic variance

**Extended Data Fig. 7:**
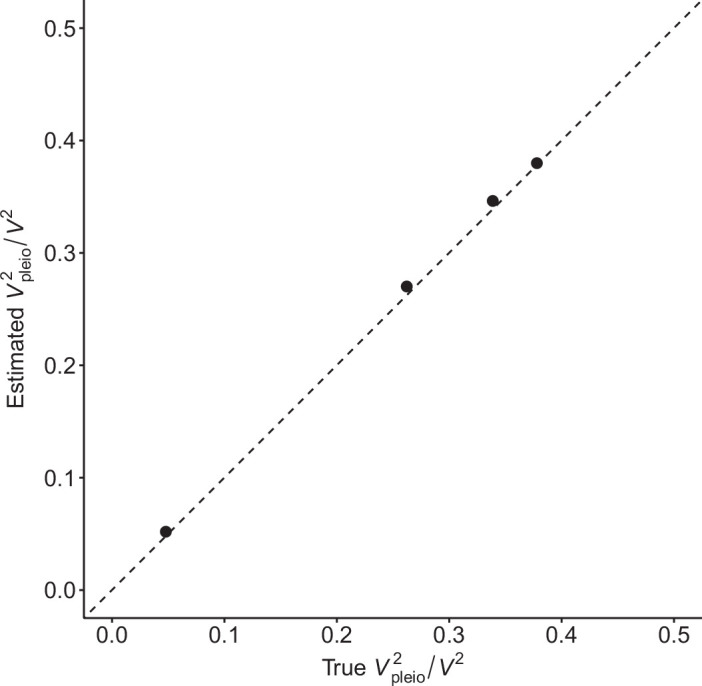
Simulations of *V*^2^_*pleio*_/*V*^2^ and its standard error. True ${r}_{o}$ is set to 0.5 within diseases categories and 0.1 between diseases categories for the 15 diseases (based on the seven Phecode disease categories), which implies four different values of true ${{V}^{2}}_{{pleio}}/{V}^{2}$ for each target disease (ranging from 0.05 to 0.38). We simulated liabilities for 228,258 individuals. We computed true ${r}_{l}$ and true ${{V}^{2}}_{{pleio}}$ based on the simulated liabilities. Then, we used liability threshold model to generate binary phenotypes based on the empirical prevalence for these 15 UK Biobank diseases. We applied our method on the simulated binary phenotypes to estimate ${{V}^{2}}_{{pleio}}$ and compared it to the true value. Each point and error bar represents the mean and standard error of diseases that has the same true ${{V}^{2}}_{{pleio}}/{V}^{2}$ across 100 simulations. Estimated ${{V}^{2}}_{{pleio}}/{V}^{2}$ is unbiased without the need for bias correction, in which error bars are smaller than point size in some cases. Detailed results are provided in Supplementary Table 25.

We sought to compare ${{h}^{2}}_{\mathrm{pleio}}/{h}^{2}$ to the proportion of liability-scale total phenotypic variance of a target disease that is shared with a given set of auxiliary diseases (pleiotropic phenotypic variance, ${{V}^{2}}_{\mathrm{pleio}}$; defined in Methods, analogous to equation (1); Fig. 1b). We extended our method to estimate ${{V}^{2}}_{\mathrm{pleio}}$ by replacing genetic correlations (${r}_{g}$) in equation (3) with liability-scale phenotypic correlations (${r}_{l}$), estimated from individual-level data as in ref. ^31^ (Methods). We determined via simulations that ${{V}^{2}}_{\mathrm{pleio}}$ estimates are unbiased without the need for bias correction, as ${r}_{l}$ is accurately estimated at large sample sizes (Extended Data Fig. 7, Supplementary Fig. 26 and Supplementary Table 25). We used the ratio of pleiotropic phenotypic variance versus total liability-scale phenotypic variance (${{V}^{2}}_{\mathrm{pleio}}/{V}^{2}$) to quantify the proportion of total liability-scale phenotypic variance that is pleiotropic (noting that ${V}^{2}=1$ in standard parametrizations). As estimating ${r}_{l}$ requires individual-level data, we analyzed 15 UK Biobank diseases only (Table 1).

**Fig. 7:**
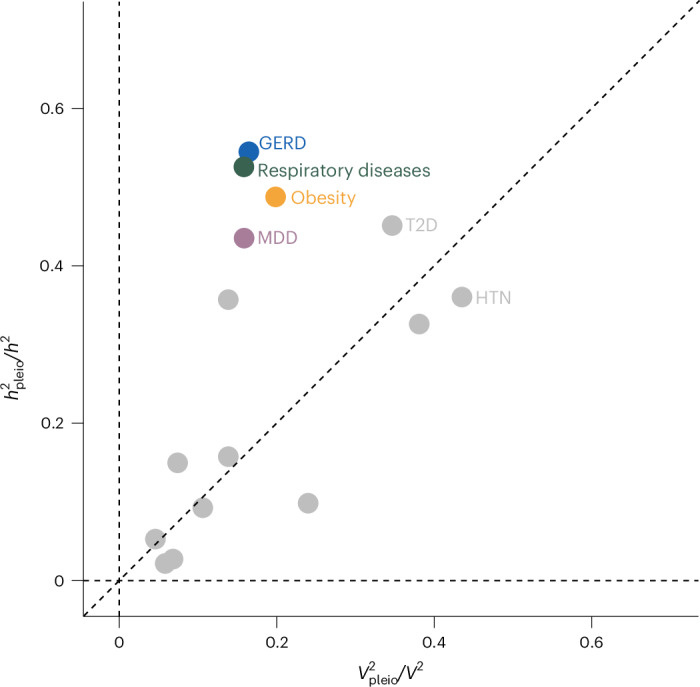
Comparison of *h*^2^_pleio_/*h*^2^ versus *V*^2^_pleio_/*V*^2^ for 15 diseases in the UK Biobank. We performed a two-sided Wald *z* test to compare ${{h}^{2}}_{\mathrm{pleio}}/{h}^{2}$ and ${{V}^{2}}_{\mathrm{pleio}}/{V}^{2}$ estimates. Colored dots denote diseases for which ${{h}^{2}}_{\mathrm{pleio}}/{h}^{2}$ estimates are significantly different from ${{V}^{2}}_{\mathrm{pleio}}/{V}^{2}$ estimates (Bonferroni-corrected two-sided *P* < 0.05/15 for multiple comparisons). Numerical results are presented in Supplementary Table 26.

A comparison of estimates of ${{h}^{2}}_{\mathrm{pleio}}/{h}^{2}$ versus ${{V}^{2}}_{\mathrm{pleio}}/{V}^{2}$ is shown in Fig. 7, Supplementary Fig. 27 and Supplementary Table 26; to ensure a fair comparison, we pruned the same auxiliary diseases in estimates of ${{V}^{2}}_{\mathrm{pleio}}/{V}^{2}$ as in prior estimates of ${{h}^{2}}_{\mathrm{pleio}}/{h}^{2}$. We determined that estimates of ${{h}^{2}}_{\mathrm{pleio}}/{h}^{2}$ were generally larger than estimates of ${{V}^{2}}_{\mathrm{pleio}}/{V}^{2}$ (ratio of averages = 1.51 × (s.e. 0.16)). ${{h}^{2}}_{\mathrm{pleio}}/{h}^{2}$ was significantly larger than ${{V}^{2}}_{\mathrm{pleio}}/{V}^{2}$ (*P* < 0.05/15) for 4 of 15 diseases, including gastroesophageal reflux disease (GERD) (0.54 (s.e. 0.09) versus 0.16 (s.e. 0.007)), respiratory diseases (0.53 (s.e. 0.08) versus 0.16 (s.e. 0.007)), obesity (0.49 (s.e. 0.06) versus 0.20 (s.e. 0.006)) and MDD (0.44 (s.e. 0.08) versus 0.16 (s.e. 0.007)). Owing to relatively small values of SNP heritability, estimates of the proportion of nongenetic variance that is shared with auxiliary diseases (${{E}^{2}}_{\mathrm{pleio}}/{E}^{2}$) were only slightly smaller than ${{V}^{2}}_{\mathrm{pleio}}/{V}^{2}$ estimates on average (Supplementary Fig. 28 and Supplementary Table 26). We note that the difference between ${{h}^{2}}_{\mathrm{pleio}}$ and ${{V}^{2}}_{\mathrm{pleio}}$ also includes heritability not captured by common SNPs, such as rare variant heritability, although we expect that rare variant heritability is generally relatively small^40–42^. A secondary analysis is described in Supplementary Notes and Supplementary Fig. 31.

## Discussion

We have defined and estimated ${{h}^{2}}_{\mathrm{pleio}}$, the genetic variance of a target disease that is shared with a specific set of auxiliary diseases. We highlight four findings. First, bias correction had a large impact on estimates of ${{h}^{2}}_{\mathrm{pleio}}$. Second, roughly half of the total SNP heritability of common diseases is shared with other disease/traits. Third, ${{h}^{2}}_{\mathrm{pleio}}/{h}^{2}$ is broadly distributed across disease categories, with ${{h}^{2}}_{\mathrm{pleio}}$ decreasing only slightly when removing one auxiliary disease category. Finally, we compared ${{h}^{2}}_{\mathrm{pleio}}/{h}^{2}$ to ${{V}^{2}}_{\mathrm{pleio}}/{V}^{2}$ and determined that genetic variance is more strongly shared across diseases than environmental variance; this finding could be explained by pleiotropic stabilizing selection on complex traits, in which common variants with effects on multiple traits are preferentially retained^43^.

We emphasize four downstream implications. First, our results can inform the incorporation of genome-wide priors in polygenic risk scores that leverage pleiotropic diseases/traits^26,44–48^. Second, our work motivates the definition and estimation of partitioned pleiotropic shared heritability, in which genetic values of auxiliary diseases/traits are partitioned into tissue/cell type-specific components^49–51^, increasing shared genetic variance with the target disease and elucidating shared biological processes^1–6,15,22,23^. Third, genetic values of auxiliary traits could also be partitioned into gene pathways, elucidating shared biological pathways^52–54^. As such, our method for estimating ${{h}^{2}}_{\mathrm{pleio}}$ may be developed in further ways to prioritize drug targets for multimorbid diseases. Finally, ${{h}^{2}}_{\mathrm{pleio}}$ could be applied to high-dimensional auxiliary traits^55^, such as brain imaging traits in studies of psychiatric or neurodegenerative diseases.

We note several limitations of our work. First, jackknife standard errors on estimates of ${{h}^{2}}_{\mathrm{pleio}}$ do not account for stochasticity introduced by pruning genetically correlated auxiliary diseases to ensure numerical stability; however, we anticipate minimal variation in the set of pruned diseases across genomic jackknife blocks, owing to limited variation in ${r}_{g}$ across blocks. Second, ${{h}^{2}}_{\mathrm{pleio}}$ depends on the set of selected auxiliary traits; however, the magnitude of the increase in ${{h}^{2}}_{\mathrm{pleio}}/{h}^{2}$ shows diminishing increases as the number of auxiliary traits increases (Fig. 6), implying that expanding the set of auxiliary traits is unlikely to substantially increase ${{h}^{2}}_{\mathrm{pleio}}/{h}^{2}$ to the high level of T2D and MDD for most other diseases, although further modest increases are possible. Finally, we restricted analyses to European individuals in the UK Biobank, but it is important to analyze more diverse cohorts^56,57^, particularly for diseases whose prevalence varies across populations. Additional limitations are described in Supplementary Notes. Despite these limitations, our definition and estimation of ${{h}^{2}}_{\mathrm{pleio}}$ provide a robust quantification of the genetic variance of a target disease that is shared with pleiotropic diseases/traits.

## Methods

### Ethics statement

The UK Biobank has received ethical approval from the National Information Governance Board for Health and Social Care and the National Health Service North West Centre for Research Ethics Committee (ref. no. 11/NW/0382).

### Definition of *h*^2^_pleio_

We assume that the total liability of the target disease consists of genetic value (*G*) and environmental value (*E*). *G* of the target disease is divided into two components: private (disease-specific) genetic value (${G}_{\mathrm{private}}$) and pleiotropic genetic value (${G}_{\mathrm{pleio}}$), with the pleiotropic genetic value defined as a weighted linear combination of auxiliary disease genetic values (Fig. 1a),

4 $${G}_{T}={G}_{\mathrm{private}}+\mathop{\sum }\limits_{j=1}^{n}{\alpha }_{j}G_{j},$$

where ${\alpha }_{j}$ is the weight of the *j*th auxiliary disease $(D_{j})$, $G_{j}$ is the genetic value of $D_{j}$, and ${G}_{{T}}$ is the genetic value of target disease.

We define pleiotropic shared heritability as the maximum proportion of the total target disease heritability explained by the auxiliary diseases in the entire population. We use the ratio of pleiotropic shared heritability versus total heritability ($\frac{{{h}^{2}}_{\mathrm{pleio}}}{{h}^{2}}$) to quantify the proportion of genetic variance that is pleiotropic,

5 $$\frac{{h}_{\mathrm{pleio}}^{2}}{{h}^{2}}=\mathop{\max }\limits_{{\alpha }_{j}}{r}_{\mathrm{ind}}^{2}\left[\mathop{\sum }\limits_{j=1}^{n}{\alpha }_{j}{G}_{j},{G}_{T}\right],$$

where ${r}_{\mathrm{ind}}$ denotes the correlation across individuals. We emphasize that ${{h}^{2}}_{\mathrm{pleio}}$ is a function of both the target disease and the selected set of auxiliary diseases/traits.

Analogously, we assume that standardized causal SNP effect sizes (number of s.d. increase in phenotype per 1 s.d. increase in genotype) on the target disease consist of two components: disease-specific effect sizes (${\beta }_{\mathrm{disease-specific}}$) and pleiotropic effect sizes (${\beta }_{\mathrm{pleio}}$), with the pleiotropic effect sizes defined as a weighted linear combination of auxiliary disease effect sizes,

6 $${\beta }_{T}={\beta }_{\mathrm{disease-specific}}+\displaystyle \mathop{\sum }\limits_{j=1}^{n}{\alpha }_{j}{\beta }_{j},$$

where ${\alpha }_{j}$ is the weight for the *j*th auxiliary disease $(D_{j})$, *β* is the vector of additive causal SNP effect sizes ($\beta =({\beta }_{1},{\beta }_{2},\ldots ,{\beta }_{m})$, where $m$ denotes the number of SNPs), ${\beta }_{{T}}$ is the vector of SNP effects on the target disease and ${\beta }_{j}$ is the vector of SNP effects on the *j*th auxiliary disease. In the general case that the genotype data do not include all causal SNPs, $\beta$ are best-fit effect sizes obtained by projecting the phenotype onto the genotype space in the infinite population.

We restate the definition of equation (2) as

7 $${h}_{\mathrm{pleio}}^{2}/{h}^{2}=\mathop{\max }\limits_{{\alpha }_{j}}{r}_{\mathrm{SNP}}^{2}\left[\mathop{\sum }\limits_{j=1}^{n}{\alpha }_{j}{\beta }_{j},{\beta }_{T}\right],$$

where ${r}_{\mathrm{SNP}}$ denotes the correlation across SNPs. We note that equation (7) applies equally to causal SNP effect sizes on either the liability or observed scale, as these effect sizes differ only by a constant scaling factor, and the squared correlation ${r}^{2}$ is invariant under linear rescaling.

We note the definition in equations (1) and (2) are equivalent under the assumptions that

8 $$\begin{array}{rcl}E\left[{\beta }_{T,i}{\beta }_{{j,i}^{{\prime} }}\times \mathrm{LD}\left(i,{i}^{{\prime} }\right)\right]=0 & \mathrm{for} & {i}^{{\prime} }\ne i,\end{array}$$

where $\beta$ denotes the causal SNP effect sizes defined under a random effect model, *j* is the disease index (including the target disease), $i,{i}^{{\prime} }$ are SNP indices and LD is the correlation between genotypes. Intuitively, the assumption requires the causal effect sizes of SNPs that are in LD with each other to be uncorrelated. We note that causal effect sizes for different SNPs have been shown to have minimal correlations, particularly among common SNPs^58^.

In detail, we model genetic value using standardized genotypes *X* and causal SNP effect vector $\beta$_._ The genetic value for the target disease is ${G}_{{T}}=X{\beta }_{{T}}$, and the weighted linear combination of genetic value for $n$ auxiliary disease is ${\sum }_{j=1}^{n}{\alpha }_{j}G_{j}=X({\sum }_{j=1}^{n}{\alpha }_{j}{\beta }_{j})$. Using the property of the trace operator,

9 $${\left(X{\beta }_{T}\right)}^{T}X{\beta }_{T}=\mathrm{Tr}\left({X}^{T}X{\beta }_{T}{\beta }_{T}^{T}\right)=\mathop{\sum }\limits_{i\in \mathrm{SNPs}}\mathop{\sum }\limits_{i{\prime} \in \mathrm{SNPs}}{\left\{{X}^{T}X\right\}}_{i,i{\prime} }{\beta }_{T,i}{\beta }_{T{,}i{{\prime} }},$$

and

10 $$\begin{array}{l}{\left(X{\beta }_{T}\right)}^{T}X\left(\mathop{\sum }\limits_{j=1}^{n}{\alpha }_{j}{\beta }_{j}\right)=\mathrm{Tr}\left({X}^{T}X\left(\mathop{\sum }\limits_{j=1}^{n}{\alpha }_{j}{\beta }_{T}{\beta }_{j}^{T}\right)\right)\\ =\mathop{\sum }\limits_{i\in \mathrm{SNPs}}\mathop{\sum }\limits_{i{\prime} \in \mathrm{SNPs}}{\left\{{X}^{T}X\right\}}_{i,i{\prime} }\left(\mathop{\sum }\limits_{j=1}^{n}{\alpha }_{j}{\beta }_{T,i}{\beta }_{j,i{\prime} }\right).\end{array}$$

Using the assumption of equation (8), ${\sum }_{i\ne i{\prime} }{\left\{{X}^{T}X\right\}}_{i,i{\prime} }{\beta }_{T,i}{\beta }_{T,i{\prime} }=0$ and ${\sum }_{i\ne i{\prime} }{\left\{{X}^{T}X\right\}}_{i,i{\prime} }({\sum }_{j=1}^{n}{\alpha }_{j}{\beta }_{T,i}{\beta }_{j,i{\prime} })=0$ when the number of SNP goes to infinity, yielding

11 $${(X{\beta }_{T})}^{T}X{\beta }_{T}=\mathop{\sum }\limits_{i\in {\mathrm{SNPs}}}{\beta }_{T,i}^{2}={\beta }_{T}^{T}{\beta }_{T},$$

and

12 $${\left(X{\beta }_{T}\right)}^{T}X\left(\mathop{\sum }\limits_{j=1}^{n}{\alpha }_{j}{\beta }_{j}\right)=\mathop{\sum }\limits_{i\in \mathrm{SNPs}}\left(\mathop{\sum }\limits_{j=1}^{n}{\alpha }_{j}{\beta }_{T,i}{\beta }_{j,i}\right)={\beta }_{T}^{T}\left(\mathop{\sum }\limits_{\,j=1}^{n}{\alpha }_{j}{\beta }_{j}\right).$$

Therefore, the covariance between the target genetic value and weighted auxiliary disease genetic value is

13 $$\mathrm{Cov}\left(\mathop{\sum }\limits_{\,j=1}^{n}{\alpha }_{j}G_{j},{G}_{T}\right)=\mathop{\sum }\limits_{j=1}^{n}{\alpha }_{j}{\beta }_{j}^{T}{\beta }_{T},$$

and the variances are

14 $$\mathrm{Var}\left(\mathop{\sum }\limits_{\,j=1}^{n}{\alpha }_{j}G_{j}\right)={\sum }_{j,k}{\alpha }_{j}{\alpha }_{k}{\beta }_{j}^{T}{\beta }_{k},$$

and

15 $$\mathrm{Var}\left({G}_{{T}}\right)={\beta }_{T}^{T}{\beta }_{{T}}.$$

Therefore, the squared correlation of genetic value across individuals (${r}_{\mathrm{ind}}^{2}$) in a population of infinite sample size equals the squared correlation across SNPs (${r}_{\mathrm{SNP}}^{2}$):

16 $${r}_{\mathrm{ind}}^{2}\left(\mathop{\sum }\limits_{j=1}^{n}{\alpha }_{j}G_{j},{G}_{T}\right)={r}_{\mathrm{SNP}}^{2}\left(\mathop{\sum }\limits_{j=1}^{n}{\alpha }_{j}{\beta }_{j},{\beta }_{T}\right).$$

### Initial estimate of *h*^2^_pleio_

We use PHBC to obtain an unbiased estimate of $\frac{{h}_{\mathrm{pleio}}^{2}}{{h}^{2}}$ with finite sample sizes.

According to the definition in equation (7), pleiotropic shared heritability is not changed when multiplying **α** by a non-zero scaling factor. We note that the quantity in equation (7) is equivalent to regression ${{\bf{R}}}^{{\bf{2}}}$; therefore, we change the optimization to

17 $$\frac{{h}_{\mathrm{pleio}}^{2}}{{h}^{2}}=\mathop{\max }\limits_{{\alpha }_{j}}\left[{R}_{{\mathrm{regression}}\,{\mathrm{across}}\,{\mathrm{SNPs}}}^{2}\left({\beta }_{T} ; \mathop{\sum }\limits_{j=1}^{n}{\alpha }_{j}{\beta }_{j}\right)\right],$$

so that the ${\alpha }_{j}$ are identifiable. We derive the expression of ${h}_{\mathrm{pleio}}^{2}$ as follows:

- Let ${{\boldsymbol{\alpha }}}^{* }$ be the estimated ${\boldsymbol{\alpha }}$ that maximizes the right-hand side of equation (17)
- Let ${{\bf{C}}}_{\mathrm{DD}}$ be the $n\times n$ covariance matrix of ${\{{\beta }_{j}\}}_{j=1}^{n}$
- Let ${{\bf{c}}}_{\mathrm{TD}}$ be the $n\times 1$ covariance vector between ${\beta }_{{T}}$ and ${\left\{{\beta }_{j}\right\}}_{j=1}^{n}$

Under the assumption of equation (8), the variance of pleiotropic genetic value $X{\sum }_{j=1}^{n}{\alpha }_{j}{\beta }_{j}$ is

18 $$\mathrm{Var}\left[X\mathop{\sum }\limits_{j=1}^{n}{\alpha }_{j}{\beta }_{j}\right]=m\times \mathop{\sum }\limits_{i=1}^{n}\mathop{\sum }\limits_{j=1}^{n}{\alpha }_{i}{\alpha }_{j}\mathrm{Cov}({\beta }_{i},{\beta }_{j})={m\times {\boldsymbol{\alpha }}}^{T}{{\bf{C}}}_{\mathrm{DD}}{\boldsymbol{\alpha }}.$$

To estimate ${{h}^{2}}_{\mathrm{pleio}}$, we first estimate the ${{\boldsymbol{\alpha }}}^{* }$ by minimizing the squared error between ${\beta }_{T}$ and ${\sum }_{j=1}^{n}{\alpha }_{j}{\beta }_{j}$: ${({\beta }_{T}-{\sum }_{j=1}^{n}{\alpha }_{j}{\beta }_{j})}^{T}({\beta }_{T}-{\sum }_{j=1}^{n}{\alpha }_{j}{\beta }_{j})$ (which is equivalent to maximizing regression ${{\bf{R}}}^{{\bf{2}}}$). The parameters are estimated across all SNPs, which is sufficiently large that prevents overfitting. By taking the partial derivative of ${({\beta }_{T}-{\sum }_{j=1}^{n}{\alpha }_{j}{\beta }_{j})}^{T}({\beta }_{T}-{\sum }_{j=1}^{n}{\alpha }_{j}{\beta }_{j})$ with respect to ${\boldsymbol{\alpha }}$,

19 $$\begin{array}{l}\frac{\partial }{\partial {\boldsymbol{\alpha }}}{\left({\beta }_{T}-\mathop{\sum }\limits_{j=1}^{n}{\alpha }_{j}{\beta }_{j}\right)}^{T}\left({\beta }_{T}-\mathop{\sum }\limits_{j=1}^{n}{\alpha }_{j}{\beta }_{j}\right)\\ \begin{array}{l}=\frac{\partial }{\partial {\boldsymbol{\alpha }}}\left({\beta }_{T}^{T}{\beta }_{T}-2\mathop{\sum }\limits_{j=1}^{n}{{\alpha }_{j}\beta }_{T}^{T}{\beta }_{j}+{\left(\mathop{\sum }\limits_{j=1}^{n}{\alpha }_{j}{\beta }_{j}\right)}^{T}\left(\mathop{\sum }\limits_{j=1}^{n}{\alpha }_{j}{\beta }_{j}\right)\right)\\ =m\times \frac{\partial }{\partial {\boldsymbol{\alpha }}}\left({h}_{T}^{2}/m-2{{\boldsymbol{\alpha }}}^{T}{{\bf{c}}}_{\mathrm{TD}}+{{\boldsymbol{\alpha }}}^{T}{{\bf{C}}}_{\mathrm{DD}}{\boldsymbol{\alpha }}\right)\\ =m\left(-2{{\bf{c}}}_{\mathrm{TD}}+\left({{\bf{C}}}_{\mathrm{DD}}{\boldsymbol{+}}{{\bf{C}}}_{\mathrm{DD}}^{{\boldsymbol{T}}}\right){\boldsymbol{\alpha }}\right),\end{array}\end{array}$$

where *m* is the number of SNPs; the expectation of each SNP effect is 0. By setting the partial derivative to zero, we can obtain the optimal weight vector for each SNP:

20 $${{\bf{C}}}_{\mathrm{DD}}{{\boldsymbol{\alpha }}}^{* }={{\bf{c}}}_{\mathrm{TD}}.$$

If ${{\bf{C}}}_{\mathrm{DD}}$ is invertible, the optimal ${{\boldsymbol{\alpha }}}^{* }$ is

21 $${{\boldsymbol{\alpha }}}^{* }={{\bf{C}}}_{\mathrm{DD}}^{-1}{{\bf{c}}}_{\mathrm{TD}}.$$

After substituting the ${{\boldsymbol{\alpha }}}^{* }$ into equation (18),

22 $${{h}^{2}}_{\mathrm{pleio}}=m\times {{\bf{c}}}_{\mathrm{TD}}^{T}{{\bf{C}}}_{\mathrm{DD}}^{-1}{{\bf{c}}}_{\mathrm{TD}}.$$

Elements in ${{\bf{c}}}_{\mathrm{TD}}$ and ${{\bf{C}}}_{\mathrm{DD}}$ denote per-SNP heritability and genetic covariance; in this study, we focus on SNP heritability and genetic correlation between causal SNP effect sizes. Let ${h}_{T}^{2}$ be the heritability of the target disease. ${{\bf{h}}}_{{\bf{D}}}^{{\bf{2}}}={\left\{{h}_{j}^{2}\right\}}_{j=1}^{n}$ is the vector consisting of the heritability of all auxiliary diseases. ${r}_{g}\left[{\bf{D}},T\right]$ is the genetic correlation vector between the target disease and all auxiliary diseases, ${r}_{g}{\left[{\bf{D}},{\bf{D}}\right]}^{-1}$ is the inverse of genetic correlation matrix of all auxiliary diseases. diag(.) is the diagonal matrix with all nondiagonal elements equal to zero.

23 $${{\bf{c}}}_{\mathrm{TD}}=\mathrm{diag}\left(\sqrt{\frac{{h}_{T}^{2}}{m}}{\left(\sqrt{\frac{{{\bf{h}}}_{{\bf{D}}}^{{\bf{2}}}}{{\bf{m}}}}\right)}^{T}\right){r}_{g}\left[{\bf{D}},T\,\right],$$

24 $${{\bf{C}}}_{{\bf{DD}}}^{-{\bf{1}}}=\mathrm{diag}\left(1/\sqrt{\frac{{{\bf{h}}}_{{\bf{D}}}^{{\bf{2}}}}{{\bf{m}}}}\right){r}_{g}{\left[{\bf{D}},{\bf{D}}\right]}^{-1}\mathrm{diag}\left(1/\sqrt{\frac{{{\bf{h}}}_{{\bf{D}}}^{{\bf{2}}}}{{\bf{m}}}}\right).$$

After substituting equations (23) and (24) into equation (22), ${{h}^{2}}_{\mathrm{pleio}}$ can be expressed as

25 $${{h}^{2}}_{\mathrm{pleio}}={h}_{T}^{2}\times {r}_{g}{\left[{\bf{D}},T\,\right]}^{T}{r}_{g}{\left[{\bf{D}},{\bf{D}}\right]}^{-1}{r}_{g}[{\bf{D}},T\,],$$

26 $$\frac{{{h}^{2}}_{\mathrm{pleio}}}{{h}^{2}}={r}_{g}{\left[{\bf{D}},T\,\right]}^{T}{r}_{g}{\left[{\bf{D}},{\bf{D}}\right]}^{-1}{r}_{g}[{\bf{D}},T\,].$$

Equation (26) provides the initial pleiotropic shared heritability estimates, before bias correction described in the next section. In this study, we used GWAS summary statistics as input and reimplemented cross-trait LDSC^8^ so that the standard errors of all quantities on the right-hand side of equation (26) are estimated through the same genomic jackknife blocks. We used 1000 Genomes Project^59^ Europeans as a reference LD panel to estimate genetic correlation. Standard error estimates were obtained via genomic block-jackknife with 200 blocks applied across all traits, which were created by partitioning 1,217,311 HapMap3 SNPs^36^. We primarily focus on ${{h}^{2}}_{\mathrm{pleio}}/{h}^{2}$ estimates (instead of ${{h}^{2}}_{\mathrm{pleio}}$ estimates) throughout the manuscript.

### Monte Carlo bias correction to initial estimate of *h*^2^_pleio_

The initial estimates ${(\frac{{h}_{\mathrm{pleio}}^{2}}{{h}^{2}})}_{\mathrm{initial}}$ are based on estimates of genetic correlation in finite GWAS sample size, which introduces an upward bias in estimating $\frac{{{h}^{2}}_{\mathrm{pleio}}}{{h}^{2}}$ owing to inverting the estimated genetic correlation matrix and sampling correlation between ${r}_{g}{\left[{\bf{D}},T\,\right]}^{T},{r}_{g}{\left[{\bf{D}},{\bf{D}}\right]}^{-1}$, and ${r}_{g}\left[{\bf{D}},T\,\right]$ in equation (26),

27 $${\left(\frac{{h}_{\mathrm{pleio}}^{2}}{{h}^{2}}\right)}_{\mathrm{initial}} > {\left(\frac{{h}_{\mathrm{pleio}}^{2}}{{h}^{2}}\right)}_{\mathrm{true}}.$$

Specifically, if only one auxiliary disease is included, $${{h}^{2}}_{\mathrm{pleio}}/{h}^{2}$$ will be equivalent to $${r}_{g}^{2}$$; the expectation of the squared genetic correlation estimates $$E[\widehat{{r}_{g}^{2}}]={r}_{g}^{2}+({\mathrm{s}}.{\mathrm{e}}.(\widehat{{r}_{g}}))^{2}$$ will be larger than the square of true genetic correlation $${r}_{g}^{2}$$, indicating an upward bias in $${{h}^{2}}_{\mathrm{pleio}}/{h}^{2}$$. If multiple auxiliary diseases are included, the expectation of equation (3) is upward biased for noisy $$\widehat{{r}_{{\rm{g}}}[{\bf{D}},{\bf{T}}]}$$ when $${r}_{g}{\left[{\bf{D}},{\bf{D}}\right]}^{-1}$$ is positive definite: $$E[\widehat{{r}_{g}{\left[{\bf{D}},{\bf{T}}\right]}^{T}}r_{g}{\left[{\bf{D}},{\bf{D}}\right]}^{-1}\widehat{{r}_{g}[{\bf{D}},{\bf{T}}]}]={{r}_{g}{\left[{\bf{D}},{\bf{T}}\right]}^{T}r}_{g}{\left[{\bf{D}},{\bf{D}}\right]}^{-1}{r}_{g}\left[{\bf{D}},{\bf{T}}\right]$$$$+E[\epsilon ({r}_{g}{\left[{\bf{D}},{\bf{T}}\right])}^{T}$$$${r}_{g}{\left[{\bf{D}},{\bf{D}}\right]}^{-1}\epsilon ({r}_{g}[{\bf{D}},{\bf{T}}])]$$, where $$\epsilon ({r}_{g}[{\bf{D}},{\bf{T}}])$$ is the estimation error of $$\widehat{{r}_{g}[{\bf{D}},{\bf{T}}]}$$; we used $$E\left[\widehat{{r}_{g}[{\bf{D}},{\bf{T}}]}\right]={r}_{g}[{\bf{D}},{\bf{T}}]$$. As $${r}_{g}{\left[{\bf{D}},{\bf{D}}\right]}^{-1}$$ is the estimate of the inverse of a genetic correlation matrix which is positive semi-definite, it is probably positive semi-definite. Therefore, the second term in this equation is non-negative, probably causing an upward bias in our initial estimate of $${{h}^{2}}_{\mathrm{pleio}}/{h}^{2}$$ in equation (3).

To obtain an unbiased estimate, we use a Monte Carlo bias correction procedure to estimate the proportion of bias in the point estimate of $\frac{{{h}^{2}}_{\mathrm{pleio}}}{{h}^{2}}$ and then correct the bias. First, we generate Monte Carlo samples of the estimation error ${\epsilon }_{i}$ in the genetic correlation matrix from the sampling covariance matrix^17^ generated from genomic block-jackknife; if the dimension of the genetic correlation matrix is *n* × *n*, the sampling covariance matrix is a symmetric matrix with dimension *n*(*n* − 1)/2 × *n*(*n* − 1)/2, which captures the joint distribution across estimation errors of different elements in ${r}_{g}[{\bf{D}},T]$ and ${r}_{g}[{\bf{D}},{\bf{D}}]$. Second, we add the estimation noise to the point estimate of genetic correlation matrix to create Monte Carlo samples of estimated ${r}_{g}[{\bf{D}},T]$ and ${r}_{g}{\left[{\bf{D}},{\bf{D}}\right]}^{-1}$. We filter these Monte Carlo samples to only keep those whose ${r}_{g}[{\bf{D}},{\bf{D}}]$ are nonsingular (that is the smallest eigenvalue of ${r}_{g}[{\bf{D}},{\bf{D}}]$ is larger than 0). We repeat this Monte Carlo procedure until we have generated 200 samples of genetic correlation estimates.

Using these Monte Carlo samples, we next estimate a scaling coefficient ${\xi }_{c}$ so that

28 $$\begin{array}{l}{{{\mathrm{Avg}}_{i}[({\xi }_{c}r}_{g}[{\bf{D}},T]+{\epsilon }_{i}[{\bf{D}},T])}^{T}({r}_{g}{[{\bf{D}},{\bf{D}}]+{\epsilon }_{i}[{\bf{D}},{\bf{D}}])}^{-1}{({\xi }_{c}r}_{g}[{\bf{D}},T\,]\\ +{\epsilon }_{i}[{\bf{D}},T\,])]={\left(\frac{{h}_{\mathrm{pleio}}^{2}}{{h}^{2}}\right)}_{\mathrm{initial}}.\end{array}$$

The left-hand side is the average across Monte Carlo samples; ${\epsilon }_{i}$ are the respective Monte Carlo samples of estimation errors for ${r}_{g}[{\bf{D}},T]$ and ${r}_{g}[{\bf{D}},{\bf{D}}]$, where *i* indexes Monte Carlo samples. The above equation is based on the heuristic that the magnitude of pleiotropic shared heritability is proportional to the average genetic correlation between target disease and auxiliary diseases ${r}_{g}[{\bf{D}},T]$. Downward scaling ${r}_{g}[{\bf{D}},T]$ will downward scale $\frac{{{h}^{2}}_{\mathrm{pleio}}}{{h}^{2}}$, which controls the upward bias in equation (27). However, ${r}_{g}[{\bf{D}},{\bf{D}}]$ is between pairs of auxiliary diseases, which is not directly connected to the scale of $\frac{{{h}^{2}}_{\mathrm{pleio}}}{{h}^{2}}$. The error term ${\epsilon }_{i}$ is not multiplied by ${\xi }_{c}$, as the estimation error for genetic correlation is a function of sample size and the heritability, which is not proportional to the scale of the point estimate of genetic correlation.

We estimate the scaling coefficient ${\xi }_{c}$ using binary search on the interval [0,1], in order to satisfy equation (28). We multiply the initial estimate ${(\frac{{h}_{\mathrm{pleio}}^{2}}{{h}^{2}})}_{\mathrm{initial}}$ by ${\xi }_{c}^{2}$ to obtain the bias-corrected estimate of ${{h}^{2}}_{\mathrm{pleio}}/{h}^{2}$. To obtain the bias-corrected standard error, we scale the uncorrected jackknife s.e. by the ratio of the s.d. of corrected estimates across Monte Carlo samples divided by the s.d. of uncorrected estimates across Monte Carlo samples,

29 $$\begin{array}{l}\displaystyle \frac{{\mathrm{s.d.}}_{i}\left(\left[\left({\xi }_{c}r_{g}\left[{\bf{D}},T\right]+{\epsilon }_{i}\left[{\bf{D}},T\right]\right)^{T}\left({r}_{g}\left[{\bf{D}},{\bf{D}}\right]+{\epsilon }_{i}\left[{\bf{D}},{\bf{D}}\right]\right)^{-1}\left({\xi }_{c}r_{g}\left[{\bf{D}},T\right]+{\epsilon }_{i}\left[{\bf{D}},T\right]\right)\right]\right)}{{\mathrm{s.d.}}_{i}\left(\left[\left(r_{g}\left[{\bf{D}},T\right]+{\epsilon }_{i}\left[{\bf{D}},T\right]\right)^{T}\left({r}_{g}\left[{\bf{D}},{\bf{D}}\right]+{\epsilon }_{i}\left[{\bf{D}},{\bf{D}}\right]\right)^{-1}{(r}_{g}\left[{\bf{D}},T\right]+{\epsilon }_{i}\left[{\bf{D}},T\right])\right]\right)}\\ \displaystyle \times {\rm{uncorrected}}\,{\rm{jackknife}}\,{\rm{s.e.}}\end{array}$$

The numerator in the ratio is the s.d. across post-bias-correction Monte Carlo samples and the denominator of the ratio is the s.d. across pre-bias-correction Monte Carlo samples, where *i* indexes Monte Carlo samples. The ratio of the two s.d. quantifies the change in s.e. attributed to Monte Carlo bias correction. We also compute the post-bias-correction standard error of the average estimate across *N* diseases using the average of this ratio to scale the uncorrected jackknife s.e. of average

30 $$\left(\frac{1}{N}\mathop{\sum }\limits_{i=1}^{N}\frac{{\mathrm{post-correction}}\,{\mathrm{s.e.}}_{i}}{{\mathrm{pre-correction}}\,{\mathrm{s.e.}}_{i}}\right)\times {\mathrm{uncorrected}}\,{\mathrm{jackknife}}\,{\mathrm{s.e.}}\,{\mathrm{of}}\,{\mathrm{average}}.$$

### Pruning auxiliary diseases with high genetic correlation

In the presence of collinearity among auxiliary diseases and large uncertainty in genetic correlation estimates, the estimator in equation (26) can be numerically unstable, which we define as (1) the pre-correction jackknife standard error of $\frac{{h}_{\mathrm{pleio}}^{2}}{{h}^{2}}$ with respect to the prepruning auxiliary disease sets is greater than 0.5 and/or (2) the scaling coefficient ${\xi }_{c}$ is less than 0.5 (implying that >75% of the initial $\frac{{h}_{\mathrm{pleio}}^{2}}{{h}^{2}}$ estimate is due to uncertainty in genetic correlation matrix estimates). We also apply the pruning procedure if (3) it takes more than 200 iterations to sample a nonsingular ${r}_{g}[{\bf{D}},{\bf{D}}]$ in the Monte Carlo sampling of bias correction, which indicates that the collinearity of auxiliary diseases causes singularity in samples of ${r}_{g}[{\bf{D}},{\bf{D}}]$.

To apply the pruning procedure, we prune the auxiliary diseases by progressively applying stricter ${r}_{g}^{2}$ thresholds between the auxiliary diseases: 0.5, 0.4, 0.3, 0.2 and 0.1. For each ${r}_{g}^{2}$ threshold, when a pair of auxiliary diseases has ${r}_{g}^{2}$ above the threshold, we keep the disease with a larger ${r}_{g}$ *z* score with the target disease. We repeat this pruning step until all three criteria no longer hold, and we report the $\frac{{{h}^{2}}_{\mathrm{pleio}}}{{h}^{2}}$ estimate from the pruned set of auxiliary diseases.

### Pleiotropic phenotypic variance

To compare ${{h}^{2}}_{\mathrm{pleio}}/{h}^{2}$ with the nongenetic variance that can be explained by the same set of auxiliary diseases, we extend our method to estimate pleiotropic phenotypic variance (${{V}^{2}}_{\mathrm{pleio}}$), defined as the proportion of phenotypic variance of target disease liability (${\sigma }_{T}$) that can be explained by any linear combination of liabilities of auxiliary disease (${\sigma }_{{\rm{D}}}$) (Fig. 1b).

We convert the observed-scale phenotypic correlation to liability-scale phenotypic correlation ${r}_{l}$ using Monte Carlo simulations^31^ under the liability threshold model^60^. To model the relationship between liability-scale correlation ${r}_{l}$ and observed-scale correlations ${r}_{o}$ for two binary traits with different prevalences, we simulate liabilities using ${r}_{l}$ on a grid between −1 and 1. For each ${r}_{l}$, we simulate pairs of disease liabilities from a multivariate normal distribution with covariance equal to specified ${r}_{l}$ and then use the liability threshold model to create the binary disease status by thresholding the liabilities on the basis of their respective prevalences. We then calculate the ${r}_{o}$ between the simulated binary traits. This process generates a map between ${r}_{l}$ and ${r}_{o}$ for two disease traits of given prevalences. Using this mapping, we can map the observed ${r}_{o}$ for the 15 UK Biobank diseases to obtain the corresponding ${r}_{l}$. We use the ${r}_{l}$ matrix to estimate ${{V}^{2}}_{\mathrm{pleio}}$ in the same way as pleiotropic shared heritability,

31 $${{V}^{2}}_{\mathrm{pleio}}=1\times {r}_{l}{\left[{{\boldsymbol{\sigma }}}_{{\bf{D}}}{,\sigma }_{T}\right]}^{T}{r}_{l}{\left[{{\boldsymbol{\sigma }}}_{{\bf{D}}},{{\boldsymbol{\sigma }}}_{{\bf{D}}}\right]}^{-1}{r}_{l}[{{\boldsymbol{\sigma }}}_{{\bf{D}}},{\sigma }_{T}],$$

where 1 is the total liability variance for the target disease, ${r}_{l}[{{\boldsymbol{\sigma }}}_{{\bf{D}}},{\sigma }_{T}]$ is the liability-scale phenotypic correlation matrix between target disease and auxiliary diseases, and ${r}_{l}{\left[{{\boldsymbol{\sigma }}}_{{\bf{D}}},{{\boldsymbol{\sigma }}}_{{\bf{D}}}\right]}^{-1}$ is the inverse of liability-scale phenotypic correlation among auxiliary diseases. We estimate the standard error of ${r}_{l}$ and ${{V}^{2}}_{\mathrm{pleio}}$ by jackknifing over 1,000 blocks of individuals across all diseases. We also compare the difference between ${r}_{g}$ and ${r}_{l}$ with a Bonferroni-corrected significant threshold set at *P* < 4.76 × 10^−4^ (0.05/105, where 105 is the number of pairs of the 15 diseases) (Fig. 3 and Supplementary Table 15).

We compare three quantities: (1) $\frac{{{h}^{2}}_{\mathrm{pleio}}}{{h}^{2}}$, the proportion of genetic variance that is pleiotropic, (2) $\frac{{{V}^{2}}_{\mathrm{pleio}}}{{V}^{2}}$, the proportion of liability variance that is shared with the set of auxiliary diseases and (3) $\frac{{{E}^{2}}_{\mathrm{pleio}}}{{E}^{2}}$, the proportion of nongenetic variance (including rare variant heritability and environmental variance) that is shared with the set of auxiliary diseases, calculated as

32 $$\frac{{{E}^{2}}_{\mathrm{pleio}}}{{E}^{2}}=\frac{{{V}^{2}}_{\mathrm{pleio}}-{{h}^{2}}_{\mathrm{pleio}}}{{V}^{2}-{h}^{2}}.$$

We computed the ratios between $\frac{{{h}^{2}}_{\mathrm{pleio}}}{{h}^{\,2}}$ versus $\frac{{{V}^{2}}_{\mathrm{pleio}}}{{V}^{\,2}}$, $\frac{{{E}^{2}}_{\mathrm{pleio}}}{{E}^{\,2}}$ versus $\frac{{{V}^{2}}_{\mathrm{pleio}}}{{V}^{\,2}}$, ${{h}^{2}}_{\mathrm{pleio}}$ versus ${{V}^{2}}_{\mathrm{pleio}}$ and $\frac{{{h}^{2}}_{\mathrm{pleio}}}{{h}^{2}}$ versus $\frac{{{E}^{2}}_{\mathrm{pleio}}}{{E}^{2}}$. Full results are shown in Supplementary Figs. 27–30 and Supplementary Table 26.

### Simulations

We performed simulation using real UK Biobank genotypes from 1,122,329 HapMap3^36^ SNPs across 157,206 unrelated individuals (see below). We simulated 16 diseases with liability-scale heritabilities equal to 0.13 (median of 15 UK Biobank diseases) and prevalences equal to 0.1 (median of 15 UK Biobank diseases); we have also included simulations with heritability *z* scores matching the median value of 15 UK Biobank diseases (Supplementary Figs. 10 and 12). True genetic correlations were set to 0.5 within diseases categories and 0.1 between diseases categories for the first 15 diseases (based on the seven PheCode disease categories from Table 1) and 0.0 for the 16th disease (with all other 15 diseases), which results in five different values of true $\frac{{{h}^{2}}_{\mathrm{pleio}}}{{h}^{2}}$. The proportion of causal SNPs was set to 5% in the primary simulations (and 1% in a secondary simulation). For each simulation, we first simulated the causal effect sizes using the infinitesimal model on the basis of the specified heritability and genetic correlation. Then, we used PLINK2^37^ to compute genetic values (which sum over causal_effect_size × causal_allele_dosage) for 157,206 unrelated individuals and added environmental noise to generate the individual liabilities for the 16 diseases. We used the liability threshold model to generate binary phenotype on the basis of the specified prevalence and computed GWAS summary statistics using PLINK2^37^. We applied cross-trait LDSC^8^ to the GWAS summary statistics for the 1,121,509 SNPs (MAF >0.01) and used reference LD from 1000 Genomes Europeans individuals (9,254,535 SNPs) to estimate genetic correlations. We applied PHBC to estimate $\frac{{{h}^{2}}_{\mathrm{pleio}}}{{h}^{2}}$ and compared it with the true value. We also compared the squared genomic jackknife standard error of $\frac{{{h}^{2}}_{\mathrm{pleio}}}{{h}^{2}}$ with the squared deviation (defined as the squared difference between the estimated post-corrected $\frac{{{h}^{2}}_{\mathrm{pleio}}}{{h}^{2}}$ and true value) to investigate whether the reported standard errors were well calibrated.

We also performed a simulation to validate ${{V}^{2}}_{\mathrm{pleio}}$ estimation. True ${r}_{o}$ was set to 0.5 within diseases categories and 0.1 between diseases categories for the 15 diseases (based on the seven PheCode disease categories). We simulated liabilities for 228,258 individuals (see below). We computed true ${r}_{l}$ and true ${{V}^{2}}_{\mathrm{pleio}}$ on the basis of the simulated liabilities. Then, we used the liability threshold model to generate binary phenotypes on the basis of the empirical prevalences of the 15 UK Biobank diseases. We applied our method to the simulated binary phenotypes to estimate ${{V}^{2}}_{\mathrm{pleio}}$ and compared it to with the true value.

### UK Biobank data

For disease phenotype preprocessing, we collected diagnoses from both inpatient data and primary care data. An individual is defined as a case if there is a diagnosis from either inpatient data or primary care data. We selected 228,258 samples with both primary care and hospital inpatient records. Diagnoses from hospital inpatient records were obtained from Hospital Episode Statistics (HES) for England, which are recorded as the International Classification of Diseases (ICD-10) system codes. Diagnoses from primary care data were retrieved from a subset of UK Biobank samples (see below), which are recorded in Read Codes v.2 (Read v.2) and Read Codes Clinical Terms v.3 (Read CTv.3). We mapped primary care data from Read v.2 to ICD-10 codes, and combined them with the ICD-10 codes from the HES data. We kept ICD-10 codes starting with the letters A to N, which are disease codes. Then, we mapped the ICD-10 records to PheCode system to obtain the phenotype definition^33,61^ and selected the PheCode phenotypes with >1% prevalence in the 228,258 samples.

We selected 15 relatively independent heritable diseases with heritability *z* scores larger than 6 (heritabilities were computed using cross-trait LDSC^8^), which are distributed across seven PheCode disease categories. We note that we did not include any UK Biobank autoimmune diseases or cancers because they did not meet our criteria of prevalence >1% and heritability *z* score >6. We used 228,258 samples to compute pleiotropic phenotypic variance across these 15 UK Biobank disease phenotypes. Detailed information on phenotype codes, prevalences and categories are presented in Table 1.

We selected 157,206 unrelated (defined as having less than 11 putative third-degree relatives in the kinship table) British ancestry individuals from the 228,258 samples to compute GWAS summary statistics for 15 UK Biobank diseases, where we used 1,141,346 HapMap3 SNPs (see below; 1,117,934 of them overlap with the SNPs used in simulations)^36^. We used BOLT-LMM^62,63^ to compute summary statistics. As BOLT-LMM test statistics are well calibrated for traits with a case fraction of at least 10%^63^, for diseases with prevalence lower than 10%, we matched each case with a subset of nine randomly sampled controls, to avoid miscalibration in unbalanced case–control samples.

Detailed genotyping and quality control procedures in the UK Biobank have been described previously^32^. We excluded SNPs with call rates <95%, minor allele frequency <0.1% and deviation from the Hardy–Weinberg equilibrium with *P* < 1 × 10^−10^. We then generated the PLINK-format data for BOLT-LMM using two steps: (1) subset to the 157,206 unrelated British individuals with genotype data and (2) subset the SNPs to HapMap3 SNPs and using PLINK2 to LD prune at ${r}^{2} < 0.8$ to obtain the set of relatively independent SNPs (PLINK2 command ‘-indep-pairwise 50 5 0.8’). A total of 470,851 SNPs from 157,206 samples remained after the pruning step and were carried forward to BOLT-REML for variance components analysis; we reused the full 1,141,346 HapMap3 SNPs to compute the BOLT-LMM association test statistics.

We selected 17 quantitative traits for relevance to disease from refs. ^34,35^ for the same set of individuals (avg *n* = 230K) and corrected for medication use, as follows. We corrected blood biochemistry measurements for cholesterol, hypertensive and diabetic medications: total cholesterol, low-density lipoprotein (LDL), and triglyceride levels were corrected for lipid-lowering medications^64^; systolic blood pressure and diastolic blood pressure were corrected for HTN medications^65^; and HbA1c level was corrected for noninsulin diabetic medications and insulin drugs^66^. We verified that the correlation between the corrected quantitative traits and corresponding binary traits was higher compared with uncorrected quantitative traits. We also included EA (years of education) to evaluate the impact of socioeconomic status. Detailed information on trait names, categories, heritability estimates and genetic correlation estimates for these 17 quantitative traits is presented in Supplementary Tables 2 and 16.

### GWAS summary statistics for 30 diseases from publicly available GWAS meta-analyses

We collected publicly available GWAS summary statistics for 30 relatively independent heritable diseases under the same criteria (heritability *z* score >6 and ${{r}_{g}}^{2} < 0.5$) that are primarily from European ancestry. We note that some of these 30 publicly available disease summary statistics are meta-analyses that include UK Biobank samples. The 30 diseases are assigned to ten PheCode categories^33^. Details of phenotype information, heritability and genetic correlation estimates from cross-trait LDSC for these 30 diseases are presented in Supplementary Tables 3 and 16.

### Analyses with one or more disease categories excluded from the set of auxiliary diseases

We performed leave-category-out analyses to investigate the change in $\frac{{{h}^{2}}_{\mathrm{pleio}}}{{h}^{2}}$ when removing PheCode categories. First, we estimated the $\frac{{{h}^{2}}_{\mathrm{pleio}}}{{h}^{2}}$ with respect to three auxiliary disease sets: (1) all auxiliary diseases, (2) all diseases excluding the auxiliary diseases in the target disease category and (3) all diseases excluding the auxiliary diseases in the target disease category and one other category. We implemented leave-category-out analyses in the PHBC software.

To evaluate the contribution of a single PheCode category to $\frac{{{h}^{2}}_{\mathrm{pleio}}}{{h}^{2}}$, we computed the reduction in $\frac{{{h}^{2}}_{\mathrm{pleio}}}{{h}^{2}}$ when removing the auxiliary PheCode category. To remove the impact of randomness in Monte Carlo bias correction when computing the reduction, we first computed the difference in $\frac{{{h}^{2}}_{\mathrm{pleio}}}{{h}^{2}}$ with respect to all auxiliary diseases versus $\frac{{{h}^{2}}_{\mathrm{pleio}}}{{h}^{2}}$ with respect to auxiliary diseases excluding a PheCode category before bias correction. We multiplied the difference by the scaling coefficient ${\xi }_{c}^{2}$ of $\frac{{{h}^{2}}_{\mathrm{pleio}}}{{h}^{2}}$ with respect to all auxiliary diseases, which represented the contribution of an auxiliary PheCode category to the $\frac{{{h}^{2}}_{\mathrm{pleio}}}{{h}^{2}}$ with respect to all auxiliary diseases. We reported the jackknife standard error of the before-bias-correction difference of $\frac{{{h}^{2}}_{\mathrm{pleio}}}{{h}^{2}}$ as the standard error of the reduction, which is an approximately well-calibrated standard error shown by simulations (Supplementary Figs. 18 and 19 and Supplementary Table 13).

### Alternative approaches for estimating *r*_*g*_

To validate the ${r}_{g}$ estimated from cross-trait LDSC without constrained intercept, we used BOLT-REML and cross-trait LDSC using constrained intercept to estimate ${r}_{g}$. We applied BOLT-REML to individual data using 470,851 SNPs across 157,206 unrelated individuals of British ancestry to estimate ${r}_{g}$ among these 15 UK Biobank diseases. We constrained the heritability intercept in cross-trait LDSC to be 1. We also computed the analytical intercept for genetic covariance considering sample overlap for each disease pair using equation 16 in the Supplementary Note of ref. ^8^. The constrained intercepts for genetic covariance for each disease pair are presented in Supplementary Table 27.

### Reporting summary

Further information on research design is available in the Nature Portfolio Reporting Summary linked to this article.

## Online content

Any methods, additional references, Nature Portfolio reporting summaries, source data, extended data, supplementary information, acknowledgements, peer review information; details of author contributions and competing interests; and statements of data and code availability are available at 10.1038/s41588-026-02607-w.

## Supplementary information

Supplementary Information Supplementary Notes, Figs. 1–31 and captions for Supplementary Tables 1–32.

Reporting Summary

Peer Review File

Supplementary Tables Supplementary Tables 1–32.

## Data Availability

Summary association statistics for all diseases/traits analyzed in this study have been made publicly available at https://alkesgroup.broadinstitute.org/PHBC/.

## Code availability

Open-source software implementing PHBC is available via Github at https://github.com/yjzhao1004/pleioh2g and was published on CRAN (https://cran.r-project.org/web/packages/pleioh2g). We have also deposited PHBC codes at 10.5281/zenodo.19550445. Other publicly available software packages used in this study are listed below: GWAS summary statistics were computed using PLINK2 (https://www.cog-genomics.org/plink/2.0/) and BOLT-LMM v2.4.1 (https://alkesgroup.broadinstitute.org/BOLT-LMM/downloads/); heritability and genetic correlation were estimated using LDSC (https://github.com/bulik/ldsc); and we used the ldscr R package (https://github.com/mglev1n/ldscr).
