# Supplementary material for: Pleiotropic shared heritability quantifies the shared genetic variance of common diseases
Source: Nat Genet. 2026 Jun 9;58(6):1248–57. doi: 10.1038/s41588-026-02607-w (PMC13256260; doi:10.1038/s41588-026-02607-w)
Supplement: Supplementary file 1 — Supplementary Notes, Figs. 1–31 and captions for Supplementary Tables 1–32. [file 41588_2026_2607_MOESM1_ESM.pdf]

nature genetics

Supplementary information

https://doi.org/10.1038/s41588-026-02607-w

Pleiotropic shared heritability quantifies the shared genetic variance of common diseases

In the format provided by the authors and unedited

Table of Contents

Supplementary Notes 5

Secondary simulation analyses 5

Secondary analyses using 15 diseases from the UK Biobank 5

Secondary analyses in comparing shared genetic and non-genetic variance 7

Additional discussion 7

References for supplementary note 8

Supplementary Table Captions 9

Supplementary Table 1. List of 15 UK Biobank diseases in primary analyses. 9

Supplementary Table 2. List of 17 UK Biobank quantitative traits. 9

Supplementary Table 3. List of 30 diseases from publicly available GWAS meta-analyses. 9

Supplementary Table 4. Simulation results when rg within-disease categories equals to 0.5 and rg between-disease categories equals to 0.1. 9

Supplementary Table 5. Simulation results when rg within-disease categories equals to 0.4 and rg between-disease categories equals to 0.1. 10

Supplementary Table 6. Simulation results when rg within-disease categories equals to 0.3 and rg between-disease categories equals to 0.1. 10

Supplementary Table 7. Simulation results when rg within-disease categories equals to 0.2 and rg between-disease categories equals to 0.1. 11

Supplementary Table 8. Simulation results with the true liability-scale heritability set to 0.25 (instead of 0.13). 12

Supplementary Table 9. Simulation results with the true liability-scale heritability set to 0.06 (instead of 0.13). 12

Supplementary Table 10. Simulation results with the prevalence set to 0.05 (instead of 0.1). 13

Supplementary Table 11. Simulation results with the proportion of causal SNPs set to 1% (instead of 5%). 13

Supplementary Table 12. Simulation results when rg within-disease categories equals to 0.5 and rg between-disease categories equals to 0.1, without pruning procedure. 14

Supplementary Table 13. Simulations results of the reduction in $h2pleio/h2$ and its standard error in analyses with one auxiliary disease category removed. 14

Supplementary Table 14. Genetic correlations between 15 UK Biobank diseases. 15

Supplementary Table 15. Genetic correlations and liability correlations between 15 UK Biobank diseases. 15

Supplementary Table 16. Genetic correlations across all 15 UK Biobank diseases, 30 non-UK Biobank diseases, 17 UK Biobank quantitative traits. 15

Supplementary Table 17. $h2pleio/h2$ before and after bias correction for all 15 UK Biobank diseases and the average with respect to 4 types of auxiliary diseases/traits. 15

Supplementary Table 18. Differences between auxiliary diseases choices for average estimates in Figure 4 and Figure 6. 16

Supplementary Table 19. $2pleio/h2$ estimates using the single auxiliary category and the reduction in $h2pleio/h2$ when one auxiliary disease category removed for all 15 UK Biobank target diseases. 16

Supplementary Table 20. Comparison of r_g from BOLT-REML and r_g from cross-trait LDSC with 4 options of constraining intercept. 16

Supplementary Table 21. Comparison of $h2pleio/h2$ estimated from BOLT-REML r_g and $h2pleio/h2$ estimated from cross-trait LDSC r_g with 4 options of constraining intercept. 17

Supplementary Table 22. $h2pleio/h2$ before and after bias correction for all 15 UK Biobank diseases and the average with respect to four types of auxiliary disease sets: 15 UK Biobank auxiliary diseases, 30 non-UK Biobank auxiliary diseases, all 45 auxiliary diseases, and all 62 auxiliary diseases and quantitative traits. 17

Supplementary Table 23. $h2pleio/h2$ before and after bias correction for all 30 non-UK Biobank diseases and the average with respect to four types of auxiliary disease sets: 15 UK Biobank auxiliary diseases, 30 non-UK Biobank auxiliary diseases, all 45 auxiliary diseases and all 62 auxiliary diseases and quantitative traits. 17

Supplementary Table 24. $h2pleio/h2$ estimates using the single auxiliary category and the reduction in $h2pleio/h2$ when one auxiliary disease category removed for all 45 UK Biobank target diseases + 30 non-UK Biobank target diseases. 17

Supplementary Table 25. Simulation results of $V2pleio/V2$ and its standard error. 18

Supplementary Table 26. Comparing $h2pleio/h2\ vs.\ V2pleio/V2\ vs.\ E2pleio/E2$. 18

Supplementary Table 27. The constrained and estimated intercepts for genetic covariance for each disease pair. 19

Supplementary Table 28. Comparison of $h2pleio/h2$ between estimate using only EA and reduction when removing the EA. 19

Supplementary Table 29. Changes of $h2pleio/h2$ across 15 UK Biobank diseases for various $rg2$ thresholds between target and auxiliary diseases. 19

Supplementary Table 30. Comparison between $h2pleio/h2$ with the sum of (bias-corrected) $rg2$ across auxiliary diseases, for different target diseases and auxiliary disease sets. 20

Supplementary Table 31. Correlation between $h2pleio/h2$ and the sum of (bias-corrected and without-bias-corrected) $rg2$ across auxiliary diseases, across the 14 auxiliary disease sets. 20

Supplementary Table 32. Single-trait LDSC intercept, GWAS average chi-square statistics and attenuation ratio of 15 UK biobank diseases. 20

Supplementary Figures *21*

Supplementary Figure 1. Estimated standard errors of $h2pleio/h2$ from PHBC were approximately well-calibrated. 21

Supplementary Figure 2. Simulations with rg within-disease categories equal to 0.4 (and rg between-disease categories still equal to 0.1). 22

Supplementary Figure 3. Estimated standard errors of $h2pleio/h2$ from PHBC were approximately well-calibrated in simulations with rg within-disease categories equal to 0.4.............................. 23

Supplementary Figure 4. Simulations with rg within-disease categories equal to 0.3 (and rg between-disease categories still equal to 0.1). 24

Supplementary Figure 5. Estimated standard errors of $h2pleio/h2$ from PHBC were approximately well-calibrated in simulations with rg within-disease categories equal to 0.3.............................. 25

Supplementary Figure 6. Simulations with rg within-disease categories equal to 0.2 (and rg between-disease categories still equal to 0.1). 26

Supplementary Figure 7. Estimated standard errors of $h2pleio/h2$ from PHBC were approximately well-calibrated in simulations with rg within-disease categories equal to 0.2.............................. 27

Supplementary Figure 8. PHBC corrects the upwards bias in simulations with the true liability-scale heritability set to 0.25 (instead of 0.13)... 28

Supplementary Figure 9. Estimated standard errors of $h2pleio/h2$ from PHBC were approximately well-calibrated in simulations with the true liability-scale heritability set to 0.25. 29

Supplementary Figure 10. PHBC corrects the upwards bias in simulations with the true liability-scale heritability set to 0.06 (instead of 0.13)... 30

Supplementary Figure 11. Estimated standard errors of $h2pleio/h2$ from PHBC were conservative in simulations with the true liability-scale heritability set to 0.06. 31

Supplementary Figure 12. PHBC corrects the upwards bias in simulations with the prevalence set to 0.05 (instead of 0.1). 32

Supplementary Figure 13. Estimated standard errors of $h2pleio/h2$ from PHBC were approximately well-calibrated in simulations with the prevalence set to 0.05. 33

Supplementary Figure 14. PHBC corrects the upwards bias in simulations with the proportion of causal SNPs set to 1% (instead of 5%).. 34

Supplementary Figure 15. Estimated standard errors of $h2pleio/h2$ from PHBC were approximately well-calibrated in simulations with the proportion of causal SNPs set to 1%. 35

Supplementary Figure 16. PHBC corrects the upwards bias in simulations without pruning procedure. 36

Supplementary Figure 17. Estimated standard errors of $h2pleio/h2$ from PHBC were approximately well-calibrated in simulations without pruning procedure. 37

Supplementary Figure 18. Simulations of the reduction in $h2pleio/h2$ in analyses with one auxiliary disease category removed. 38

Supplementary Figure 19. Estimated standard errors of the reduction in $h2pleio/h2$ when removing one auxiliary disease category were approximately well-calibrated. 39

Supplementary Figure 20. Distribution of $h2pleio/h2$ across Phecode disease categories........... 43

Supplementary Figure 21. Comparison on liability-scale heritability and $h2pleio/h2$ across the 15 UK Biobank diseases. 45

Supplementary Figure 22. Comparison on r_g from BOLT-REML and r_g from cross-trait LDSC with 4 options of constraining intercept. **46**

Supplementary Figure 23. Comparison on $h2pleio/h2$ estimated from BOLT-REML r_g and $h2pleio/h2$ estimated from cross-trait LDSC r_g with 4 options of constraining intercept. **47**

Supplementary Figure 24. Estimates of genetic correlation of 30 non-UK Biobank diseases. **48**

Supplementary Figure 25. Distribution of $h2pleio/h2$ across Phecode disease categories using all 45 auxiliary diseases. **54**

Supplementary Figure 26. Calibration of estimated standard errors of $V2pleio/V2$. **56**

Supplementary Figure 27. Scatter plot of $h2pleio/h2$ vs. $V2pleio/V2$ for the 15 UK Biobank diseases. **57**

Supplementary Figure 28. Scatter plot of $V2pleio/V2$ vs. $E2pleio/E2$ for the 15 diseases. **59**

Supplementary Figure 29. Scatter plot of $h2pleio$ vs. $V2pleio$ for the 15 diseases. **60**

Supplementary Figure 30. Scatter plot of $h2pleio/h2$ vs. $E2pleio/E2$ for the 15 diseases. **61**

Supplementary Figure 31. Scatter plot of liability-scale heritability and the ratio of $h2pleio/h2$ to $V2pleio/V2$ across the 15 UK Biobank diseases. **62**

Supplementary Notes

Secondary simulation analyses

We performed six secondary analyses. First, we performed simulations with the proportion of causal SNPs set to 1% (instead of 5%), and determined the results were similar to **Figure 2** (**Supplementary Figures 14-15** and **Supplementary Table 11**). Second, we simulated pleiotropic architectures with r_g within disease categories equal to 0.4, 0.3, or 0.2 (instead of 0.5) (and r_g between disease categories still equal to 0.1), and observed approximately unbiased results (**Supplementary Figures 2-7** and **Supplementary Tables 5-7**). Third, we performed simulations with liability-scale heritabilities equal to 0.25 or 0.06 (instead of 0.13). We observed approximately unbiased results with liability-scale heritability equal to 0.25 (**Supplementary Figures 8-9** and **Supplementary Table 8**). With liability-scale heritability equal to 0.06, we observed modest downward bias for values of $h^2{}_{pleio}/h^2$ above 25% and modest upward bias for values below 5%, with average bias of -0.011 (s.e. 0.0021) (**Supplementary Figures 10-11** and **Supplementary Table 9)**. Fourth, we performed simulations with the prevalence set to 0.05 (instead of 0.1), and determined the results were similar to **Figure 2** (**Supplementary Figures 12-13** and **Supplementary Table 10**). Fifth, we estimated $h^2{}_{pleio}/h^2$ without pruning highly correlated auxiliary diseases, and we observed that $h^2{}_{pleio}/h^2$ estimates were approximately unbiased (**Supplementary Figures 16-17** and **Supplementary Table 12**). Across these five experiments, we determined that uncorrected estimates of $h^2{}_{pleio}/h^2$ suffered upward bias (ranging from 1.0% (s.e. 0.06%) to 9.9% (s.e. 0.80%)), emphasizing the need for a bias correction procedure. Finally, we performed simulations to evaluate the genomic block-jackknife standard errors on the reduction in $h^2{}_{pleio}/h^2$ in analyses with one auxiliary disease category removed, and determined that standard errors were approximately well-calibrated (**Supplementary Figure 19** and **Supplementary Table 13**).

Secondary analyses using 15 diseases from the UK Biobank

We performed six secondary analyses. First, we investigated whether there is a correlation between liability-scale heritability and $h^2{}_{pleio}/h^2$ across the 15 UK Biobank diseases. We observed no significant correlation (Pearson's r = -0.18 (*P* = 0.52); **Supplementary Figure 21**). Second, we estimated the average $h^2{}_{pleio}/h^2$ across 15 UK Biobank diseases using different $r_g{}^2$ thresholds between target and auxiliary diseases (ranging from 0.1 to 0.8 in increments of 0.05) (**Extended Data Fig. 4** and **Supplementary Table 29**). Average $h^2{}_{pleio}/h^2$ increased at $r_g{}^2$ thresholds larger than 0.5, but we consider this increase to be not biologically meaningful because most instances of $r_g{}^2$ > 0.5 involved auxiliary traits that *are not biologically distinct* from the target trait, e.g. hypertension-diastolic blood pressure ($r_g{}^2$ = 0.72), hypertension-systolic blood pressure ($r_g{}^2$ = 0.72), type 2 diabetes-glucose ($r_g{}^2$ = 0.69), type 2 diabetes-HbA1C ($r_g{}^2$ = 0.79). In addition, average $h^2{}_{pleio}/h^2$ decreased at $r_g{}^2$ thresholds smaller than 0.5, but all instances of 0.25 < $r_g{}^2$ < 0.5 involved auxiliary traits that *are biologically distinct* from the target

trait: coronary atherosclerosis-hypertension (r_g^2 = 0.25), coronary atherosclerosis-type 2 diabetes (r_g^2 = 0.25), hypertension-type 2 diabetes (r_g^2 = 0.26), depression-tobacco use disorder (r_g^2 = 0.35), gastroesophageal reflux disease (GERD)-tobacco use disorder (r_g^2 = 0.27), obesity-type 2 diabetes (r_g^2 = 0.36), obesity-respiratory diseases (r_g^2 = 0.29), obesity- HbA1c (r_g^2 = 0.26), obesity-glucose (r_g^2 = 0.26), type 2 diabetes-body WHR (r_g^2 = 0.36), type 2 diabetes-BMI (r_g^2 = 0.34), type 2 diabetes-triglycerides (r_g^2 = 0.31), gastroesophageal reflux disease (GERD)-depression (r_g^2 = 0.40), gastroesophageal reflux disease (GERD)-respiratory diseases (r_g^2 = 0.39) and depression-respiratory diseases (r_g^2 = 0.32). These findings support the use of an estimand based on r_g^2 < 0.5 between target and auxiliary diseases/traits. Third, we compared h^2_{pleio}/h^2 to the sum of (bias-corrected) r_g^2 across auxiliary diseases, across auxiliary disease sets for a given target disease. For each of 15 UK Biobank target diseases, we considered 14 auxiliary disease sets (containing 1, …, 14 auxiliary diseases), starting with the auxiliary disease with highest r_g^2 with the target disease and iteratively adding auxiliary diseases in the order of decreasing r_g^2 with the target disease; we then computed the correlation between h^2_{pleio}/h^2 and the sum of (bias-corrected) r_g^2 across the 14 auxiliary disease sets. The average correlation (across 15 target diseases) was 0.28, implying that h^2_{pleio}/h^2 captures different information than the sum of (bias-corrected) r_g^2 (**Supplementary Tables 30-31**). Fourth, we estimated genetic correlations by applying BOLT-REML[1] to individual-data, instead of applying cross-trait LDSC[2] to summary-level data. We determined that estimates of genetic correlation and h^2_{pleio}/h^2 were broadly consistent (**Supplementary Figures 22-23**, **Supplementary Tables 20-21**). Fifth, we estimated genetic correlations using three modified versions of cross-trait LDSC: constraining heritability intercept, constraining genetic covariance intercept, and constraining both intercepts. We determined that all three modified versions deviated from BOLT-REML results in estimates of genetic correlation and h^2_{pleio}/h^2 (**Supplementary Figures 22-23**, **Supplementary Tables 20-21**), supporting our primary use of the default version of cross-trait LDSC. Sixth, we assessed the presence of two types of population structure that may impact h^2_{pleio}/h^2 estimates: (i) population structure impacting a single trait, which inflates GWAS chi-square statistics of that trait, and (ii) population structure that jointly impacts two traits, which inflates or deflates the products of z-scores for those two traits. The first type of population structure is captured by the intercept of LD score regression applied to a single trait[3], aiming to prevent inflation in single-trait SNP-heritability estimates. The second type of population structure is captured by the intercept of cross-trait LD score regression applied to two traits, aiming to prevent inflation or deflation in genetic covariance estimates[2]. For the first type of population structure, we observed that the average single-trait intercept across 15 UK Biobank diseases was 1.02 (s.d. = 0.02), with an average attenuation ratio (the ratio of inflation in intercept to inflation in chi-square statistics[4]) of 0.11 (s.d. = 0.09) (**Supplementary Table 32)**, implying a limited impact of population structure and other confounders on GWAS chi-square statistics for a single trait. For the second type of population structure, we compared empirical cross-trait intercepts (impacted by both sample overlap and population structure) to analytical cross-trait intercepts (impacted by sample overlap only) across 105 pairs of UK Biobank diseases. We found these to be concordant, with average empirical cross-trait intercept of 0.0461 (s.d. = 0.0464), average analytical intercept of 0.0520 (s.d. = 0.0522), and average difference of −0.0059 (s.d. = 0.0161)

(**Supplementary Table 27)**, implying a limited impact of population structure and other confounders on products of z-scores for two traits.

Secondary analyses in comparing shared genetic and non-genetic variance

We performed a secondary analysis to investigate whether there is a correlation between liability-scale heritability and the ratio of $h^2{}_{pleio}/h^2$ to $V^2{}_{pleio}/V^2$ across the 15 UK Biobank diseases. We observed a negative correlation (Pearson's r = -0.65, *P* = 0.008; **Supplementary Figure 31**). The p-value of 0.008 is anti-conservative, as it treats the 15 diseases as independent when they are in fact correlated, and it is thus unclear whether the correlation is statistically significant.

Additional discussion

We note several limitations of our work. First, jackknife standard errors on estimates of $h^2{}_{pleio}$ do not account for stochasticity that may in principle be introduced by pruning genetically correlated auxiliary diseases to ensure numerical stability; however, we anticipate that the set of pruned diseases would exhibit minimal variation across genomic jackknife blocks, due to limited variation in genetic correlation estimates across blocks. Second, the definition of $h^2{}_{pleio}$ is with respect to a specific set of auxiliary traits, and results depends on the set of auxiliary traits; however, the magnitude of the increase in $h^2{}_{pleio}/h^2$ shows diminishing increases as the number of auxiliary traits increases (**Figure 6A** and **Figure 6B**), implying that expanding the set of auxiliary traits is unlikely to substantially increase the $h^2{}_{pleio}/h^2$ to the high level of T2D and MDD for most other diseases; however, we note that further increases in $h^2{}_{pleio}/h^2$ for these diseases are possible if additional auxiliary traits are incorporated. In addition, removing disease categories from the set of auxiliary diseases does not reduce $h^2{}_{pleio}/h^2$ much, implying that individual auxiliary diseases do not have a large impact on $h^2{}_{pleio}/h^2$ when a sufficiently large set of auxiliary diseases are included. Third, genetic correlation estimates include the effects of assortative mating, which can be substantial for some diseases/traits[5] (e.g. educational attainment); our results do not distinguish between genetic correlation due to shared causal effects vs. genetic correlation due to assortative mating, which would require applying new methods to individual-level data[6]; however, we believe that cross-trait assortative mating effects involving disease risk (e.g. with educational attainment) are likely to be modest in most cases[5]. Fourth, definition and estimation of $h^2{}_{pleio}$ is with respect to a specific phenotype definition in a specific cohort. Ascertainment bias will carry through to our definition and estimation of $h^2{}_{pleio}$; in particular, UK Biobank is known to be subject to participation bias[7,8]. Phenotypic misclassification will also carry through to our definition and estimation of $h^2{}_{pleio}$; we have restricted our analysis to individuals in UK Biobank with both primary care data and hospital record data to limit the impact of phenotypic misclassification. However, shared controls do not

impact definition or estimation of h^2_{pleio}, because definition is with respect to a population with infinite sample size and cross-trait LD score regression, which we use to estimate genetic correlations, is robust to shared controls in a finite sample. Finally, we restricted our analyses to individuals of European ancestry (represented in large sample size in UK Biobank), but it is important to analyze more diverse cohorts[9,10], particularly for diseases whose prevalence varies across populations.

References for supplementary note

1. Loh, P.-R. *et al.* Contrasting genetic architectures of schizophrenia and other complex diseases using fast variance-components analysis. *Nat Genet* **47**, 1385–1392 (2015).
2. Bulik-Sullivan, B. *et al.* An atlas of genetic correlations across human diseases and traits. *Nat Genet* **47**, 1236–1241 (2015).
3. Bulik-Sullivan, B. K. *et al.* LD Score regression distinguishes confounding from polygenicity in genome-wide association studies. *Nat Genet* **47**, 291–295 (2015).
4. Loh, P.-R., Kichaev, G., Gazal, S., Schoech, A. P. & Price, A. L. Mixed-model association for biobank-scale datasets. *Nat Genet* **50**, 906–908 (2018).
5. Border, R. *et al.* Cross-trait assortative mating is widespread and inflates genetic correlation estimates. *Science* **378**, 754–761 (2022).
6. Zhang, Y. *et al.* The contribution of gametic phase disequilibrium to the heritability of complex traits. *Nat Genet* https://doi.org/10.1038/s41588-025-02192-4 (2025) doi:10.1038/s41588-025-02192-4.
7. Benonisdottir, S. & Kong, A. Studying the genetics of participation using footprints left on the ascertained genotypes. *Nat Genet* **55**, 1413–1420 (2023).
8. Schoeler, T. *et al.* Participation bias in the UK Biobank distorts genetic associations and downstream analyses. *Nat Hum Behav* **7**, 1216–1227 (2023).
9. Zhou, W. *et al.* Global Biobank Meta-analysis Initiative: Powering genetic discovery across human disease. *Cell Genomics* **2**, 100192 (2022).
10. The All of Us Research Program Genomics Investigators *et al.* Genomic data in the All of Us Research Program. *Nature* **627**, 340–346 (2024).

Supplementary Table Captions

Supplementary Table 1. List of 15 UK Biobank diseases in primary analyses.

We select 15 relatively independent heritable UK Biobank diseases with > 1% prevalence in 228,258 samples (see **Methods**) and heritability *z* scores larger than 6. We restrict their squared genetic correlations to be smaller than 0.5 among 15 diseases (**Supplementary Table 14**) (both heritability and genetic correlation are computed using cross-trait LDSC). We obtain their PheCode definitions by mapping their ICD-10 records to PheCode system (See **Methods**). These 15 diseases are distributed across 7 PheCode disease categories. Detailed sample size using in computing GWAS are described in **Methods**. We report the disease name, PheCode, observed-scale and liability-scale SNP-heritability, standard error, z-score, category, prevalence and ICD-10 code.

Supplementary Table 2. List of 17 UK Biobank quantitative traits.

We select 17 relatively independent heritable UK Biobank quantitative traits with heritability *z* scores larger than 6 and squared genetic correlation smaller than 0.5 (**Supplementary Table 16**). The preprocess of these 17 traits is described in **Methods**. We report the trait name, observed-scale SNP-heritability, standard error, z-score, and category.

Supplementary Table 3. List of 30 diseases from publicly available GWAS meta-analyses.

We selected publicly available GWAS summary statistics from 30 relatively independent heritable diseases with heritability *z* scores larger than 6 and squared genetic correlation smaller than 0.5 (**Supplementary Table 16**). These 30 GWAS summary statistics are all from European ancestry. For brevity, we subsequently refer to the 30 as 'non-UK Biobank' diseases (while duly noting that a subset of the latter includes both non-UK Biobank and UK Biobank data). These 30 non-UKB diseases are assigned to 10 PheCode categories. We reported the trait name, trait identifier used to index traits in plots and tables, source reference, sample size, observed-scale SNP-heritability, standard error, z-score and category.

Supplementary Table 4. Simulation results when r_g within-disease categories equals to 0.5 and r_g between-disease categories equals to 0.1.

The first column for **sub-tables A-D** lists the disease PheCodes that have the same true $h^2{}_{pleio}/h^2$ based on the specified genetic architecture. In the **sub-tables A-B**, we show the estimated $h^2{}_{pleio}/h^2$, true $h^2{}_{pleio}/h^2$ and their absolute bias for 5 different values both before bias correction and after bias correction. Estimated $h^2{}_{pleio}/h^2$ before bias correction shows an upward bias, but it is approximately unbiased after bias correction. For the zero-trait

simulations, we observe no inflation of the type I error rate (one-sided type I error rate = 0.01; two-sided type I error rate = 0). These results correspond to **Figure 2**. In the **sub-tables C-D**, we report the calibration of s.e. using four ratios: (1) (average jackknife s.e.) / (standard deviation); (2) (average jackknife s.e.) / ($\sqrt{average\ squared\ deviation}$); (3) (average jackknife s.e.^2) / (squared standard deviation); (4) (average jackknife s.e.^2) / (average squared deviation). Deviation is the difference between true ${h^2}_{pleio}/h^2$ and the average estimated ${h^2}_{pleio}/h^2$. The estimated standard errors of ${h^2}_{pleio}/h^2$ after bias correction were approximately well-calibrated. For maximum transparency, we report calibration using above four ratios (1.07 or 0.95 or 1.27 or 0.93). We note that $deviation^2 = s.d.^2 + bias^2$, therefore ratio (2) is smaller than ratio (1), and ratio (4) is smaller than ratio (3). Results for simulated diseases that have the same true ${h^2}_{pleio}/h^2$ are averaged which results in five different points from 16 simulated diseases. These results correspond to **Supplementary Figure 1**. In the **sub-tables E-F**, we report the same four ratios, but for the 16 individual diseases (0.99 or 0.95 or 1.03 or 0.93).

Supplementary Table 5. Simulation results when r_g within-disease categories equals to 0.4 and r_g between-disease categories equals to 0.1.

The first column for **sub-tables A-D** lists the disease PheCodes that have the same true ${h^2}_{pleio}/h^2$ based on the specified genetic architecture. In the **sub-tables A-B**, we show the estimated ${h^2}_{pleio}/h^2$, true ${h^2}_{pleio}/h^2$ and their absolute bias for 5 different values both before bias correction and after bias correction. Estimated ${h^2}_{pleio}/h^2$ before bias correction shows an upward bias, but it is approximately unbiased after bias correction. For the zero-trait simulations, we observe no inflation of the type I error rate (one-sided type I error rate = 0.02; two-sided type I error rate = 0). These results correspond to **Supplementary Figure 2**. In the **sub-tables C-D**, we report the calibration of s.e. using four ratios: (1) (average jackknife s.e.) / (standard deviation); (2) (average jackknife s.e.) / ($\sqrt{average\ squared\ deviation}$); (3) (average jackknife s.e.^2) / (squared standard deviation); (4) (average jackknife s.e.^2) / (average squared deviation). Deviation is the difference between true ${h^2}_{pleio}/h^2$ and the average estimated ${h^2}_{pleio}/h^2$. The estimated standard errors of ${h^2}_{pleio}/h^2$ after bias correction were approximately well-calibrated. For maximum transparency, we report four different ratios (1.03 or 0.93 or 1.22 or 0.93) based on the above four different ways to compute this ratio. We note that $deviation^2 = s.d.^2 + bias^2$, therefore ratio (2) is smaller than ratio (1), and ratio (4) is smaller than ratio (3). These results correspond to **Supplementary Figure 3**. In the **sub-tables E-F**, we report the same four ratios, but for the 16 individual diseases (0.95 or 0.92 or 0.99 or 0.90).

Supplementary Table 6. Simulation results when r_g within-disease categories equals to 0.3 and r_g between-disease categories equals to 0.1.

The first column for **sub-tables A-D** lists the disease PheCodes that have the same true ${h^2}_{pleio}/h^2$ based on the specified genetic architecture. In the **sub-tables A-B**, we show the

estimated $h^2{}_{pleio}/h^2$, true $h^2{}_{pleio}/h^2$ and their absolute bias for 5 different values both before bias correction and after bias correction. Estimated $h^2{}_{pleio}/h^2$ before bias correction shows an upward bias, but it is approximately unbiased after bias correction. For the zero-trait simulations, we observe no inflation of the type I error rate (one-sided type I error rate = 0.02; two-sided type I error rate = 0). These results correspond to **Supplementary Figure 4**. In the **sub-tables C-D**, we report the calibration using four ratios: (1) (average jackknife s.e.) / (standard deviation); (2) (average jackknife s.e.) / ($\sqrt{average\ squared\ deviation}$); (3) (average jackknife s.e.^2) / (squared standard deviation); (4) (average jackknife s.e.^2) / (average squared deviation). Deviation is the difference between true $h^2{}_{pleio}/h^2$ and the average estimated $h^2{}_{pleio}/h^2$. The estimated standard errors of $h^2{}_{pleio}/h^2$ after bias correction were approximately well-calibrated. For maximum transparency, we report four different ratios (1.10 or 1.00 or 1.27 or 1.03) based on the above four different ways to compute this ratio. We note that $deviation^2 = s.d.^2 + bias^2$, therefore ratio (2) is smaller than ratio (1), and ratio (4) is smaller than ratio (3). Results for simulated diseases that have the same true $h^2{}_{pleio}/h^2$ are averaged which results in five different points from 16 simulated diseases. These results correspond to **Supplementary Figure 5**. In the **sub-tables E-F**, we report the same four ratios, but for the 16 individual diseases (1.05 or 1.02 or 1.15 or 1.07)**.**

Supplementary Table 7. Simulation results when r_g within-disease categories equals to 0.2 and r_g between-disease categories equals to 0.1.

The first column for **sub-tables A-D** lists the disease PheCodes that have the same true $h^2{}_{pleio}/h^2$ based on the specified genetic architecture. In the **sub-tables A-B**, we show the estimated $h^2{}_{pleio}/h^2$, true $h^2{}_{pleio}/h^2$ and their absolute bias for 5 different values both before bias correction and after bias correction. Estimated $h^2{}_{pleio}/h^2$ before bias correction shows an upward bias, but it is approximately unbiased after bias correction. For the zero-trait simulations, we observe no inflation of the type I error rate (one-sided type I error rate = 0.03; two-sided type I error rate = 0). These results correspond to **Supplementary Figure 6**. In the **sub-tables C-D**, we report the calibration of s.e. using four ratios: (1) (average jackknife s.e.) / (standard deviation); (2) (average jackknife s.e.) / ($\sqrt{average\ squared\ deviation}$); (3) (average jackknife s.e.^2) / (squared standard deviation); (4) (average jackknife s.e.^2) / (average squared deviation). Deviation is the difference between true $h^2{}_{pleio}/h^2$ and the average estimated $h^2{}_{pleio}/h^2$. The estimated standard errors of $h^2{}_{pleio}/h^2$ after bias correction were approximately well-calibrated. For maximum transparency, we report four different ratios (1.00 or 0.94 or 1.09 or 0.92) based on the above four different ways to compute this ratio. We note that $deviation^2 = s.d.^2 + bias^2$, therefore ratio (2) is smaller than ratio (1), and ratio (4) is smaller than ratio (3). These results correspond to **Supplementary Figure 7**. In the **sub-tables E-F**, we report the same four ratios, but for the 16 individual diseases (0.94 or 0.91 or 0.92 or 0.86).

Supplementary Table 8. Simulation results with the true liability-scale heritability set to 0.25 (instead of 0.13).

The first column for **sub-tables A-D** lists the disease PheCodes that have the same true $h^2{}_{pleio}/h^2$ based on the specified genetic architecture. In the **sub-tables A-B**, we show the estimated $h^2{}_{pleio}/h^2$, true $h^2{}_{pleio}/h^2$ and their absolute bias for 5 different values both before bias correction and after bias correction. Estimated $h^2{}_{pleio}/h^2$ before bias correction shows a slight upward bias, but it is approximately unbiased after bias correction. For the zero-trait simulations, we observe no inflation of the type I error rate (one-sided type I error rate = 0.04; two-sided type I error rate = 0.01). These results correspond to **Supplementary Figure 8**. In the **sub-tables C-D**, we report the calibration of s.e. using four ratios: (1) (average jackknife s.e.) / (standard deviation); (2) (average jackknife s.e.) / ($\sqrt{average\ squared\ deviation}$); (3) (average jackknife s.e.^2) / (squared standard deviation); (4) (average jackknife s.e.^2) / (average squared deviation). Deviation is the difference between true $h^2{}_{pleio}/h^2$ and the average estimated $h^2{}_{pleio}/h^2$. The estimated standard errors of $h^2{}_{pleio}/h^2$ after bias correction were approximately well-calibrated. For maximum transparency, we report four different ratios (1.00 or 0.88 or 1.09 or 0.82) based on the above four different ways to compute this ratio. We note that $deviation^2 = s.d.^2 + bias^2$, therefore ratio (2) is smaller than ratio (1), and ratio (4) is smaller than ratio (3). These results correspond to **Supplementary Figure 9**. In the **sub-tables E-F**, we report the same four ratios, but for the 16 individual diseases (0.98 or 0.89 or 0.99 or 0.81).

Supplementary Table 9. Simulation results with the true liability-scale heritability set to 0.06 (instead of 0.13).

The first column for **sub-tables A-D** lists the disease PheCodes that have the same true $h^2{}_{pleio}/h^2$ based on the specified genetic architecture. In the **sub-tables A-B**, we show the estimated $h^2{}_{pleio}/h^2$, true $h^2{}_{pleio}/h^2$ and their absolute bias for 5 different values both before bias correction and after bias correction. Estimated $h^2{}_{pleio}/h^2$ before bias correction shows a slight upward bias, but it is approximately unbiased after bias correction. For the zero-trait simulations, we observe no inflation of the type I error rate (one-sided type I error rate = 0; two-sided type I error rate = 0). These results correspond to **Supplementary Figure 10**. In the **sub-tables C-D**, we report the calibration of s.e. using four ratios: (1) (average jackknife s.e.) / (standard deviation); (2) (average jackknife s.e.) / ($\sqrt{average\ squared\ deviation}$); (3) (average jackknife s.e.^2) / (squared standard deviation); (4) (average jackknife s.e.^2) / (average squared deviation). Deviation is the difference between true $h^2{}_{pleio}/h^2$ and the average estimated $h^2{}_{pleio}/h^2$. The estimated standard errors of $h^2{}_{pleio}/h^2$ after bias correction were approximately well-calibrated. For maximum transparency, we report four different ratios (1.23 or 1.06 or 1.85 or 1.30) based on the above four different ways to compute this ratio. We note that $deviation^2 = s.d.^2 + bias^2$, therefore ratio (2) is smaller than ratio (1), and ratio (4) is smaller than ratio (3). These results correspond to **Supplementary Figure 11**. In the **sub-**

tables E-F, we report the same four ratios, but for the 16 individual diseases (1.12 or 1.04 or 1.47 or 1.26).

Supplementary Table 10. Simulation results with the prevalence set to 0.05 (instead of 0.1).

The first column for **sub-tables A-D** lists the disease PheCodes that have the same true $h^2{}_{pleio}/h^2$ based on the specified genetic architecture. In the **sub-tables A-B**, we show the estimated $h^2{}_{pleio}/h^2$, true $h^2{}_{pleio}/h^2$ and their absolute bias for 5 different values both before bias correction and after bias correction. Estimated $h^2{}_{pleio}/h^2$ before bias correction shows a slight upward bias, but it is approximately unbiased after bias correction. For the zero-trait simulations, we observe no inflation of the type I error rate (one-sided type I error rate = 0; two-sided type I error rate = 0). These results correspond to **Supplementary Figure 12**. In the **sub-tables C-D**, we report the calibration of s.e. using four ratios: (1) (average jackknife s.e.) / (standard deviation); (2) (average jackknife s.e.) / ($\sqrt{average\ squared\ deviation}$); (3) (average jackknife s.e.^2) / (squared standard deviation); (4) (average jackknife s.e.^2) / (average squared deviation). Deviation is the difference between true $h^2{}_{pleio}/h^2$ and the average estimated $h^2{}_{pleio}/h^2$. The estimated standard errors of $h^2{}_{pleio}/h^2$ after bias correction were approximately well-calibrated. For maximum transparency, we report four different ratios (1.14 or 1.02 or 1.48 or 1.11) based on the above four different ways to compute this ratio. We note that $deviation^2 = s.d.^2 + bias^2$, therefore ratio (2) is smaller than ratio (1), and ratio (4) is smaller than ratio (3). These results correspond to **Supplementary Figure 13**. In the **sub-tables E-F**, we report the same four ratios, but for the 16 individual diseases (1.09 or 1.06 or 1.29 or 1.18).

Supplementary Table 11. Simulation results with the proportion of causal SNPs set to 1% (instead of 5%).

The first column for **sub-tables A-D** lists the disease PheCodes that have the same true $h^2{}_{pleio}/h^2$ based on the specified genetic architecture. In the **sub-tables A-B**, we show the estimated $h^2{}_{pleio}/h^2$, true $h^2{}_{pleio}/h^2$ and their absolute bias for 5 different values both before bias correction and after bias correction. Estimated $h^2{}_{pleio}/h^2$ before bias correction shows a slight upward bias, but it is approximately unbiased after bias correction. For the zero-trait simulations, we observe no inflation of the type I error rate (one-sided type I error rate = 0.01; two-sided type I error rate = 0). These results correspond to **Supplementary Figure 14**. In the **sub-tables C-D**, we report the calibration of s.e. using four ratios: (1) (average jackknife s.e.) / (standard deviation); (2) (average jackknife s.e.) / ($\sqrt{average\ squared\ deviation}$); (3) (average jackknife s.e.^2) / (squared standard deviation); (4) (average jackknife s.e.^2) / (average squared deviation). Deviation is the difference between true $h^2{}_{pleio}/h^2$ and the average estimated $h^2{}_{pleio}/h^2$. The estimated standard errors of $h^2{}_{pleio}/h^2$ after bias correction were approximately well-calibrated. For maximum transparency, we report four different ratios

(0.99 or 0.91 or 1.11 or 0.88) based on the above four different ways to compute this ratio. We note that $deviation^2 = s.d.^2 + bias^2$, therefore ratio (2) is smaller than ratio (1), and ratio (4) is smaller than ratio (3). These results correspond to **Supplementary Figure 15**. In the **sub-tables E-F**, we report the same four ratios, but for the 16 individual diseases (0.95 or 0.92 or 0.95 or 0.87)**.**

Supplementary Table 12. Simulation results when r_g within-disease categories equals to 0.5 and r_g between-disease categories equals to 0.1, without pruning procedure.

The first column for **sub-tables A-D** lists the disease PheCodes that have the same true ${h^2}_{pleio}/h^2$ based on the specified genetic architecture. In the **sub-tables A-B**, we show the estimated ${h^2}_{pleio}/h^2$, true ${h^2}_{pleio}/h^2$ and their absolute bias for 5 different values both before bias correction and after bias correction. Estimated ${h^2}_{pleio}/h^2$ before bias correction shows a slight upward bias, but it is approximately unbiased after bias correction. For the zero-trait simulations, we observe no inflation of the type I error rate (one-sided type I error rate = 0; two-sided type I error rate = 0). These results correspond to **Supplementary Figure 16**. In the **sub-tables C-D**, we report the calibration of s.e. using four ratios: (1) (average jackknife s.e.) / (standard deviation); (2) (average jackknife s.e.) / ($\sqrt{average\ squared\ deviation}$); (3) (average jackknife s.e.^2) / (squared standard deviation); (4) (average jackknife s.e.^2) / (average squared deviation). Deviation is the difference between true ${h^2}_{pleio}/h^2$ and the average estimated ${h^2}_{pleio}/h^2$. The estimated standard errors of ${h^2}_{pleio}/h^2$ after bias correction were approximately well-calibrated. For maximum transparency, we report four different ratios (1.08 or 1.01 or 1.25 or 1.05) based on the above four different ways to compute this ratio. We note that $deviation^2 = s.d.^2 + bias^2$, therefore ratio (2) is smaller than ratio (1), and ratio (4) is smaller than ratio (3). These results correspond to **Supplementary Figure 17**. In the **sub-tables E-F**, we report the same four ratios, but for the 16 individual diseases (0.99 or 0.97 or 1.02 or 0.96).

Supplementary Table 13. Simulations results of the reduction in ${\boldsymbol{h}^2}_{\boldsymbol{pleio}}/\boldsymbol{h}^2$ and its standard error in analyses with one auxiliary disease category removed.

In each sub-table, we report the reduction of estimated ${h^2}_{pleio}/h^2$, its standard error across simulations, true reduction of ${h^2}_{pleio}/h^2$, average jackknife standard error, standard deviation across simulation estimates, $\sqrt{average\ squared\ deviation}$ across simulations, average squared jackknife standard error, squared standard deviation simulation estimates, and average squared deviation across simulations, when removing each of the 7 auxiliary categories and zero-trait-category from the 15 diseases. Deviation is the difference between true reduction of ${h^2}_{pleio}/h^2$ and the before-bias-correction estimated reduction of ${h^2}_{pleio}/h^2$. We average results for diseases that have the same true reduction of ${h^2}_{pleio}/h^2$. These results correspond to **Supplementary Figures 18-19**.

Supplementary Table 14. Genetic correlations between 15 UK Biobank diseases.

We report the genetic correlations and their corresponding categories. These results correspond to **Figure 3A**.

Supplementary Table 15. Genetic correlations and liability correlations between 15 UK Biobank diseases.

We reported the genetic correlations, liability correlations, and their corresponding standard errors. We computed the z value using $\frac{r_g - r_l}{\sqrt{r_{g_{se}}^2 + r_{l_{se}}^2}}$, and then compute the right tailed p value. A Bonferroni-corrected significance threshold of $P < 4.76 \times 10^{-4}$(0.05/105) is applied. We also tested the $\frac{r_l - r_g}{\sqrt{r_{g_{se}}^2 + r_{l_{se}}^2}}$, and found that there is no pairs with liability correlation significantly larger than genetic correlation. These results correspond to **Figure 3**.

Supplementary Table 16. Genetic correlations across all 15 UK Biobank diseases, 30 non-UK Biobank diseases, 17 UK Biobank quantitative traits.

These results correspond to **Figure 3A** and **Supplementary Figure 24**.

Supplementary Table 17. $h^2{}_{pleio}/h^2$ before and after bias correction for all 15 UK Biobank diseases and the average with respect to 4 types of auxiliary diseases/traits.

D+Q: $h^2{}_{pleio}/h^2$ estimates with respect to 14 UK Biobank auxiliary diseases and 17 UK Biobank quantitative auxiliary traits. D: $h^2{}_{pleio}/h^2$ estimates with respect to 14 UK Biobank auxiliary diseases. D\target: $h^2{}_{pleio}/h^2$ estimates with respect to 14 UK Biobank auxiliary diseases excluding those from the same disease category as the target disease. D\(target+best other): $h^2{}_{pleio}/h^2$ estimates with respect to 14 UK Biobank auxiliary diseases excluding those from the same disease category as the target disease and from the next disease category whose removal had the greatest impact. We reported disease PheCode, pre-correction and post-correction $h^2{}_{pleio}/h^2$ and its standard error, scaling coefficient ξ_c in bias correction, selected target and auxiliary diseases/traits. These results correspond to **Figure 4**, **Extended Data Fig. 1** and **Extended Data Fig. 2**.

Supplementary Table 18. Differences between auxiliary diseases choices for average estimates in Figure 4 and Figure 6.

In **Figure 4**, we compared average estimates from D+Q, D\target and D\(target+best other) to estimates from D. To compare estimates of different auxiliary trait selection, we computed the jackknife standard error of the before-bias-correction difference which is an approximately well-calibrated standard error shown by simulations (See Analyses with one or more disease categories excluded from the set of auxiliary diseases section in the Methods section (pp. 23) and **Supplementary Figures 18-19**). We reported the p value of these comparisons 1) using flat average; 2) using inverse-variance weighting average by weighting each disease (weightings were computed using the D group and applied to all four groups). In **Figure 6A** and **6B**, we compared average estimates from 30 non-UK Biobank auxiliary diseases, all 45 auxiliary diseases, and all 62 auxiliary diseases and quantitative traits to estimates from 15 UK Biobank auxiliary diseases. We reported p values of these comparisons 1) using flat average; 2) using inverse-variance weighting average by weighting each disease based on its $\frac{1}{s.e.^2}$ from the 15 UK Biobank auxiliary diseases group and applied them to all auxiliary trait choices.

Supplementary Table 19. $\boldsymbol{h^2}_{\boldsymbol{pleio}}/\boldsymbol{h^2}$ estimates using the single auxiliary category and the reduction in $\boldsymbol{h^2}_{\boldsymbol{pleio}}/\boldsymbol{h^2}$ when one auxiliary disease category removed for all 15 UK Biobank target diseases.

In each sub-table, we report the $h^2{}_{pleio}/h^2$ estimates using the single auxiliary category, reduction of $h^2{}_{pleio}/h^2$ when one auxiliary disease category removed and their standard errors for 15 UK Biobank diseases with respect to all 7 auxiliary categories, respectively. $h^2{}_{pleio}/h^2$ of single-auxiliary-category estimate are zero when this auxiliary category only has the target disease. The zero reduction of $h^2{}_{pleio}/h^2$ when removing the auxiliary category indicates that the estimation of $h^2{}_{pleio}/h^2$ for all auxiliary diseases has already excluded those auxiliary diseases in the removed auxiliary category after the pruning procedure. Therefore, further removal of this auxiliary category makes no difference. These results correspond to **Figure 5** and **Supplementary Figure 20**.

Supplementary Table 20. Comparison of r_g from BOLT-REML and r_g from cross-trait LDSC with 4 options of constraining intercept.

We reported five estimates of r_g in following order: r_g from BOLT-REML, r_g from cross-trait LDSC without constraint on intercept, r_g from cross-trait LDSC constraining heritability intercept, r_g from cross-trait LDSC constraining genetic covariance intercept, r_g from cross-trait LDSC constraining both intercepts. These results correspond to **Supplementary Figure 22**.

Supplementary Table 21. Comparison of $\boldsymbol{h^2}_{\boldsymbol{pleio}}/\boldsymbol{h^2}$ estimated from BOLT-REML r_g and $\boldsymbol{h^2}_{\boldsymbol{pleio}}/\boldsymbol{h^2}$ estimated from cross-trait LDSC r_g with 4 options of constraining intercept.

We reported five estimates in following order: $h^2{}_{pleio}/h^2$ from BOLT-REML r_g, $h^2{}_{pleio}/h^2$ estimated from cross-trait LDSC r_g without constraint on intercept, $h^2{}_{pleio}/h^2$ estimated from cross-trait LDSC r_g constraining heritability intercept, $h^2{}_{pleio}/h^2$ estimated from cross-trait LDSC r_g constraining genetic covariance intercept, $h^2{}_{pleio}/h^2$ estimated from cross-trait LDSC r_g constraining both intercepts. These results correspond to **Supplementary Figure 23**.

Supplementary Table 22. $\boldsymbol{h^2}_{\boldsymbol{pleio}}/\boldsymbol{h^2}$ before and after bias correction for all 15 UK Biobank diseases and the average with respect to four types of auxiliary disease sets: 15 UK Biobank auxiliary diseases, 30 non-UK Biobank auxiliary diseases, all 45 auxiliary diseases, and all 62 auxiliary diseases and quantitative traits.

We reported disease PheCode, pre-correction and post-correction $h^2{}_{pleio}/h^2$ and its standard error, scaling coefficient ξ_c in bias correction, selected target and auxiliary diseases/traits. These results correspond to **Figure 6A** and **Extended Data Fig. 5**.

Supplementary Table 23. $\boldsymbol{h^2}_{\boldsymbol{pleio}}/\boldsymbol{h^2}$ before and after bias correction for all 30 non-UK Biobank diseases and the average with respect to four types of auxiliary disease sets: 15 UK Biobank auxiliary diseases, 30 non-UK Biobank auxiliary diseases, all 45 auxiliary diseases and all 62 auxiliary diseases and quantitative traits.

We reported disease PheCode, pre-correction and post-correction $h^2{}_{pleio}/h^2$ and its standard error, scaling coefficient ξ_c in bias correction, selected target and auxiliary diseases/traits. These results correspond to **Figure 6B** and **Extended Data Fig. 6**.

Supplementary Table 24. $\boldsymbol{h^2}_{\boldsymbol{pleio}}/\boldsymbol{h^2}$ estimates using the single auxiliary category and the reduction in $\boldsymbol{h^2}_{\boldsymbol{pleio}}/\boldsymbol{h^2}$ when one auxiliary disease category removed for all 45 UK Biobank target diseases + 30 non-UK Biobank target diseases.

In each sub-table, we reported the $h^2{}_{pleio}/h^2$ estimates using the single auxiliary category, reduction of $h^2{}_{pleio}/h^2$ when one auxiliary disease category removed and their standard errors for 45 UK Biobank diseases with respect to all 11 auxiliary categories, respectively. $h^2{}_{pleio}/h^2$ of single-auxiliary-category estimate are zero when this auxiliary category only has the target disease. The zero reduction of $h^2{}_{pleio}/h^2$ when removing the auxiliary category indicates that the estimation of $h^2{}_{pleio}/h^2$ for all auxiliary diseases has already excluded those auxiliary diseases in the removed auxiliary category after the pruning procedure. Therefore, further

removal of this auxiliary category makes no difference. These results correspond to **Supplementary Figure 25**.

Supplementary Table 25. Simulation results of $V^2{}_{pleio}/V^2$ and its standard error.

True r_o is set to 0.5 within diseases categories and 0.1 between diseases categories for the 15 diseases (based on the 7 Phecode disease categories), which implies 4 different values of true $V^2{}_{pleio}/V^2$ for each target disease (ranging from 0.05 to 0.38). We simulated liabilities for 228,258 individuals. We computed true r_l and true $V^2{}_{pleio}$ based on the simulated liabilities. Then, we used liability threshold model to generate binary phenotypes based on the empirical prevalence for these 15 UKB diseases. We applied our method on the simulated binary phenotypes to estimate $V^2{}_{pleio}$ and compared it to the true value. The first column for **sub-tables A-B** lists the disease PheCodes that have the same true $V^2{}_{pleio}/V^2$ based on the specified correlation architecture. In the **sub-table A**, we show the estimated $V^2{}_{pleio}/V^2$, true $V^2{}_{pleio}/V^2$ and their absolute bias for 4 different values before bias correction. Estimated $V^2{}_{pleio}/V^2$ is unbiased without the need for bias correction. These results correspond to **Extended Data Fig. 7**. In the **sub-table B**, we report the calibration of s.e. using four ratios: (1) (average jackknife s.e.) / (standard deviation); (2) (average jackknife s.e.) / (sqrt(average(deviation^2))); (3) (average jackknife s.e.^2) / (squared standard deviation); (4) (average jackknife s.e.^2) / (average deviation^2). Deviation is the difference between true $V^2{}_{pleio}/V^2$ and the average estimated $V^2{}_{pleio}/V^2$ across diseases that have the same true $V^2{}_{pleio}/V^2$. We estimate the standard error by jackknifing over blocks of individuals across all diseases. The estimated standard errors of $V^2{}_{pleio}/V^2$ are anti-conservative. For maximum transparency, we report calibration using above four ratios (1.15 or 0.75 or 1.38 or 0.65); we determined that this does not impact our results, as the standard errors of $V^2{}_{pleio}/V^2$ estimates are small given the large sample size of UK Biobank data. These results correspond to **Supplementary Figure 26**. In the **sub-table C**, we report the same four ratios, but for the 16 individual diseases.

Supplementary Table 26. Comparing $h^2{}_{pleio}/h^2$ $vs.$ $V^2{}_{pleio}/V^2$ $vs.$ $E^2{}_{pleio}/E^2$.

In the top sub-table, we report the post-correction $h^2{}_{pleio}/h^2$, pre-correction $V^2{}_{pleio}/V^2$, standard errors, z score for comparing their difference and two-tailed p value. We computed the s.e. of difference between $h^2{}_{pleio}/h^2$ and $V^2{}_{pleio}/V^2$ using $\sqrt{\left(s.e.of\,\frac{h^2{}_{pleio}}{h^2}\right)^2+\left(s.e.of\,\frac{V^2{}_{pleio}}{V^2}\right)^2}$. A Bonferroni-corrected significance threshold of $P < 3.33 \times 10^{-3}$(0.05/15) is applied. In the bottom sub-table, we report the post-correction $h^2{}_{pleio}$, pre-correction $V^2{}_{pleio}/V^2$, $E^2{}_{pleio}/E^2$, and their standard errors. We approximated the s.e of $h^2{}_{pleio}$ using $s.e.of\ h^2_{pleio}/h^2 \times h^2$ as the s.e. of h^2 is small relative to h^2_{pleio}. We approximated

the s.e of $E^2{}_{pleio}/E^2$ using $\frac{\sqrt{(s.e.of\ V^2{}_{pleio})^2+(s.e.of\ h^2{}_{pleio})^2}}{V^2-h^2}$ where we assumed that s.e. of E^2 is 0. These results correspond to **Figure 7** and **Supplementary Figures 27-30**.

Supplementary Table 27. The constrained and estimated intercepts for genetic covariance for each disease pair.

Computation of constrained intercepts for genetic covariance are described in **Methods** section. We compared empirical cross-trait intercept estimates from cross-trait LDSC (impacted by both sample overlap and population structure) to analytical cross-trait intercepts (impacted by sample overlap only) across 105 pairs of UK Biobank diseases. We found these to be concordant, with average empirical cross-trait intercept of 0.0461 (s.d. = 0.0464), average analytical intercept of 0.0520 (s.d. = 0.0522), and average difference of −0.0059 (s.d. = 0.0161), implying a limited impact of population structure and other confounders on products of z-scores for two traits.

Supplementary Table 28. Comparison of $h^2{}_{pleio}/h^2$ between estimate using only EA and reduction when removing the EA.

To investigate the impact of educational attainment (EA) on pleiotropy, we performed two analyses. First, we estimated $h^2{}_{pleio}/h^2$ with respect to EA as the only auxiliary trait for each of the 15 UK Biobank diseases and determined that EA had a substantial contribution, with an average of 9.9% (s.e. 0.8%). Second, we assessed the impact of removing EA from the set of auxiliary traits by estimating the difference between (i) $h^2{}_{pleio}/h^2$ with respect to 15 UK Biobank auxiliary diseases + 17 UK Biobank auxiliary quantitative traits vs. (ii) $h^2{}_{pleio}/h^2$ with respect to 15 UK Biobank auxiliary diseases + 16 UK Biobank quantitative traits excluding EA. We determined that differences were small, with an average reduction of 0.87% (s.e. 0.38%). These results correspond to **Extended Data Fig. 3**.

Supplementary Table 29. Changes of $h^2{}_{pleio}/h^2$ across 15 UK Biobank diseases for various ${r_g}^2$ thresholds between target and auxiliary diseases.

We performed analyses to test the $h^2{}_{pleio}/h^2$ for 15 UK Biobank diseases and the average for all ${r_g}^2$ thresholds between target and auxiliary diseases ranging from 0.1 to 0.8 in increments of 0.05. These results correspond to **Extended Data Fig. 4**.

Supplementary Table 30. Comparison between $h^2{}_{pleio}/h^2$ with the sum of (bias-corrected) ${r_g}^2$ across auxiliary diseases, for different target diseases and auxiliary disease sets.

For each of 15 UK Biobank target diseases, we considered 14 auxiliary disease sets (containing 1, …, 14 auxiliary diseases), defined by starting with the auxiliary disease with highest ${r_g}^2$ with the target disease and iteratively adding auxiliary diseases in order of decreasing ${r_g}^2$ with the target disease. We note that for some target disease, pruning will happen when adding auxiliary diseases one by one, so that the number of the final auxiliary disease set will decrease.

Supplementary Table 31. Correlation between $h^2{}_{pleio}/h^2$ and the sum of (bias-corrected and without-bias-corrected) ${r_g}^2$ across auxiliary diseases, across the 14 auxiliary disease sets.

The average correlation (across 15 target diseases) was 0.28 (0.30 when not applying bias correction to ${r_g}^2$), implying that $h^2{}_{pleio}/h^2$ captures different information than the sum of (bias-corrected) ${r_g}^2$. These results correspond to **Supplementary Tables 30**.

Supplementary Table 32. Single-trait LDSC intercept, GWAS average chi-square statistics and attenuation ratio of 15 UK biobank diseases.

We observed that the average single-trait intercept across 15 UK Biobank diseases was 1.02 (s.d. = 0.02), average chi-square statistics across 15 UK Biobank diseases was 1.16 (s.d. = 0.098), and average attenuation ratio (the ratio of inflation in intercept to inflation in chi-square statistics) is 0.11 (s.d. = 0.09), implying a limited impact of population structure and other confounders on GWAS chi-square statistics for a single trait.

Supplementary Figures

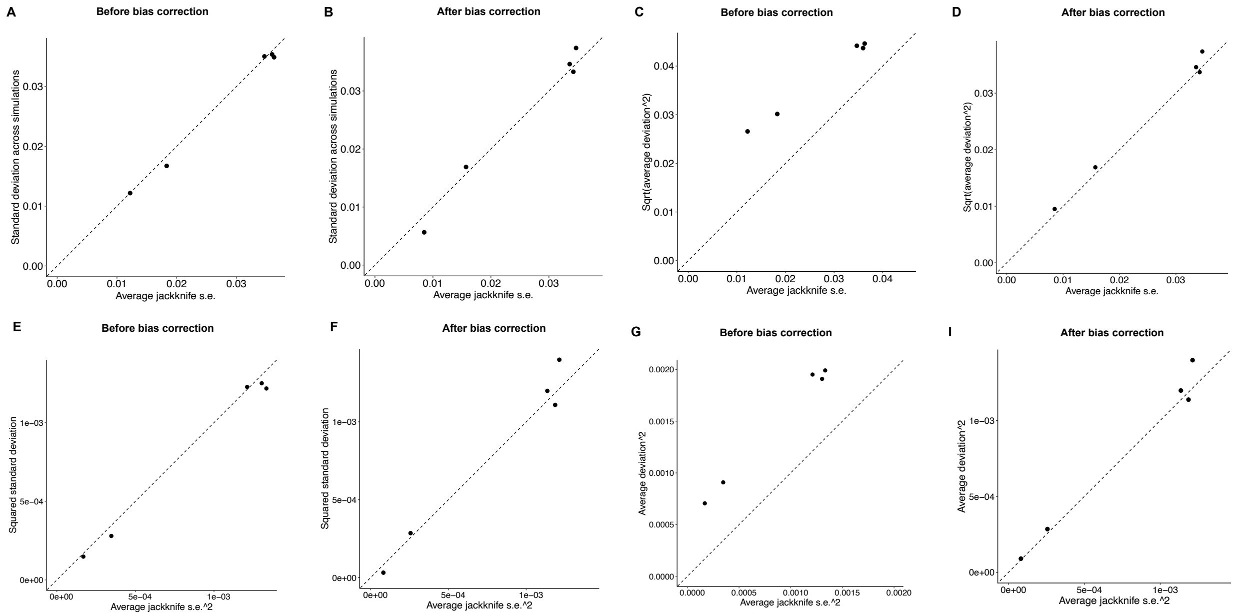

Supplementary Figure 1. Estimated standard errors of $h^2{}_{pleio}/h^2$ from PHBC were approximately well-calibrated.

Each point is the average of simulated diseases that have the same true values of $h^2{}_{pleio}/h^2$; in total, 16 simulated diseases have 5 distinct true values of $h^2{}_{pleio}/h^2$. We report calibration of s.e. using four ratios: (1) (average jackknife s.e.) / (standard deviation of point estimates across simulations); (2) (average jackknife s.e.) / ($\sqrt{average\ squared\ deviation}$); (3) (average jackknife s.e.^2) / (squared standard deviation of point estimates across simulations); (4) (average jackknife s.e.^2) / (average squared deviation). Deviation is the difference between true $h^2{}_{pleio}/h^2$ and the average estimated $h^2{}_{pleio}/h^2$. We note that $deviation^2 = s.d.^2 + bias^2$, therefore ratio (2) is smaller than ratio (1), and ratio (4) is smaller than ratio (3). (A-B) Average ratio (1) for both before-bias-correction estimates and post-bias-correction estimates. The average of the ratio across 5 categories is 1.07 for post-bias-correction estimates. (C-D) Average ratio (2) for both before-bias-correction estimates and post-bias-correction estimates. The average of the ratio across 5 categories is 0.95 for post-bias-correction estimates. (E-F) Average ratio (3) for both before-bias-correction estimates and post-bias-correction estimates. The average of the ratio across 5 categories is 1.27 for post-bias-correction estimates. (G-H) Average ratio (4) for both before-bias-correction estimates and post-bias-correction estimates. The average of the ratio across 5 categories is 0.93 for post-bias-correction estimates. Detailed results are provided in **Supplementary Table 4**.

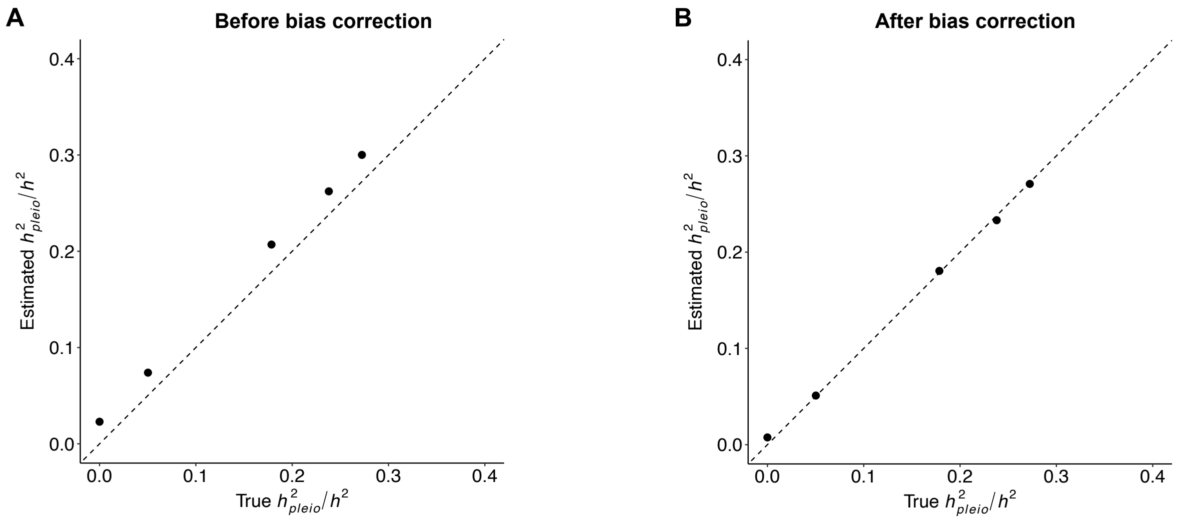

Supplementary Figure 2. Simulations with r_g within-disease categories equal to 0.4 (and r_g between-disease categories still equal to 0.1).

(A) In simulation with r_g within-disease categories equal to 0.4, estimated $h^2{}_{pleio}/h^2$ without the bias correction step shows an upward bias. (B) In simulation with r_g within-disease categories equal to 0.4, we observed that approximately unbiased results after Monte-Carlo bias correction. Each point and error bar in each panel represents the mean and standard error of diseases that has the same true $h^2{}_{pleio}/h^2$ across 100 simulations, in which error bars are smaller than point size in some cases. Detailed results are reported in **Supplementary Table 5**.

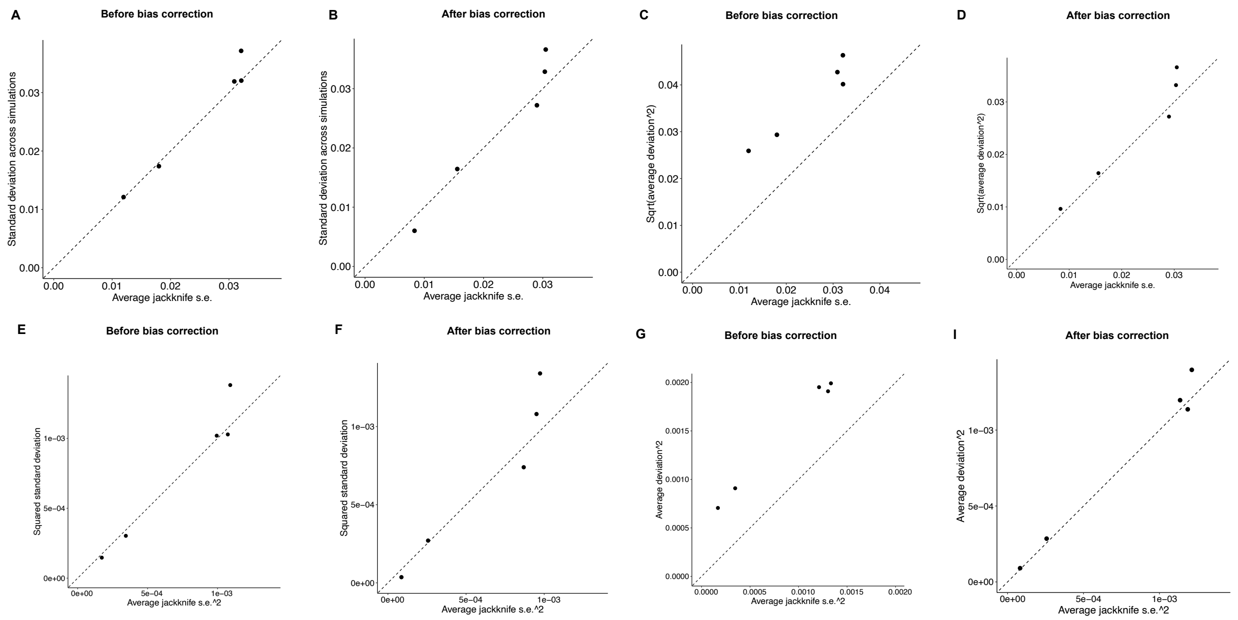

Supplementary Figure 3. Estimated standard errors of $h^2{}_{pleio}/h^2$ from PHBC were approximately well-calibrated in simulations with r_g within-disease categories equal to 0.4.

Each point is the average of simulated diseases that have the same true values of $h^2{}_{pleio}/h^2$; in total, 16 simulated diseases have 5 distinct true values of $h^2{}_{pleio}/h^2$. We report calibration of s.e. using four ratios: (1) (average jackknife s.e.) / (standard deviation of point estimates across simulations); (2) (average jackknife s.e.) / ($\sqrt{average\ squared\ deviation}$); (3) (average jackknife s.e.^2) / (squared standard deviation of point estimates across simulations); (4) (average jackknife s.e.^2) / (average squared deviation). Deviation is the difference between true $h^2{}_{pleio}/h^2$ and the average estimated $h^2{}_{pleio}/h^2$. We note that $deviation^2 = s.d.^2 + bias^2$, therefore ratio (2) is smaller than ratio (1), and ratio (4) is smaller than ratio (3). (A-B) Average ratio (1) for both before-bias-correction estimates and post-bias-correction estimates. The average of the ratio across 5 categories is 1.03 for post-bias-correction estimates. (C-D) Average ratio (2) for both before-bias-correction estimates and post-bias-correction estimates. The average of the ratio across 5 categories is 0.93 for post-bias-correction estimates. (E-F) Average ratio (3) for both before-bias-correction estimates and post-bias-correction estimates. The average of the ratio across 5 categories is 1.22 for post-bias-correction estimates. (G-H) Average ratio (4) for both before-bias-correction estimates and post-bias-correction estimates. The average of the ratio across 5 categories is 0.93 for post-bias-correction estimates. Detailed results are provided in **Supplementary Table 5**.

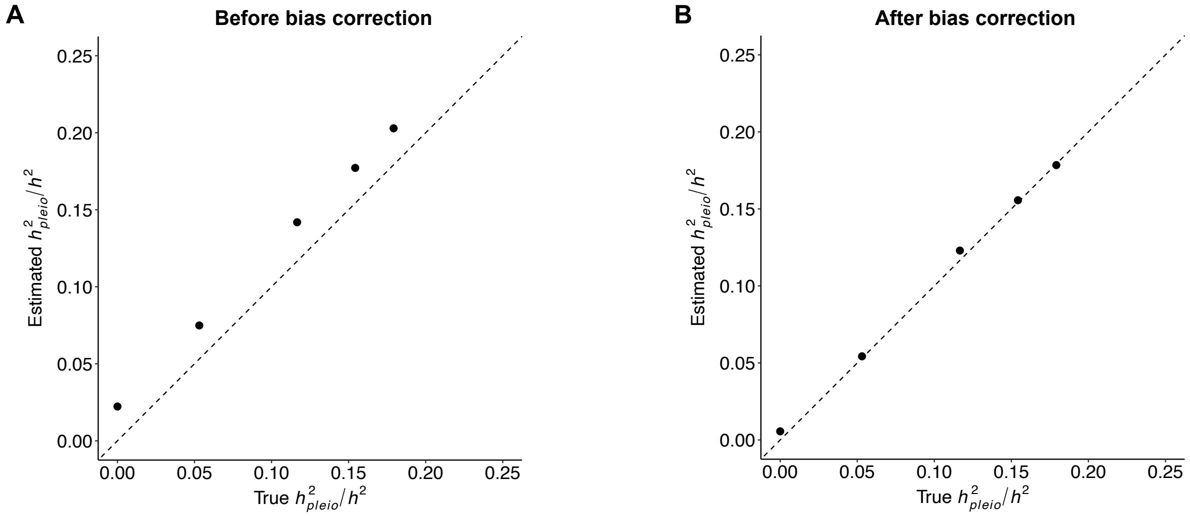

Supplementary Figure 4. Simulations with r_g within-disease categories equal to 0.3 (and r_g between-disease categories still equal to 0.1).

(A) In simulation with r_g within-disease categories equal to 0.3, estimated $h^2{}_{pleio}/h^2$ without the bias correction step shows an upward bias. (B) In simulation with r_g within-disease categories equal to 0.3, we observed that approximately unbiased results after Monte-Carlo bias correction. Each point and error bar in each panel represents the mean and standard error of diseases that has the same true $h^2{}_{pleio}/h^2$ across 100 simulations, in which error bars are smaller than point size in some cases. Detailed results are reported in **Supplementary Table 6**.

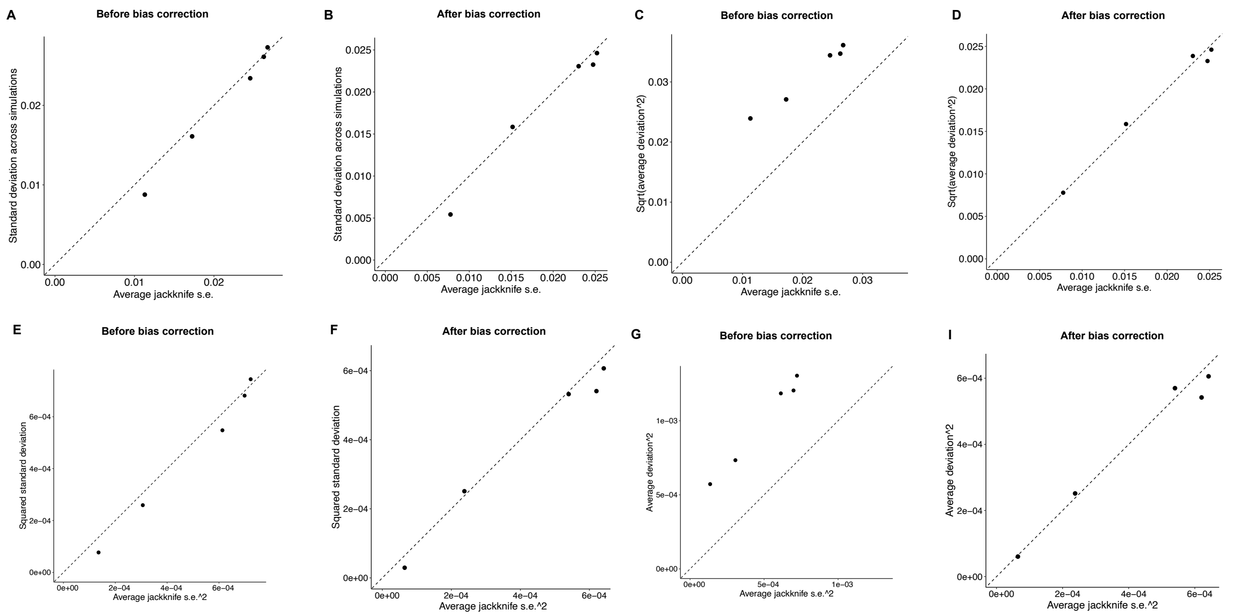

Supplementary Figure 5. Estimated standard errors of $h^2{}_{pleio}/h^2$ from PHBC were approximately well-calibrated in simulations with r_g within-disease categories equal to 0.3.

Each point is the average of simulated diseases that have the same true values of $h^2{}_{pleio}/h^2$; in total, 16 simulated diseases have 5 distinct true values of $h^2{}_{pleio}/h^2$. We report calibration of s.e. using four ratios: (1) (average jackknife s.e.) / (standard deviation of point estimates across simulations); (2) (average jackknife s.e.) / ($\sqrt{average\ squared\ deviation}$); (3) (average jackknife s.e.^2) / (squared standard deviation of point estimates across simulations); (4) (average jackknife s.e.^2) / (average squared deviation). Deviation is the difference between true $h^2{}_{pleio}/h^2$ and the average estimated $h^2{}_{pleio}/h^2$. We note that $deviation^2 = s.d.^2 + bias^2$, therefore ratio (2) is smaller than ratio (1), and ratio (4) is smaller than ratio (3). (A-B) Average ratio (1) for both before-bias-correction estimates and post-bias-correction estimates. The average of the ratio across 5 categories is 1.10 for post-bias-correction estimates. (C-D) Average ratio (2) for both before-bias-correction estimates and post-bias-correction estimates. The average of the ratio across 5 categories is 1.00 for post-bias-correction estimates. (E-F) Average ratio (3) for both before-bias-correction estimates and post-bias-correction estimates. The average of the ratio across 5 categories is 1.27 for post-bias-correction estimates. (G-H) Average ratio (4) for both before-bias-correction estimates and post-bias-correction estimates. The average of the ratio across 5 categories is 1.03 for post-bias-correction estimates. Detailed results are provided in **Supplementary Table 6**.

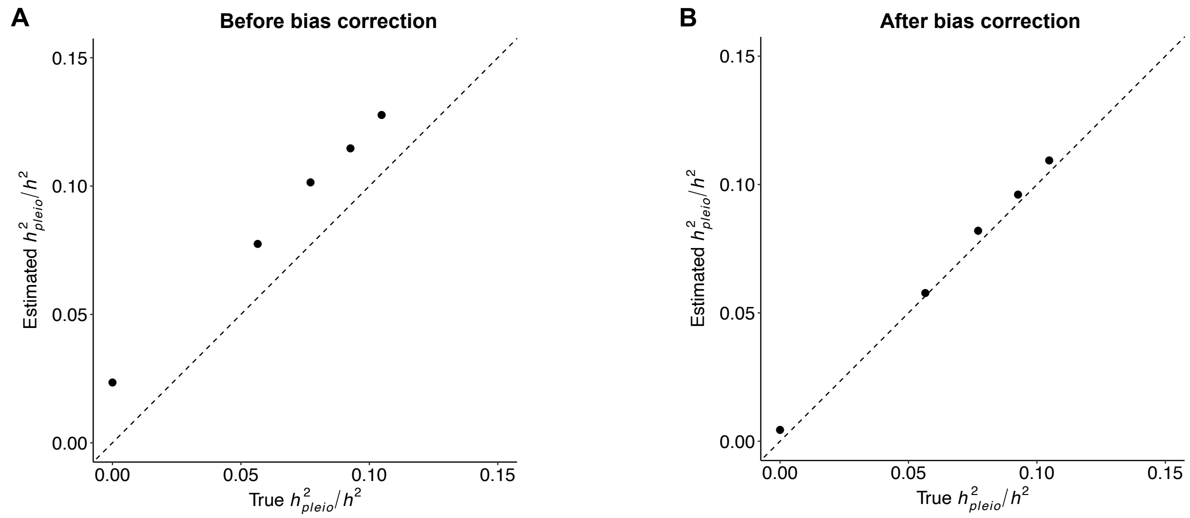

Supplementary Figure 6. Simulations with r_g within-disease categories equal to 0.2 (and r_g between-disease categories still equal to 0.1).

(A) In simulation with r_g within-disease categories equal to 0.2, estimated $h^2{}_{pleio}/h^2$ without the bias correction step shows an upward bias. (B) In simulation with r_g within-disease categories equal to 0.2, we observed that approximately unbiased results after Monte-Carlo bias correction. Each point and error bar in each panel represents the mean and standard error of diseases that has the same true $h^2{}_{pleio}/h^2$ across 100 simulations, in which error bars are smaller than point size in some cases. Detailed results are reported in **Supplementary Table 7**.

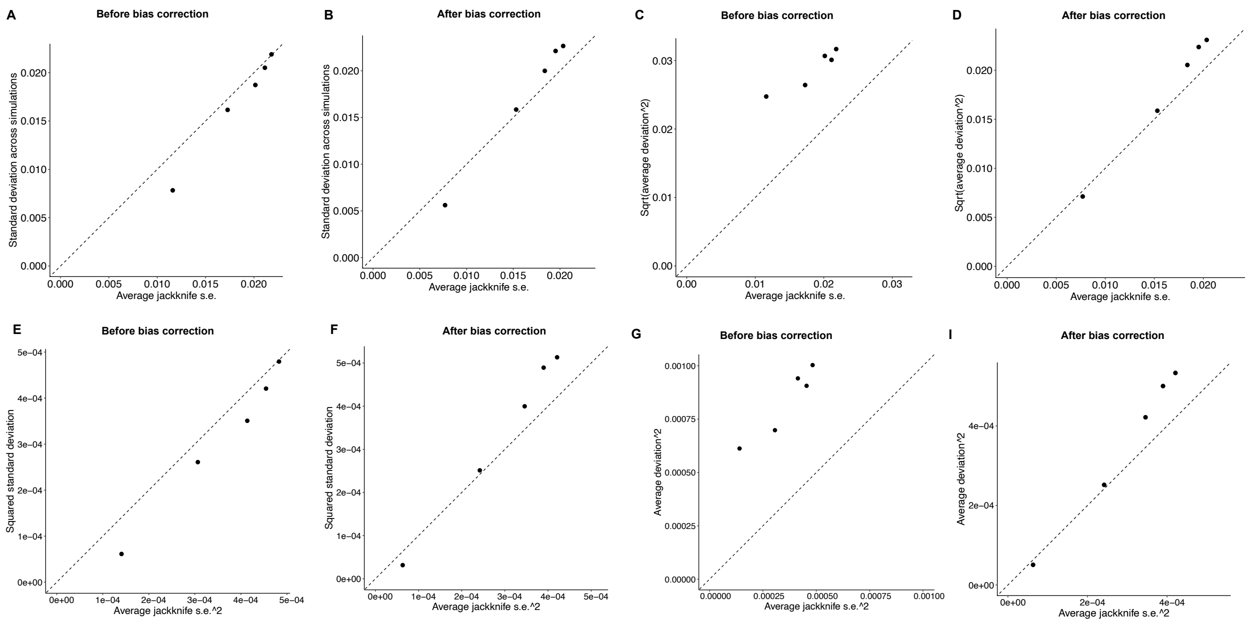

Supplementary Figure 7. Estimated standard errors of $h^2{}_{pleio}/h^2$ from PHBC were approximately well-calibrated in simulations with r_g within-disease categories equal to 0.2.

Each point is the average of simulated diseases that have the same true values of $h^2{}_{pleio}/h^2$; in total, 16 simulated diseases have 5 distinct true values of $h^2{}_{pleio}/h^2$. We report calibration of s.e. using four ratios: (1) (average jackknife s.e.) / (standard deviation of point estimates across simulations); (2) (average jackknife s.e.) / ($\sqrt{average\ squared\ deviation}$); (3) (average jackknife s.e.^2) / (squared standard deviation of point estimates across simulations); (4) (average jackknife s.e.^2) / (average squared deviation). Deviation is the difference between true $h^2{}_{pleio}/h^2$ and the average estimated $h^2{}_{pleio}/h^2$. We note that $deviation^2 = s.d.^2 + bias^2$, therefore ratio (2) is smaller than ratio (1), and ratio (4) is smaller than ratio (3). (A-B) Average ratio (1) for both before-bias-correction estimates and post-bias-correction estimates. The average of the ratio across 5 categories is 1.00 for post-bias-correction estimates. (C-D) Average ratio (2) for both before-bias-correction estimates and post-bias-correction estimates. The average of the ratio across 5 categories is 0.94 for post-bias-correction estimates. (E-F) Average ratio (3) for both before-bias-correction estimates and post-bias-correction estimates. The average of the ratio across 5 categories is 1.09 for post-bias-correction estimates. (G-H) Average ratio (4) for both before-bias-correction estimates and post-bias-correction estimates. The average of the ratio across 5 categories is 0.92 for post-bias-correction estimates. Detailed results are provided in **Supplementary Table 7**.

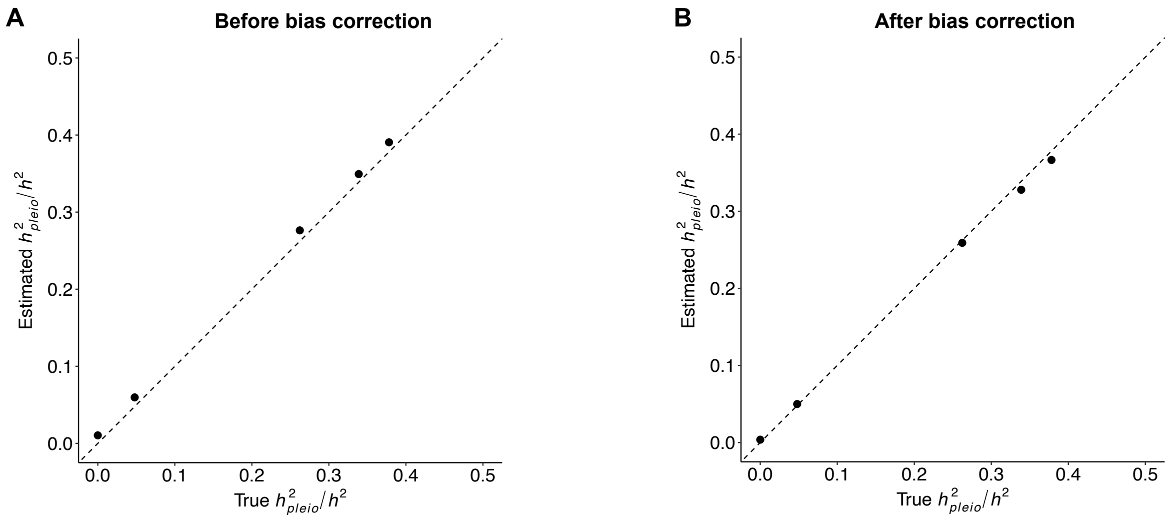

Supplementary Figure 8. PHBC corrects the upwards bias in simulations with the true liability-scale heritability set to 0.25 (instead of 0.13).

Estimated $h^2{}_{pleio}/h^2$ is approximately unbiased compared to the simulated truth, which is similar to **Figure 2**. (A) Estimated $h^2{}_{pleio}/h^2$ without the bias correction step shows an upward bias. (B) Estimated $h^2{}_{pleio}/h^2$ is approximated unbiased after the Monte-Carlo bias correction. Each point and error bar represents the mean and standard error of diseases that has the same true $h^2{}_{pleio}/h^2$ across 100 simulations, in which error bars are smaller than point size in some cases. Detailed results are reported in **Supplementary Table 8**.

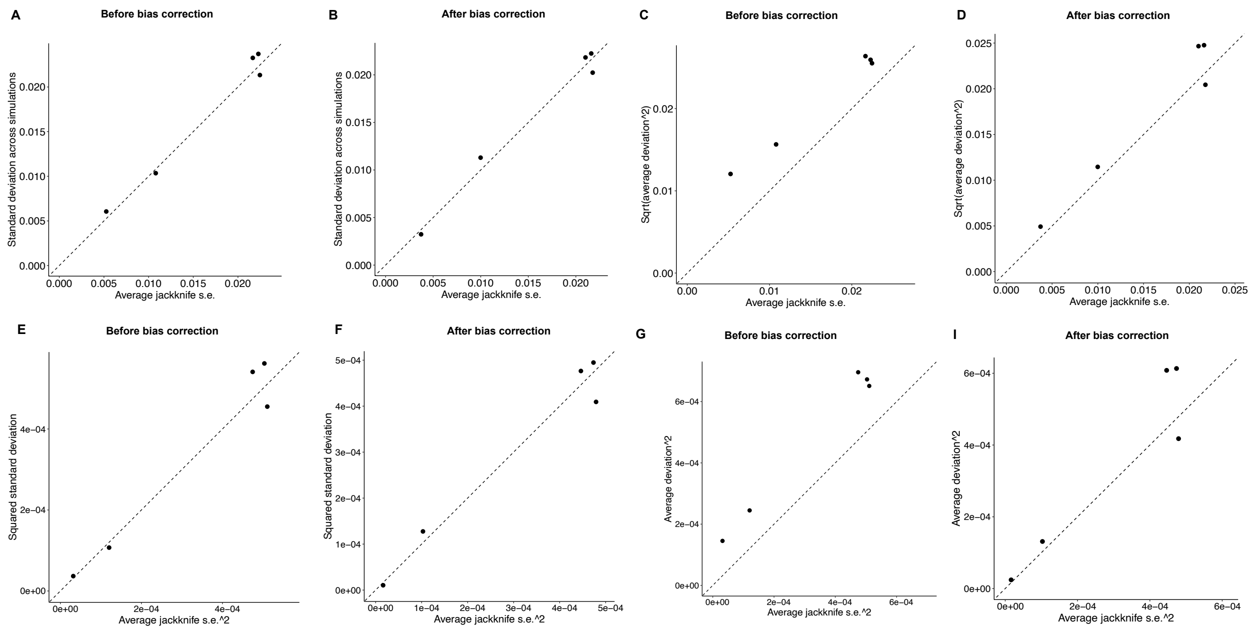

Supplementary Figure 9. Estimated standard errors of $h^2{}_{pleio}/h^2$ from PHBC were approximately well-calibrated in simulations with the true liability-scale heritability set to 0.25.

Each point is the average of simulated diseases that have the same true values of $h^2{}_{pleio}/h^2$; in total, 16 simulated diseases have 5 distinct true values of $h^2{}_{pleio}/h^2$. We report calibration of s.e. using four ratios: (1) (average jackknife s.e.) / (standard deviation of point estimates across simulations); (2) (average jackknife s.e.) / ($\sqrt{average\ squared\ deviation}$); (3) (average jackknife s.e.^2) / (squared standard deviation of point estimates across simulations); (4) (average jackknife s.e.^2) / (average squared deviation). Deviation is the difference between true $h^2{}_{pleio}/h^2$ and the average estimated $h^2{}_{pleio}/h^2$. We note that $deviation^2 = s.d.^2 + bias^2$, therefore ratio (2) is smaller than ratio (1), and ratio (4) is smaller than ratio (3). (A-B) Average ratio (1) for both before-bias-correction estimates and post-bias-correction estimates. The average of the ratio across 5 categories is 1.00 for post-bias-correction estimates. (C-D) Average ratio (2) for both before-bias-correction estimates and post-bias-correction estimates. The average of the ratio across 5 categories is 0.88 for post-bias-correction estimates. (E-F) Average ratio (3) for both before-bias-correction estimates and post-bias-correction estimates. The average of the ratio across 5 categories is 1.09 for post-bias-correction estimates. (G-H) Average ratio (4) for both before-bias-correction estimates and post-bias-correction estimates. The average of the ratio across 5 categories is 0.82 for post-bias-correction estimates. Detailed results are provided in **Supplementary Table 8**.

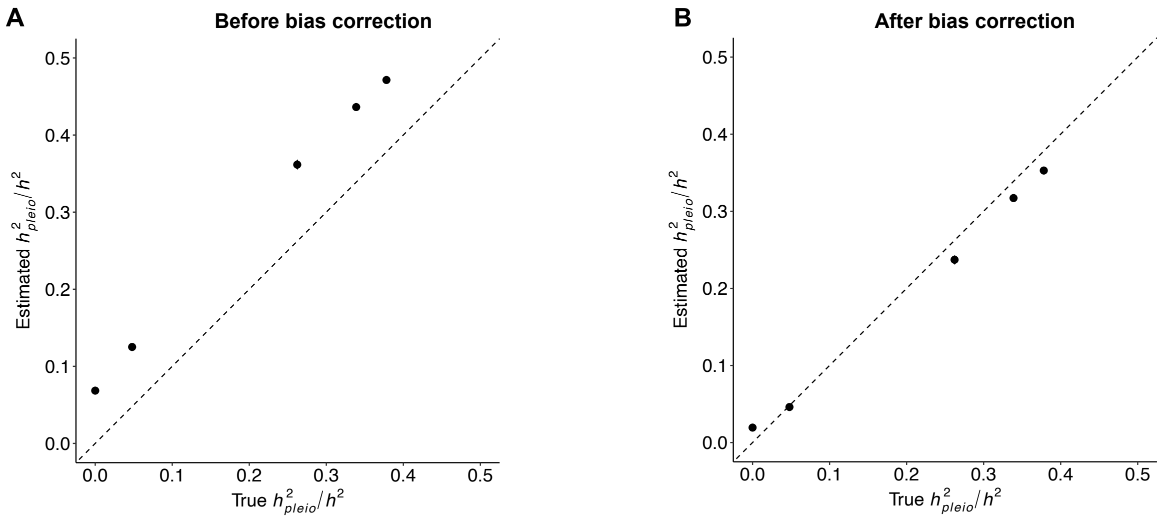

Supplementary Figure 10. PHBC corrects the upwards bias in simulations with the true liability-scale heritability set to 0.06 (instead of 0.13).

Estimated $h^2{}_{pleio}/h^2$ is approximately unbiased compared to the simulated truth, which is similar to **Figure 2**. (A) Estimated $h^2{}_{pleio}/h^2$ without the bias correction step shows an upward bias. (B) Estimated $h^2{}_{pleio}/h^2$ show modest downward bias for values above 25% and modest upward bias for values below 5% after the Monte-Carlo bias correction. Each point and error bar represents the mean and standard error of diseases that has the same true $h^2{}_{pleio}/h^2$ across 100 simulations, in which error bars are smaller than point size in some cases. The average simulated heritability z-score (average of 11.3 across simulated diseases) is similar to the average of 10.2 across 15 UK Biobank diseases. Detailed results are reported in **Supplementary Table 9**.

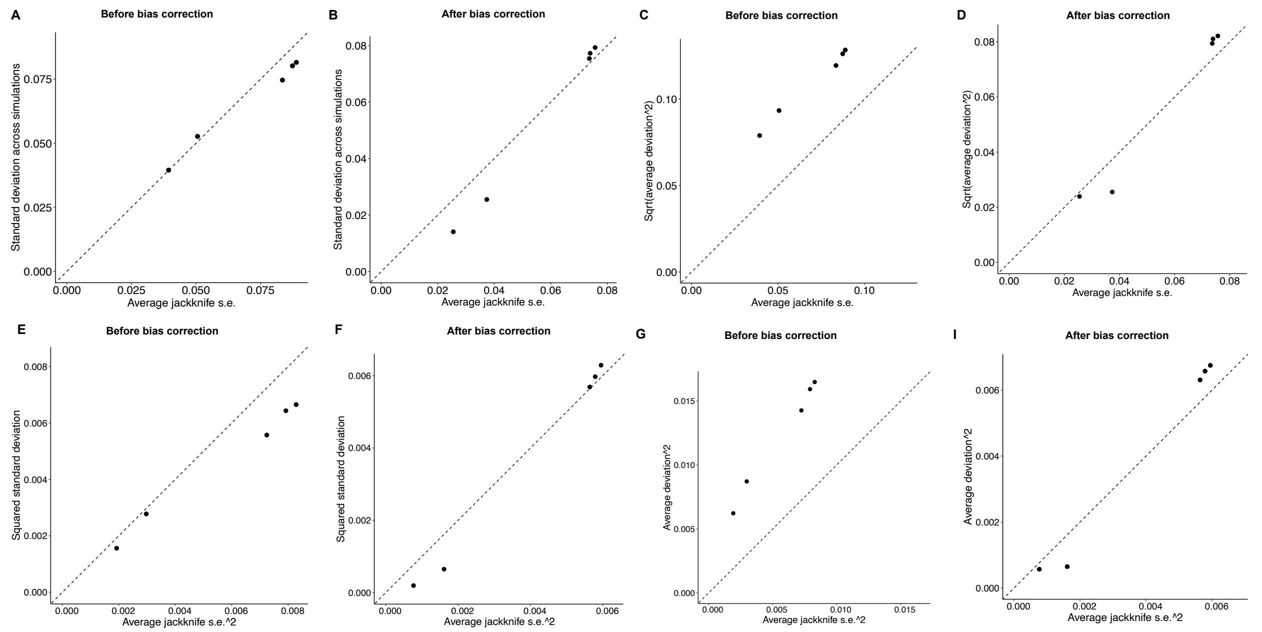

Supplementary Figure 11. Estimated standard errors of $h^2{}_{pleio}/h^2$ from PHBC were conservative in simulations with the true liability-scale heritability set to 0.06.

Each point is the average of simulated diseases that have the same true values of $h^2{}_{pleio}/h^2$; in total, 16 simulated diseases have 5 distinct true values of $h^2{}_{pleio}/h^2$. We report calibration of s.e. using four ratios: (1) (average jackknife s.e.) / (standard deviation of point estimates across simulations); (2) (average jackknife s.e.) / ($\sqrt{average\ squared\ deviation}$); (3) (average jackknife s.e.^2) / (squared standard deviation of point estimates across simulations); (4) (average jackknife s.e.^2) / (average squared deviation). Deviation is the difference between true $h^2{}_{pleio}/h^2$ and the average estimated $h^2{}_{pleio}/h^2$. We note that $deviation^2 = s.d.^2 + bias^2$, therefore ratio (2) is smaller than ratio (1), and ratio (4) is smaller than ratio (3). (A-B) Average ratio (1) for both before-bias-correction estimates and post-bias-correction estimates. The average of the ratio across 5 categories is 1.23 for post-bias-correction estimates. (C-D) Average ratio (2) for both before-bias-correction estimates and post-bias-correction estimates. The average of the ratio across 5 categories is 1.06 for post-bias-correction estimates. (E-F) Average ratio (3) for both before-bias-correction estimates and post-bias-correction estimates. The average of the ratio across 5 categories is 1.85 for post-bias-correction estimates. (G-H) Average ratio (4) for both before-bias-correction estimates and post-bias-correction estimates. The average of the ratio across 5 categories is 1.30 for post-bias-correction estimates. We note that our method produces conservative s.e. when true $h^2{}_{pleio}/h^2$ is small, which drives the overall conservative s.e. in panel (F). Detailed results are provided in **Supplementary Table 9**.

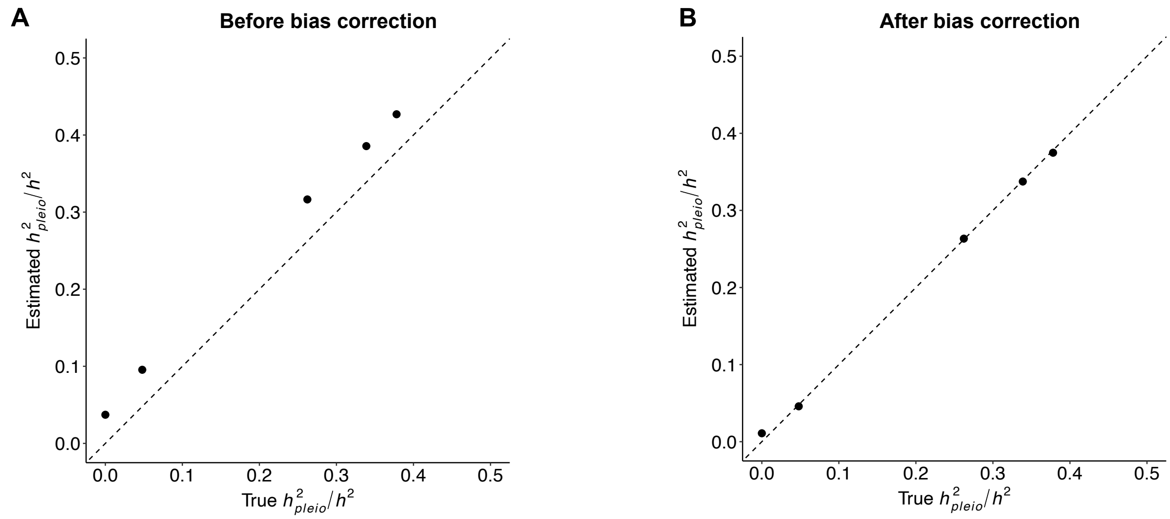

Supplementary Figure 12. PHBC corrects the upwards bias in simulations with the prevalence set to 0.05 (instead of 0.1).

Estimated $h^2{}_{pleio}/h^2$ is approximately unbiased compared to the simulated truth, which is similar to **Figure 2**. (A) Estimated $h^2{}_{pleio}/h^2$ without the bias correction step shows an upward bias. (B) Estimated $h^2{}_{pleio}/h^2$ is approximated unbiased after the Monte-Carlo bias correction. The average simulated heritability z-score (average of 9.6 across simulated diseases) is similar to the average of 10.2 across 15 UK Biobank diseases. Each point and error bar represents the mean and standard error of diseases that has the same true $h^2{}_{pleio}/h^2$ across 100 simulations, in which error bars are smaller than point size in some cases. Detailed results are reported in **Supplementary Table 10**.

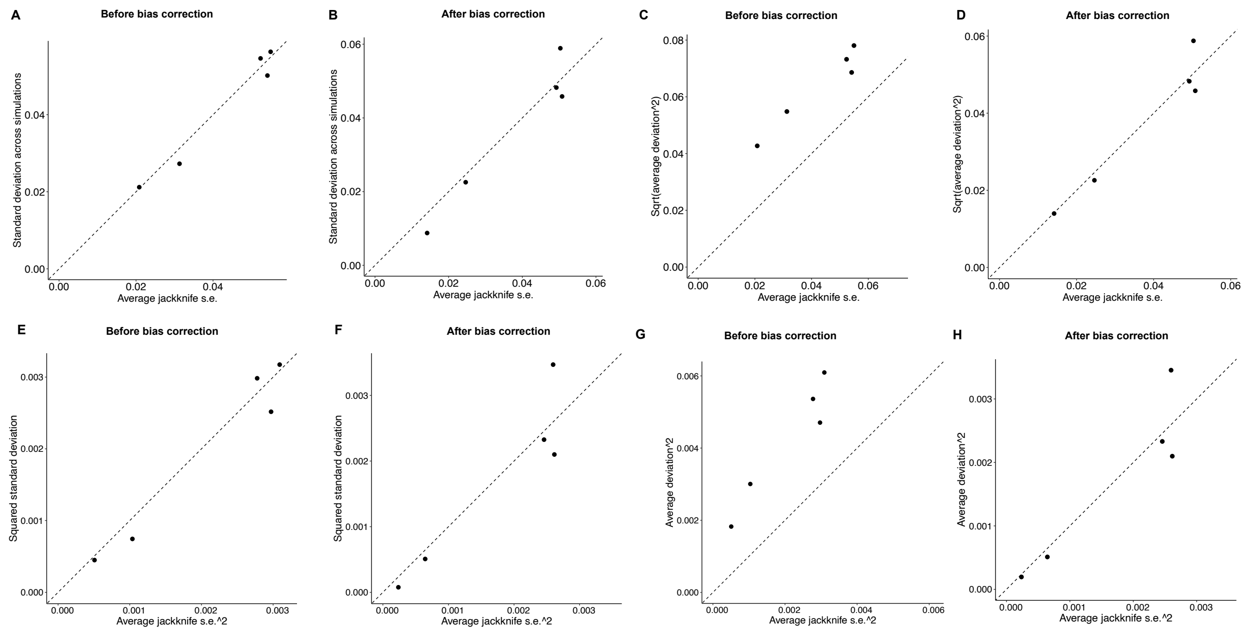

Supplementary Figure 13. Estimated standard errors of $h^2{}_{pleio}/h^2$ from PHBC were approximately well-calibrated in simulations with the prevalence set to 0.05.

Each point is the average of simulated diseases that have the same true values of $h^2{}_{pleio}/h^2$; in total, 16 simulated diseases have 5 distinct true values of $h^2{}_{pleio}/h^2$. We report calibration of s.e. using four ratios: (1) (average jackknife s.e.) / (standard deviation of point estimates across simulations); (2) (average jackknife s.e.) / ($\sqrt{average\ squared\ deviation}$); (3) (average jackknife s.e.^2) / (squared standard deviation of point estimates across simulations); (4) (average jackknife s.e.^2) / (average squared deviation). Deviation is the difference between true $h^2{}_{pleio}/h^2$ and the average estimated $h^2{}_{pleio}/h^2$. We note that $deviation^2 = s.d.^2 + bias^2$, therefore ratio (2) is smaller than ratio (1), and ratio (4) is smaller than ratio (3). (A-B) Average ratio (1) for both before-bias-correction estimates and post-bias-correction estimates. The average of the ratio across 5 categories is 1.14 for post-bias-correction estimates. (C-D) Average ratio (2) for both before-bias-correction estimates and post-bias-correction estimates. The average of the ratio across 5 categories is 1.02 for post-bias-correction estimates. (E-F) Average ratio (3) for both before-bias-correction estimates and post-bias-correction estimates. The average of the ratio across 5 categories is 1.48 for post-bias-correction estimates. (G-H) Average ratio (4) for both before-bias-correction estimates and post-bias-correction estimates. The average of the ratio across 5 categories is 1.11 for post-bias-correction estimates. Detailed results are provided in **Supplementary Table 10**.

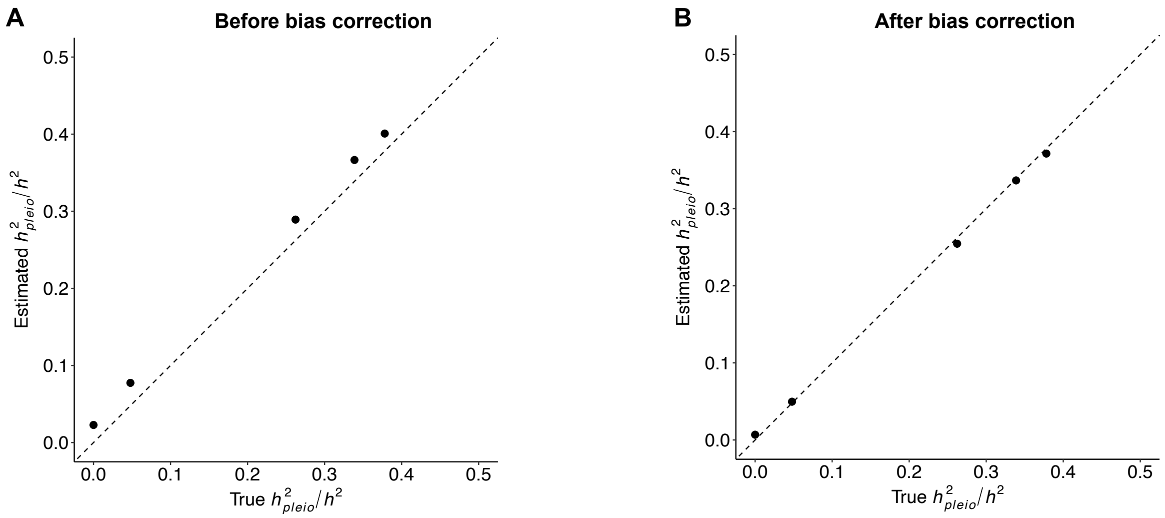

Supplementary Figure 14. PHBC corrects the upwards bias in simulations with the proportion of causal SNPs set to 1% (instead of 5%).

Estimated ${h^2}_{pleio}/h^2$ is approximately unbiased compared to the simulated truth, which is similar to **Figure 2**. (A) Estimated ${h^2}_{pleio}/h^2$ without the bias correction step shows an upward bias. (B) Estimated ${h^2}_{pleio}/h^2$ is approximated unbiased after the Monte-Carlo bias correction. Each point and error bar represents the mean and standard error of diseases that has the same true ${h^2}_{pleio}/h^2$ across 100 simulations, in which error bars are smaller than point size in some cases. Detailed results are reported in **Supplementary Table 11**.

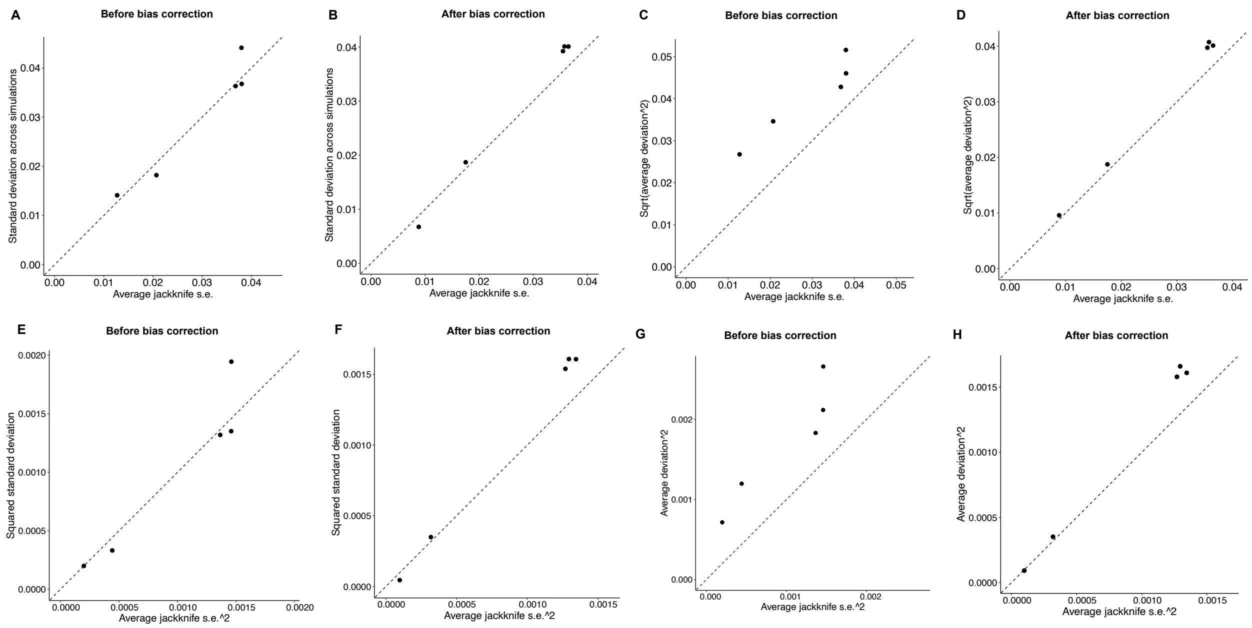

Supplementary Figure 15. Estimated standard errors of h^2_{pleio}/h^2 from PHBC were approximately well-calibrated in simulations with the proportion of causal SNPs set to 1%.

Each point is the average of simulated diseases that have the same true values of $h^2{}_{pleio}/h^2$; in total, 16 simulated diseases have 5 distinct true values of $h^2{}_{pleio}/h^2$. We report calibration of s.e. using four ratios: (1) (average jackknife s.e.) / (standard deviation of point estimates across simulations); (2) (average jackknife s.e.) / ($\sqrt{average\ squared\ deviation}$); (3) (average jackknife s.e.^2) / (squared standard deviation of point estimates across simulations); (4) (average jackknife s.e.^2) / (average squared deviation). Deviation is the difference between true $h^2{}_{pleio}/h^2$ and the average estimated $h^2{}_{pleio}/h^2$. We note that $deviation^2 = s.d.^2 + bias^2$, therefore ratio (2) is smaller than ratio (1), and ratio (4) is smaller than ratio (3). (A-B) Average ratio (1) for both before-bias-correction estimates and post-bias-correction estimates. The average of the ratio across 5 categories is 0.99 for post-bias-correction estimates. (C-D) Average ratio (2) for both before-bias-correction estimates and post-bias-correction estimates. The average of the ratio across 5 categories is 0.91 for post-bias-correction estimates. (E-F) Average ratio (3) for both before-bias-correction estimates and post-bias-correction estimates. The average of the ratio across 5 categories is 1.11 for post-bias-correction estimates. (G-H) Average ratio (4) for both before-bias-correction estimates and post-bias-correction estimates. The average of the ratio across 5 categories is 0.88 for post-bias-correction estimates. Detailed results are provided in **Supplementary Table 11**.

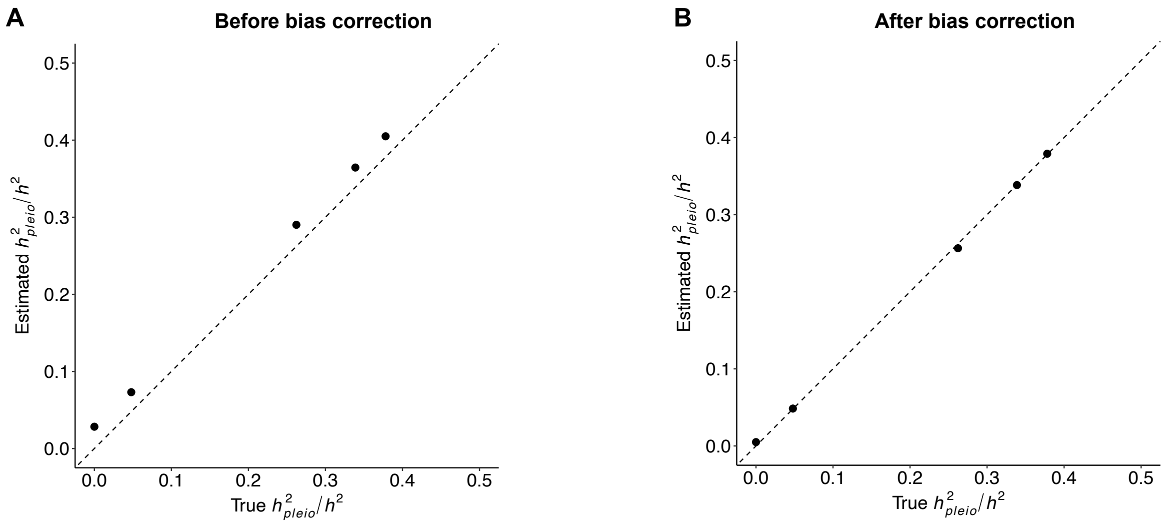

Supplementary Figure 16. PHBC corrects the upwards bias in simulations without pruning procedure.

Estimated ${h^2}_{pleio}/h^2$ is approximately unbiased compared to the simulated truth, which is similar to **Figure 2**. (A) Estimated ${h^2}_{pleio}/h^2$ without the bias correction step shows an upward bias. (B) Estimated ${h^2}_{pleio}/h^2$ is approximated unbiased after the Monte-Carlo bias correction. Each point and error bar represents the mean and standard error of diseases that has the same true ${h^2}_{pleio}/h^2$ across 100 simulations, in which error bars are smaller than point size in some cases. Detailed results are reported in **Supplementary Table 12**.

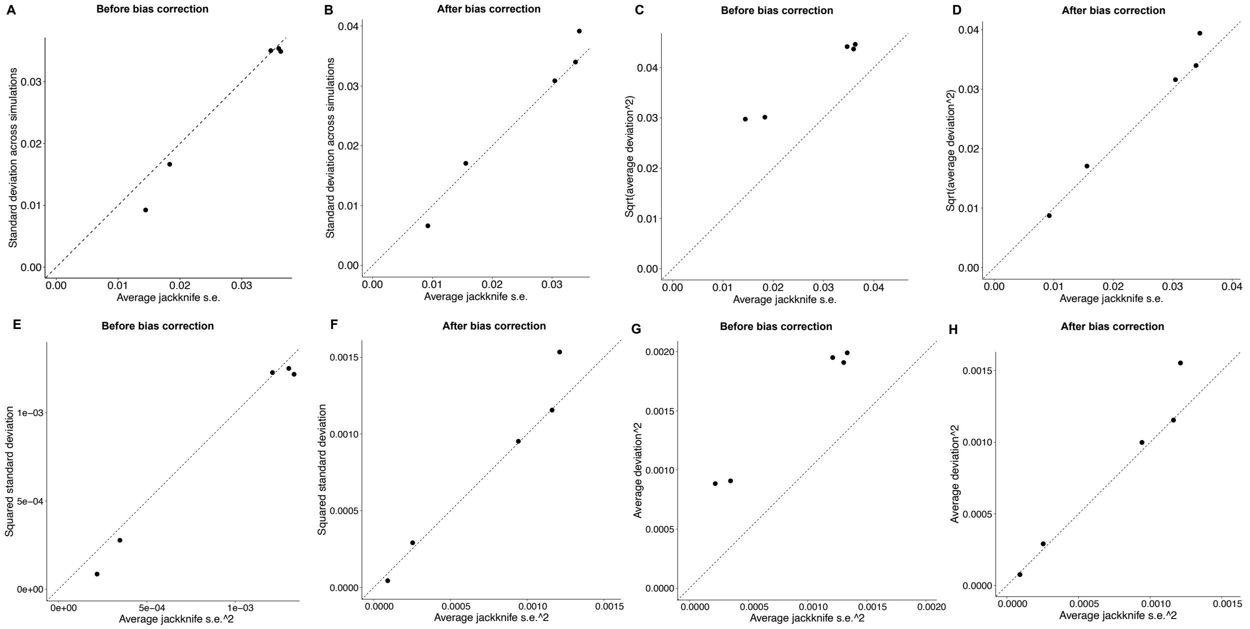

Supplementary Figure 17. Estimated standard errors of $h^2{}_{pleio}/h^2$ from PHBC were approximately well-calibrated in simulations without pruning procedure.

Each point is the average of simulated diseases that have the same true values of $h^2{}_{pleio}/h^2$; in total, 16 simulated diseases have 5 distinct true values of $h^2{}_{pleio}/h^2$. We report calibration of s.e. using four ratios: (1) (average jackknife s.e.) / (standard deviation of point estimates across simulations); (2) (average jackknife s.e.) / ($\sqrt{average\ squared\ deviation}$); (3) (average jackknife s.e.^2) / (squared standard deviation of point estimates across simulations); (4) (average jackknife s.e.^2) / (average squared deviation). Deviation is the difference between true $h^2{}_{pleio}/h^2$ and the average estimated $h^2{}_{pleio}/h^2$. We note that $deviation^2 = s.d.^2 + bias^2$, therefore ratio (2) is smaller than ratio (1), and ratio (4) is smaller than ratio (3). (A-B) Average ratio (1) for both before-bias-correction estimates and post-bias-correction estimates. The average of the ratio across 5 categories is 1.08 for post-bias-correction estimates. (C-D) Average ratio (2) for both before-bias-correction estimates and post-bias-correction estimates. The average of the ratio across 5 categories is 1.01 for post-bias-correction estimates. (E-F) Average ratio (3) for both before-bias-correction estimates and post-bias-correction estimates. The average of the ratio across 5 categories is 1.25 for post-bias-correction estimates. (G-H) Average ratio (4) for both before-bias-correction estimates and post-bias-correction estimates. The average of the ratio across 5 categories is 1.05 for post-bias-correction estimates. Detailed results are provided in **Supplementary Table 12**.

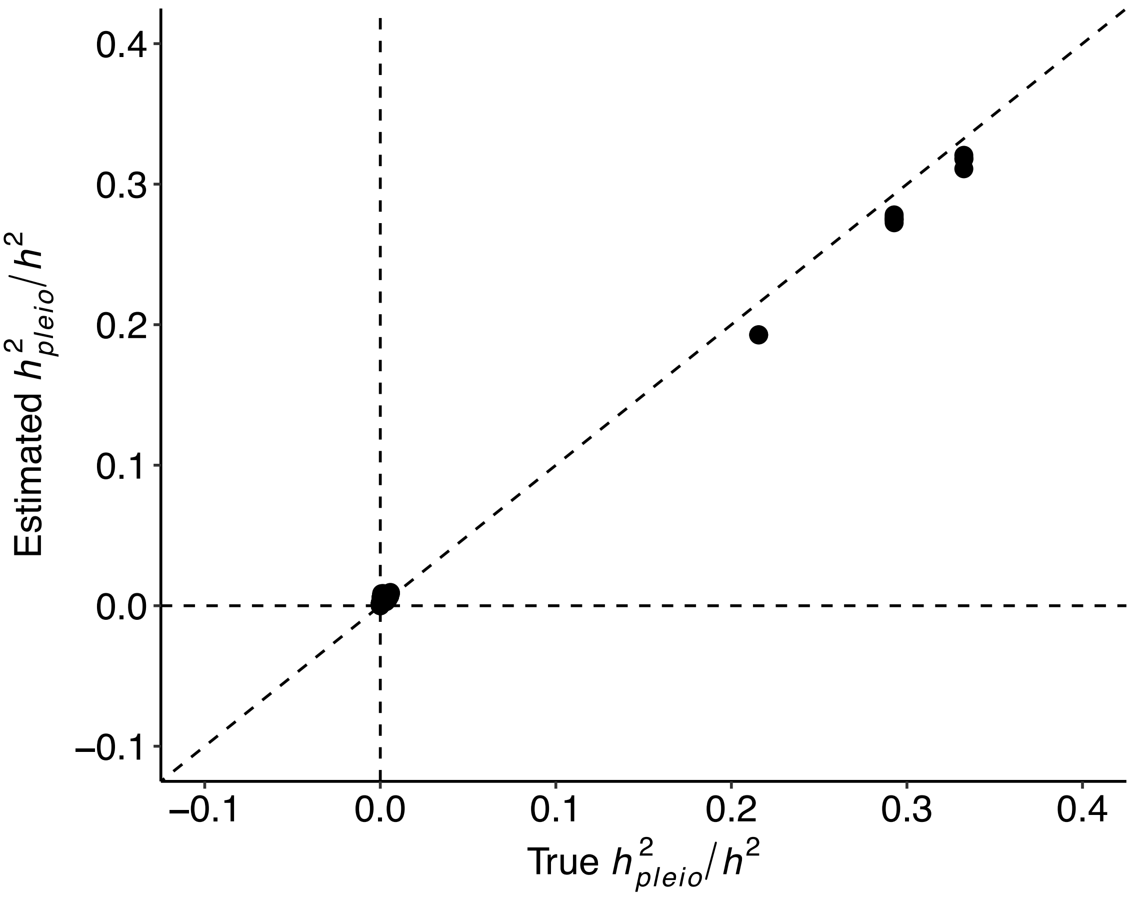

Supplementary Figure 18. Simulations of the reduction in $h^2{}_{pleio}/h^2$ in analyses with one auxiliary disease category removed.

To remove the impact of randomness in Monte-Carlo bias correction when computing the reduction, we first computed the difference of $h^2{}_{pleio}/h^2$ w.r.t. all auxiliary diseases vs. $h^2{}_{pleio}/h^2$ w.r.t. auxiliary diseases excluding a PheCode category before bias correction. We multiplied the difference by the scaling coefficient ξ_c^2 of $h^2{}_{pleio}/h^2$ w.r.t. all auxiliary diseases, which represented the contribution of an auxiliary PheCode category to the $h^2{}_{pleio}/h^2$ w.r.t. all auxiliary diseases. Each point and error bar represents the mean and standard error that has the same true reduction of $h^2{}_{pleio}/h^2$ across 100 simulations, in which error bars are smaller than point size in some cases. The estimated reduction of $h^2{}_{pleio}/h^2$ will have modest downward bias when true reduction is above 20% but noted that estimates in empirical data are usually very small (less than 10% in **Supplementary Tables 19** and **24**).

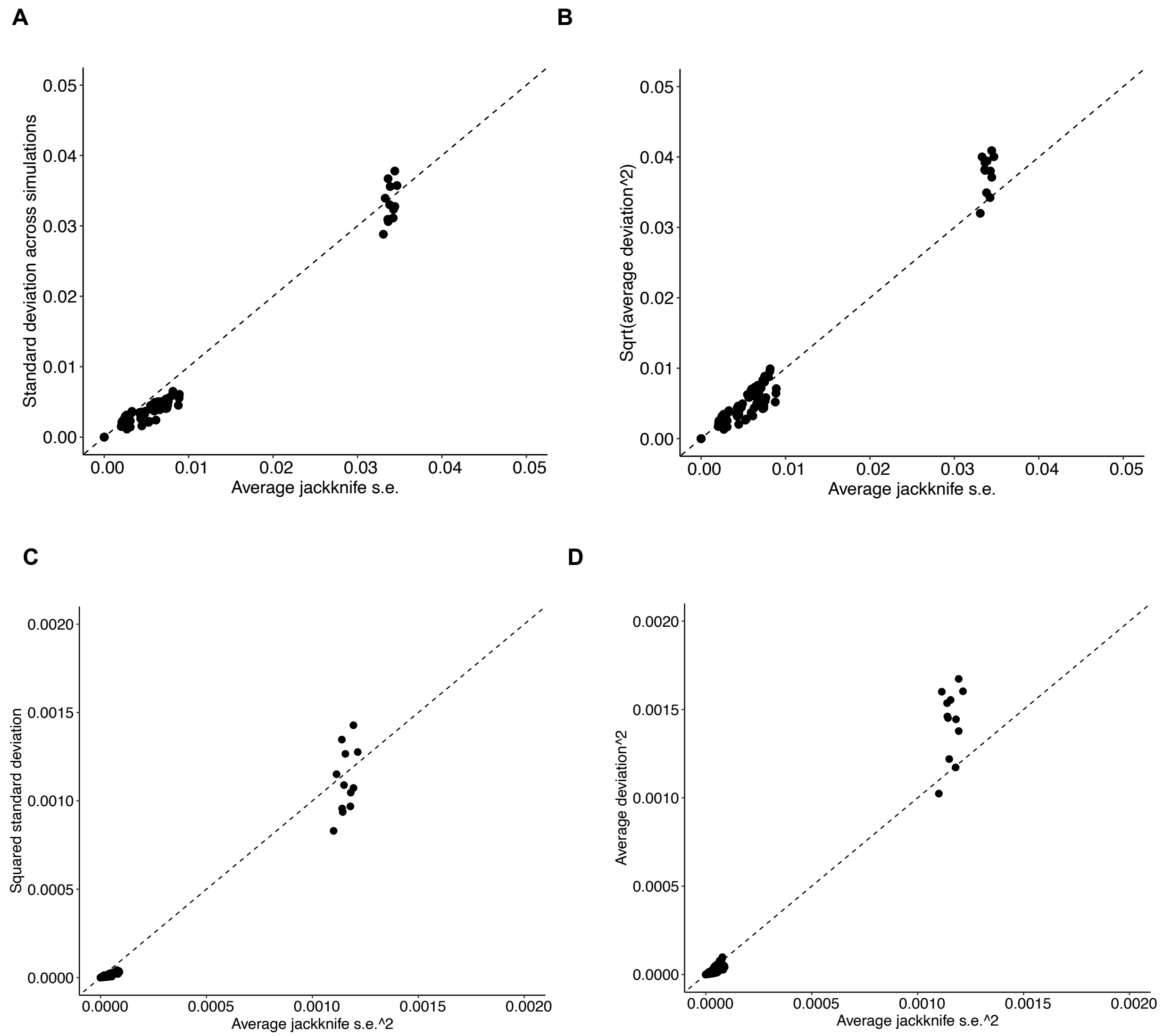

Supplementary Figure 19. Estimated standard errors of the reduction in $h^2{}_{pleio}/h^2$ when removing one auxiliary disease category were approximately well-calibrated.

Each point corresponds the change in $\boldsymbol{h^2{}_{pleio}/h^2}$ when removing one auxiliary category (totally 128 points (16 simulated diseases × 8 auxiliary categories)). We report calibration of s.e. using four ratios: (1) (average jackknife s.e.) / (standard deviation of point estimates across simulations); (2) (average jackknife s.e.) / ($\sqrt{average\ squared\ deviation}$); (3) (average jackknife s.e.^2) / (squared standard deviation of point estimates across simulations); (4) (average jackknife s.e.^2) / (average squared deviation). Deviation is the difference between true reduction of $h^2{}_{pleio}/h^2$ when removing auxiliary category and corresponding estimated reduction using before-bias-correction $h^2{}_{pleio}/h^2$. We reported the ratio of averages across the points, as this is a more robust estimate (compared to average of ratios) when many points have

small values. (A) Average jackknife s.e. vs. standard deviation of estimates across simulations. The ratio of average is 1.19. (B) Average jackknife s.e. vs. $\sqrt{average\ squared\ deviation}$. The ratio of average is 0.99. (C) Average squared jackknife s.e. vs. squared standard deviation of estimates across simulations. The ratio of average is 1.17. (D) Avearge squared jackknife s.e. vs. average squared deviation. The ratio of average across is 0.88. Numeric values are provided in **Supplementary Table 13**.

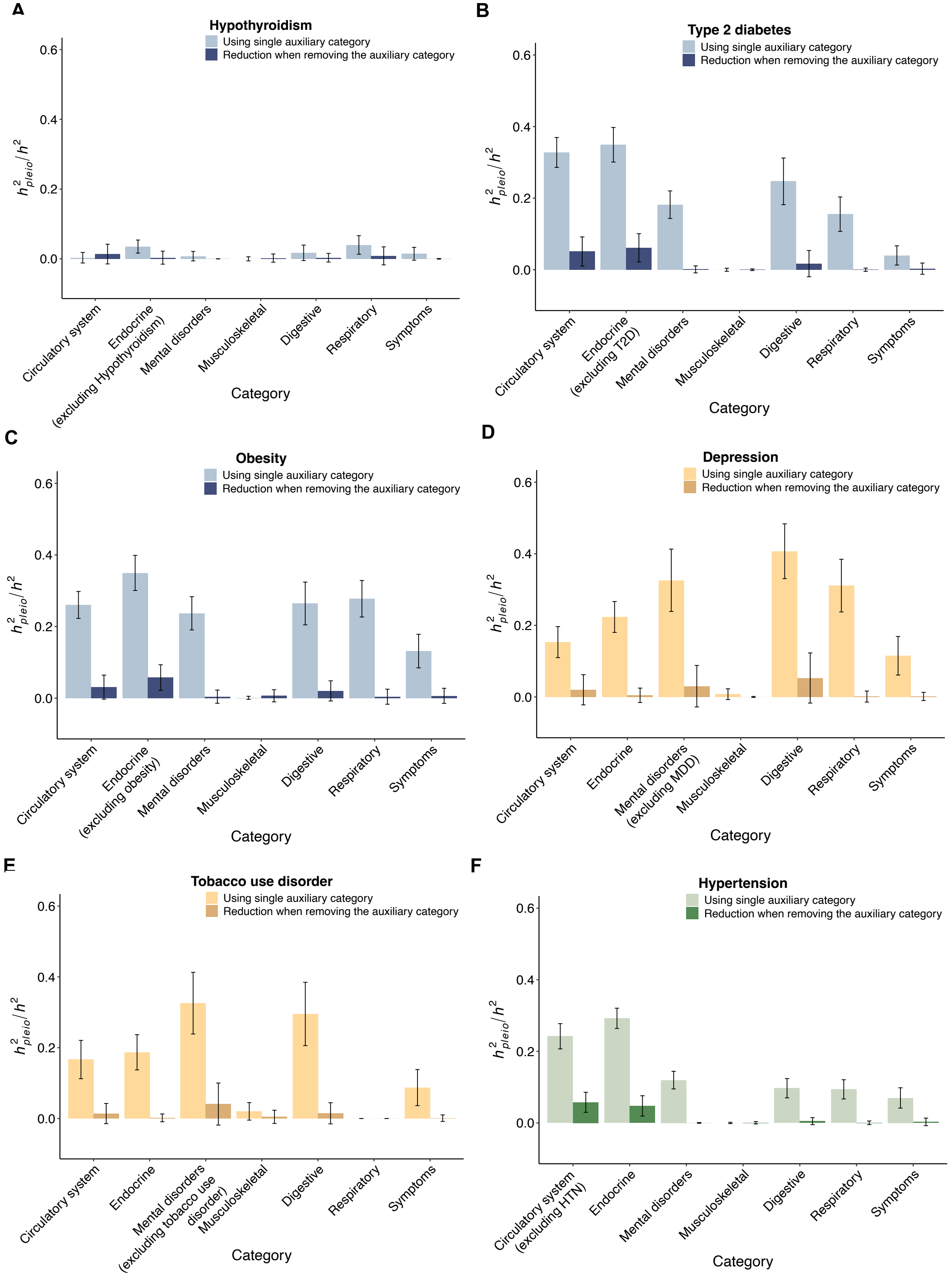

A
Hypothyroidism
Using single auxiliary category
Reduction when removing the auxiliary category
h^2_{pleio}/h^2
0.6
0.4
0.2
0.0
Circulatory system
Endocrine (excluding Hypothyroidism)
Mental disorders
Musculoskeletal
Digestive
Respiratory
Symptoms
Category
B
Type 2 diabetes
Using single auxiliary category
Reduction when removing the auxiliary category
Circulatory system
Endocrine (excluding T2D)
Mental disorders
Musculoskeletal
Digestive
Respiratory
Symptoms
Category
C
Obesity
Using single auxiliary category
Reduction when removing the auxiliary category
Circulatory system
Endocrine (excluding obesity)
Mental disorders
Musculoskeletal
Digestive
Respiratory
Symptoms
Category
D
Depression
Using single auxiliary category
Reduction when removing the auxiliary category
Circulatory system
Endocrine
Mental disorders (excluding MDD)
Musculoskeletal
Digestive
Respiratory
Symptoms
Category
E
Tobacco use disorder
Using single auxiliary category
Reduction when removing the auxiliary category
Circulatory system
Endocrine
Mental disorders (excluding tobacco use disorder)
Musculoskeletal
Digestive
Respiratory
Symptoms
Category
F
Hypertension
Using single auxiliary category
Reduction when removing the auxiliary category
Circulatory system (excluding HTN)
Endocrine
Mental disorders
Musculoskeletal
Digestive
Respiratory
Symptoms
Category

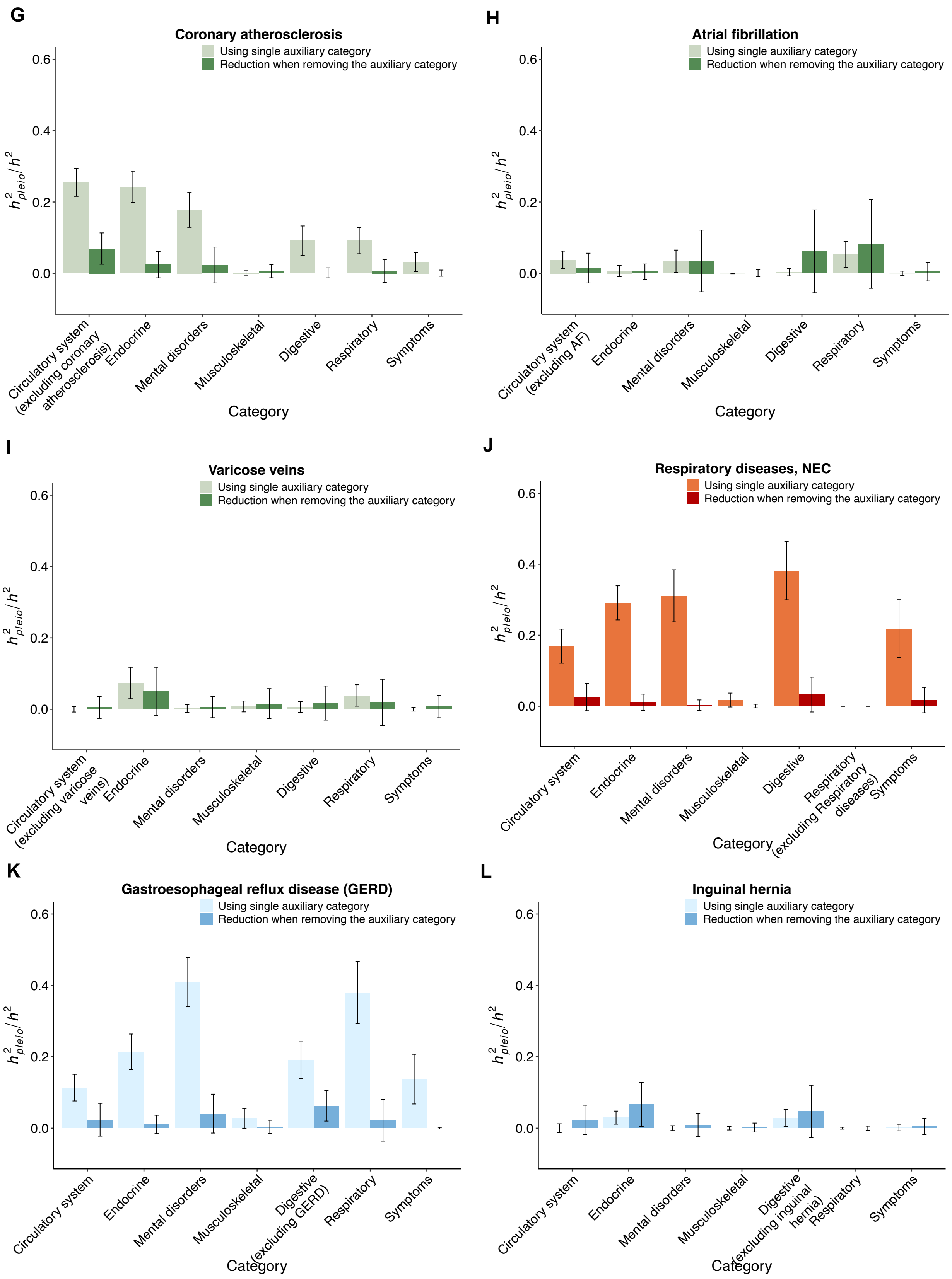
G
Coronary atherosclerosis
Using single auxiliary category
Reduction when removing the auxiliary category
0.6
0.4
0.2
0.0
Circulatory system (excluding coronary atherosclerosis)
Endocrine
Mental disorders
Musculoskeletal
Digestive
Respiratory
Symptoms
Category
H
Atrial fibrillation
Using single auxiliary category
Reduction when removing the auxiliary category
Circulatory system (excluding AF)
Endocrine
Mental disorders
Musculoskeletal
Digestive
Respiratory
Symptoms
Category
I
Varicose veins
Using single auxiliary category
Reduction when removing the auxiliary category
Circulatory system (excluding varicose veins)
Endocrine
Mental disorders
Musculoskeletal
Digestive
Respiratory
Symptoms
Category
J
Respiratory diseases, NEC
Using single auxiliary category
Reduction when removing the auxiliary category
Circulatory system
Endocrine
Mental disorders
Musculoskeletal
Digestive
Respiratory (excluding Respiratory diseases)
Symptoms
Category
K
Gastroesophageal reflux disease (GERD)
Using single auxiliary category
Reduction when removing the auxiliary category
Circulatory system
Endocrine
Mental disorders
Musculoskeletal
Digestive (excluding GERD)
Respiratory
Symptoms
Category
L
Inguinal hernia
Using single auxiliary category
Reduction when removing the auxiliary category
Circulatory system
Endocrine
Mental disorders
Musculoskeletal
Digestive (excluding inguinal hernia)
Respiratory
Symptoms
Category
h^2_{pleio}/h^2

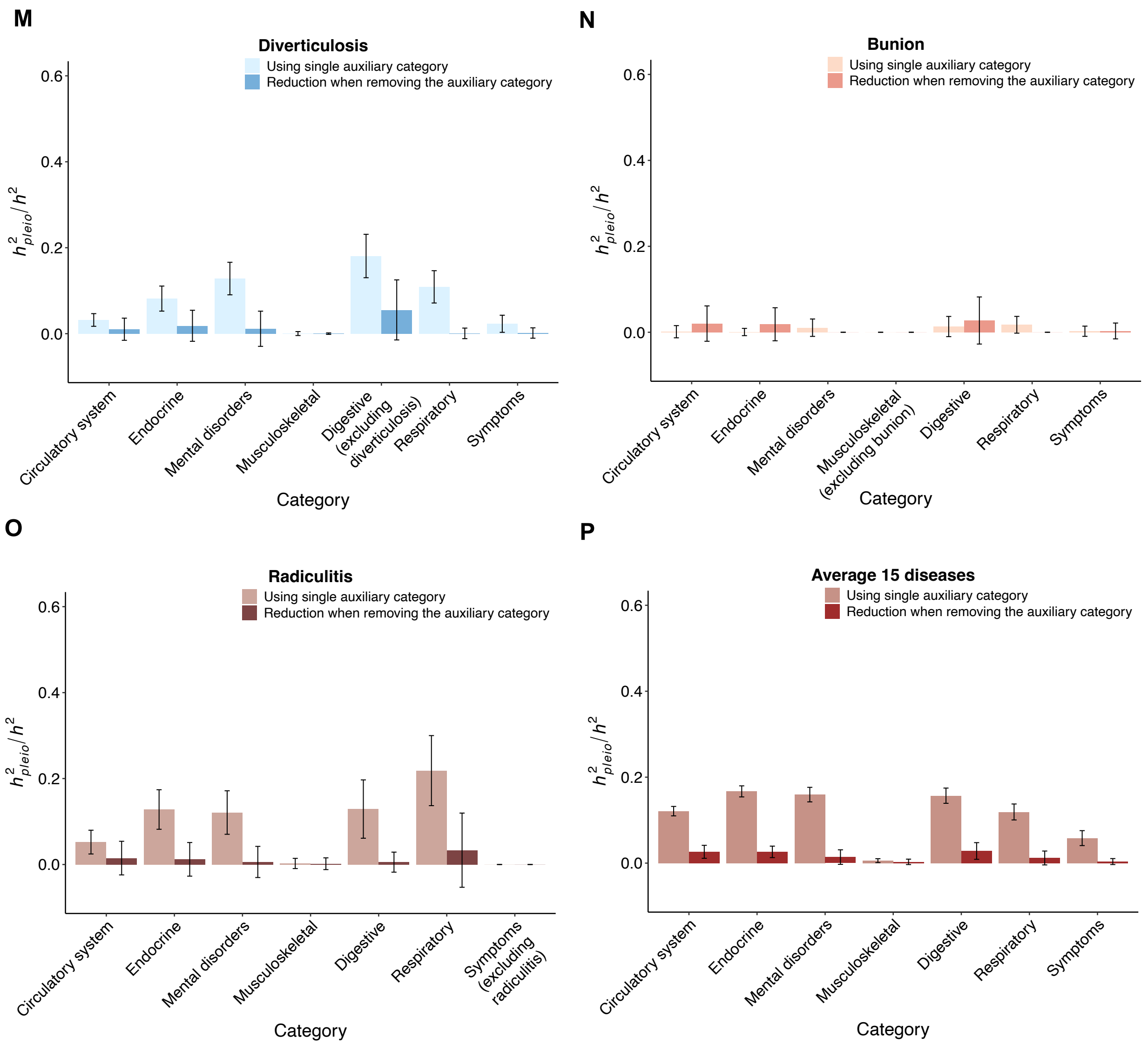

Supplementary Figure 20. Distribution of $h^2{}_{pleio}/h^2$ across Phecode disease categories.

(A-O) Comparison of $h^2{}_{pleio}/h^2$ between single-auxiliary-category estimate and reduction when removing the auxiliary category for all 15 UK Biobank target diseases and (P) the average across 15 UK Biobank diseases. Light color bar is the single-auxiliary-category estimate of $h^2{}_{pleio}/h^2$, dark color bar is the reduction of $h^2{}_{pleio}/h^2$ when removing the auxiliary category. The zero $h^2{}_{pleio}/h^2$ of single-auxiliary-category estimate indicates that this auxiliary category only has the target disease. The zero reduction of $h^2{}_{pleio}/h^2$ when removing the auxiliary category indicates that the estimation of $h^2{}_{pleio}/h^2$ for all auxiliary diseases has already excluded those auxiliary diseases in the removed auxiliary category after the pruning procedure. Therefore, further removal of this auxiliary category makes no difference. Different colors for different disease panels represent their target disease category. Data are presented as point estimate +/- s.e.. Error bar shows the jackknife standard error obtained via 200 genomic blocks. We reported

s.e. of reduction using the jackknife standard error of the $\frac{h^2{}_{pleio}}{h^2}$ reduction before bias correction. We compute the post-bias-correction standard error of the average estimate across 15 diseases using the average of ratio to scale the uncorrected jackknife s.e. of average: $(\frac{1}{15}\sum_{i=1}^{15}\frac{post-correction\ s.e._i}{pre-correction\ s.e._i})$ × uncorrected jackknife s.e. of average. Detailed results are provided in **Supplementary Table 19**. Abbreviation: T2D: type 2 diabetes; MDD: depression; HTN: hypertension; GERD: gastroesophageal reflux disease; AF: Atrial Fibrillation.

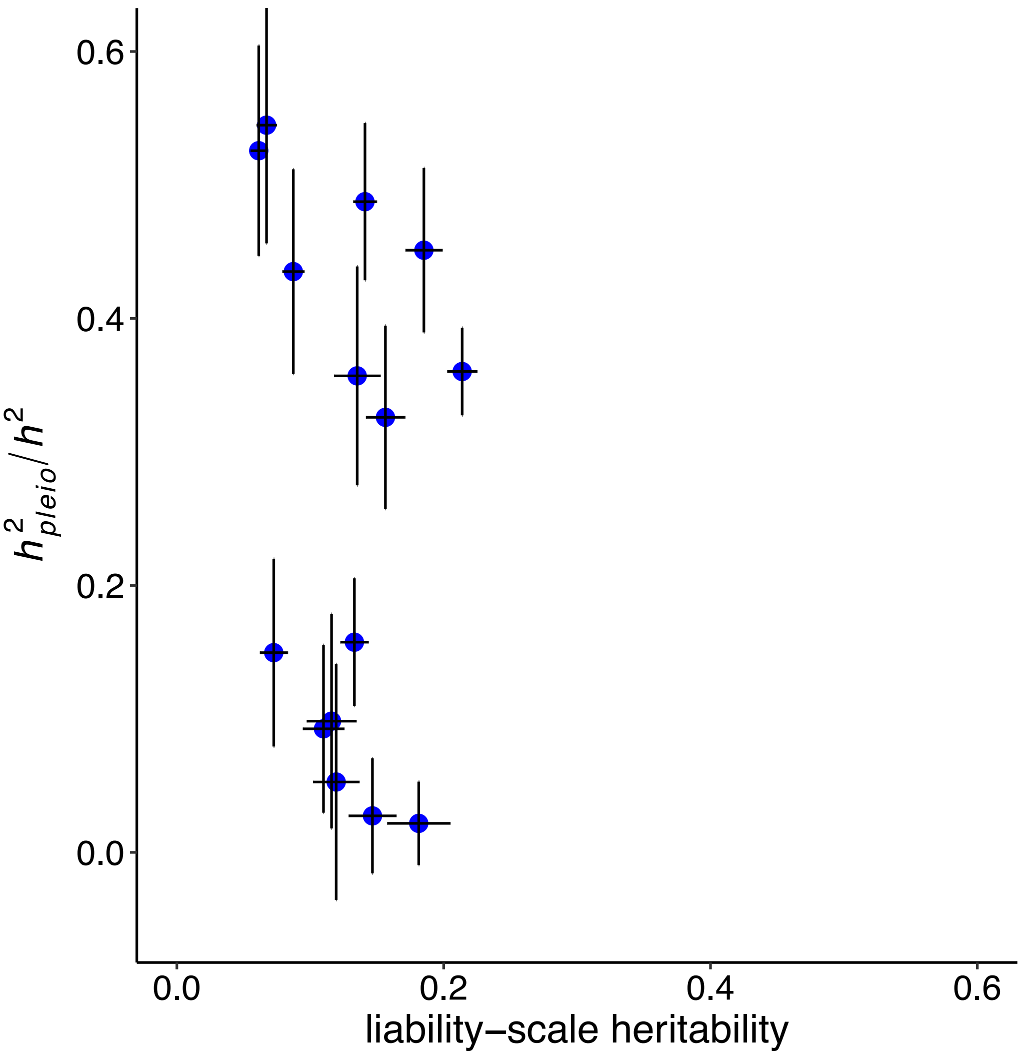

Supplementary Figure 21. Comparison on liability-scale heritability and h^2_{pleio}/h^2 across the 15 UK Biobank diseases.

Error bars are the jackknife standard error obtained via 200 genomic blocks. We observed no significant correlation (Pearson's r = -0.18 (*P* = 0.52)).

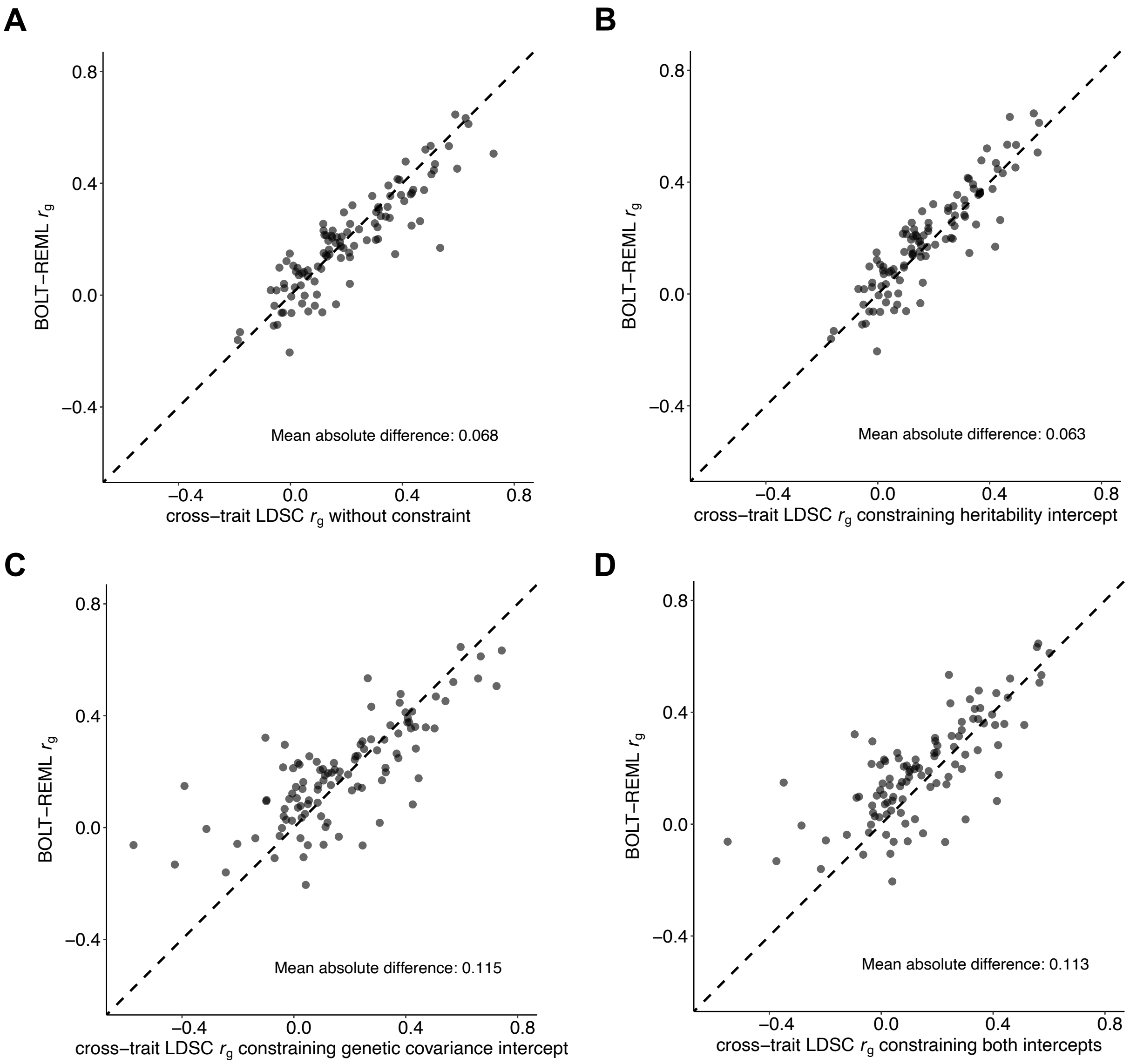

Supplementary Figure 22. Comparison on r_g from BOLT-REML and r_g from cross-trait LDSC with 4 options of constraining intercept.

(A) Compare r_g from BOLT-REML and r_g from cross-trait LDSC without constraint on intercept, which is the default version we used in main analyses. (B-D) Compare r_g from BOLT-REML and r_g from three modified version of cross-trait LDSC: constraining heritability intercept, constraining genetic covariance intercept, and constraining both intercepts. The computation of constrained intercept is provided in **Methods** section and **Supplementary Table 27**. Detailed results are provided in **Supplementary Table 20**.

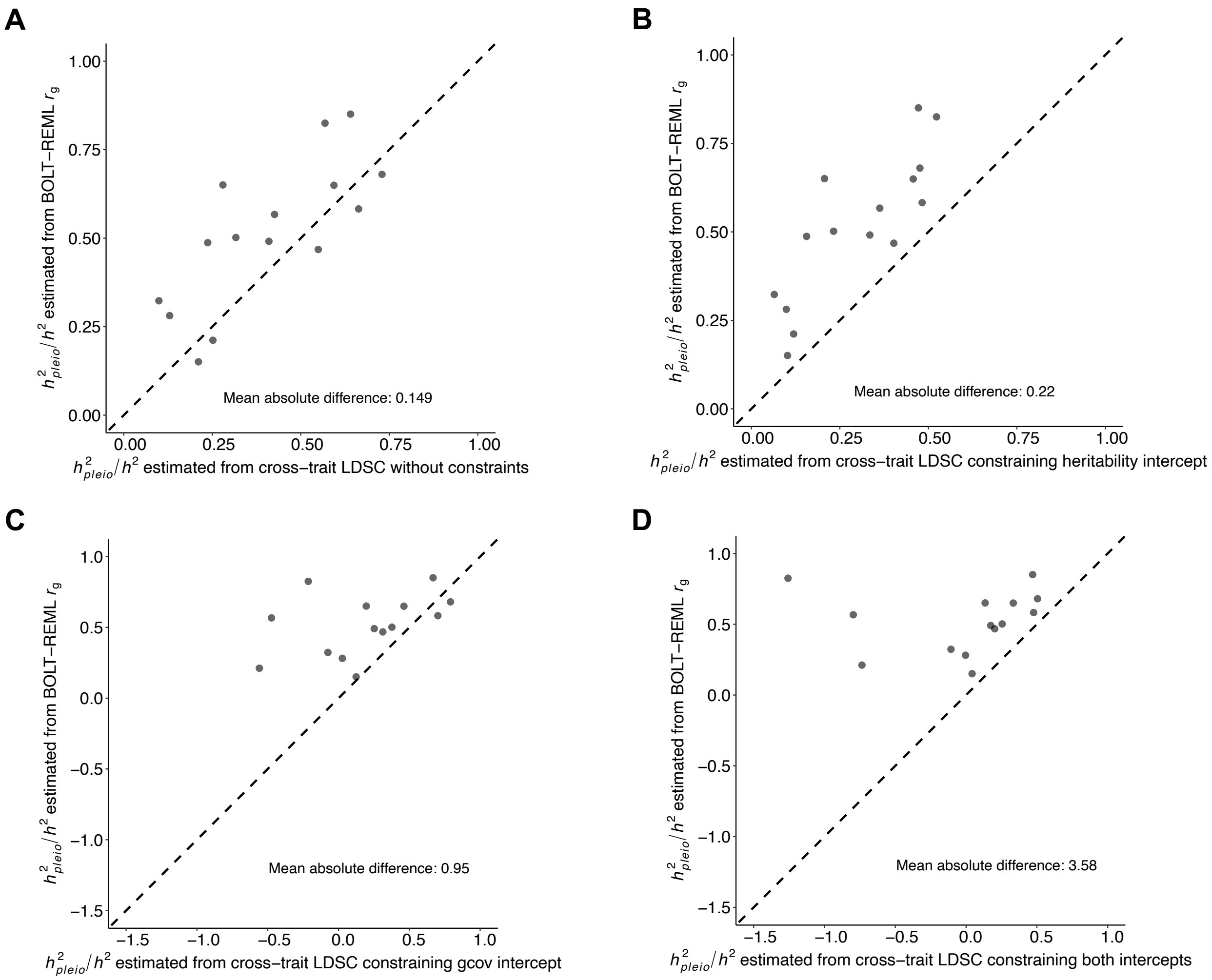

Supplementary Figure 23. Comparison on $h^2{}_{pleio}/h^2$ estimated from BOLT-REML r_g and $h^2{}_{pleio}/h^2$ estimated from cross-trait LDSC r_g with 4 options of constraining intercept.

(A) Compare $h^2{}_{pleio}/h^2$ estimated from BOLT-REML r_g and $h^2{}_{pleio}/h^2$ estimated from cross-trait LDSC r_g without constraint on intercept, which is the default version we estimated in main analyses. (B-D) Compare $h^2{}_{pleio}/h^2$ estimated from BOLT-REML r_g and $h^2{}_{pleio}/h^2$ estimated from r_g from three modified version of cross-trait LDSC: constraining heritability intercept, constraining genetic covariance intercept, and constraining both intercepts. We found that our results on $h^2{}_{pleio}/h^2$ using default version of cross-trait LDSC were broadly consistent with results from BOLT-REML, and all three modified versions deviated from BOLT-REML results in estimates of $h^2{}_{pleio}/h^2$. One outlier point was removed from both panels C and D for clarity in the figure visualization, while the mean absolute difference was computed based on the full 15 diseases. Detailed results are provided in **Supplementary Table 21**.

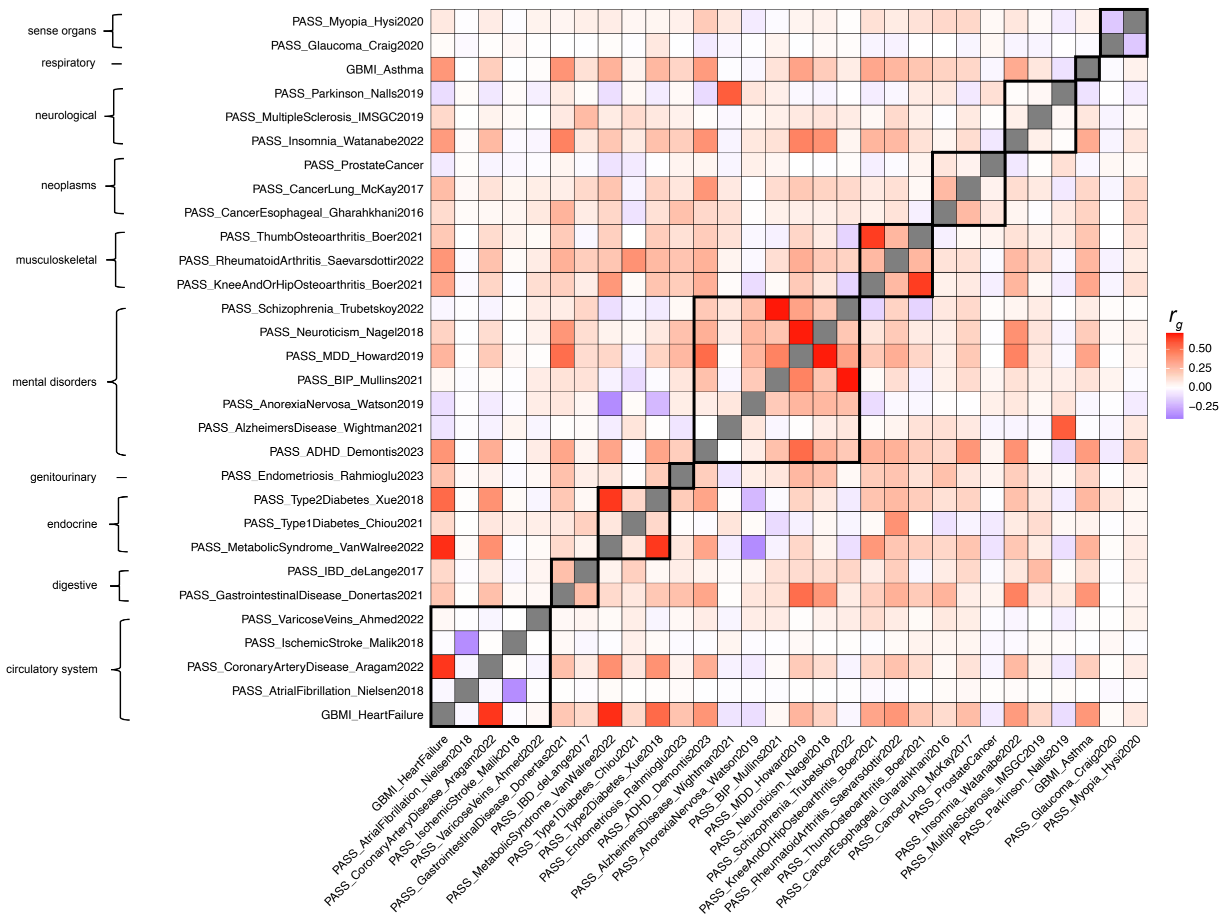

Supplementary Figure 24. Estimates of genetic correlation of 30 non-UK Biobank diseases.

Most diseases have moderate genetic correlations within disease categories and between disease categories. Black boxes demarcate correlations between diseases within the same disease category. Detailed results are provided in **Supplementary Table 16**.

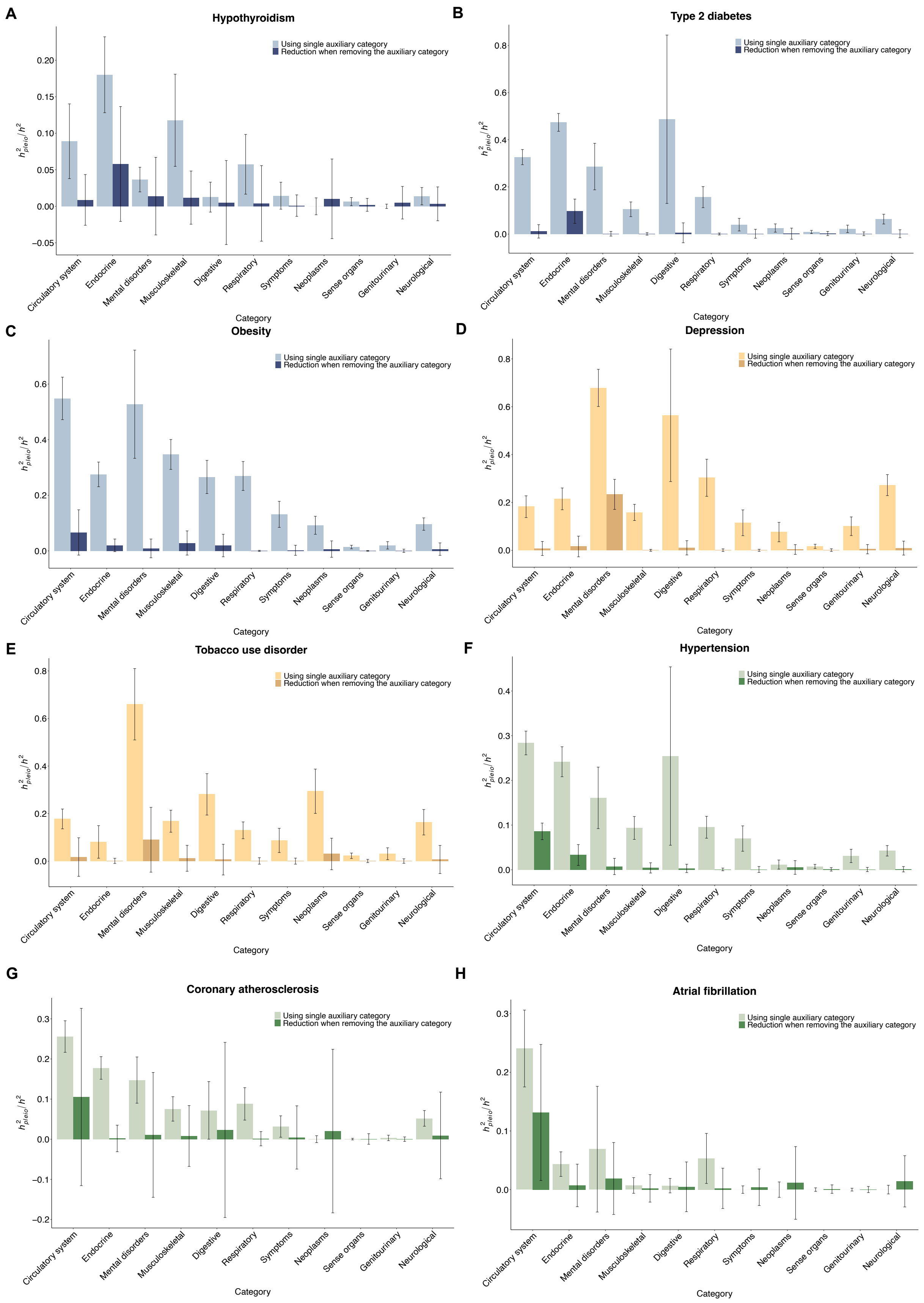
A
Hypothyroidism
B
Type 2 diabetes
C
Obesity
D
Depression
E
Tobacco use disorder
F
Hypertension
G
Coronary atherosclerosis
H
Atrial fibrillation
Using single auxiliary category
Reduction when removing the auxiliary category
h^2_{pleio}/h^2
Category
Circulatory system
Endocrine
Mental disorders
Musculoskeletal
Digestive
Respiratory
Symptoms
Neoplasms
Sense organs
Genitourinary
Neurological

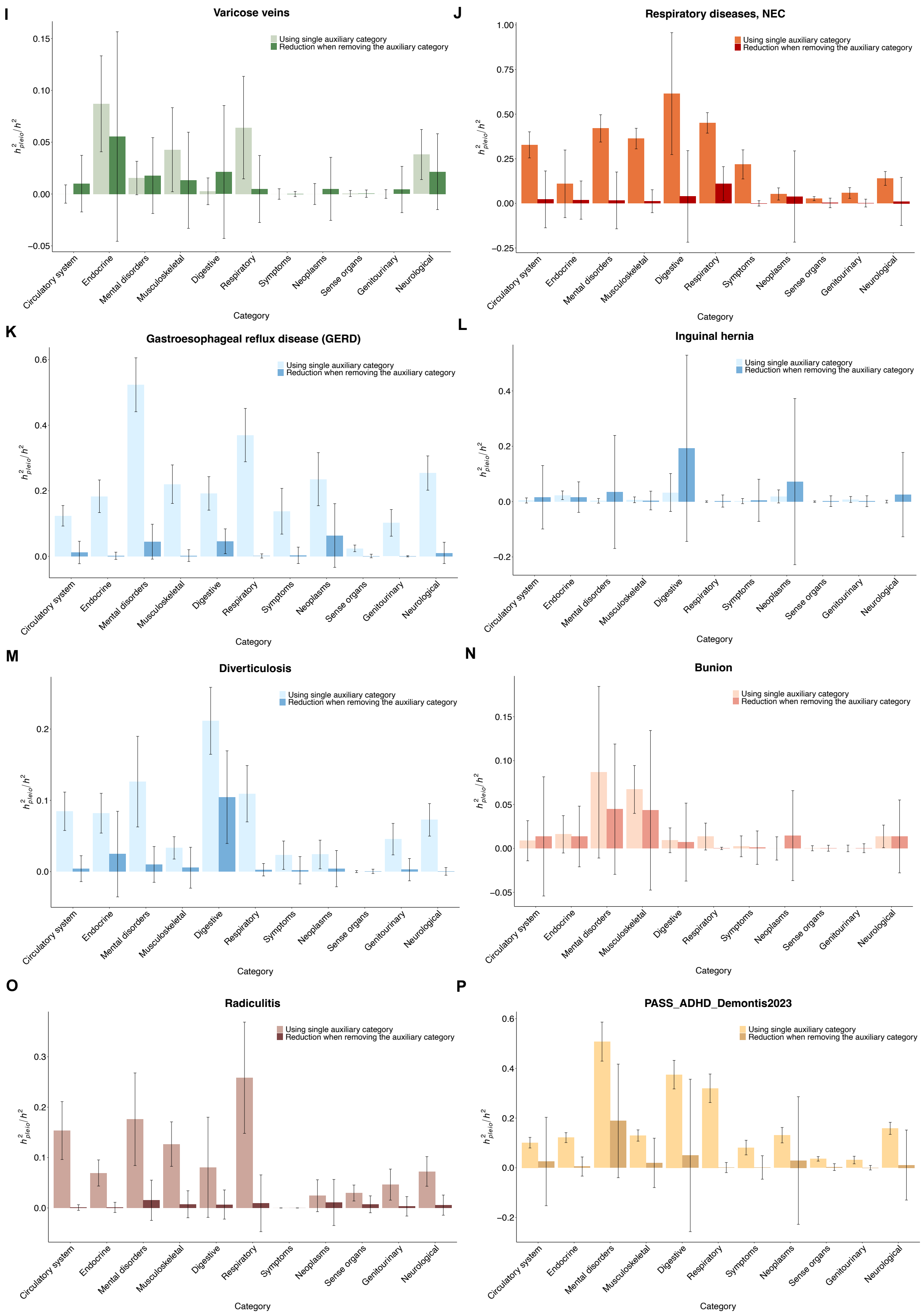
I
Varicose veins
Using single auxiliary category
Reduction when removing the auxiliary category
J
Respiratory diseases, NEC
Using single auxiliary category
Reduction when removing the auxiliary category
K
Gastroesophageal reflux disease (GERD)
Using single auxiliary category
Reduction when removing the auxiliary category
L
Inguinal hernia
Using single auxiliary category
Reduction when removing the auxiliary category
M
Diverticulosis
Using single auxiliary category
Reduction when removing the auxiliary category
N
Bunion
Using single auxiliary category
Reduction when removing the auxiliary category
O
Radiculitis
Using single auxiliary category
Reduction when removing the auxiliary category
P
PASS_ADHD_Demontis2023
Using single auxiliary category
Reduction when removing the auxiliary category
h^2_{pleio}/h^2
Circulatory system
Endocrine
Mental disorders
Musculoskeletal
Digestive
Respiratory
Symptoms
Neoplasms
Sense organs
Genitourinary
Neurological
Category

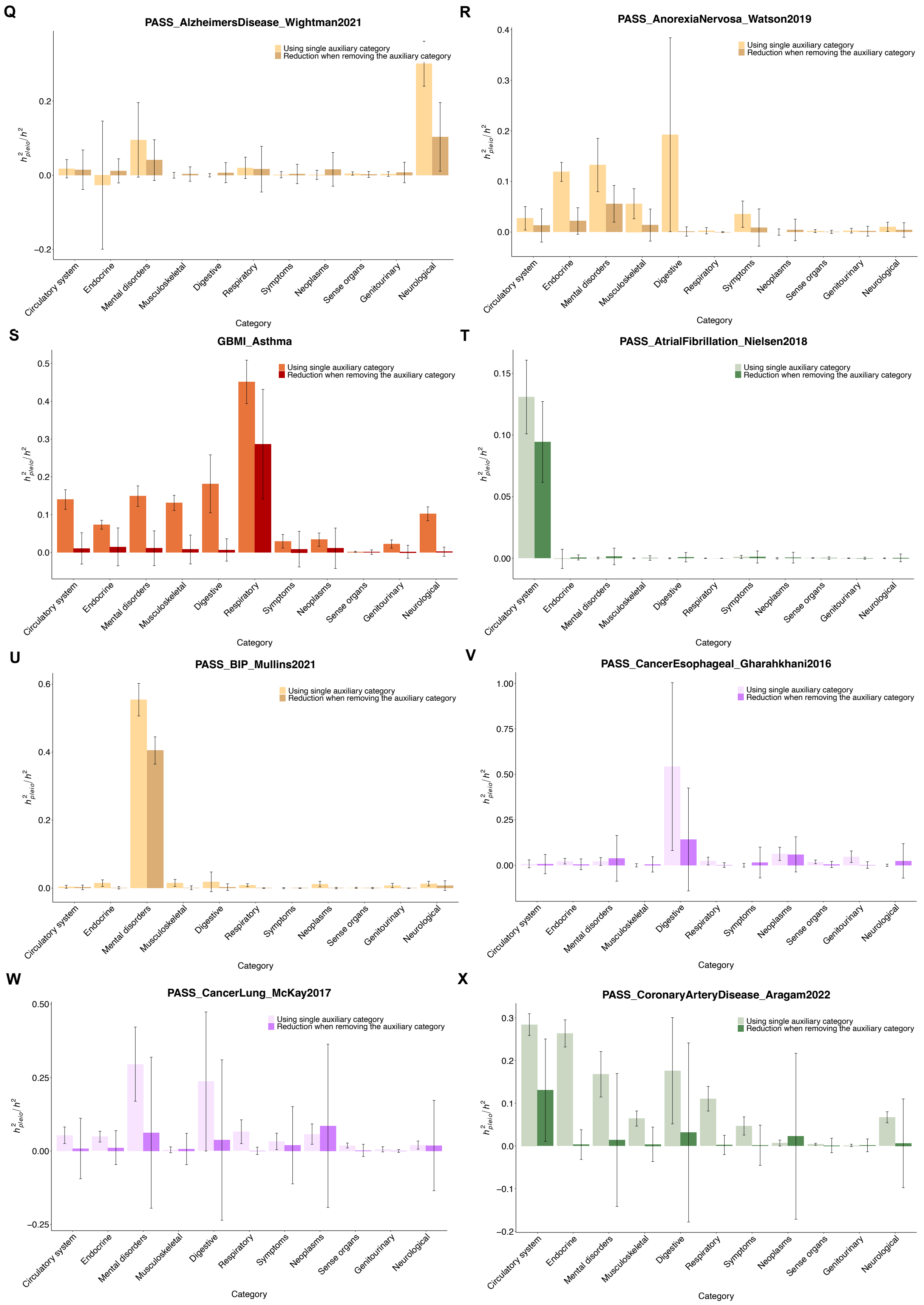
Q
PASS_AlzheimersDisease_Wightman2021
Using single auxiliary category
Reduction when removing the auxiliary category
h^2_{pleio}/h^2
0.2
0.0
−0.2
Circulatory system
Endocrine
Mental disorders
Musculoskeletal
Digestive
Respiratory
Symptoms
Neoplasms
Sense organs
Genitourinary
Neurological
Category
R
PASS_AnorexiaNervosa_Watson2019
Using single auxiliary category
Reduction when removing the auxiliary category
0.4
0.3
0.2
0.1
0.0
Category
S
GBMI_Asthma
Using single auxiliary category
Reduction when removing the auxiliary category
0.5
0.4
0.3
0.2
0.1
0.0
Category
T
PASS_AtrialFibrillation_Nielsen2018
Using single auxiliary category
Reduction when removing the auxiliary category
0.15
0.10
0.05
0.00
Category
U
PASS_BIP_Mullins2021
Using single auxiliary category
Reduction when removing the auxiliary category
0.6
0.4
0.2
0.0
Category
V
PASS_CancerEsophageal_Gharahkhani2016
Using single auxiliary category
Reduction when removing the auxiliary category
1.00
0.75
0.50
0.25
0.00
Category
W
PASS_CancerLung_McKay2017
Using single auxiliary category
Reduction when removing the auxiliary category
0.50
0.25
0.00
−0.25
Category
X
PASS_CoronaryArteryDisease_Aragam2022
Using single auxiliary category
Reduction when removing the auxiliary category
0.3
0.2
0.1
0.0
−0.1
−0.2
Category

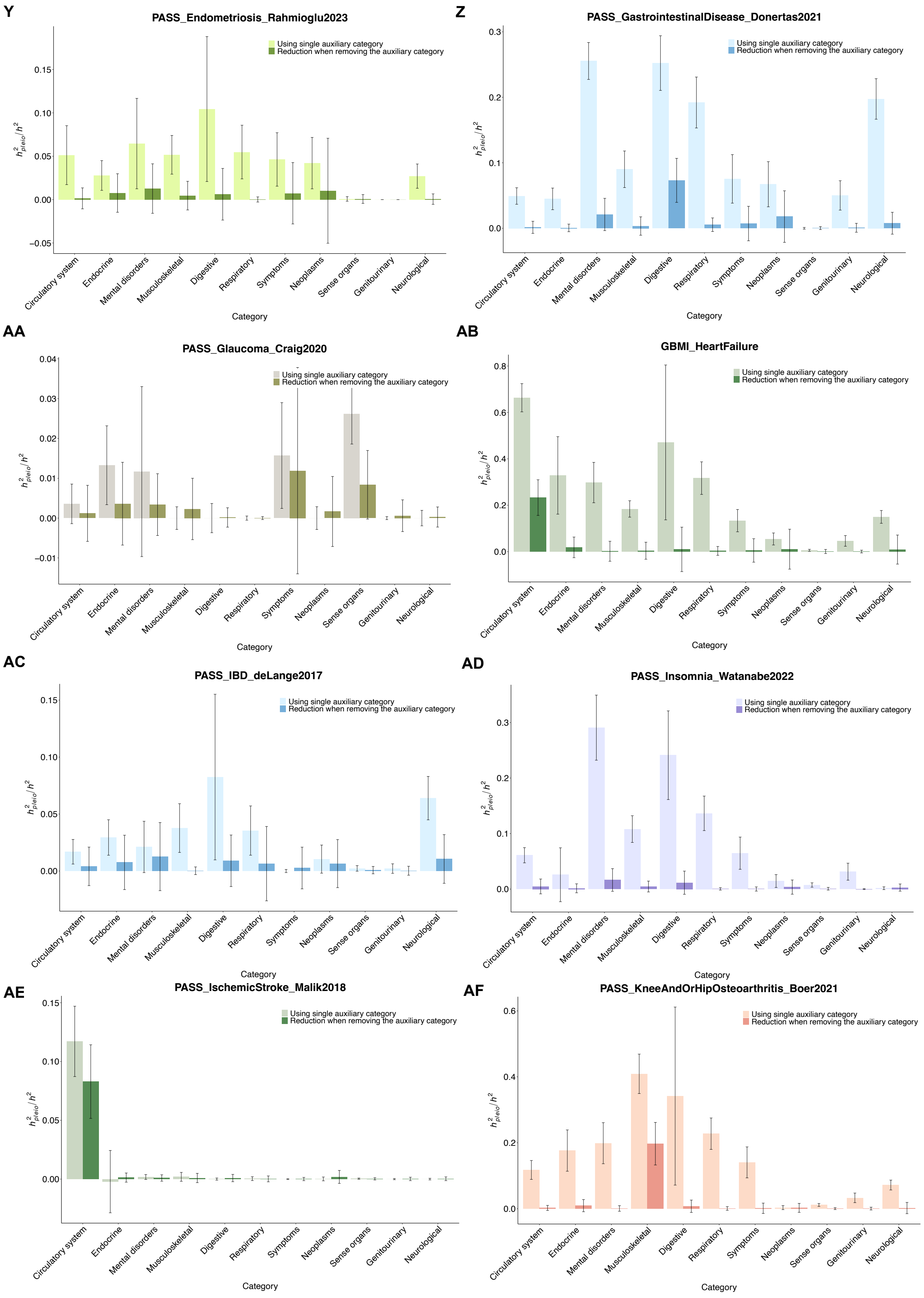
Y
PASS_Endometriosis_Rahmioglu2023
Using single auxiliary category
Reduction when removing the auxiliary category
Z
PASS_GastrointestinalDisease_Donertas2021
Using single auxiliary category
Reduction when removing the auxiliary category
AA
PASS_Glaucoma_Craig2020
Using single auxiliary category
Reduction when removing the auxiliary category
AB
GBMI_HeartFailure
Using single auxiliary category
Reduction when removing the auxiliary category
AC
PASS_IBD_deLange2017
Using single auxiliary category
Reduction when removing the auxiliary category
AD
PASS_Insomnia_Watanabe2022
Using single auxiliary category
Reduction when removing the auxiliary category
AE
PASS_IschemicStroke_Malik2018
Using single auxiliary category
Reduction when removing the auxiliary category
AF
PASS_KneeAndOrHipOsteoarthritis_Boer2021
Using single auxiliary category
Reduction when removing the auxiliary category
h^2_{pleio}/h^2
Category
Circulatory system
Endocrine
Mental disorders
Musculoskeletal
Digestive
Respiratory
Symptoms
Neoplasms
Sense organs
Genitourinary
Neurological

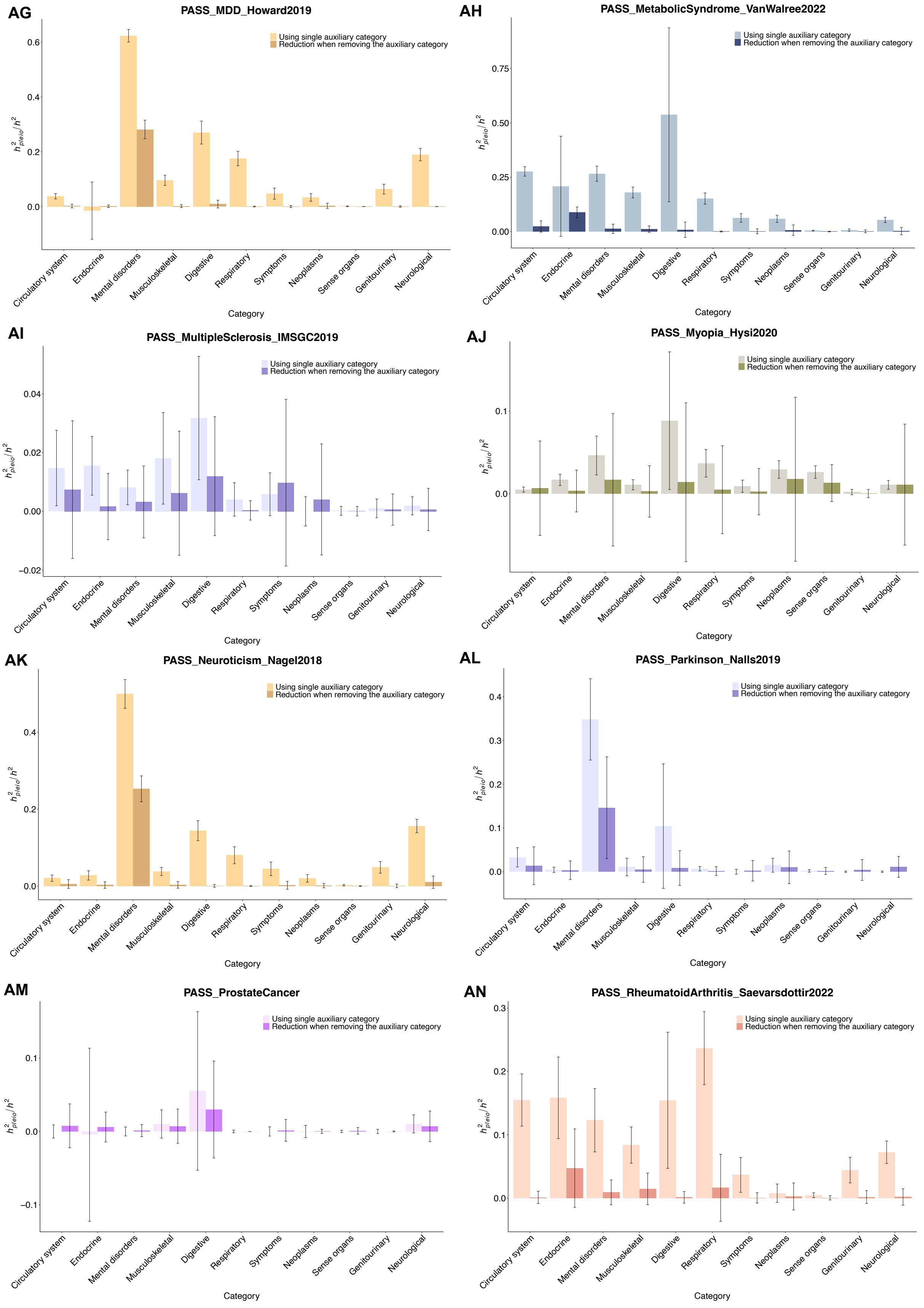
AG
PASS_MDD_Howard2019
AH
PASS_MetabolicSyndrome_VanWalree2022
AI
PASS_MultipleSclerosis_IMSGC2019
AJ
PASS_Myopia_Hysi2020
AK
PASS_Neuroticism_Nagel2018
AL
PASS_Parkinson_Nalls2019
AM
PASS_ProstateCancer
AN
PASS_RheumatoidArthritis_Saevarsdottir2022
Using single auxiliary category
Reduction when removing the auxiliary category
h^2_{pleio}/h^2
Category
Circulatory system
Endocrine
Mental disorders
Musculoskeletal
Digestive
Respiratory
Symptoms
Neoplasms
Sense organs
Genitourinary
Neurological

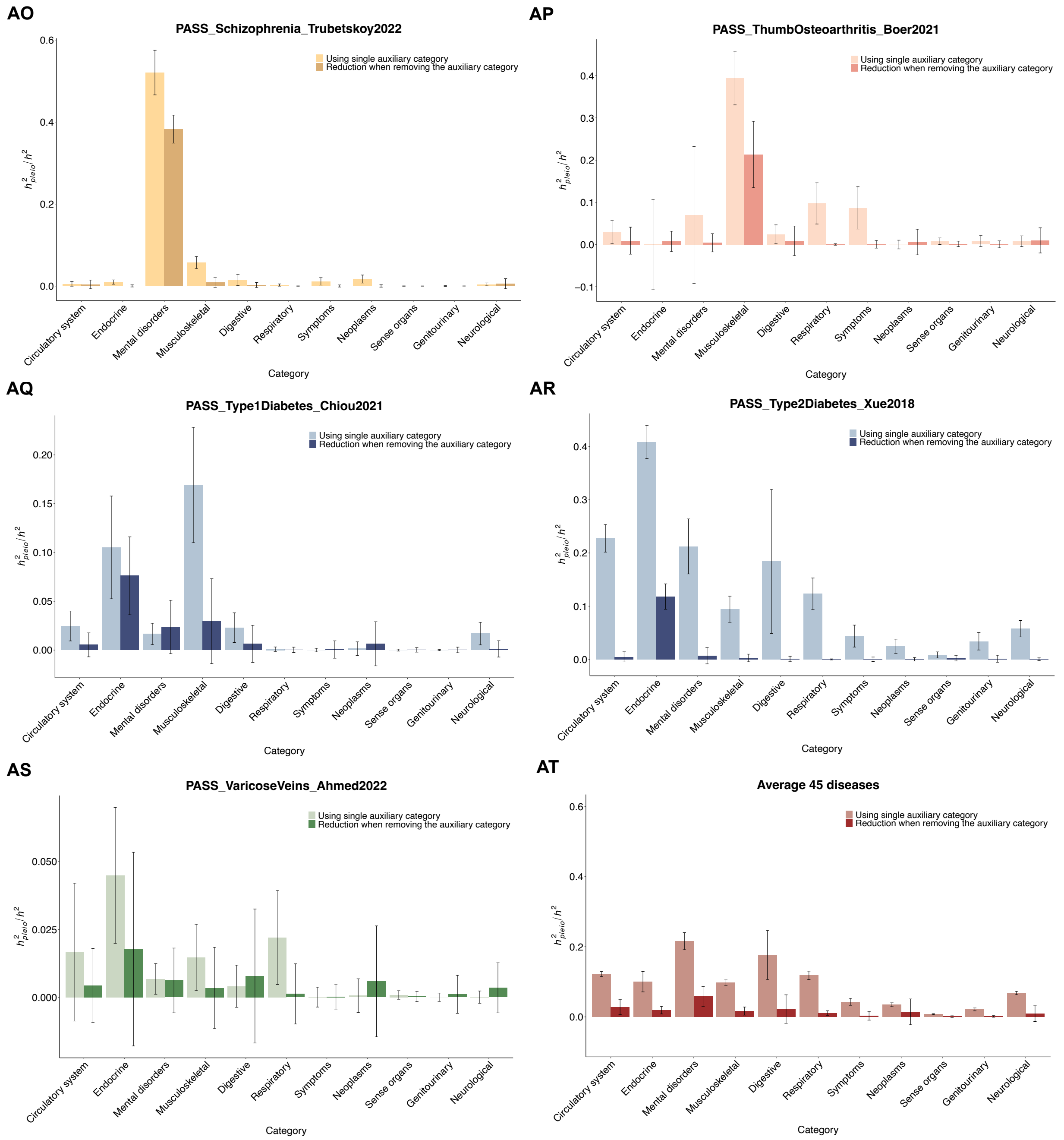

Supplementary Figure 25. Distribution of $h^2{}_{pleio}/h^2$ across Phecode disease categories using all 45 auxiliary diseases.

(A-O) Comparison of $h^2{}_{pleio}/h^2$ between single-auxiliary-category estimate and reduction when removing the auxiliary category for all 15 UK Biobank target diseases, (P-AS) for all 30 non-UK Biobank target diseases, and (AT) the average across all 45 UK Biobank + non-UK Biobank diseases. Light color bar is the single-auxiliary-category estimate of $h^2{}_{pleio}/h^2$, dark color bar is the reduction of $h^2{}_{pleio}/h^2$ when removing the auxiliary category. The zero $h^2{}_{pleio}/h^2$ of single-auxiliary-category estimate indicates that this auxiliary category only has the target

disease. The zero reduction of $h^2{}_{pleio}/h^2$ when removing the auxiliary category indicates that the estimation of $h^2{}_{pleio}/h^2$ for all auxiliary diseases has already excluded those auxiliary diseases in the removed auxiliary category after the pruning procedure. Therefore, further removal of this auxiliary category makes no difference. Different colors of different disease panels represent their target disease category. Data are presented as point estimate +/- s.e.. Error bar shows the jackknife standard error obtained via 200 genomic blocks. We reported s.e. of reduction using the jackknife standard error $\frac{h^2{}_{pleio}}{h^2}$ reduction before bias correction. We compute the post-bias-correction standard error of the average estimate across 45 diseases using the average of ratio to scale the uncorrected jackknife s.e. of average: $(\frac{1}{45}\sum_{i=1}^{45}\frac{post-correction\ s.e._i}{pre-correction\ s.e._i}) \times$ uncorrected jackknife s.e. of average. When the target disease category serves as the auxiliary category, auxiliary diseases within this category exclude the target disease. Detailed results are provided in **Supplementary Table 24**. Abbreviation: T2D: type 2 diabetes; MDD: depression; HTN: hypertension; GERD: gastroesophageal reflux disease; AF: Atrial Fibrillation.

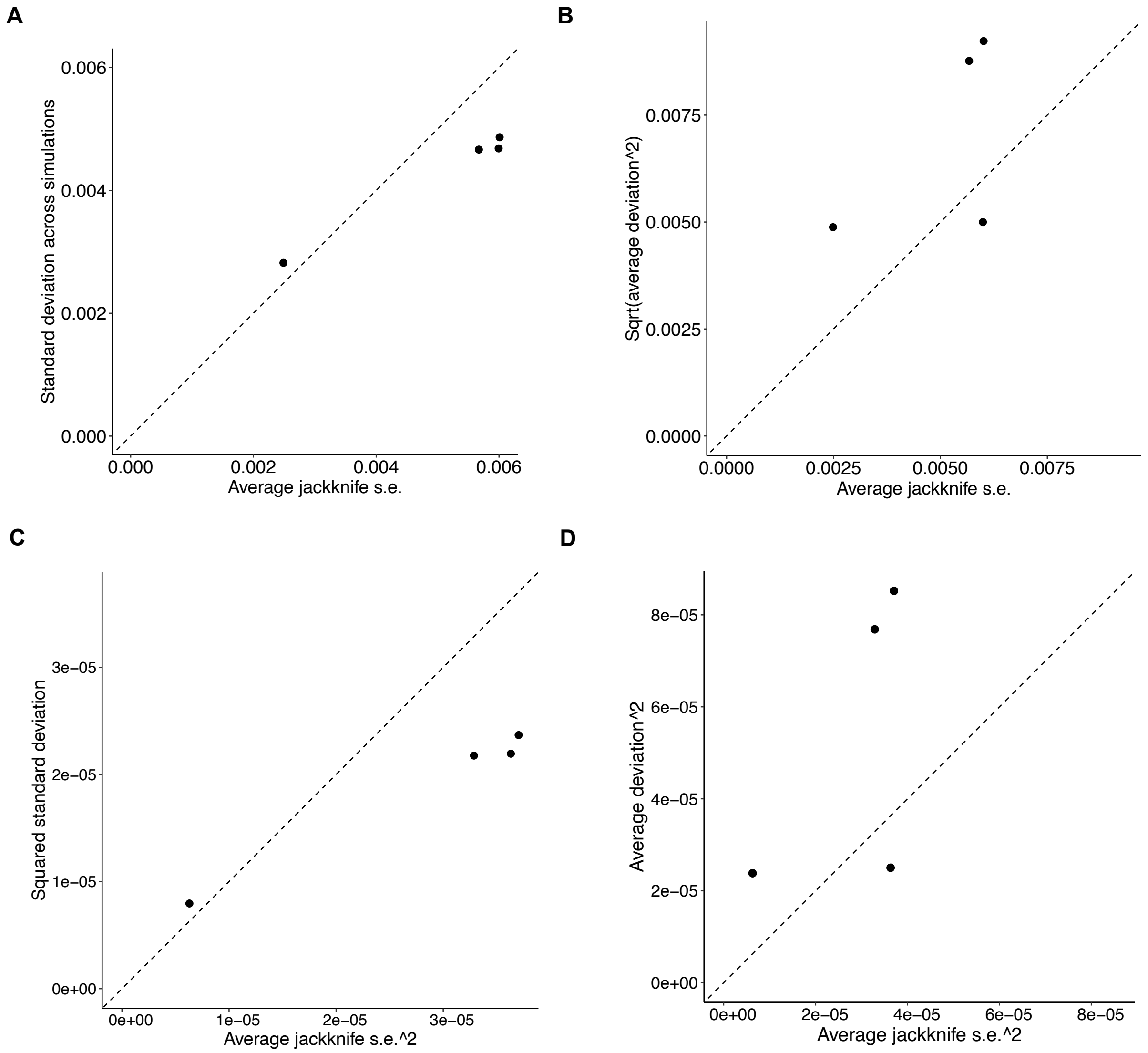

Supplementary Figure 26. Calibration of estimated standard errors of $V^2{}_{pleio}/V^2$.

Four points correspond to four different values of true $V^2{}_{pleio}/V^2$. We estimate the standard error by jackknifing over blocks of individuals across all diseases. We report four different ratios: (1) (average jackknife s.e.) / (standard deviation); (2) (average jackknife s.e.) / ($\sqrt{average\ squared\ deviation}$); (3) (average jackknife s.e.^2) / (squared standard deviation); (4) (average jackknife s.e.^2) / (average squared deviation). (A) The average of ratio (1) across 4 categories is 1.15. (B) The average of ratio (2) across 4 categories is 0.75. Deviation is the difference between true $V^2{}_{pleio}/V^2$ and the estimated $V^2{}_{pleio}/V^2$. (C) The average of ratio (3) across 4 categories is 1.38. (D) The average of ratio (4) across 4 categories is 0.65. We determined that this anti-conservative standard error does not impact our results, as the standard errors of $V^2{}_{pleio}/V^2$ estimates are small given the large sample size of UK Biobank data. Detailed results are provided in **Supplementary Table 25**.

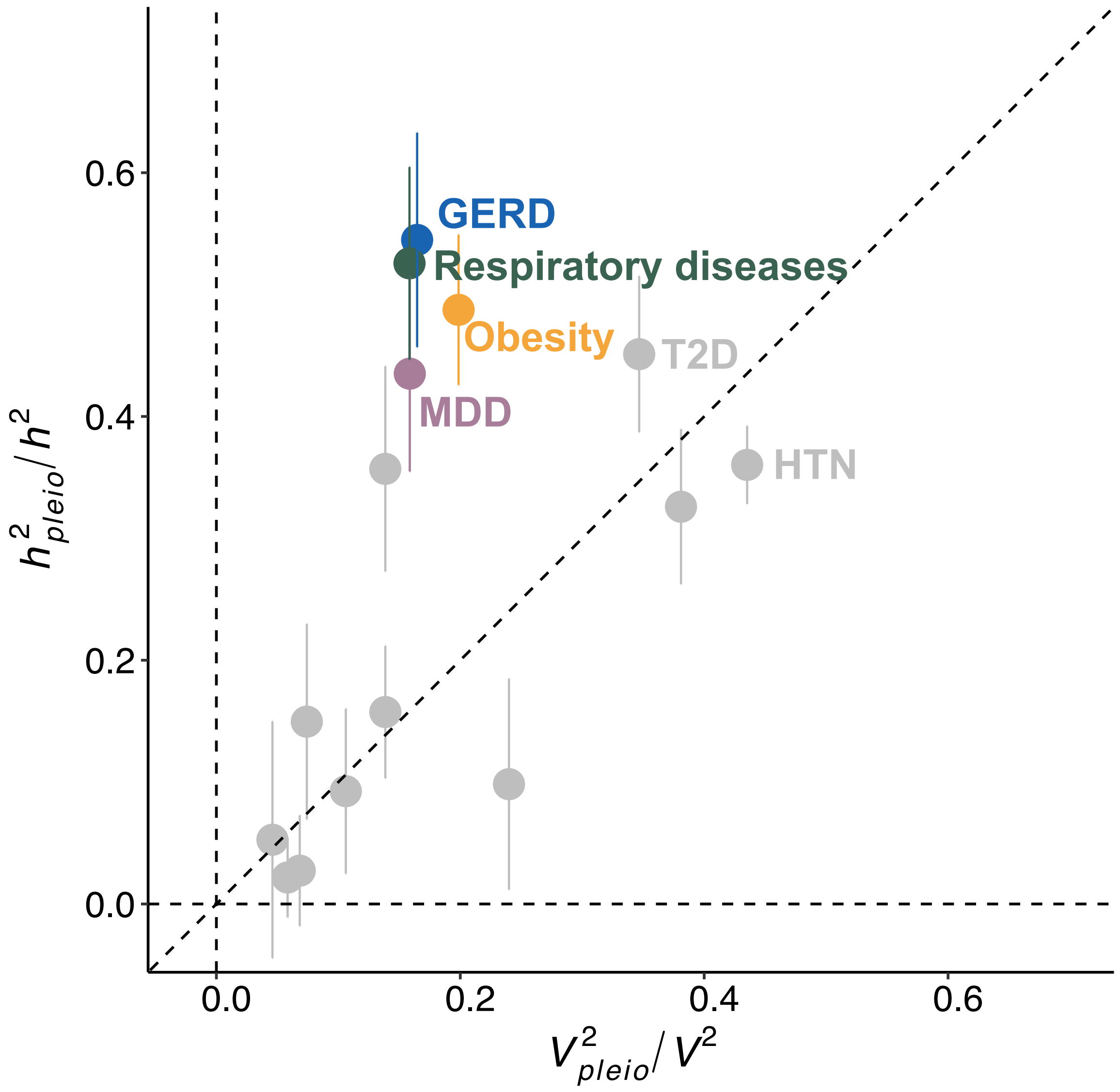

Supplementary Figure 27. Scatter plot of $h^2{}_{pleio}/h^2$ vs. $V^2{}_{pleio}/V^2$ for the 15 UK Biobank diseases.

We use the same set of auxiliary diseases after pruning in comparing $h^2{}_{pleio}/h^2$ and $V^2{}_{pleio}/V^2$. Four points with color have $h^2{}_{pleio}/h^2$ estimation significantly different from $V^2{}_{pleio}/V^2$ (Bonferroni-corrected two-sided $P < 0.05/15$ for multiple comparisons). This figure is the same as **Figure 7** but adding error bar. Data are presented as point estimate +/- s.e.. Error bar shows the jackknife standard error (but s.e. of $V^2{}_{pleio}/V^2$ is generally smaller than point size). The estimates of $h^2{}_{pleio}/h^2$ were generally larger than estimates of $V^2{}_{pleio}/V^2$ (ratio of averages = 1.51x (s.e. 0.16)). We compute the ratio of averages using $\frac{average\ h^2{}_{pleio}/h^2 \text{ across 15 diseases}}{average\ V^2{}_{pleio}/V^2\ across\ 15\ diseases}$ and s.e. using $\frac{s.e. of\ average\ h^2{}_{pleio}/h^2 \text{ across 15 diseases}}{average\ V^2{}_{pleio}/V^2 \text{ across 15 diseases}}$ which we

assume s.e. of average ${V^2}_{pleio}/V^2$ is 0. Detailed results are provided in **Supplementary Table 26**. Abbreviation: MDD: depression; T2D: type 2 diabetes; HTN: hypertension; GERD: gastroesophageal reflux disease; AF: Atrial Fibrillation.

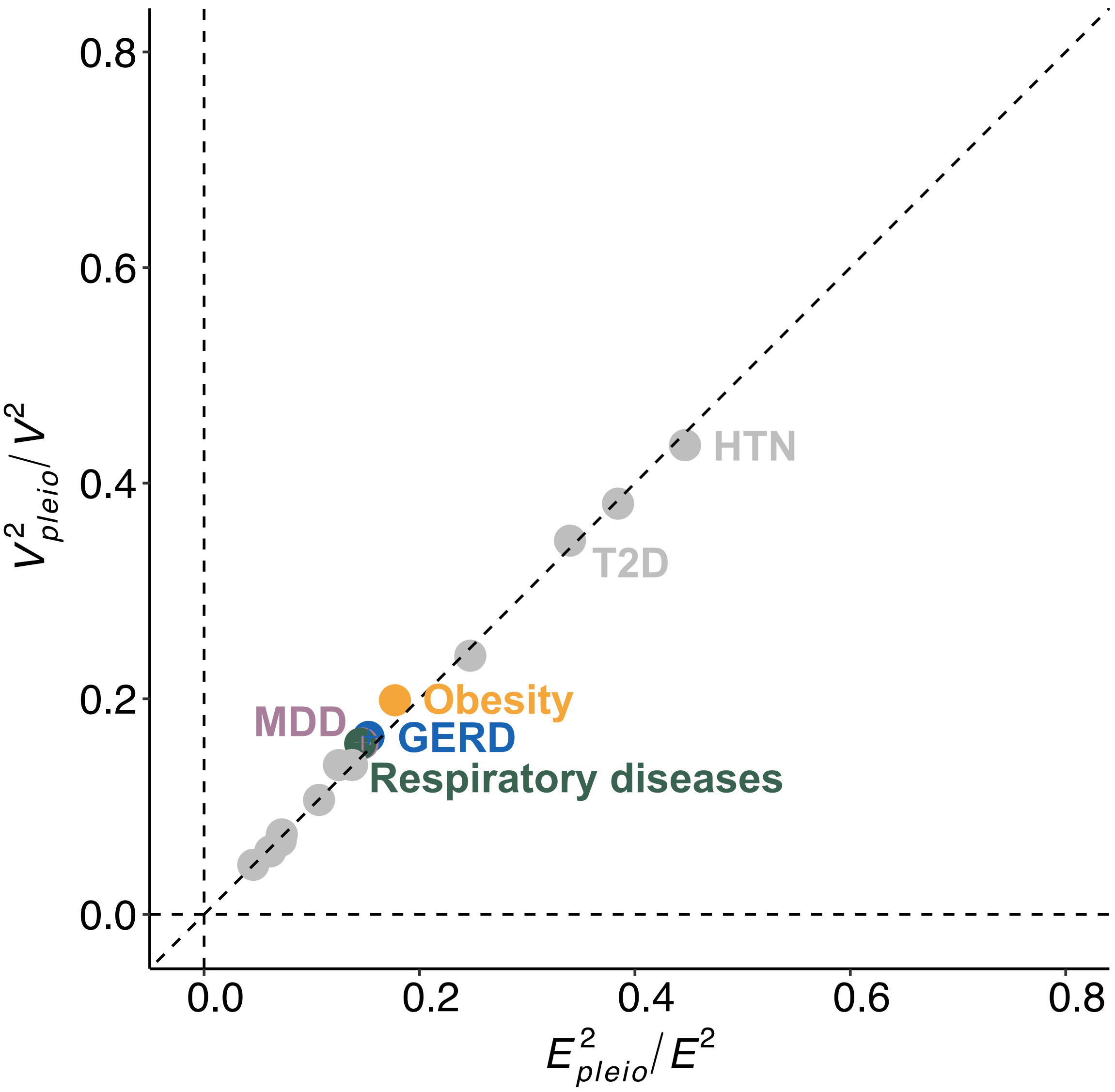

Supplementary Figure 28. Scatter plot of $V^2{}_{pleio}/V^2$ vs. $E^2{}_{pleio}/E^2$ for the 15 diseases.

We used the same set of auxiliary diseases after pruning in comparing $V^2{}_{pleio}/V^2$ and $E^2{}_{pleio}/E^2$. The colors of points are the same with **Figure 7**. Detailed results are provided in **Supplementary Table 26**. $E^2{}_{pleio}/E^2$ were slightly smaller than estimates of $V^2{}_{pleio}/V^2$ (ratio of averages = 0.98x). We computed the s.e of $E^2{}_{pleio}/E^2$ using $\frac{\sqrt{(s.e. of\ V^2{}_{pleio})^2+(s.e. of\ h^2{}_{pleio})^2}}{V^2-h^2}$ where we assumed that s.e. of E^2 is 0. Data are presented as point estimate +/- s.e.. Error bars denote standard errors (but are generally smaller than point size). Abbreviation: MDD: depression; T2D: type 2 diabetes; HTN: hypertension; GERD: gastroesophageal reflux disease; AF: Atrial Fibrillation.

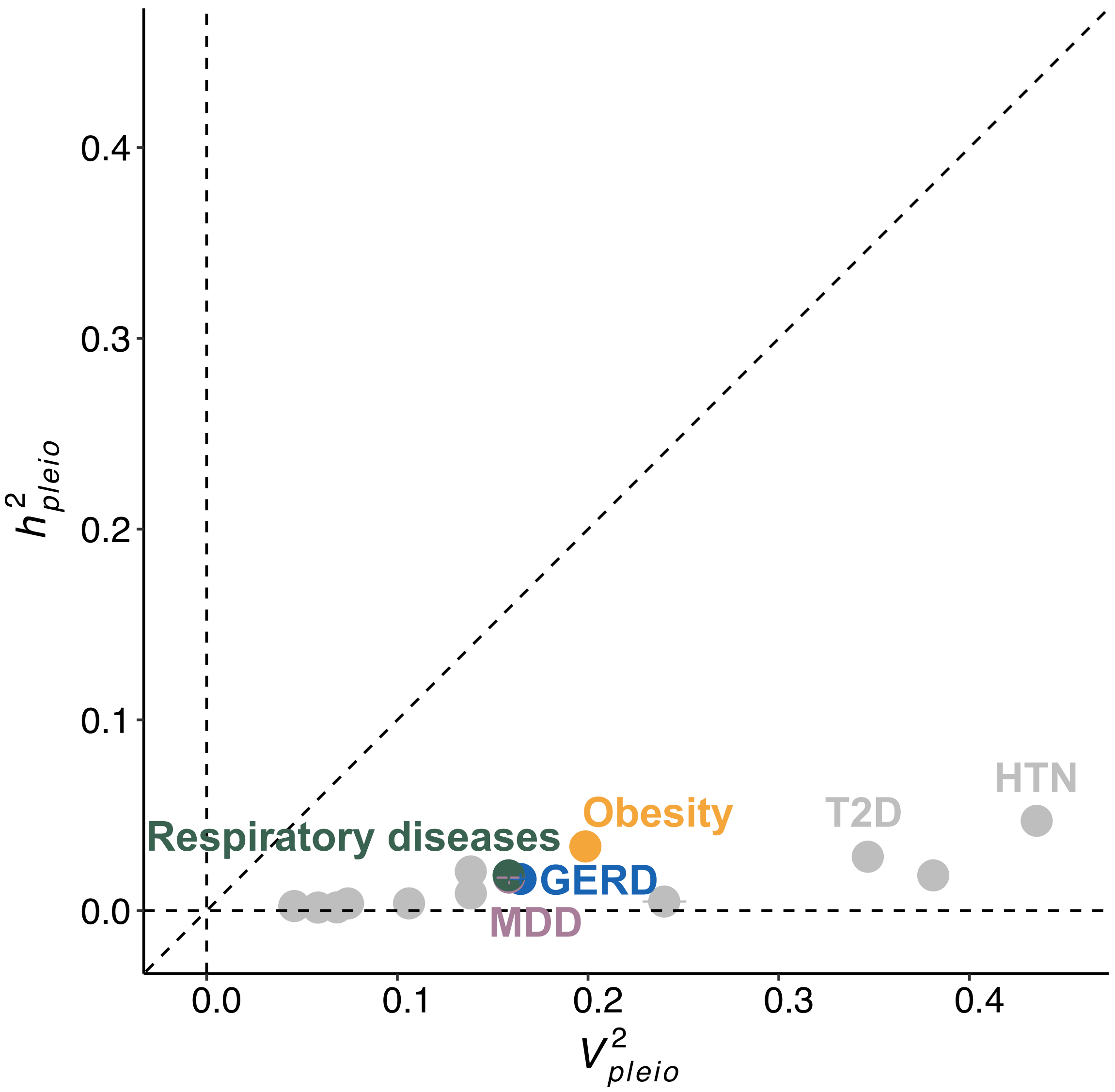

Supplementary Figure 29. Scatter plot of $h^2{}_{pleio}$ vs. $V^2{}_{pleio}$ for the 15 diseases.

We used the same set of auxiliary diseases after pruning in comparing $h^2{}_{pleio}$ and $V^2{}_{pleio}$ (the ratio of averages = 0.084 (s.e. 0.009). We computed the ratio of averages using $\frac{average\ h^2{}_{pleio}\ \text{across 15 diseases}}{average\ V^2{}_{pleio}\ across\ 15\ diseases}$ and s.e. using $\frac{s.e. of\ average\ h^2{}_{pleio}\ \text{across 15 diseases}}{average\ V^2{}_{pleio}\ \text{across 15 diseases}}$ where we assumed s.e. of average $V^2{}_{pleio}$ is 0. We computed the s.e of $h^2{}_{pleio}$ using $s.e. of\ h^2_{pleio}/h^2 \times h^2$ where we assumed that s.e. of h^2 is small relative to h^2_{pleio}. The colors of points are the same with **Figure 7.** Data are presented as point estimate +/- s.e.. Error bars denote standard errors (but are generally smaller than point size). Detailed results are provided in **Supplementary Table 26**. Abbreviation: MDD: depression; T2D: type 2 diabetes; HTN: hypertension; GERD: gastroesophageal reflux disease.

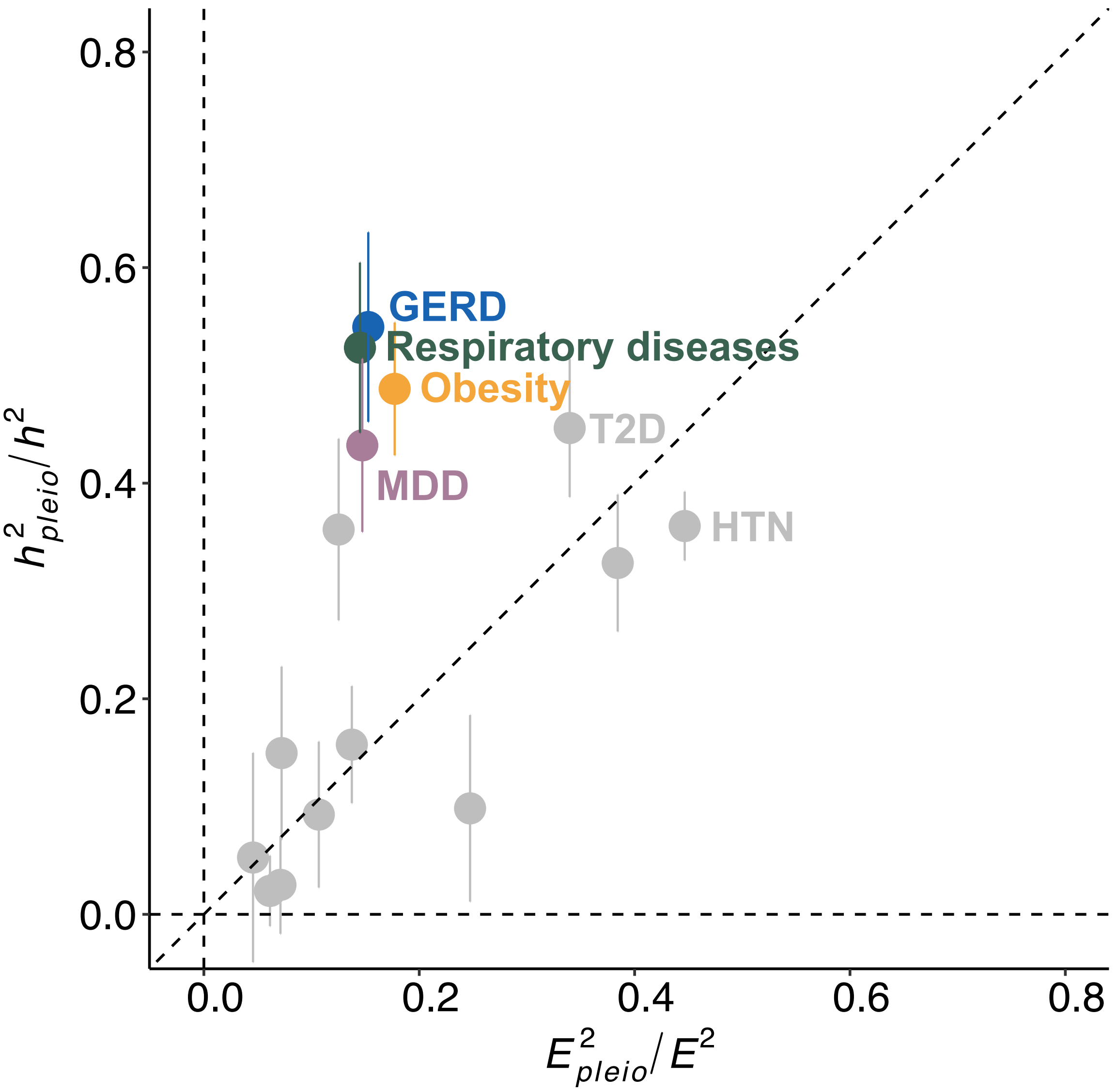

Supplementary Figure 30. Scatter plot of $h^2{}_{pleio}/h^2$ vs. $E^2{}_{pleio}/E^2$ for the 15 diseases.

We used the same set of auxiliary diseases after pruning in comparing $h^2{}_{pleio}/h^2$ and $E^2{}_{pleio}/E^2$. The colors of points are the same with **Figure 7**. The estimates of $h^2{}_{pleio}/h^2$ were generally larger than estimates of $E^2{}_{pleio}/E^2$ (ratio of averages = 1.54x). We computed the s.e of $E^2{}_{pleio}/E^2$ using $\frac{\sqrt{(s.e.of\ V^2{}_{pleio})^2+(s.e.of\ h^2{}_{pleio})^2}}{V^2-h^2}$ where we assumed that s.e. of E^2 is 0. Data are presented as point estimate +/- s.e.. Error bars denote standard errors (but s.e. of $E^2{}_{pleio}/E^2$ is generally smaller than point size). Detailed results are provided in **Supplementary Table 26**. Abbreviation: MDD: depression; T2D: type 2 diabetes; HTN: hypertension; GERD: gastroesophageal reflux disease; AF: Atrial Fibrillation.

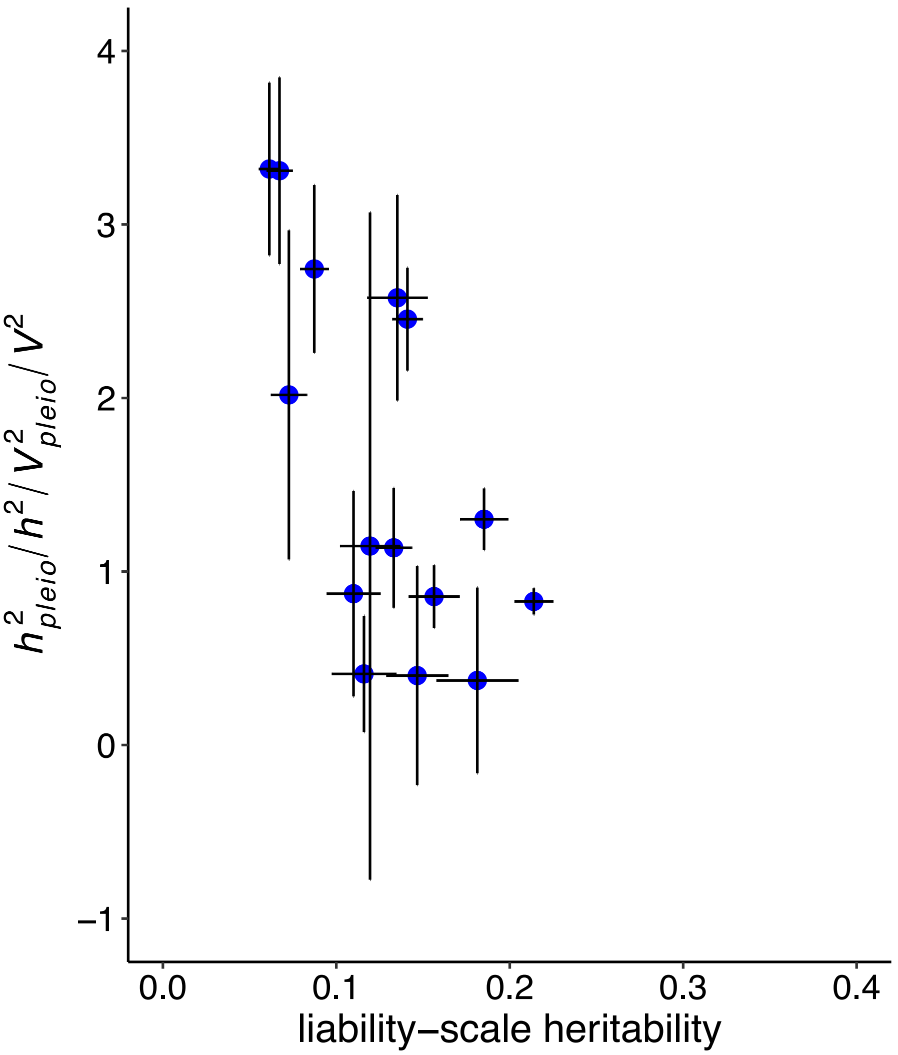

Supplementary Figure 31. Scatter plot of liability-scale heritability and the ratio of $\boldsymbol{h^2}_{\boldsymbol{pleio}}/\boldsymbol{h^2}$ to $\boldsymbol{V^2}_{\boldsymbol{pleio}}/\boldsymbol{V^2}$ across the 15 UK Biobank diseases.

Error bars are the jackknife standard error. We compute the s.e. of $\frac{h^2{}_{pleio}/h^2}{V^2{}_{pleio}/V^2}$ using $\frac{s.e.of\ h^2{}_{pleio}/h^2}{V^2{}_{pleio}/V^2}$ which we assume s.e. of $V^2{}_{pleio}/V^2$ is 0. We observed a negative correlation (Pearson's r = -0.65, *P* = 0.008). The p-value of 0.008 is anti-conservative, as it treats the 15 diseases as independent when they are in fact correlated, and it is thus unclear whether the correlation is statistically significant.
