# Supplementary material for: Pleiotropic shared heritability quantifies the shared genetic variance of common diseases
Source: Nat Genet. 2026 Jun 9;58(6):1248–57. doi: 10.1038/s41588-026-02607-w (PMC13256260; doi:10.1038/s41588-026-02607-w)
Supplement: Supplementary file 2 — Reporting Summary [file 41588_2026_2607_MOESM2_ESM.pdf]

Corresponding author(s): Xilin Jiang
Alkes Price
Yujie Zhao

Last updated by author(s): Apr 12, 2026

Reporting Summary

Nature Portfolio wishes to improve the reproducibility of the work that we publish. This form provides structure for consistency and transparency in reporting. For further information on Nature Portfolio policies, see our Editorial Policies and the Editorial Policy Checklist.

Statistics

For all statistical analyses, confirm that the following items are present in the figure legend, table legend, main text, or Methods section.

n/a	Confirmed	
☐	☒	The exact sample size (*n*) for each experimental group/condition, given as a discrete number and unit of measurement
☐	☒	A statement on whether measurements were taken from distinct samples or whether the same sample was measured repeatedly
☐	☒	The statistical test(s) used AND whether they are one- or two-sided<br>*Only common tests should be described solely by name; describe more complex techniques in the Methods section.*
☐	☒	A description of all covariates tested
☐	☒	A description of any assumptions or corrections, such as tests of normality and adjustment for multiple comparisons
☐	☒	A full description of the statistical parameters including central tendency (e.g. means) or other basic estimates (e.g. regression coefficient) AND variation (e.g. standard deviation) or associated estimates of uncertainty (e.g. confidence intervals)
☐	☒	For null hypothesis testing, the test statistic (e.g. *F*, *t*, *r*) with confidence intervals, effect sizes, degrees of freedom and *P* value noted<br>*Give P values as exact values whenever suitable.*
☐	☒	For Bayesian analysis, information on the choice of priors and Markov chain Monte Carlo settings
☐	☒	For hierarchical and complex designs, identification of the appropriate level for tests and full reporting of outcomes
☐	☒	Estimates of effect sizes (e.g. Cohen's *d*, Pearson's *r*), indicating how they were calculated

Our web collection on statistics for biologists contains articles on many of the points above.

Software and code

Policy information about availability of computer code

Data collection	No software was involved in data collection (data used is all directly available from UK Biobank, as described in detail in the paper)
Data analysis	We have released open-source software implementing PHBC at https://github.com/yjzhao1004/pleioh2g, and published on CRAN (https://cran.r-project.org/web/packages/pleioh2g). We have also deposited PHBC codes in https://doi.org/10.5281/zenodo.19550445. Other publicly available software packages used in this study are listed below: GWAS summary statistics were computed using PLINK2 (https://www.cog-genomics.org/plink/2.0/) and BOLT-LMM v2.4.1 (https://alkesgroup.broadinstitute.org/BOLT-LMM/downloads/), Heritability and genetic correlation were estimated using LDSC (https://github.com/bulik/ldsc), the ldscr R package (https://github.com/mglev1n/ldscr), and BOLT-REML as implemented in BOLT-LMM v 2.4.1.

For manuscripts utilizing custom algorithms or software that are central to the research but not yet described in published literature, software must be made available to editors and reviewers. We strongly encourage code deposition in a community repository (e.g. GitHub). See the Nature Portfolio guidelines for submitting code & software for further information.

Data

Policy information about availability of data

All manuscripts must include a data availability statement. This statement should provide the following information, where applicable:

- Accession codes, unique identifiers, or web links for publicly available datasets
- A description of any restrictions on data availability
- For clinical datasets or third party data, please ensure that the statement adheres to our policy

Access to individual-level UK Biobank data, both phenotypic and genetic, is available to bona fide researchers through application on the UK Biobank website (https://www.ukbiobank.ac.uk). Additional information about registration for access to the data is available at http://www.ukbiobank.ac.uk/register-apply/. Use of UK Biobank data was performed under application number 19542. LD-scores and HapMap 3 SNPs list are available at https://data.broadinstitute.org/alkesgroup/LDSCORE. Summary association statistics for all diseases/traits analyzed in this study are available at https://alkesgroup.broadinstitute.org/PHBC/.

Research involving human participants, their data, or biological material

Policy information about studies with human participants or human data. See also policy information about sex, gender (identity/presentation), and sexual orientation and race, ethnicity and racism.

Reporting on sex and gender	Both male and female subjects from UK Biobank study were included in the study. All GWAS summary statistics computation using BOLT-LMM were adjusted for sex.
Reporting on race, ethnicity, or other socially relevant groupings	We restricted our analyses to UK Biobank individuals of European ancestry. We collected the 30 diseases from publicly available GWAS meta-analyses that are primarily from European ancestry.
Population characteristics	We selected 228,258 samples with both primary care and hospital inpatient records. Diagnoses from primary care data were retrieved from a subset of UK Biobank samples (see below), which are recorded in Read Codes v.2 (Read v.2) and Read Codes Clinical Terms v.3 (Read CTv.3). We mapped primary care data from Read v.2 to ICD-10 codes, and combine with the ICD-10 codes from HES data. We kept ICD-10 codes starting with the letters A to N which are disease codes. Then, we mapped the ICD-10 records to PheCode system to obtain the phenotype definition and selected the PheCode phenotypes with > 1% prevalence in the 228,258 samples. We selected 157,206 unrelated (defined as having less than 11 putative third-degree relatives in the kinship table) British ancestry individuals from the 228,258 samples to compute GWAS summary statistics, where we used 1,141,346 HapMap 3 SNPs. Details are stated in Methods section - UK Biobank data. We used age and sex as the covariates in constructing GWAS for 15 UK Biobank diseases using BOLT-LMM. For 17 UK Biobank quantitative traits, we also corrected blood biochemistry measurements for cholesterol, hypertensive, and diabetic medications: total cholesterol, LDL, and triglyceride levels were corrected for lipid lowering medications (Ref. 64); systolic blood pressure and diastolic blood pressure were corrected for hypertension medications (Ref. 65); and HbA1c level was corrected for non-insulin diabetic medications and insulin drugs (Ref. 66).
Recruitment	The UK Biobank is a prospective, population-based cohort that recruited more than 500,000 participants aged 37 - 73 years who attended 1 of 22 assessment centers across the United Kingdom between 2006 and 2010. Previous investigation showed UK biobank subject to a healthy sample bias.
Ethics oversight	The UKB has received approval from the National Information Governance Board for Health and Social Care and the National Health Service North West Centre for Research Ethics Committee (Ref: 11/NW/0382). All participants provided informed consent through electronic signature at the baseline assessment. This study utilized publicly available dataset UK Biobank and therefore did not require ethical approval.

Note that full information on the approval of the study protocol must also be provided in the manuscript.

Field-specific reporting

Please select the one below that is the best fit for your research. If you are not sure, read the appropriate sections before making your selection.

☒ Life sciences ☐ Behavioural & social sciences ☐ Ecological, evolutionary & environmental sciences

For a reference copy of the document with all sections, see nature.com/documents/nr-reporting-summary-flat.pdf

Life sciences study design

All studies must disclose on these points even when the disclosure is negative.

Sample size	No statistical methods were used to predetermine sample sizes and all currently available sample. We selected 228,258 samples UK Biobank with both primary care and hospital inpatient records. We selected 157,206 unrelated (defined as having less than 11 putative third-degree relatives in the kinship table) British ancestry individuals from the 228,258 samples to compute GWAS summary statistics, where we used 1,141,346 HapMap 3 SNPs. Details are stated in Methods section - UK Biobank data.
Data exclusions	We selected 228,258 samples from UK Biobank with both primary care and hospital inpatient records. We selected 157,206 unrelated

Data exclusions	(defined as having less than 11 putative third-degree relatives in the kinship table) British ancestry individuals from the 228,258 samples to compute GWAS summary statistics, where we used 1,141,346 HapMap 3 SNPs.
Replication	We performed 100 simulations to test the unbiasedness of pleiotropic shared heritability estimates in each simulation scenario. All repeated experiments were performed using Monte-Carlo simulations. In each simulation, samples were independently generated under the predefined simulation framework, so no experimental group allocation or randomization process was involved.
Randomization	This study utilized publicly available dataset UK Biobank.
Blinding	The investigators were blinded to group allocation during data collection and/or analysis.

Reporting for specific materials, systems and methods

We require information from authors about some types of materials, experimental systems and methods used in many studies. Here, indicate whether each material, system or method listed is relevant to your study. If you are not sure if a list item applies to your research, read the appropriate section before selecting a response.

Materials & experimental systems

n/a	Involved in the study
☒	☐ Antibodies
☒	☐ Eukaryotic cell lines
☒	☐ Palaeontology and archaeology
☒	☐ Animals and other organisms
☒	☐ Clinical data
☒	☐ Dual use research of concern
☒	☐ Plants

Methods

n/a	Involved in the study
☒	☐ ChIP-seq
☒	☐ Flow cytometry
☒	☐ MRI-based neuroimaging

Plants

Seed stocks	*Report on the source of all seed stocks or other plant material used. If applicable, state the seed stock centre and catalogue number. If plant specimens were collected from the field, describe the collection location, date and sampling procedures.*
Novel plant genotypes	*Describe the methods by which all novel plant genotypes were produced. This includes those generated by transgenic approaches, gene editing, chemical/radiation-based mutagenesis and hybridization. For transgenic lines, describe the transformation method, the number of independent lines analyzed and the generation upon which experiments were performed. For gene-edited lines, describe the editor used, the endogenous sequence targeted for editing, the targeting guide RNA sequence (if applicable) and how the editor was applied.*
Authentication	*Describe any authentication procedures for each seed stock used or novel genotype generated. Describe any experiments used to assess the effect of a mutation and, where applicable, how potential secondary effects (e.g. second site T-DNA insertions, mosiacism, off-target gene editing) were examined.*
