# Supplementary material for: Pleiotropic shared heritability quantifies the shared genetic variance of common diseases
Source: Nat Genet. 2026 Jun 9;58(6):1248–57. doi: 10.1038/s41588-026-02607-w (PMC13256260; doi:10.1038/s41588-026-02607-w)
Supplement: Supplementary file 3 — Peer Review File [file 41588_2026_2607_MOESM3_ESM.pdf]

nature portfolio

Peer Review File

Pleiotropic shared heritability quantifies the shared genetic variance of common diseases

Corresponding Author: Dr Xilin Jiang

Version 0:

Decision Letter:

23rd May 2025

Dear Xilin,

Thank you for inquiring about our interest in this proposed submission. It sounds interesting and we would like to see it, although I am sure you will appreciate that it can be very difficult to judge a paper without having seen it in its entirety. We cannot guarantee that we will send it out for review until we have had a chance to look at the full manuscript.

Please note that supplementary materials must not be included with the main text and figures of your manuscript. Supplementary items should be uploaded as separate files.

At this stage you will also need to upload:

1) The Editorial Policy Checklist:
https://www.nature.com/documents/nr-editorial-policy-checklist.pdf

2) The Reporting Summary:
https://www.nature.com/documents/nr-reporting-summary.pdf

(Here you can read about the role of the Reporting Summary in reproducible science:
https://www.nature.com/news/announcement-towards-greater-reproducibility-for-life-sciences-research-in-nature-1.22062)

In order to submit your complete manuscript, please use the link below and follow the prompt to “Revise NG-PI69103”:

Link Redacted

We look forward to receiving it.

Sincerely,
Kyle

Kyle Vogan, PhD
Senior Editor
Nature Genetics
https://orcid.org/0000-0001-9565-9665

Version 1:

Decision Letter:

13th August 2025

Dear Xilin,

Your Article "Pleiotropic heritability quantifies the shared genetic variance of common diseases" has now been seen by three referees. You will see from their comments below that, while they find your work of interest, they have raised several relevant points. We are interested in the possibility of publishing your study in Nature Genetics, but we would like to consider your response to these points in the form of a revised manuscript before we make a final decision on publication.

To guide the scope of the revisions, the editors discuss the referee reports in detail within the team, including with the chief editor, with a view to identifying key priorities that should be addressed in revision, and sometimes overruling referee requests that are deemed beyond the scope of the current study. In this case, we ask that you address all technical points related to the method and its implementation, extend the simulations to encompass a wider range of scenarios and use cases, and revise the terminology and framing as requested by the referees. We hope you will find this prioritized set of referee points to be useful when revising your study. Please do not hesitate to get in touch if you would like to discuss these issues further.

We therefore invite you to revise your manuscript taking into account all reviewer and editor comments. Please highlight all changes in the manuscript text file. At this stage, we will need you to upload a copy of the manuscript in MS Word .docx or similar editable format.

We are committed to providing a fair and constructive peer-review process. Do not hesitate to contact us if there are specific requests from the reviewers that you believe are technically impossible or unlikely to yield a meaningful outcome.

When revising your manuscript:

*1) Include a “Response to referees” document detailing, point-by-point, how you addressed each referee comment. If no action was taken to address a point, you must provide a compelling argument. This response will be sent back to the referees along with the revised manuscript.

*2) If you have not done so already, please begin to revise your manuscript so that it conforms to our Article format instructions, available
here.
Refer also to any guidelines provided in this letter.

*3) Include a revised version of any required Reporting Summary: https://www.nature.com/documents/nr-reporting-summary.pdf
It will be available to referees (and, potentially, statisticians) to aid in their evaluation if the manuscript goes back for peer review.
A revised checklist is essential for re-review of the paper.

Please be aware of our guidelines on digital image standards.

EXTENDED DATA FIGURES

When re-submitting your manuscript, please ensure that any supplementary figures and tables that are crucial to the manuscript's conclusions are converted into Extended Data figures and tables to increase visibility of these data. Extended Data figures and tables are online-only (present in the online PDF and full-text HTML versions of the paper), peer-reviewed display items that provide essential background to the article but are not included in the main article due to space constraints. A maximum of ten Extended Data display items (figures and tables) is permitted.

Please use the link below to submit your revised manuscript and related files:

Link Redacted

Note: This URL links to your confidential home page and associated information about manuscripts you may have submitted, or that you are reviewing for us. If you wish to forward this email to co-authors, please delete the link to your homepage.

We hope to receive your revised manuscript within 8-12 weeks. If you cannot send it within this time, please let us know.

Please do not hesitate to contact me if you have any questions or would like to discuss these revisions further.

Nature Genetics is committed to improving transparency in authorship. As part of our efforts in this direction, we are now requesting that all authors identified as ‘corresponding author’ on published papers create and link their Open Researcher and Contributor Identifier (ORCID) with their account on the Manuscript Tracking System (MTS), prior to acceptance. ORCID helps the scientific community achieve unambiguous attribution of all scholarly contributions. You can create and link your ORCID from the home page of the MTS by clicking on ‘Modify my Springer Nature account’. For more information, please visit www.springernature.com/orcid.

We look forward to seeing the revised manuscript and thank you for the opportunity to review your work.

Sincerely,
Kyle

Kyle Vogan, PhD
Senior Editor
Nature Genetics
https://orcid.org/0000-0001-9565-9665

Referee expertise:

Referee #1: Genetics, complex traits, statistical methods

Referee #2: Genetics, complex traits, statistical methods

Referee #3: Genetics, complex traits, statistical methods

Reviewers' Comments:

Reviewer #1 (Remarks to the Author):

Reviewer Overview: The current submission provides an overview, validation, and empirical application of a new method for estimating the proportion of SNP-based heritability in a target trait that overlaps with a multivariate set of external traits (i.e., pleiotropic heritability). There are several notable strengths. The manuscript is well-written, and the figures are well-designed. There is also clearly careful thought given to the method, with issues around possible bias largely resolved by their Monte-Carlo bias correction. The comparison to phenotypic correlations also yields important insights around how shared genetic and phenotypic architecture may differ. Finally, the corresponding GitHub appears well-documented, which will encourage uptake and citability of the work going forward. I have three larger comments and some minor concerns:

Major Comments:

1. I sympathize that no matter what kind of simulation you perform, the reviewers always want something more for these methods papers. The simulations are already fairly thorough, considering the effect of, for example, specifying either 100% or 5% causal SNPs in the population. With that said, there are two aspects that could be evaluated to make the simulation more informative:

a. The authors simulate at 25% heritability, yet none of the traits in ST1 have a liability scale heritability that high (average ~= 12%). I recommend simulating a wider range of heritability estimates that cover the lower range of heritabilities seen in their empirical applications to the 15 traits.

b. The authors used in-sample LD scores to estimate genetic correlations, thereby optimizing performance. Yet, the GitHub provides LD-scores from the 1000 Genomes reference panel which will be out-of-sample for empirical applications. I'd like to see simulations when using the 1000 Genomes panel to mirror the recommended pipeline outlined on their GitHub.

2. Related to the point above about adding simulations, I'm hopeful that there are some general guidelines that could be provided regarding what kinds of QC future users should apply when deciding what traits to exclude from analyses. For example, is there a minimum h2 Z-statistic for the target or auxiliary traits a user should look for? Are their minimum or maximum values of rg between target and auxiliary traits that should be considered? I realize that these guidelines are tough because it can depend on so many different aspects of someone's particular use case, but I think the simulations could be used to provide some bare minimum guidance along this front.

3. The authors discuss on line 133 (and expand in line 498 of the method) how, in the presence of unstable estimates, high genetic correlations (rg > 0.5) among the auxiliary variables can be an issue and pruning based on LDSC Z-statistics is recommended to improve stability. That makes sense, but then the authors go on to write on lines 152-153: "In all analyses, we restricted to auxiliary diseases with modest genetic correlation to the target disease ($rg < 0.5$)." The correlations amongst the auxiliary variables, as compared to the correlations between the target and auxiliary variable, seem to be two different considerations. Moreover, the decision to remove traits with rg < 0.5 with the target trait seems to me to be a critical one in terms of the conclusions you would draw from applying the method.

Minor comments:

4. The authors write on line 71 of the Introduction: "However, to our knowledge, we lack a method to estimate the genetic variance of a given target disease that is shared with other diseases and traits; such a method would inform efforts to prioritize therapeutic targets while accounting for disease comorbidities and potential drug side effects30–35." It's not clear from this sentence alone how understanding how much of the SNP-based heritability is pleiotropic would allow you to prioritize therapeutic targets. Let's say I find that MDD is broadly pleiotropic, but that genome-wide estimate doesn't give me

a biological pathway to target. The authors need to walk this claim out a bit as to how this method, in its current form, could aid in drug discovery.

5. Worth adding (line 102) that you are looking at common variant SNPs: “In this study, we restrict both pleiotropic heritability and total heritability to heritability explained by SNPs.”

6. The authors write on line 144 that they look at: “15 highly heritable target diseases (prevalence > 1% and heritability z-score > 6) from the UK Biobank”. The Z-statistic from LDSC does not tell you if the trait has high heritability (e.g., small point estimate and even smaller standard error can yield z > 6). Indeed, the heritability estimate on the observed scale in ST3 for the Alzheimer’s GWAS is < 1%, but has a Z-statistic of 8.75. I would add a line about what the minimum liability h2 was for these 15 traits and report what those liability scale heritabilities are in the main text (e.g., in a table).

7. Figure 4 shows that Type 2 Diabetes and MDD are both characterized by highly pleiotropic signal. They are also among the least heritable traits in ST1. It would be interesting to see the plot between liability scale heritability and the ratio of h2pleio/h2 to see if pleiotropy increases as liability scale h2 decreases.

8. The authors introduce the empirical application on line 144 but then talk about the simulations before the empirical results on line 156. That seems out of order; I would shift all mention of the empirical results to a single section (i.e., shift paragraph encompassing current lines 144-155 down to line 197).

9. Can the authors report the ratio of empirical standard deviations to estimated standard errors to confirm the ratio is near 1. Approximately well-calibrated is overly vague for this point (line 179). “We compared the estimated standard errors to empirical standard deviations, and determined that estimated standard errors were approximately well-calibrated.”

10. It would be helpful if the authors included some examples of what the 16 quantitative traits are in the results section and clarify how they were selected.

11. In the section comparing genetic and non-genetic variance that begins on line 296, it seems that the phenotypic correlation will also encapsulate rare variant architecture not captured by common variant estimates from LDSC. Does this affect interpretation that the difference between h2pleio and V2pleio is due solely to environmental mechanisms?

12. As presented, the reader might get the impression that the method is only applicable to binary traits: all target traits are binary in the empirical applications and the focus is on liability scale heritability throughout the manuscript. However, it’s easy to imagine use cases where a quantitative trait is the target. It would be helpful for the authors to clarify that the method could be used for both binary and quantitative targets.

Reviewer #1 (Remarks on code availability):

The code is well-documented on the dedicated GitHub for the method.

Reviewer #2 (Remarks to the Author):

This is a useful paper that introduces a new method, PHBC, to estimate “pleiotropic” heritability. This is defined as the proportion of genetic variance in a target disease that is shared with a given set of auxiliary diseases. The authors propose a well-designed method and support it with simulations and real data. Below are several comments regarding interpretation, scope, and presentation.

- The method is presented in the context of case-control traits. This raises the question: why not continuous traits? It would be helpful to clarify whether there are any real limitations to applying PHBC to continuous outcomes, or whether this was simply a choice for the current study.

- The paper frames “pleiotropic heritability” as the proportion of SNP-heritability that is pleiotropic. This is a misleading way to phrase it. In reality, it is the proportion of heritability that is shared with the included auxiliary traits. Only a limited number of traits were included, and many others are not; the results are strongly dependent on the trait set used.

- Some traits, such as T2D and MDD, reach pleiotropic heritability estimates near 1 when more auxiliary traits are added. This raises the question whether any disease might reach a similar value if enough traits are included. If so, the metric reflects the breadth and choice of the auxiliary set more than any intrinsic feature of the target trait. It might be better to frame this as a measure of genetic overlap with a specific reference set, rather than as a general statement about pleiotropy.

- Figure 5 shows that MDD has high pleiotropic overlap with endocrine and digestive disorders, while T2D overlaps strongly with mental disorders. I think these findings deserve further discussion.

- One likely explanation of the previous point, but also of the more general results, is that a substantial part of this shared genetic variance is driven by socio-economic status. SES influences many complex traits and has a genetic component itself which is correlated with many environmental circumstances. I expect that a substantial (maybe even majority?) part of the pleiotropy observed in this paper is due to SES, which tends to increase the risk for all these diseases. You can probably check this by including educational attainment in your analyses (although, there is also strong assortative mating on EA).

- Figure 3: instead of “NA” in the diagonal, consider leaving it blank or, better, displaying heritability estimates.

- Supplementary Figure 20 could easily be Figure 1B.

- Ethics approval says: “This study utilized publicly available datasets and therefore did not require ethical approval.” That’s not true, UK Biobank did get ethical approval, and cannot be used by just anyone, it’s not publicly available in that sense. UK Biobank has received ethical approval from the National Health Service North West Centre for Research Ethics Committee (reference: 11/NW/0382). If you get permission to use UKB for your study (which you should), then it falls under this ethical approval.

- The comparison between pleiotropic genetic variance and pleiotropic phenotypic variance is a very interesting result in the paper. I would be curious to hear how the authors would explain this observation.

- The bias correction for finite-sample error in genetic correlation estimates is essential and is supported by simulations. Still, the procedure remains a bit opaque. A clearer explanation of why the Monte Carlo approach works, perhaps with a small example or illustration, would help readers unfamiliar with this kind of bias adjustment.

Reviewer #3 (Remarks to the Author):

The authors proposed a method called Pleiotropic Heritability with Bias Correction (PHBC) to estimate pleiotropic heritability. Simulations suggested that PHBC is robust. Applying the method to the UK Biobank, they found pleiotropic heritability to be broadly distributed across disease categories. However, a major concern is that the bias-correction approach appears somewhat ad hoc, and it is unclear whether the method performs well when disease heritability is low (h^2 = 0.02–0.05), which is typical for many diseases. It is also uncertain whether the reported conclusions reflect genuine biological signals or are driven by bias.

Major comments:

1. The definition of h2pleio is unclear. There are different types of pleiotropy (see their ref. #2), and it is not evident what type of pleiotropic variation h2pleio is intended to capture. Based on the formula provided, h2pleio represents the proportion of SNP heritability of a target disease that is jointly explained by a specific set of auxiliary diseases, which could arise from true pleiotropy or from spurious pleiotropy. In the simulation models, the authors assumed an infinitesimal model, meaning that all genetic variants are pleiotropic. Under this assumption, the genetic variation from pleiotropic variants would be identical to the SNP heritability. Therefore, defining h2pleio as “pleiotropic heritability” could be misleading. A different terminology seems more reasonable.

2. It is unclear what new insights can be gained from estimating h2pleio over the genetic correlation. It is well established that many diseases share common risk factors and biological pathways, and therefore share heritability, which can also be represented through genetic correlation. As the authors demonstrated, including additional diseases can substantially alter the h2pleio/h2 estimate. Consequently, the magnitude of h2pleio/h2 is highly dependent on the number and selection of diseases or traits included in the analysis.

3. The proposed bias correction for estimating h2pleio/h2 relies on a strong assumption that the true value is simply a scaled version of the initial estimate. It is unclear why this relationship should hold in general, or why the bias would necessarily be upward. In the simulations, only the infinitesimal model was considered. Could the observed upward bias be a consequence of this model choice? If the infinitesimal model does not hold, it is uncertain whether the same bias pattern or the validity of the correction would persist.

4. The simulations were conducted assuming a liability-scale heritability of 0.25, whereas disease heritability is typically much lower (h2<0.13; for more than half of the 15 diseases in Supplemental Table 1, h2<0.05). Therefore, it is not convincing that the good performance of PHBC in simulations will necessarily translate to real data, as the bias in h2pleio/h2 could be inflated when h2 is small.

5. The claim that estimates of h2pleio/h2 were generally larger than those of V2pleio/V2 may also be explained by the bias in estimating h2pleio/h2 when h2 is small. For example, in hypertension (HTN), which has the largest h2 among the 15 diseases, the estimate of h2pleio/h2 was much closer to the estimate of V2pleio/V2 (Figure 7).

6. In the GWAS analysis, LD prune at r2 <0.8 was applied. Will the estimate of h2pleio/h2 also sensitive to this LD pruning?

Version 2:

Decision Letter:

8th December 2025

Dear Xilin,

Your revised Article "Pleiotropic heritability quantifies the shared genetic variance of common diseases" has been seen by the original referees. You will see from their comments below that, while they find the revision improved, they have raised a few ongoing concerns. We remain interested in the possibility of publishing your study in Nature Genetics, but we would like to consider your response to these ongoing concerns in the form of a further revision before we make a final decision on publication.

We therefore invite you to further revise your manuscript taking into account all reviewer comments. Please highlight all changes in the manuscript text file. At this stage, we will need you to upload a copy of the manuscript in MS Word .docx or similar editable format.

We are committed to providing a fair and constructive peer-review process. Do not hesitate to contact us if there are specific requests from the reviewers that you believe are technically impossible or unlikely to yield a meaningful outcome.

When revising your manuscript:

*1) Include a “Response to referees” document detailing, point-by-point, how you addressed each referee comment. If no action was taken to address a point, you must provide a compelling argument. This response will be sent back to the referees along with the revised manuscript.

*2) If you have not done so already, please begin to revise your manuscript so that it conforms to our Article format instructions, available
here.
Refer also to any guidelines provided in this letter.

*3) Include a revised version of your Reporting Summary: https://www.nature.com/documents/nr-reporting-summary.pdf
It will be available to referees (and, potentially, statisticians) to aid in their evaluation if the manuscript goes back for peer review.
A revised checklist is essential for re-review of the paper.

Please be aware of our guidelines on digital image standards.

EXTENDED DATA FIGURES

When re-submitting your manuscript, please ensure that any supplementary figures and tables that are crucial to the manuscript’s conclusions are converted into Extended Data figures and tables to increase visibility of these data. Extended Data figures and tables are online-only (present in the online PDF and full-text HTML versions of the paper), peer-reviewed display items that provide essential background to the article but are not included in the main article due to space constraints. A maximum of ten Extended Data display items (figures and tables) is permitted.

Please use the link below to submit your revised manuscript and related files:

Link Redacted

Note: This URL links to your confidential home page and associated information about manuscripts you may have submitted, or that you are reviewing for us. If you wish to forward this email to co-authors, please delete the link to your homepage.

We hope to receive your revised manuscript within 8-12 weeks. If you cannot send it within this time, please let us know.

Please do not hesitate to contact me if you have any questions or would like to discuss these revisions further.

Nature Genetics is committed to improving transparency in authorship. As part of our efforts in this direction, we are now requesting that all authors identified as ‘corresponding author’ on published papers create and link their Open Researcher and Contributor Identifier (ORCID) with their account on the Manuscript Tracking System (MTS), prior to acceptance. ORCID helps the scientific community achieve unambiguous attribution of all scholarly contributions. You can create and link your ORCID from the home page of the MTS by clicking on ‘Modify my Springer Nature account’. For more information, please visit www.springernature.com/orcid.

We look forward to seeing the revised manuscript and thank you for the opportunity to review your work.

Sincerely,
Kyle

Kyle Vogan, PhD
Senior Editor

Nature Genetics
https://orcid.org/0000-0001-9565-9665

Referee expertise:

Referee #1: Genetics, complex traits, statistical methods

Referee #2: Genetics, complex traits, statistical methods

Referee #3: Genetics, complex traits, statistical methods

Reviewers' Comments:

Reviewer #1 (Remarks to the Author):

The authors have been very responsive in their resubmission, with the manuscript including updated simulations that reflect more realistic population generating scenarios and use out-of-sample LD, newly added guidelines around trait power and genetic overlap, and a built-in R package. I very much appreciate their detailed and clear responses. I have one remaining, but important, comment.

The previous version of the manuscript described the standard errors as approximately well-calibrated. The resubmission has been updated to clarify that the simulations reveal the standard errors on the Monte-Carlo corrected estimates may be upwardly biased by a ratio of 1.63. This, I think, is exceedingly high and is better described as the standard errors not being appropriately calibrated. To be clear, I recognize that this ratio is never exactly 1, but in my own work and when reviewing other methods manuscripts that report this value, my take is that a reasonable upper limit on this estimate is ~1.1.

As the authors state in the note of Supplementary Figure 1, comparison of Panels A and B shows that the SEs from the block jackknife are anti-conservative before correction and conservative after applying the Monte-Carlo bias correction. The upward bias in SE after the correction seems to be largely driven by two scenarios that are much further off the dashed line. Is it possible something strange happened with that set of specific simulation runs?

I also note that they compare the squared block jackknife standard errors to the squared deviation (squared difference between post-corrected and true value). In my experience, the standard errors are typically compared to the standard deviation of the sampling distribution of estimates obtained from simulation (not the deviation from the true value), which may produce a ratio more in-line with what I'm used to seeing? I don't need the authors to address my speculations about what may be going on (I pose them here only in case useful), but I do ask that more is done to figure out why the standard errors currently appear to be exceedingly conservative as this is obviously critical to the method.

Reviewer #1 (Remarks on code availability):

The code is well-documented.

Reviewer #2 (Remarks to the Author):

I am happy with the way the authors have addressed my previous comments. The revisions improve conceptual clarity, especially regarding the dependence of pleiotropic heritability on the chosen auxiliary trait set, and the expanded simulations and clarifications strengthen the methodological presentation. Overall, this is a valuable and timely contribution to the literature on multivariate genetic architectures.

I have only one remaining suggestion. In the current version, the manuscript describes the increase in h_{pleio}^2/h^2as "plateauing" when more auxiliary traits are added. Figure 6 does show diminishing returns, but the estimates still rise with each expansion of the auxiliary set, and the current collection includes only a subset of potentially relevant complex traits. I would therefore suggest softening the language from "plateaus" to something along the lines of "shows diminishing increases," and to note explicitly that further increases are possible if additional (and more diverse) traits are incorporated. This would better reflect the empirical pattern without overstating the convergence.

Beyond this minor point, I am satisfied with the revision.

Reviewer #3 (Remarks to the Author):

The revised manuscript has improved significantly. However, I still have some concerns I would like to authors to address.

1. I appreciate that the authors now provide a much clearer definition of h_{pleio}^2 and its interpretation. However, I remain concerned that referring to h_{pleio}^2 as "pleiotropic heritability" may be misleading. As the authors themselves note, $h_{pleio}^2 = 0$ does not imply the absence of pleiotropic variants. Rather, numerous variants may still influence multiple

disease traits. However, I do not have a better name.

2. I previously raised concerns about the proposed bias-correction approach, particularly the strong assumption that the true value is a scaled version of the initial estimate. This concern remains unresolved. I am not opposed to the use of Monte Carlo sampling; rather, I question whether this scaling strategy can truly yield an unbiased estimate of (h_pleio^2)/h^2. Although I agree with the authors that the post bias correction estimates show reduced absolute bias, as demonstrated in ST4–10, the remaining bias is still substantial. For example, when testing whether there is no zero pleiotropic heritability in the zero_trait simulations, ST4–10 shows a markedly inflated type I error rate.

3. Since UK Biobank has been reported of substantial population structure, it is not clear whether the estimate of (h_pleio^2)/h^2 also included the effect by population structure.

4. It appears that the results in the revised manuscript differ substantially from those in the previous submission. For example, the current manuscript reports that h_pleio^2/h^2 is 1.51× larger than the proportion of liability-scale total phenotypic variance explained by the same set of auxiliary diseases, compared with 2.4× in the earlier version. Although the authors attribute some of these changes to phenotype misclassification, the magnitude of the differences seems quite large.

Version 3:

Decision Letter:

Our ref: NG-A69103R2

24th March 2026

Dear Xilin,

Your revised manuscript "Pleiotropic shared heritability quantifies the shared genetic variance of common diseases" (NG-A69103R2) has been seen by Reviewers #1 and #3. As you will see from their comments below, they are satisfied with the revision and have no further requests, and therefore we will be happy in principle to publish your study in Nature Genetics as an Article pending final revisions to comply with our editorial and formatting guidelines.

We are now performing detailed checks on your paper, and we will send you a checklist detailing our editorial and formatting requirements soon. Please do not upload the final materials or make any revisions until you receive this additional information from us.

Thank you again for your interest in Nature Genetics. Please do not hesitate to contact me if you have any questions.

Sincerely,
Kyle

Kyle Vogan, PhD
Senior Editor
Nature Genetics
https://orcid.org/0000-0001-9565-9665

Reviewer #1 (Remarks to the Author):

In this second round revisions the authors corrected a prior bug in the code for evaluating calibration of SEs in their simulations and furthermore devised a new method for calculating SEs that produces a ratio between empirical and observed SEs that are near 1. In summary, they have been very responsive to my remaining concern and I really do appreciate the authors taking this final concern seriously, even though it was part of stage 2 revision.

Reviewer #1 (Remarks on code availability):

The code is well documented.

Reviewer #3 (Remarks to the Author):

I have no further questions.

Open Access This Peer Review File is licensed under a Creative Commons Attribution 4.0 International License, which permits use, sharing, adaptation, distribution and reproduction in any medium or format, as long as you give appropriate credit to the original author(s) and the source, provide a link to the Creative Commons license, and indicate if changes were made.
In cases where reviewers are anonymous, credit should be given to 'Anonymous Referee' and the source.
The images or other third party material in this Peer Review File are included in the article’s Creative Commons license, unless indicated otherwise in a credit line to the material. If material is not included in the article’s Creative Commons license and your intended use is not permitted by statutory regulation or exceeds the permitted use, you will need to obtain permission directly from the copyright holder.
To view a copy of this license, visit https://creativecommons.org/licenses/by/4.0/

Response to reviewers for NG-A69103R (Zhao et al.)

Overview of main changes to manuscript

We thank all three reviewers for their helpful suggestions. Here, we provide a brief overview of main changes to the manuscript; responses to specific reviewer comments are provided below.

1. Updated SNP set. We now analyze 1.1M UK Biobank HapMap3 SNPs with MAF > 0.01 in both simulations and real data analyses (whereas we previously analyzed LD-pruned SNPs in both simulations and real data analyses). See response to Reviewer #3 Comment 6.

2. Changes to simulation framework. We made several changes to our simulation framework:
(i) We now use reference LD from 1000 Genomes Europeans to estimate genetic correlations, as in our real data analyses. See response to Reviewer #1 Comment 1b.
(ii) We now set liability-scale heritability to 0.13 (median across the 15 UK Biobank diseases) and prevalence to 0.10 (median across the 15 UK Biobank diseases) in our main simulations. See response to Reviewer #1 Comment 1a, part (ii) of response to Reviewer #3 Introductory comments, and response to Reviewer #3 Comment 4.
(iii) We now set the proportion of causal SNPs to 5% in our main simulations, thus assuming a non-infinitesimal model. See part (ii) of response to Reviewer #3 Comment 1 and 3.

3. Update to UK Biobank disease phenotypes and summary statistics. We corrected an error in mapping ICD-10 records to the PheCode system; we regret the error. As a result, we relabeled some samples from controls to cases (1.7% of samples on average across 15 diseases; 4.7% of samples for T2D). We recomputed summary statistics accordingly.

4. Update to PHBC software. We have updated our software by including a built-in R package, which is available on CRAN after passing appropriate checks (https://cran.r-project.org/web/packages/pleioh2g). See response to Reviewer #1 Introductory comments. We have also updated our software to automatically remove auxiliary diseases with ${r_g}^2 > 0.5$ to the target diseases (and to report the excluded auxiliary diseases) See response to Reviewer #1 comment 2.

In simulation analyses, the updated SNP set (#1) and changes to simulation framework (#2) had little impact on our results, and our qualitative conclusions have not changed.

In empirical data analyses, the updated SNP set (#1) and update to UK Biobank disease phenotypes and summary statistics (#3) impacted some of our point estimates, but our qualitative conclusions have not changed. In particular, our estimate of average $h^2{}_{pleio}/h^2$ for 15 UK Biobank diseases (using the same diseases as auxiliary diseases) changed from 0.34 (s.e. 0.07) to 0.27 (s.e. 0.02) (P=0.32 for difference), and our estimate of the average ratio of $h^2{}_{pleio}/h^2$ to $V^2{}_{pleio}/V^2$ changed from 2.40 (s.e. 0.52) to 1.51x (s.e. 0.14) (P=0.07 for difference).

Reviewer #1:

Reviewer Overview: The current submission provides an overview, validation, and empirical application of a new method for estimating the proportion of SNP-based heritability in a target trait that overlaps with a multivariate set of external traits (i.e., pleiotropic heritability). There are several notable strengths. The manuscript is well-written, and the figures are well-designed. There is also clearly careful thought given to the method, with issues around possible bias largely resolved by their Monte-Carlo bias correction. The comparison to phenotypic correlations also yields important insights around how shared genetic and phenotypic architecture may differ. Finally, the corresponding GitHub appears well-documented, which will encourage uptake and citability of the work going forward. I have three larger comments and some minor concerns:

We thank the reviewer for suggesting that there are several notable strengths. We have updated our software by including a built-in R package (pleioh2g), which is available on CRAN after passing appropriate checks (https://cran.r-project.org/web/packages/pleioh2g) and we believe this will facilitate its use. We have updated the *Code Availability* section (p.12) accordingly. The reviewer's comments are addressed in detail below.

Major Comments:

1. I sympathize that no matter what kind of simulation you perform, the reviewers always want something more for these methods papers. The simulations are already fairly thorough, considering the effect of, for example, specifying either 100% or 5% causal SNPs in the population. With that said, there are two aspects that could be evaluated to make the simulation more informative:

a. The authors simulate at 25% heritability, yet none of the traits in ST1 have a liability scale heritability that high (average ~= 12%). I recommend simulating a wider range of heritability estimates that cover the lower range of heritabilities seen in their empirical applications to the 15 traits.

(Also see part (ii) of response to Reviewer #3 Introductory comments and response to Reviewer #3 Comment 4)
We agree that a liability-scale heritability of 0.25 is not reflective of our real data analyses. We have updated the liability-scale heritability in main simulations to 0.13, which is the median across 15 UK Biobank diseases (we also consider values 0.06 and 0.25 in secondary simulations). We have also updated the prevalence in main simulations to 0.10, which is the median across 15 UK Biobank diseases (we also consider value of 0.05 in secondary simulations). We have updated Figure 2, Supplementary Figures 1,6-11 and Supplementary Tables 4, 8-10 accordingly. We have updated the *Simulations* subsection of the Results section (p.6, citing Figure 2) and the *Simulations* subsection of the Methods section (p.20) to reflect these changes. These changes did not significantly impact the conclusions of our simulations.

b. The authors used in-sample LD scores to estimate genetic correlations, thereby optimizing performance. Yet, the GitHub provides LD-scores from the 1000 Genomes reference panel which will be out-of-sample for empirical applications. I'd like to see simulations when using the

1000 Genomes panel to mirror the recommended pipeline outlined on their GitHub.

We agree that in-sample LD is not reflective of our real data analyses, and have updated our simulations to use reference LD from 1000 Genomes Europeans to estimate genetic correlations. We have updated Figure 2, Supplementary Figures 1-13 and Supplementary Tables 4-12 accordingly. We have updated the *Simulations* subsection of the Results section (p.6, citing Figure 2) and the *Simulations* subsection of the Methods section (p.21) to reflect this change. This change did not significantly impact the conclusions of our simulations.

2. Related to the point above about adding simulations, I'm hopeful that there are some general guidelines that could be provided regarding what kinds of QC future users should apply when deciding what traits to exclude from analyses. For example, is there a minimum h2 Z-statistic for the target or auxiliary traits a user should look for? Are their minimum or maximum values of rg between target and auxiliary traits that should be considered? I realize that these guidelines are tough because it can depend on so many different aspects of someone's particular use case, but I think the simulations could be used to provide some bare minimum guidance along this front.

We agree that it is appropriate to provide guidance on the minimum heritability z-score for target and auxiliary diseases and maximum genetic correlation between target and auxiliary disease.

For the heritability z-score for target and auxiliary diseases, we recommend heritability z-score > 6, based on our previous work (Finucane et al., 2015 *Nat Genet*, ref. 49; Bulik-Sullivan*, Finucane* et al. 2015 *Nat Genet*; ref. 8); we note that generative model parameters for main and secondary simulations were chosen based on median values of prevalence, heritability, and heritability z-score for the real traits that we analyzed. We have updated the *Overview of estimation of pleiotropic heritability* subsection of the Results section (p.5-6) and the *Simulations* subsection of the Methods section (p.20) accordingly.

For the genetic correlation between target and auxiliary diseases, we recommend squared genetic correlation (${r_g}^2$) < 0.5 (also see response to Reviewer #1 Comment 3). We have updated the *Overview of estimation of pleiotropic heritability* subsection of the Results section (p.5-6) and the *Application to 15 diseases from the UK Biobank* subsection of the Results section (p.8-9) to discuss this choice.

We have also updated the online README.md file in our PHBC software package (https://github.com/yjzhao1004/pleioh2g) to discuss these recommendations, and updated our software to remove auxiliary diseases with ${r_g}^2$ > 0.5 to the target diseases (and report the excluded auxiliary disease).

3. The authors discuss on line 133 (and expand in line 498 of the method) how, in the presence of unstable estimates, high genetic correlations (rg > 0.5) among the auxiliary variables can be an issue and pruning based on LDSC Z-statistics is recommended to improve stability. That makes sense, but then the authors go on to write on lines 152-153: "In all analyses, we restricted to auxiliary diseases with modest genetic correlation to the target disease (rg < 0.5)." The correlations amongst the auxiliary variables, as compared to the correlations between the target and auxiliary variable, seem to be two different considerations. Moreover, the decision to remove

traits with rg < 0.5 with the target trait seems to me to be a critical one in terms of the conclusions you would draw from applying the method.

We agree that (i) maximum squared genetic correlation (${r_g}^2$) between auxiliary diseases and (ii) maximum squared genetic correlation (${r_g}^2$) between target and auxiliary diseases are two different choices. We discuss each of these choices in turn.

(i) Maximum squared genetic correlation (${r_g}^2$) between auxiliary diseases

The maximum ${r_g}^2$ between auxiliary diseases is imposed to ensure numerical stability when estimating ${h^2}_{pleio}/h^2$. When an iterative pruning procedure is necessary due to numerical instability, we prune auxiliary disease pairs with ${r_g}^2 > 0.5$ (keeping the auxiliary disease with higher r_g z-score with the target disease) and iteratively reduce this threshold. We have updated the *Overview of estimation of pleiotropic heritability* subsection of the Results section (p.5-6) to clarify these points, emphasizing that this choice is distinct from the maximum ${r_g}^2$ between target and auxiliary diseases.

(ii) Maximum squared genetic correlation (${r_g}^2$) between target and auxiliary diseases
(also see response to Reviewer #1 Comment 2)

We recommend setting this maximum to ${r_g}^2 < 0.5$. We agree that this can be a critical choice in interpreting the conclusions drawn, as the estimand can depend strongly on this choice. We have updated the *Overview of estimation of pleiotropic heritability* subsection of the Results section (p.6) to discuss this point.

To investigate this further, we performed secondary analyses to estimate the average ${h^2}_{pleio}/h^2$ across 15 UK Biobank diseases at various ${r_g}^2$ thresholds (Supplementary Figure 20). Average ${h^2}_{pleio}/h^2$ increased at ${r_g}^2$ thresholds larger than 0.5, but we consider this increase to be not biologically meaningful because most instances of ${r_g}^2 > 0.5$ involved auxiliary traits that *are not biologically distinct* from the target trait, e.g. hypertension-diastolic blood pressure (${r_g}^2 = 0.72$), hypertension-systolic blood pressure (${r_g}^2 = 0.72$), type 2 diabetes-glucose (${r_g}^2 = 0.69$), type 2 diabetes-HbA1C (${r_g}^2 = 0.79$). In addition, average ${h^2}_{pleio}/h^2$ decreased at ${r_g}^2$ thresholds smaller than 0.5, but all instances of $0.25 < {r_g}^2 < 0.5$ involved auxiliary traits that *are biologically distinct* from the target trait: coronary atherosclerosis-hypertension (${r_g}^2 = 0.25$), coronary atherosclerosis-type 2 diabetes (${r_g}^2 = 0.25$), hypertension-type 2 diabetes (${r_g}^2 = 0.26$), depression-tobacco use disorder (${r_g}^2 = 0.35$), gastroesophageal reflux disease (GERD)-tobacco use disorder (${r_g}^2 = 0.27$), obesity-type 2 diabetes (${r_g}^2 = 0.36$), obesity-respiratory diseases (${r_g}^2 = 0.29$), obesity- HbA1c (${r_g}^2 = 0.26$), obesity-glucose (${r_g}^2 = 0.26$), type 2 diabetes-body WHR (${r_g}^2 = 0.36$), type 2 diabetes-BMI (${r_g}^2 = 0.34$), type 2 diabetes-triglycerides (${r_g}^2 = 0.31$), gastroesophageal reflux disease (GERD)-depression (${r_g}^2 = 0.40$), gastroesophageal reflux disease (GERD)-respiratory diseases (${r_g}^2 = 0.39$) and depression-respiratory diseases (${r_g}^2 = 0.32$)—such that we believe that an estimand based on an ${r_g}^2$ threshold of 0.5 is preferred. We have updated the *Application to 15 diseases from the UK Biobank* subsection of the Results

section (p.9, citing new Supplementary Figure 20) to report these findings and discuss this choice.

Minor comments:

4. The authors write on line 71 of the Introduction: “However, to our knowledge, we lack a method to estimate the genetic variance of a given target disease that is shared with other diseases and traits; such a method would inform efforts to prioritize therapeutic targets while accounting for disease comorbidities and potential drug side effects30–35.” It’s not clear from this sentence alone how understanding how much of the SNP-based heritability is pleiotropic would allow you to prioritize therapeutic targets. Let’s say I find that MDD is broadly pleiotropic, but that genome-wide estimate doesn’t give me a biological pathway to target. The authors need to walk this claim out a bit as to how this method, in its current form, could aid in drug discovery.

We thank the reviewer for pointing out that estimating pleiotropic heritability cannot directly prioritize therapeutic targets. We have updated the Introduction section (p.3) to delete this statement.

5. Worth adding (line 102) that you are looking at common variant SNPs: “In this study, we restrict both pleiotropic heritability and total heritability to heritability explained by SNPs.”

We have updated the *Definition of pleiotropic heritability* subsection of the Results section (p.4) to clarify that in this study we restrict to heritability explained by common SNPs.

6. The authors write on line 144 that they look at: “15 highly heritable target diseases (prevalence > 1% and heritability z-score > 6) from the UK Biobank”. The Z-statistic from LDSC does not tell you if the trait has high heritability (e.g., small point estimate and even smaller standard error can yield z > 6). Indeed, the heritability estimate on the observed scale in ST3 for the Alzheimer’s GWAS is < 1%, but has a Z-statistic of 8.75. I would add a line about what the minimum liability h2 was for these 15 traits and report what those liability scale heritabilities are in the main text (e.g., in a table).

We have added a sentence to the *Overview of estimation of pleiotropic heritability* (p.5) and *Application to 15 diseases from the UK Biobank* subsection of the Results section (p.7) to report the range of liability-scale heritabilities for the 15 UK Biobank diseases (0.061-0.21). We have added a new Table 1, cited on p.5 and 7, listing the PheCode disease category, UK Biobank prevalence, observed-scale heritability, liability-scale heritability, and heritability z-score of the 15 UK Biobank diseases.

We have also added a sentence to the *Application to 30 diseases from publicly available GWAS meta-analyses* subsection of the Results section (p.9) to report the range of observed-scale heritabilities under case-control ascertainment for the 30 diseases from publicly available GWAS meta-analyses (0.007-0.821) as we lack disease prevalences to compute liability-scale heritability.

7. Figure 4 shows that Type 2 Diabetes and MDD are both characterized by highly pleiotropic signal. They are also among the least heritable traits in ST1. It would be interesting to see the plot between liability scale heritability and the ratio of h2pleio/h2 to see if pleiotropy increases as liability scale h2 decreases.

(Also see part (i) of response to Reviewer #3 Comment 5)
We agree that it is of interest to assess whether there is a correlation between liability-scale heritability and ${h^2}_{pleio}/h^2$ across the 15 UK Biobank diseases. We observed no significant correlation (correlation = -0.18 (P = 0.52); Supplementary Figure 19). We have updated the *Application to 15 diseases from the UK Biobank* subsection of the Results section (p.8-9, citing Supplementary Figure 19) to report this result.

8. The authors introduce the empirical application on line 144 but then talk about the simulations before the empirical results on line 156. That seems out of order; I would shift all mention of the empirical results to a single section (i.e., shift paragraph encompassing current lines 144-155 down to line 197).

We agree that simulation results should appear before empirical results. In addition, unless reviewers and/or editors have a strong preference, it is generally our preferred style to include an overview of the empirical data analyzed in the overview of methods subsection of the Results section (which in our previous manuscript was titled *Estimation of pleiotropic heritability*, to distinguish it from the preceding *Definition of pleiotropic heritability* subsection). In addition, we recognize that it is our responsibility to distinguish between overview content and results content. With this in mind, we have made three changes:

First, we have changed the title of the subsection to *Overview of estimation of pleiotropic heritability* (p.4).

Second, we have added the following text to the beginning of the paragraph: "An overview of empirical data analyzed is as follows" (*Overview of estimation of pleiotropic heritability* subsection of the Results section, p.5).

Third, we have changed "We first estimated ${h^2}_{pleio}/h^2$ for …" to "We first analyzed …" (*Overview of estimation of pleiotropic heritability* subsection of the Results section, p.5).

9. Can the authors report the ratio of empirical standard deviations to estimated standard errors to confirm the ratio is near 1. Approximately well-calibrated is overly vague for this point (line 179). "We compared the estimated standard errors to empirical standard deviations, and determined that estimated standard errors were approximately well-calibrated."

We have updated the *Simulations* subsection of the Results section (p.6, citing Supplementary Figure 1) to report that the ratio of the average estimated squared jackknife standard error to the average empirical squared deviation equal to 1.63, implying conservative calibration.

10. It would be helpful if the authors included some examples of what the 16 quantitative traits

are in the results section and clarify how they were selected.

We thank the reviewer for this suggestion. We have updated the *Overview of estimation of pleiotropic heritability* subsection of the Results section (p.5-6) to provide examples of the selected quantitative traits (including blood biochemistry measurements, e.g. total cholesterol, blood pressure and HbA1c, and demographic traits, e.g. height and BMI) and clarify that they were selected for relevance to disease based on Weissbrod et al. 2020 *Nat Genet*, ref. 34 and Jiang et al. 2025 *medRxiv*, ref. 35. We also included years of education (see response to Reviewer #2 Comment 5).

11. In the section comparing genetic and non-genetic variance that begins on line 296, it seems that the phenotypic correlation will also encapsulate rare variant architecture not captured by common variant estimates from LDSC. Does this affect interpretation that the difference between h2pleio and V2pleio is due solely to environmental mechanisms?

We agree that the difference between ${h^2}_{pleio}$ and ${V^2}_{pleio}$ also includes heritability not captured by common SNPs, such as rare variant heritability—although we expect that environmental variance will generally be larger than rare variant heritability, which is generally relatively small (Zeng et al. 2018 *Nat Genet*, ref. 40; Gazal et al. 2018 *Nat Genet*, ref. 41; Schoech et al. 2019 *Nat Commun*, ref. 42). We have updated the *Comparing shared genetic and non-genetic variance* subsection of the Results section (p.11) and the *Pleiotropic phenotypic variance* subsection of the Methods section (p.20) to clarify these points.

12. As presented, the reader might get the impression that the method is only applicable to binary traits: all target traits are binary in the empirical applications and the focus is on liability scale heritability throughout the manuscript. However, it's easy to imagine use cases where a quantitative trait is the target. It would be helpful for the authors to clarify that the method could be used for both binary and quantitative targets.

We thank the reviewer for this suggestion. We have updated the *Overview of estimation of pleiotropic heritability* subsection of the Results section (p.6) to clarify that the definition and estimation of pleiotropic heritability can be also applied to target quantitative traits.

Reviewer #1 (Remarks on code availability):

The code is well-documented on the dedicated GitHub for the method.

We thank the reviewer for suggesting that the code is well-documented.

Reviewer #2 (Remarks to the Author):

This is a useful paper that introduces a new method, PHBC, to estimate "pleiotropic" heritability. This is defined as the proportion of genetic variance in a target disease that is shared with a given set of auxiliary diseases. The authors propose a well-designed method and support it with simulations and real data. Below are several comments regarding interpretation, scope, and presentation.

We thank the reviewer for suggesting that the method is well-designed and supported by simulations and real data. The reviewer's comments are addressed in detail below.

1. The method is presented in the context of case-control traits. This raises the question: why not continuous traits? It would be helpful to clarify whether there are any real limitations to applying PHBC to continuous outcomes, or whether this was simply a choice for the current study.

We thank the reviewer for this suggestion. We have updated the *Overview of estimation of pleiotropic heritability* subsection of the Results section (p.6) to clarify that, while we elected to focus here on target diseases, the definition and estimation of pleiotropic heritability can be also applied to target quantitative traits.

2. The paper frames "pleiotropic heritability" as the proportion of SNP-heritability that is pleiotropic. This is a misleading way to phrase it. In reality, it is the proportion of heritability that is shared with the included auxiliary traits. Only a limited number of traits were included, and many others are not; the results are strongly dependent on the trait set used.

(Also see response to Reviewer #2 Comment 3 and part (ii) of response to Reviewer #3 Comment 2)
We agree with the reviewer that the definition of pleiotropic heritability is with respect to a specific set of auxiliary traits, and that the results are strongly dependent on the set of auxiliary traits. We recognize that it is our responsibility to provide a clear exposition. We have updated the *Definition of pleiotropic heritability* subsection of the Results section (p. 3) and the *Definition of* ${h^2}_{pleio}$ subsection of the Methods section (p.14) to clarify these points.

We have also updated the *Application to 30 diseases from publicly available GWAS meta-analyses* subsection of the Results section (p.9-10) to discuss in more detail how pleiotropic heritability increased with the inclusion of additional auxiliary traits. In particular, we note that average ${h^2}_{pleio}/h^2$, e.g. across 30 non-UK Biobank diseases, tends to increase and then plateau as the number of auxiliary traits increases (e.g. 35% (s.e. 3%) for all 45 auxiliary diseases to 37% (s.e. 2%) for all 62 auxiliary diseases and quantitative traits) (Fig 6B).

3. Some traits, such as T2D and MDD, reach pleiotropic heritability estimates near 1 when more auxiliary traits are added. This raises the question whether any disease might reach a similar value if enough traits are included. If so, the metric reflects the breadth and choice of the auxiliary set more than any intrinsic feature of the target trait. It might be better to frame this as a measure of genetic overlap with a specific reference set, rather than as a general statement about

pleiotropy.

(Also see response to Reviewer #2 Comment 2 and part (ii) of response to Reviewer #3 Comment 2)
We agree with the reviewer that the definition of pleiotropic heritability is with respect to a specific set of auxiliary traits, and that the results are strongly dependent on the set of auxiliary traits. We recognize that it is our responsibility to provide a clear exposition. We have updated the *Definition of pleiotropic heritability* subsection of the Results section (p.3) and the *Definition of* $h^2{}_{pleio}$ subsection of the Methods section (p.14) to clarify these points.

We have also updated the *Application to 30 diseases from publicly available GWAS meta-analyses* subsection of the Results section (p.9-10) to discuss in more detail how pleiotropic heritability increased with the inclusion of additional auxiliary traits. In particular, we note that average $h^2{}_{pleio}/h^2$, e.g. across 30 non-UK Biobank diseases, tends to increase and then plateau as the number of auxiliary traits increases (e.g. 35% (s.e. 3%) for all 45 auxiliary diseases to 37% (s.e. 2%) for all 62 auxiliary diseases and quantitative traits) (Fig 6B).

The reviewer has asked whether other diseases might reach the $h^2{}_{pleio}/h^2$ of T2D and MDD (now 59% (s.e. 4%) and 74% (s.e. 7%), using 62 auxiliary diseases and quantitative traits) if enough auxiliary traits are included. Although $h^2{}_{pleio}/h^2$ increases as more auxiliary traits are added (Figure 6A and Figure 6B), the magnitude of the increase plateaus as the number of auxiliary traits grows. Thus, we believe that expanding the set of auxiliary traits is unlikely to increase the $h^2{}_{pleio}/h^2$ to the level of T2D and MDD for most other diseases. We have updated the Discussion section (p.12) to discuss this point.

4. Figure 5 shows that MDD has high pleiotropic overlap with endocrine and digestive disorders, while T2D overlaps strongly with mental disorders. I think these findings deserve further discussion.

We thank the reviewer for the suggestion. As noted in Overview of main changes to manuscript (p.1 of this document), some of our point estimates have changed, although the differences were not statistically significant. In our updated results, MDD has high pleiotropic overlap with digestive diseases, while T2D has high overlap with circulatory system diseases. (Differences in estimates of $h^2{}_{pleio}/h^2$ for MDD explained by endocrine diseases and for T2D explained by mental disorders, compared to our previous manuscript, were not statistically significant: $P >$ 0.05/105 after correcting for 105 hypotheses tested (15 target diseases × 7 disease categories).) We have updated the *Application to 15 diseases from the UK Biobank* in Results section (p.7) to discuss the pleiotropic overlap of MDD with digestive diseases, and the pleiotropic overlap of T2D with circulatory system diseases.

5. One likely explanation of the previous point, but also of the more general results, is that a substantial part of this shared genetic variance is driven by socio-economic status. SES influences many complex traits and has a genetic component itself which is correlated with many environmental circumstances. I expect that a substantial (maybe even majority?) part of the pleiotropy observed in this paper is due to SES, which tends to increase the risk for all these diseases. You can probably check this by including educational attainment in your analyses

(although, there is also strong assortative mating on EA).

We agree that SES may contribute substantially to pleiotropy, and agree that it is of interest to include educational attainment (EA) in our analyses. We have thus made 3 changes to the paper.

First, we added EA (years of education) to the set of 16 (now 17) auxiliary quantitative traits. This change impacted main Figures 4,6 and Supplementary Figures 15, 16.

Second, we estimated $h^2{}_{pleio}/h^2$ with respect to EA as the only auxiliary trait for each of the 15 UK Biobank diseases. We determined that EA had a substantial contribution, with an average of 9.9% (s.e. 0.8%). We have updated the *Application to 15 diseases from the UK Biobank* subsection of the Results section (p.8), citing new Supplementary Figure 18 and Supplementary Table 28, to report this result.

Third, we assessed the impact of removing EA from the set of auxiliary traits by estimating the difference between (i) $h^2{}_{pleio}/h^2$ with respect to 15 UK Biobank auxiliary diseases + 17 UK Biobank auxiliary quantitative traits vs. (ii) $h^2{}_{pleio}/h^2$ with respect to 15 UK Biobank auxiliary diseases + 16 UK Biobank quantitative traits excluding EA. We determined that differences were small, with an average reduction of 0.87% (s.e. 0.39%) – analogous to Figure 5, which reports large contributions for single auxiliary disease categories but small reductions when removing a single auxiliary disease category. We have updated the *Application to 15 diseases from the UK Biobank* subsection of the Results section (p.8), citing new Supplementary Figure 18 and Supplementary Table 28, to report this result.

As noted by the reviewer, there is strong assortative mating on EA. However, this would only impact estimates of genetic correlations with target diseases (and $h^2{}_{pleio}/h^2$) in the case of cross-trait assortative mating (i.e. in which individuals with high/low EA choose mates with high/low disease risk) (Border et al. 2022 *Science*, ref. 57, formerly ref. 50). We believe that cross-trait assortative mating effects involving EA and disease risk are likely to be modest in most cases. We have updated the Discussion section (p.12) to discuss this point.

6. Figure 3: instead of "NA" in the diagonal, consider leaving it blank or, better, displaying heritability estimates.

We have updated Figure 3 by adding liability-scale SNP-heritability estimates in the diagonal squares.

7. Supplementary Figure 20 could easily be Figure 1B.

We have revised Figure 1 by adding Supplementary Figure 20 from the previously submitted version as a new Figure 1B, cited and discussed in the *Overview of estimation of pleiotropic heritability* subsection of the Results section (p.5).

8. - Ethics approval says: "This study utilized publicly available datasets and therefore did not require ethical approval." That's not true, UK Biobank did get ethical approval, and cannot be

used by just anyone, it's not publicly available in that sense. UK Biobank has received ethical approval from the National Health Service North West Centre for Research Ethics Committee (reference: 11/NW/0382). If you get permission to use UKB for your study (which you should), then it falls under this ethical approval.

We thank the reviewer for pointing out UK Biobank did get ethical approval. We have updated the *Ethics statement* subsection of the Methods section (p.14) to note this ethical approval.

9. - The comparison between pleiotropic genetic variance and pleiotropic phenotypic variance is a very interesting result in the paper. I would be curious to hear how the authors would explain this observation.

We thank the reviewer for this comment. We hypothesize that our finding that ${h^2}_{pleio}/h^2$ is generally larger than ${V^2}_{pleio}/V^2$ could be explained by pleiotropic stabilizing selection on complex traits, in which common variants with effects on multiple traits are preferentially retained (Simons et al. 2018 *PLoS Biol*, ref. 43). We have updated the Discussion section (p.11) to discuss these points.

#10 - The bias correction for finite-sample error in genetic correlation estimates is essential and is supported by simulations. Still, the procedure remains a bit opaque. A clearer explanation of why the Monte Carlo approach works, perhaps with a small example or illustration, would help readers unfamiliar with this kind of bias adjustment.

(Also see part (i) of response to Reviewer #3 Introductory comments and part (i) of response to Reviewer #3 Comment 3)
We agree with the reviewer that it is helpful to provide a clearer explanation of why Monte-Carlo sampling works. Monte-Carlo sampling is a widely used method that relies on random sampling to capture properties of a noise distribution (Efron & Tibshirani 1993, An Introduction to the Bootstrap, ref. 30). In our application, Monte-Carlo sampling captures the noise distribution of the noisily estimated genetic correlations; when the sampled genetic correlations are used to estimate ${h^2}_{pleio}/h^2$, the resulting distribution captures the bias in ${h^2}_{pleio}/h^2$ arising from noisily estimated genetic correlations. Specifically, Monte-Carlo sampling is used to estimate the expected ratio between the true value (unbiased) vs. initial estimate (biased) of ${h^2}_{pleio}/h^2$, so that the initial estimate can be scaled by this ratio to obtain an unbiased estimate. We have updated the *Overview of estimation of pleiotropic heritability* subsection of the Results section (p.4) to provide this intuition, and to explain this intuition in the context of our example involving a target disease with true genetic correlation of 0 with each auxiliary disease.

Reviewer #3 (Remarks to the Author):

The authors proposed a method called Pleiotropic Heritability with Bias Correction (PHBC) to estimate pleiotropic heritability. Simulations suggested that PHBC is robust. Applying the method to the UK Biobank, they found pleiotropic heritability to be broadly distributed across disease categories. However, a major concern is that the bias-correction approach appears somewhat ad hoc, and it is unclear whether the method performs well when disease heritability is low ($h^2 = 0.02–0.05$), which is typical for many diseases. It is also uncertain whether the reported conclusions reflect genuine biological signals or are driven by bias.

The reviewer raised three concerns: (i) The bias correction approach is ad hoc; (ii) It is unclear whether the method performs well when disease heritability is low; and (iii) It is unclear whether the conclusions reflect biological signals or bias. We address each of these concerns in turn.

(i) The bias correction approach is ad hoc

(also see response to Reviewer #2 Comment 10 and part (i) of response to Reviewer #3 Comment 3)
We recognize that it is our responsibility to provide a clear exposition. Monte-Carlo sampling is a widely used method that relies on random sampling to capture properties of a noise distribution (Efron & Tibshirani 1993, An Introduction to the Bootstrap, ref. 30). In our application, Monte-Carlo sampling captures the noise distribution of the noisily estimated genetic correlations; when the sampled genetic correlations are used to estimate $h^2{}_{pleio}/h^2$, the resulting distribution captures the bias in $h^2{}_{pleio}/h^2$ arising from noisily estimated genetic correlations. Specifically, Monte-Carlo sampling is used to estimate the expected ratio between the true value (unbiased) vs. initial estimate (biased) of $h^2{}_{pleio}/h^2$, so that the initial estimate can be scaled by this ratio to obtain an unbiased estimate. We have updated the *Overview of estimation of pleiotropic heritability* subsection of the Results section (p.4) to provide this intuition, and to explain this intuition in the context of our example involving a target disease with true genetic correlation of 0 with each auxiliary disease.

(ii) It is unclear whether the method performs well when disease heritability is low

(Also see response to Reviewer #1 Comment 1a and response to Reviewer #3 Comment 4)
We agree that a liability-scale heritability of 0.25 is not reflective of our real data analyses. We have updated the liability-scale heritability in main simulations to 0.13, which is the median across 15 UK Biobank diseases (we also consider values 0.06 and 0.25 in secondary simulations). We have also updated the prevalence in main simulations to 0.10, which is the median across 15 UK Biobank diseases (we also consider values 0.05 in secondary simulations). We have updated Figure 2, Supplementary Figures 1,6-11 and Supplementary Tables 4, 8-10 accordingly. We have updated the *Simulations* subsection of the Results section (p.6, citing Figure 2) and the *Simulations* subsection of the Methods section (p.20) to reflect these changes. These changes did not significantly impact the conclusions of our simulations.

(iii) It is unclear whether the conclusions reflect biological signals or bias

We believe that our empirical conclusions reflect biological signals rather than bias, because simulations show that our PHBC is effective in correcting bias. We have updated the *Simulations* subsection of the Results section (p.6-7) to clarify this point.

Major comments:

1. The definition of h2pleio is unclear. There are different types of pleiotropy (see their ref. #2), and it is not evident what type of pleiotropic variation h2pleio is intended to capture. Based on the formula provided, h2pleio represents the proportion of SNP heritability of a target disease that is jointly explained by a specific set of auxiliary diseases, which could arise from true pleiotropy or from spurious pleiotropy. In the simulation models, the authors assumed an infinitesimal model, meaning that all genetic variants are pleiotropic. Under this assumption, the genetic variation from pleiotropic variants would be identical to the SNP heritability. Therefore, defining h2pleio as "pleiotropic heritability" could be misleading. A different terminology seems more reasonable.

The reviewer has raised two concerns: (i) It is unclear whether the definition of $h^2{}_{pleio}$ includes spurious pleiotropy; (ii) Under the infinitesimal model used in simulations, the genetic variation from pleiotropic variants would be identical to SNP-heritability, such that the term "pleiotropic heritability" could be misleading. We respond to each of these concerns in turn.

(i) It is unclear whether the definition of $h^2{}_{pleio}$ includes spurious pleiotropy

We recognize that it is our responsibility to provide a clear exposition. According to Solovieff et al. 2013 *Nat Rev Genet* (ref. 2), spurious pleiotropy can arise due to phenotype definition (ascertainment bias, phenotypic misclassification, or shared controls) or due to LD involving different causal variants for two traits. We consider each of these possibilities in turn:

Phenotype definition (ascertainment bias, phenotypic misclassification, or shared controls): Definition and estimation of $h^2{}_{pleio}$ is with respect to a specific phenotype definition in a specific cohort. Ascertainment bias will carry through to our definition and estimation of $h^2{}_{pleio}$; in particular, UK Biobank is known to be subject to participation bias (Benonisdottir et al. 2023 *Nat Genet*, ref. 59; Schoeler et al. 2023 *Nat Hum Behav*, ref. 60). Phenotypic misclassification will also carry through to our definition and estimation of $h^2{}_{pleio}$; we have restricted our analysis to individuals in UK Biobank with both primary care data and hospital record data to limit the impact of phenotypic misclassification. Shared controls do not impact definition or estimation of $h^2{}_{pleio}$, because definition is with respect to a population with infinite sample size and cross-trait LD score regression (Bulik-Sullivan*, Finucane* et al. 2015 *Nat Genet*; ref. 8), which we use to estimate genetic correlations, is robust to shared controls in a finite sample. We have updated the *Definition of pleiotropic heritability* subsection of the Results section (p.3) and the limitations paragraph of the Discussion section (p.12) to clarify these points.

LD involving different causal variants for two traits: Genetic correlation can be defined either using correlations across individuals between genetic values, or using correlations across SNPs between causal effect sizes (or best-fit effect sizes, in the case of missing causal SNPs).

Importantly, LD between causal variants (e.g. different causal variants for two traits) causes these two definitions to be not equivalent; we thank the reviewer for drawing our attention to this important point. We have thus made 2 changes to the manuscript. First, we have provided a second definition of pleiotropic heritability using per-SNP causal effect sizes, illustrated in a new Equation 2, cited and discussed in the *Definition of pleiotropic heritability* subsection of the Results section (p.3-4). Second, we now discuss the correspondence between the definition based on per-individual genetic values and the definition based on per-SNP causal effect sizes, and the conditions under which these are equivalent: *Definition of pleiotropic heritability* subsection of the Results section (p.3-4) and *Definition of* ${h^2}_{pleio}$ subsection of the Methods section (p.14-15).

(ii) Under the infinitesimal model used in simulations, the genetic variation from pleiotropic variants would be identical to SNP-heritability, such that the term "pleiotropic heritability" could be misleading

(Also see part (iii) of response to Reviewer #3 Comment 3)
We now set the proportion of causal SNPs to 5% in main simulations (resp. 1% in secondary simulations). This change did not significantly impact the conclusions of our simulations. We have updated the Simulations subsection of the Results section (p.6), citing updated Figure 2 and Supplementary Figures 1-3 and Supplementary Tables 4, 11.

If all traits have infinitesimal genetic architectures with uncorrelated effects, then all variants are pleiotropic and the heritability contributed by all pleiotropic variants is equal to SNP-heritability, whereas genetic correlations and "pleiotropic heritability" (as we have defined it) are equal to 0; specifically, the *variants* are all pleiotropic but the *heritability* of a given trait is not pleiotropic. We have updated the *Definition of pleiotropic heritability* subsection of the Results section (p.4) to clarify that our definition of "pleiotropic heritability" is different from the heritability contributed by all pleiotropic variants. The question of whether "pleiotropic heritability" is a good choice of name for the quantity that we have defined and estimated is a semantic question, not a scientific one. We were unable to come up with a better name for this quantity, but are open to the possibility of choosing a different name, at the preference of reviewers and editors.

2. It is unclear what new insights can be gained from estimating h2pleio over the genetic correlation. It is well established that many diseases share common risk factors and biological pathways, and therefore share heritability, which can also be represented through genetic correlation. As the authors demonstrated, including additional diseases can substantially alter the h2pleio/h2 estimate. Consequently, the magnitude of h2pleio/h2 is highly dependent on the number and selection of diseases or traits included in the analysis.

The reviewer has raised two concerns: (i) It is unclear what new insights can be gained from estimating ${h^2}_{pleio}$ as opposed to genetic correlations; and (ii) The value of ${h^2}_{pleio}/h^2$ is strongly dependent on the set of auxiliary traits. We address each of these concerns in turn.

(i) It is unclear what new insights can be gained from estimating ${h^2}_{pleio}$ as opposed to genetic correlations

We agree that it is of interest to investigate what new insights can be gained from $h^2{}_{pleio}/h^2$ as opposed to genetic correlation. We thus performed a new analysis in which we compared $h^2{}_{pleio}/h^2$ to the sum of (bias-corrected) $r_g{}^2$ across auxiliary diseases, for different target diseases and auxiliary disease sets. For each of 15 UK Biobank target diseases, instead of considering 2^{14} auxiliary disease sets (based on subsets of the remaining 14 auxiliary diseases), we considered 14 auxiliary disease sets (containing 1, …, 14 auxiliary diseases), defined by starting with the auxiliary disease with highest $r_g{}^2$ with the target disease and iteratively adding auxiliary diseases in order of decreasing $r_g{}^2$ with the target disease; we then computed the correlation between $h^2{}_{pleio}/h^2$ and the sum of (bias-corrected) $r_g{}^2$ across auxiliary diseases, across the 14 auxiliary disease sets. The average correlation (across 15 target diseases) was 0.28, implying that $h^2{}_{pleio}/h^2$ captures different information than the sum of (bias-corrected) $r_g{}^2$. We have updated the *Application to 15 diseases from the UK Biobank* subsection of the Results section (p.9, citing Supplementary Table 30-31) to report this result.

We note that pleiotropic heritability could be applied to high-dimensional auxiliary traits, such as brain imaging traits in studies of psychiatric disease (for which summing a large set of genetic correlations may be even less informative). We have updated the *Discussion* section (p.12).

(ii) The value of $h^2{}_{pleio}/h^2$ is strongly dependent on the set of auxiliary traits

(Also see response to Reviewer #2 Comment 3)
We agree with the reviewer that the definition of pleiotropic heritability is with respect to a specific set of auxiliary traits, and that the results are strongly dependent on the set of auxiliary traits. We recognize that it is our responsibility to provide a clear exposition. We have updated the *Definition of pleiotropic heritability* subsection of the Results section (p.3) and the *Definition of* $h^2{}_{pleio}$ subsection of the Methods section (p.14) to clarify these points.

We have also updated the *Application to 30 diseases from publicly available GWAS meta-analyses* subsection of the Results section (p.9-10) to discuss in more detail how pleiotropic heritability increased with the inclusion of additional auxiliary traits. In particular, we note that average $h^2{}_{pleio}/h^2$, e.g. across 30 non-UK Biobank diseases, tends to increase and then plateau as the number of auxiliary traits increases (e.g. 35% (s.e. 3%) for all 45 auxiliary diseases to 37% (s.e. 2%) for all 62 auxiliary diseases and quantitative traits) (Fig 6B).

3. The proposed bias correction for estimating h2pleio/h2 relies on a strong assumption that the true value is simply a scaled version of the initial estimate. It is unclear why this relationship should hold in general, or why the bias would necessarily be upward. In the simulations, only the infinitesimal model was considered. Could the observed upward bias be a consequence of this model choice? If the infinitesimal model does not hold, it is uncertain whether the same bias pattern or the validity of the correction would persist.

The reviewer has raised three concerns: (i) It is unclear why (an unbiased estimate of) the true value can be obtained by applying a scalar factor to the initial estimate; (ii) It is unclear why the bias would necessarily be upward; and (iii) It is unclear whether the bias correction is valid when the infinitesimal model does not hold. We address each of these concerns in turn.

(i) It is unclear why (an unbiased estimate of) the true value can be obtained by applying a scalar factor to the initial estimate

(Also see response to Reviewer #2 Comment 10 and part (i) of response to Reviewer #3 Introductory comments)
We recognize that it is our responsibility to provide a clear exposition. Monte-Carlo sampling is a widely used method that relies on random sampling to capture properties of a noise distribution (Efron & Tibshirani 1993, An Introduction to the Bootstrap, ref. 30). In our application, Monte-Carlo sampling captures the noise distribution of the noisily estimated genetic correlations; when the sampled genetic correlations are used to estimate $h^2{}_{pleio}/h^2$, the resulting distribution captures the bias in $h^2{}_{pleio}/h^2$ arising from noisily estimated genetic correlations. Specifically, Monte-Carlo sampling is used to estimate the expected ratio between the true value (unbiased) vs. initial estimate (biased) of $h^2{}_{pleio}/h^2$, so that the initial estimate can be scaled by this ratio to obtain an unbiased estimate. We have updated the *Overview of estimation of pleiotropic heritability* subsection of the Results section (p.4) to provide this intuition, and to explain this intuition in the context of our example involving a target disease with true genetic correlation of 0 with each auxiliary disease.

(ii) It is unclear why the bias would necessarily be upward

If only one auxiliary disease is included, $h^2{}_{pleio}/h^2$ is equivalent to r_g^2. We now clarify in the *Overview of estimation of pleiotropic heritability* subsection of the Results section (p.4) that the square of an unbiased estimate of a parameter provides an upward biased estimate of the squared parameter, and provide a mathematical derivation of this in the *Monte Carlo bias correction to initial estimate of* $h^2{}_{pleio}$ subsection of the Methods section (p.19).

If multiple auxiliary diseases are included, we now clarify in the *Overview of estimation of pleiotropic heritability* subsection of the Results section (p.4) that the estimate of $h^2{}_{pleio}/h^2$ will be upward biased if $r_g[\boldsymbol{D},\boldsymbol{D}]^{-1}$ is positive definite (which is likely to be the case), and provide a mathematical derivation of this in the *Monte Carlo bias correction to initial estimate of* $h^2{}_{pleio}$ subsection of the Methods section (p.18).

In simulations, we determined that uncorrected estimates of $h^2{}_{pleio}/h^2$ suffered upward bias (ranging from 1.1% (s.e. 0.12%) to 9.9% (s.e. 0.80%)) across a wide range of simulation settings. We have updated the *Simulations* subsection of the Results section (p.6-7) to clarify this point.

(iii) It is unclear whether the bias correction is valid when the infinitesimal model does not hold

(Also see part (ii) of response to Reviewer #3 Comment 1)
We now set the proportion of causal SNPs to 5% in main simulations (resp. 1% in secondary simulations). This change did not significantly impact the conclusions of our simulations. We have updated the Simulations subsection of the Results section (p.6-7), citing updated Figures 2 and Supplementary Figures 1-3 and Supplementary Tables 4, 11.

4. The simulations were conducted assuming a liability-scale heritability of 0.25, whereas

disease heritability is typically much lower (h2<0.13; for more than half of the 15 diseases in Supplemental Table 1, h2<0.05). Therefore, it is not convincing that the good performance of PHBC in simulations will necessarily translate to real data, as the bias in h2pleio/h2 could be inflated when h2 is small.

(Also see response to Reviewer #1 Comment 1a and part (ii) of response to Reviewer #3 Introductory comments)
We agree that a liability-scale heritability of 0.25 is not reflective of our real data analyses. We have updated the liability-scale heritability in main simulations to 0.13, which is the median across 15 UK Biobank diseases (we also consider values 0.06 and 0.25 in secondary simulations). We have also updated the prevalence in main simulations to 0.10, which is the median across 15 UK Biobank diseases (we also consider value of 0.05 in secondary simulations). We have updated Figure 2, Supplementary Figures 1,6-11 and Supplementary Tables 4, 8-10 accordingly. We have updated the *Simulations* subsection of the Results section (p.6-7, citing Figure 2) and the *Simulations* subsection of the Methods section (p.20-21) to reflect these changes. These changes did not significantly impact the conclusions of our simulations.

5. The claim that estimates of h2pleio/h2 were generally larger than those of V2pleio/V2 may also be explained by the bias in estimating h2pleio/h2 when h2 is small. For example, in hypertension (HTN), which has the largest h2 among the 15 diseases, the estimate of h2pleio/h2 was much closer to the estimate of V2pleio/V2 (Figure 7).

The reviewer has raised two concerns: (i) Estimates of $h^2{}_{pleio}/h^2$ may be upward biased when h^2 is small; and (ii) estimates of $h^2{}_{pleio}/h^2$ may be inflated relative to estimates of $V^2{}_{pleio}/V^2$ when h^2 is small. We address each of these concerns in turn.

(i) Estimates of $h^2{}_{pleio}/h^2$ may be upward biased when h^2 is small

(Also see part (ii) of response to Reviewer #3 Introductory comments and response to Reviewer #3 Comment 4)
In simulations with liability-scale h^2=0.13, we observed an average bias of -0.0020 (s.e. 0.0011) in bias-corrected estimates of $h^2{}_{pleio}/h^2$ (Figure 2). In simulations with liability-scale h^2=0.06, we observed an average bias of -0.0171 (s.e. 0.0023) in bias-corrected estimates of $h^2{}_{pleio}/h^2$ (Supplementary Figure 8). Thus, our simulations provide no evidence of upward bias in bias-corrected estimates of $h^2{}_{pleio}/h^2$ when h^2 is small. We have updated the *Simulations* subsection of the Results section (p.6-7), citing Figure 2 and Supplementary Figure 8 and Supplementary Table 9, to clarify this point.

(Also see response to Reviewer #1 Comment 7)
We also assessed whether there is a correlation between liability-scale heritability and $h^2{}_{pleio}/h^2$ across the 15 UK Biobank diseases. We observed no significant correlation (correlation = -0.18 (*P* = 0.52); Supplementary Figure 19). We have updated the *Application to 15 diseases from the UK Biobank* subsection of the Results section (p.8-9, citing Supplementary Figure 19 to report this result.

(ii) Estimates of $h^2{}_{pleio}/h^2$ may be inflated relative to estimates of $V^2{}_{pleio}/V^2$ when h^2 is small

We assessed whether there is a correlation between liability-scale heritability and the ratio of $h^2{}_{pleio}/h^2$ to $V^2{}_{pleio}/V^2$ across the 15 UK Biobank diseases. We observed a negative correlation (correlation = -0.65, *P* = 0.008; Supplementary Figure 32). The p-value of 0.008 is anti-conservative, as it treats the 15 diseases as independent when they are in fact correlated, and it is thus unclear whether the correlation is statistically significant. We have updated the *Comparing shared genetic and non-genetic variance* subsection of the Results section (p.11) to report this result. However, per (i) above, there is no upward bias in estimates of $h^2{}_{pleio}/h^2$ when h^2 is small.

6. In the GWAS analysis, LD prune at r2 <0.8 was applied. Will the estimate of h2pleio/h2 also sensitive to this LD pruning?

We agree that it is logical to analyze the data without LD pruning. We now analyze 1.1M UK Biobank HapMap3 SNPs with MAF > 0.01 in both simulations and real data analyses, consistent with recommendations for running cross-trait LD score regression (Bulik-Sullivan*, Finucane* et al. 2015 *Nat Genet*, ref. 8) (whereas we previously analyzed LD-pruned SNPs in both simulations and real data analyses). This change did not significantly impact our results. We have updated Figure 2, 4-7 and Supplementary Figures 1-22, 24-26, 28-32 accordingly. We have updated the *Simulations* subsection of the Results section (p.6-7) and the *Simulations* subsection of the Methods section (p.20-21) to indicate all simulations analyze 1.1M UK Biobank HapMap3 SNPs, and we have updated the *UK Biobank data* subsection of the Methods section (p.21-22) to indicate that all real data analyses analyze 1.1M UK Biobank HapMap3 SNPs.

Response to reviewers for NG-A69103R1 (Zhao et al.)

Reviewer #1:

The authors have been very responsive in their resubmission, with the manuscript including updated simulations that reflect more realistic population generating scenarios and use out-of-sample LD, newly added guidelines around trait power and genetic overlap, and a built-in R package. I very much appreciate their detailed and clear responses. I have one remaining, but important, comment.

We thank the reviewer for suggesting that the resubmission was very responsive. The reviewer comments are addressed in detail below.

1. The previous version of the manuscript described the standard errors as approximately well-calibrated. The resubmission has been updated to clarify that the simulations reveal the standard errors on the Monte-Carlo corrected estimates may be upwardly biased by a ratio of 1.63. This, I think, is exceedingly high and is better described as the standard errors not being appropriately calibrated. To be clear, I recognize that this ratio is never exactly 1, but in my own work and when reviewing other methods manuscripts that report this value, my take is that a reasonable upper limit on this estimate is ~1.1.

(also see response to Reviewer #3 Comment 2)
We thank the reviewer for flagging the issue of calibration of standard errors. After considering this issue carefully, we have decided to change the method that we use to compute s.e. Roughly, we scale the uncorrected jackknife s.e. by the ratio of the s.d. of corrected estimates across Monte-Carlo samples divided by the s.d. of uncorrected estimates across Monte-Carlo samples (instead of scaling the uncorrected jackknife s.e. by the ratio of the corrected estimate divided by the uncorrected estimate, as in our previous submission). We believe that this is a superior approach to computing standard errors. We have thus made three changes to the manuscript.

First, we have updated the *Overview of methods* subsection of the Results section (p.5) and the Methods section (p.20, including new Equation 29 and Equation 30) to describe the new method for computing s.e.

Second, we have updated the *Simulations* subsection of the Results section (p.6, citing updated Supplementary Figure 1) to report the calibration of s.e. using the new method for computing s.e. Reassuringly, the ratio of estimated vs. true errors is reasonably close to 1 (1.07 or 0.95 or 1.27 or 0.93, varying with four ways to compute this ratio; details in Supplementary Figure 1 caption). Based on the reviewer comment about a reasonable upper limit, we now describe our s.e. as "approximately well-calibrated"; we are open to changing this wording if that would be preferred. We note that our updated Supplementary Figure 1 makes clear that no specific simulated trait drives the slight biases in our new s.e. We agree with the reviewer that the ratio of 1.63 in our prior resubmission was exceedingly high (see response to Reviewer #1 Comment 2).

Third, we have updated per-trait s.e., s.e. of average estimate across traits (details in Methods section, p.20), and assessments of statistical significance in all analyses of empirical disease data.

We generally observed moderate increases in s.e., e.g. the s.e. on our estimate of 27% for average $h^2{}_{pleio}/h^2$ across 15 UK Biobank diseases increased from 2.0% to 3.0%.

2. As the authors state in the note of Supplementary Figure 1, comparison of Panels A and B shows that the SEs from the block jackknife are anti-conservative before correction and conservative after applying the Monte-Carlo bias correction. The upward bias in SE after the correction seems to be largely driven by two scenarios that are much further off the dashed line. Is it possible something strange happened with that set of specific simulation runs?

We thank the reviewer for flagging the issue of calibration of standard errors. We agree with the reviewer that the ratio of 1.63 in our prior resubmission was exceedingly high. We in fact determined that this was due to a bug in our code for aggregating simulated traits into categories. We regret that bug, which we have now fixed. Results for our new method of computing s.e. are described above (see response to Reviewer #1 Comment 1).

For maximum transparency, we would like to share corrected results for our old method of computing s.e. with reviewers (even though they are not included in the current resubmission). The ratio of estimated vs. true errors was not satisfactorily close to 1 (0.84 or 0.78 or 0.76 or 0.68, varying with four ways to compute this ratio described in Supplementary Figure 1 caption). Thus, we found the new method of computing s.e. to be superior.

3. I also note that they compare the squared block jackknife standard errors to the squared deviation (squared difference between post-corrected and true value). In my experience, the standard errors are typically compared to the standard deviation of the sampling distribution of estimates obtained from simulation (not the deviation from the true value), which may produce a ratio more in-line with what I'm used to seeing? I don't need the authors to address my speculations about what may be going on (I pose them here only in case useful), but I do ask that more is done to figure out why the standard errors currently appear to be exceedingly conservative as this is obviously critical to the method.

We agree with the reviewer that comparing standard errors to the standard deviation across simulation estimates is an appropriate computation. This computation produced a ratio of 1.07 (also see response to Reviewer #1 Comment 1), such that we now describe our s.e. as "approximately well-calibrated"; we are open to changing this wording if that would be preferred. For maximum transparency, we report four different ratios (1.07 or 0.95 or 1.27 or 0.93) based on four different ways to compute this ratio (details in Supplementary Figure 1 caption).

Reviewer #1 (Remarks on code availability):
The code is well-documented.

We thank the reviewer for suggesting that the code is well-documented. We have updated our built-in R package (pleioh2g) (cited in the Code Availability section, p.13) to incorporate our new method of computing s.e.

Reviewer #2 (Remarks to the Author):

I am happy with the way the authors have addressed my previous comments. The revisions improve conceptual clarity, especially regarding the dependence of pleiotropic heritability on the chosen auxiliary trait set, and the expanded simulations and clarifications strengthen the methodological presentation. Overall, this is a valuable and timely contribution to the literature on multivariate genetic architectures.

We thank the reviewer for the positive comments about the revisions and the overall contribution to the literature.

I have only one remaining suggestion. In the current version, the manuscript describes the increase in h_"pleio" ^2/h^2as “plateauing” when more auxiliary traits are added. Figure 6 does show diminishing returns, but the estimates still rise with each expansion of the auxiliary set, and the current collection includes only a subset of potentially relevant complex traits. I would therefore suggest softening the language from “plateaus” to something along the lines of “shows diminishing increases,” and to note explicitly that further increases are possible if additional (and more diverse) traits are incorporated. This would better reflect the empirical pattern without overstating the convergence.

We thank the reviewer for this suggestion. We have updated this part of the *Application to 30 diseases from publicly available GWAS meta-analyses* subsection of the Results section (p.10-11) to state that average ${h^2}_{pleio}/h^2$ across 30 non-UK Biobank diseases tends to show diminishing increases as the number of auxiliary traits increases, and to explicitly note that further increases are possible if additional auxiliary traits are incorporated. We have also made both of these updates to the Discussion section (p.12-13).

Beyond this minor point, I am satisfied with the revision.

We thank the reviewer for the positive feedback.

Reviewer #3 (Remarks to the Author):

The revised manuscript has improved significantly. However, I still have some concerns I would like to authors to address.

We thank the reviewer for the positive feedback. The reviewer concerns are addressed in detail below.

1. I appreciate that the authors now provide a much clearer definition of h_"pleio"^2 and its interpretation. However, I remain concerned that referring to h_pleio^2 as “pleiotropic heritability” may be misleading. As the authors themselves note, h_pleio^2 = 0 does not imply the absence of pleiotropic variants. Rather, numerous variants may still influence multiple disease traits. However, I do not have a better name.

The reviewer makes a good point that the term “pleiotropic heritability” may be misleading. After careful consideration, we have decided to change the name of the estimand throughout the manuscript to “pleiotropic shared heritability”, highlighting that we are quantifying *shared* pleiotropic variance rather than the variance of all pleiotropic variants.

2. I previously raised concerns about the proposed bias-correction approach, particularly the strong assumption that the true value is a scaled version of the initial estimate. This concern remains unresolved. I am not opposed to the use of Monte Carlo sampling; rather, I question whether this scaling strategy can truly yield an unbiased estimate of (h_pleio^2)/h^2. Although I agree with the authors that the post bias correction estimates show reduced absolute bias, as demonstrated in ST4–10, the remaining bias is still substantial. For example, when testing whether there is no zero pleiotropic heritability in the zero_trait simulations, ST4–10 shows a markedly inflated type I error rate.

(also see response to Reviewer #1 Comment 2)
The reviewer is correct that Supplementary Tables 4-10 of our last resubmission showed an inflated type I error rate for zero_trait simulations. After considering this issue carefully, we have decided to change the method that we use to compute s.e. Roughly, we scale the uncorrected jackknife s.e. by the ratio of the s.d. of corrected estimates across Monte-Carlo samples divided by the s.d. of uncorrected estimates across Monte-Carlo samples (instead of scaling the uncorrected jackknife s.e. by the ratio of the corrected estimate divided by the uncorrected estimate, as in our previous submission). We believe that this is a superior approach to computing standard errors. We have thus made three changes to the manuscript.

First, we have updated the *Overview of methods* subsection of the Results section (p.5) and the Methods section (p.20, including new Equation 29 and Equation 30) to describe the new method for computing s.e.

Second, we have updated the *Simulations* subsection of the Results section (p. 6, citing updated Supplementary Figure 1 and updated Supplementary Tables 4-10) to report the calibration using the new method for computing s.e. Reassuringly, the type I error rate for zero_trait simulations is

no longer inflated (ranging from 0.00 to 0.01 in main simulation and from 0.00 to 0.04 in secondary simulations). We believe that this addresses the reviewer concern, which pertains to the simulation results rather than the use of Monte Carlo sampling.

Third, we have updated per-trait s.e., s.e. of average estimate across traits (details in *Methods* section, pp.20), and assessments of statistical significance in all analyses of empirical disease data. We generally observed moderate increases in s.e., e.g. the s.e. on our estimate of 27% for average $h^2{}_{pleio}/h^2$ across 15 UK Biobank diseases increased from 2.0% to 3.0%.

3. Since UK Biobank has been reported of substantial population structure, it is not clear whether the estimate of (h_pleio^2)/h^2 also included the effect by population structure.

We thank the reviewer for raising the issue of how population structure may impact our results. We note there are two types of population structure: (i) population structure impacting a single trait, which inflates GWAS chi-square statistics of that trait, and (ii) population structure that jointly impacts two traits, which inflates or deflates the products of z-scores for those two traits. The first type of population structure is captured by the intercept of LD score regression applied to a single trait (Bulik-Sullivan et al. 2015a Nat Genet; ref. 40), aiming to prevent inflation in single-trait SNP-heritability estimates. The second type of population structure is captured by the intercept of cross-trait LD score regression applied to two traits, aiming to prevent inflation or deflation in genetic covariance estimates (Bulik-Sullivan*, Finucane* et al. 2015b Nat Genet; ref. 8). Although these methods are designed to prevent population structure from impacting their results, we nonetheless believe it to be of high interest to assess both (i) and (ii), using the intercepts of LD score regression and intercepts of cross-trait LD score regression.

First, the average single-trait intercept across 15 UK Biobank diseases was 1.02 (s.d. = 0.02), with an average attenuation ratio (the ratio of inflation in intercept to inflation in chi-square statistics; Loh et al. 2018 Nat Genet, ref. 41) of 0.11 (s.d. = 0.09), implying a limited impact of population structure and other confounders on GWAS chi-square statistics for a single trait. Second, noting that the cross-trait intercept is known to be impacted by sample overlap, we compared empirical cross-trait intercepts (impacted by sample overlap + population structure) to analytical cross-trait intercepts (impacted by sample overlap only) across 105 pairs of UK Biobank diseases. We found these to be concordant, with average empirical cross-trait intercept of 0.0461 (s.d. = 0.0464), average analytical intercept of 0.0520 (s.d. = 0.0522), and average difference of −0.0059 (s.d. = 0.0161), implying a limited impact of population structure and other confounders on products of z-scores for two traits. We have updated the *Application to 15 diseases from the UK Biobank* subsection of the Results section (p.9-10), citing new/updated Supplementary Tables 27,32, to report these findings and their interpretation.

4. It appears that the results in the revised manuscript differ substantially from those in the previous submission. For example, the current manuscript reports that h_pleio^2/h^2 is 1.51× larger than the proportion of liability-scale total phenotypic variance explained by the same set of auxiliary diseases, compared with 2.4× in the earlier version. Although the authors attribute some of these changes to phenotype misclassification, the magnitude of the differences seems quite large.

The reviewer is correct that some point estimates changed substantially, due to our updated SNP set and update to UK Biobank disease phenotypes due to prior phenotype misclassification. As noted on p.1 of our prior response to reviewers, our estimate of average $h^2{}_{pleio}/h^2$ for 15 UK Biobank diseases changed from 0.34 (s.e. 0.07) to 0.27 (s.e. 0.02) (P=0.46 for difference), and our estimate of the average ratio of $h^2{}_{pleio}/h^2$ to $V^2{}_{pleio}/V^2$ changed from 2.4x (s.e. 0.52) to 1.51x (s.e. 0.16) (P=0.17 for difference). (We note that the P-values of 0.46 and 0.17 are slightly different from the P-values of 0.32 and 0.07 that we reported in our previous response to reviewers, due to a change in the method that we use to compute s.e. for $h^2{}_{pleio}/h^2$ estimates; see response to response to Reviewer #1 Comment 2 and Reviewer #3 Comment 2.) We have updated Figure 7 and Supplementary Table 26 to reflect the resulting updates to s.e. In addition, we note here that our estimate of $V^2{}_{pleio}/V^2$ changed from 0.14 (s.e. 0.0017) to 0.18 (s.e. 0.0023) ($P < 1 \times 10^{-16}$ for difference). We agree that these changes are large in magnitude (although only the change in $V^2{}_{pleio}/V^2$ was statistically significant). For maximum transparency, we have quantified each of these changes for each disease in turn, as reported in the table below. We determined that, for individual traits, some old vs. new values of ratio of $h^2{}_{pleio}/h^2$ to $V^2{}_{pleio}/V^2$ changed substantially, but no changes were significant at P<0.05/15.

Table R0 (not included in manuscript)**.** Changes in $h^2{}_{pleio}/h^2$ and $V^2{}_{pleio}/V^2$ and (ratio of $h^2{}_{pleio}/h^2$ to $V^2{}_{pleio}/V^2$) for each of 15 UK Biobank diseases in 06/11/25 vs. 11/11/25 submissions.

**Disease**	$\boldsymbol{h^2{}_{pleio}/h^2}$ **old, new** (P-val for difference)	$\boldsymbol{V^2{}_{pleio}/V^2}$ **old, new** (P-val for difference)	**(Ratio of** $\boldsymbol{h^2{}_{pleio}/h^2}$ **to** $\boldsymbol{V^2{}_{pleio}/V^2}$**) old, new** (P-val for difference)
Hypothyroidism	1.2×10^{-2}, 2.2×10^{-2} (P = 0.85)	3.9×10^{-2}, 5.8×10^{-2} ($P = 4.4\times 10^{-3}$)	0.31, 0.37 (P = 0.95)
Type 2 diabetes	0.65, 0.45 (P = 0.39)	0.20, 0.35 ($P < 10^{-16}$)	3.29, 1.30 (P = 0.093)
Obesity	0.54, 0.49 (P = 0.70)	0.13, 0.20 ($P = 1.4\times 10^{-13}$)	4.23, 2.45 (P = 0.072)
Depression	0.71, 0.44 (P = 0.072)	0.12, 0.16 ($P = 1.4\times 10^{-5}$)	5.98, 2.74 (P = 0.010)
Tobacco use disorder	0.47, 0.36 (P = 0.59)	0.12, 0.14 (P = 0.1)	3.79, 2.58 (P = 0.46)
Hypertension	0.39, 0.36 (P = 0.77)	0.41, 0.44 ($P = 4.1\times 10^{-3}$)	0.95, 0.83 (P = 0.63)
Coronary atherosclerosis	0.43, 0.33 (P = 0.42)	0.37, 0.38 (P = 0.44)	1.15, 0.86 (P = 0.39)
Atrial fibrillation	2.7×10^{-2}, 0.10 (P = 0.60)	0.19, 0.24 ($P = 1.0\times 10^{-4}$)	0.14, 0.41 (P = 0.64)
Varicose veins	7.9×10^{-2}, 5.3×10^{-2} (P = 0.88)	4.1×10^{-2}, 4.6×10^{-2} (P = 0.39)	1.93, 1.15 (P = 0.86)
Respiratory diseases	0.60, 0.53 (P = 0.75)	0.11, 0.16 ($P = 1.3\times 10^{-9}$)	5.48, 3.32 (P = 0.31)
GERD	0.50, 0.54 (P = 0.65)	0.15, 0.16 (P = 0.062)	3.33, 3.31 (P = 0.97)
Inguinal hernia	2.0×10^{-2}, 9.3×10^{-2} (P = 0.41)	6.4×10^{-2}, 0.11 ($P = 2.4\times 10^{-7}$)	0.31, 0.87 (P = 0.55)
Diverticulosis	0.21, 0.16 (P = 0.81)	7.0×10^{-2}, 0.14 ($P < 10^{-16}$)	2.96, 1.14 (P = 0.53)
Bunion	2.3×10^{-2}, 2.7×10^{-2} (P = 0.96)	4.9×10^{-2}, 6.8×10^{-2} ($P = 7.1\times 10^{-3}$)	0.47, 0.40 (P = 0.96)
Radiculitis	0.44, 0.15 (P = 0.31)	6.8×10^{-2}, 7.4×10^{-2} (P = 0.3)	6.38, 2.02 (P = 0.30)
Avg of 15 diseases	0.34, 0.27 (P = 0.46)	0.14, 0.18 ($P < 10^{-16}$)	2.39, 1.51 (P = 0.17)

Finally, we would like to disclose 2 additional small changes to our results:

First, we have fixed a bug in identifying trait pairs with large absolute genetic correlation in our pruning procedure (we previously pruned trait pairs with large positive genetic correlation, instead of pruning trait pairs with large absolute genetic correlation). We regret the error. The impact of this fix was extremely small, e.g. $h^2{}_{pleio}/h^2$ estimates for
15 UK Biobank diseases wrt 15 UK Biobank diseases was unchanged at 27.25%;
15 UK Biobank diseases wrt 30 non-UK Biobank diseases was unchanged at 42.36%;
15 UK Biobank diseases wrt all 45 auxiliary diseases was unchanged at 44.21%;
15 UK Biobank diseases wrt all 62 auxiliary traits changed from 48.58% to 48.09%;
30 non-UK Biobank diseases wrt 15 UK Biobank diseases was unchanged at 25.24%;
30 non-UK Biobank diseases wrt 30 non-UK Biobank diseases changed from 30.71% to 30.73%;
30 non-UK Biobank diseases wrt all 45 auxiliary diseases was unchanged at 34.88%;
30 non-UK Biobank diseases wrt all 62 auxiliary traits changed from 37.01% to 37.13%;
We have updated all relevant display items (including main Figure 6), while noting that updates to Figures were not visually discernable in all instances.

Second, we updated our method (and software) to increase the maximum number of Monte Carlo iterations for sampling a non-singular $r_g[\boldsymbol{D}, \boldsymbol{D}]$ matrix from 50 to 200 (3rd criterion for triggering the pruning procedure, Methods section p. 20)—we determined that the previous choice of a maximum of 50 iterations was not large enough, as additional iterations are occasionally helpful. On average, this change would be expected to increase $h^2{}_{pleio}/h^2$ estimates, due to reducing the pruning of auxiliary traits; in practice, due to stochasticity with respect to the set of auxiliary traits, this change actually increased $h^2{}_{pleio}/h^2$ estimates for 3/15 UK Biobank diseases and decreased $h^2{}_{pleio}/h^2$ estimates for 2/15 UK Biobank diseases (w.r.t. all 62 auxiliary traits):
Atrial fibrillation (AF) increased from 36.9% to 39.1%.
Gastroesophageal reflux disease (GERD) increased from 95.7% to 96.5%.
Depression (MDD) increased from 74.4% to 77.0%.
Type 2 diabetes (T2D) decreased from 59.0% to 51.6%.
Hypothyroidism decreased from 21.7% to 16.2%.
Average of 15 UK Biobank diseases decreased from 48.6% to 48.1%.
